# Catalytic (3 + 2) annulation of donor–acceptor aminocyclopropane monoesters and indoles†

**DOI:** 10.1039/d1sc01127h

**Published:** 2021-05-05

**Authors:** Vincent Pirenne, Emma G. L. Robert, Jerome Waser

**Affiliations:** Laboratory of Catalysis and Organic Synthesis, Institut des Sciences et Ingénierie Chimique, Ecole Polytechnique Fédérale de Lausanne Ch-1015 Lausanne Switzerland jerome.waser@epfl.ch

## Abstract

The efficient catalytic activation of donor–acceptor aminocyclopropanes lacking the commonly used diester acceptor is reported here in a (3 + 2) dearomative annulation with indoles. Bench-stable tosyl-protected aminocyclopropyl esters were converted into cycloadducts in 46–95% yields and up to 95 : 5 diastereomeric ratio using catalytic amounts of triethylsilyl triflimide. Tricyclic indoline frameworks containing four stereogenic centers including all-carbon quaternary centers were obtained.

## Abstract 2

A catalytic dearomatization of indoles with D–A aminocyclopropane monoesters for the synthesis of highly substituted indolines.

## Graphical abstract

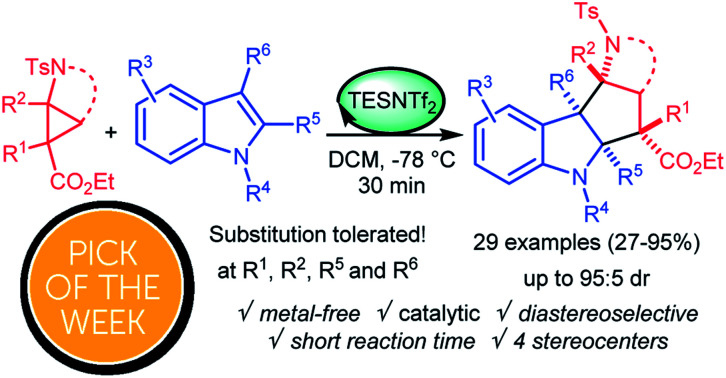

## 1. Introduction

**Scheme 1:**
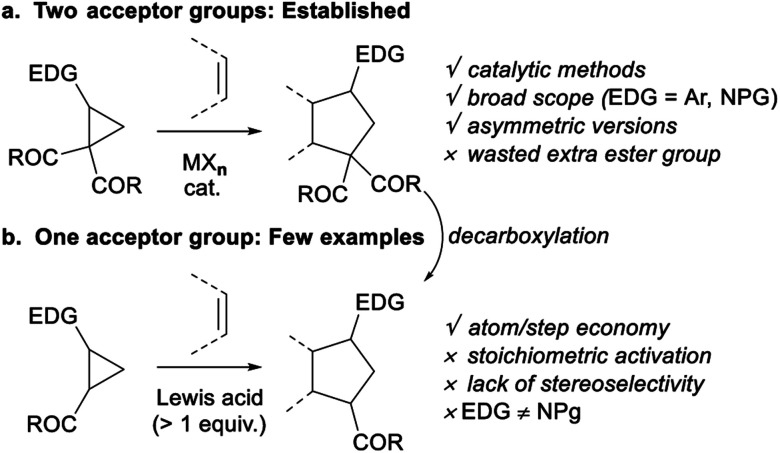
D–A cyclopropanes with one or two acceptor groups in annulation reactions.

Vicinal donor–acceptor (D–A) cyclopropanes are useful three-carbon 1,3-zwitterion synthetic equivalents for the synthesis of carbocyclic scaffolds.^1^ The electronic properties of the donor and acceptor groups are essential to obtain stable yet reactive enough push–pull systems. Dicarbonyl motifs are acceptors of choice for metal-catalyzed ring opening reactions.^1*d*^ Among the many possible transformations, (3 + 2) annulations giving access to five-membered rings are especially useful and have been thoroughly investigated with several donor substituents, with a particular focus on aryl^2^ and protected amines^3^ (Scheme 1a). Enantioselective methods have also been reported.^4^

In contrast, D–A cyclopropanes with a single carbonyl acceptor have been less studied (Scheme 1b). Such substrates lead to the formation of one more stereocenter and do not require a decarboxylation step to remove the diester group.^5^ However, activation and control over diastereoselectivity is challenging for these less reactive cyclopropanes. Only rare examples of (3 + 2) annulations have been reported, and they are often neither catalytic nor stereoselective.^1*d*,*e*^ Cyclopropanecarbaldehydes were mostly suitable in iminium–enamine catalysis for ring-opening reactions rather than annulations.^6^ Annulation reactions of cyclopropyl ketones were performed using stoichiometric Lewis acids such as SnCl_4_, TiCl_4_, BF_3_·Et_2_O and Me_2_AlCl.^7^ Reactions with less reactive cyclopropyl monoesters are limited to alkoxycyclopropanes using silyl triflates or organoaluminium reagents as stoichiometric activators.^7*a*,8^ Catalytic activation remains limited to ring expansion, intramolecular annulation and spirocyclic D–A cyclopropanes.^9^ Only one catalytic intermolecular (3 + 2) annulation of 2-butoxycyclopropanecarboxylate with silyl enol ethers was described by Ihara and co-workers using bistriflimide, but no stereoselectivity was observed.^10^ Furthermore, in contrast to the numerous reports for annulation of aminocyclopropane diesters,^3^ there is currently no report on the use of aminocyclopropane monoesters in annulation reactions, despite the importance of nitrogen-containing building blocks in synthetic and medicinal chemistry. Phthalimide and succinimide have the appropriate electronic properties in the case of aminocyclopropane diesters,^3^ but are not donating enough when a single ester group is present. With carbamate protecting groups, the ring-opening processes of aminocyclopropane monoesters are limited to hydrolysis and rearrangements.^11^ A carbamate-protected aminocyclopropyl ketone was also showed by our group to react intramolecularly in a formal homo-Nazarov cyclization.^12^

**Scheme 2:**
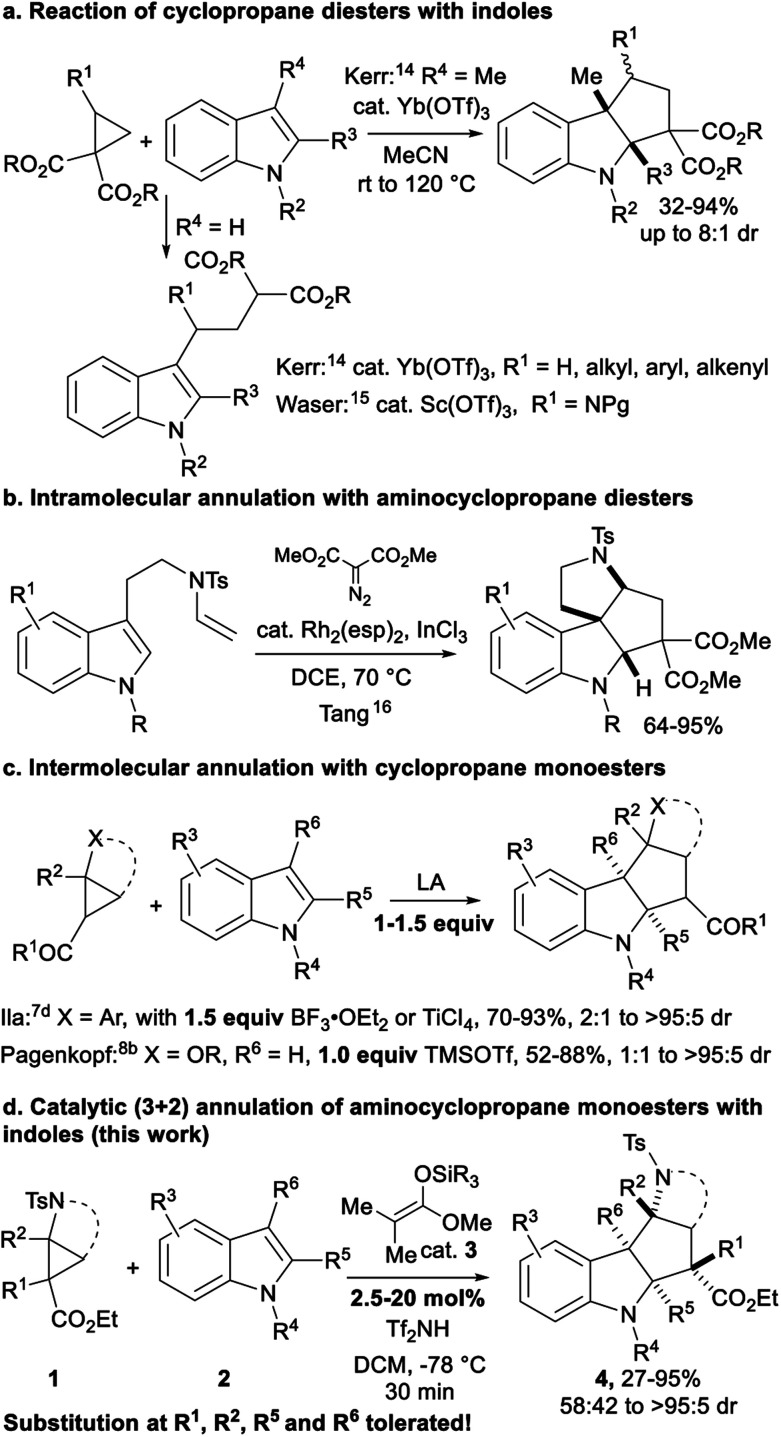
Annulations of D–A cyclopropanes and indoles.

Annulations of D–A cyclopropanes with indole derivatives are particularly interesting, as they provide a quick access to polycyclic indoline scaffolds (Scheme 2). Aminocyclopropanes are especially attractive starting materials, as the obtained indoline-fused cyclopentylamines are present in the core of alkaloid natural products, such as vindolinine, pleiomutinine or huncaniterine A and B.^13^ 3-Methylindoles were used by Kerr and co-workers in a ytterbium triflate catalyzed (3 + 2) annulation with cyclopropane diesters (Scheme 2a).^14^ However, in the absence of substituent at the C-3 position, ring-opening products were obtained. Ring-opening was also observed with aminocyclopropanes by our group.^15^ Ila and co-workers later showed that annulation products can be obtained not only with 3-alkylsubstituted, but also with unsubstituted indoles and arylcyclopropanes, but only using a stoichiometric amount of boron trifluoride etherate as activator.^7*d*^ Recently, Tang and co-workers described the *in situ* formation of unstable tosyl-protected aminocyclopropane diesters and their use in intramolecular annulation with indoles leading to tetracyclic indolines (Scheme 2b).^16^ By comparison, arylcyclopropyl ketones^7*d*^ and alkoxycyclopropyl monoesters^8*b*^ gave annulation products in good yields, but these methods are not catalytic and are limited to the synthesis of tertiary stereocenters at the acceptor position (Scheme 2c). Low diastereoselectivities are obtained for several substitution patterns and in the case of alkoxycyclopropanes, annulation was successful only for C3-unsubstituted indoles.

Herein, we describe the first catalytic (3 + 2) annulation of bench-stable tosyl-protected aminocyclopropane monoesters **1** (Scheme 2d). With indoles **2** as partners, key for success was the use of a silyl bistriflimide as catalyst, generated *in situ* from silyl ketene acetal **3** through protodesilylation.^17,18^ In contrast to previous (3 + 2) annulations of cyclopropane monoesters that all required stoichiometric amounts of Lewis acid,^7*d*,8*b*^ full conversion could be achieved with only 2.5 mol% TESNTf_2_ for several substrates. Furthermore, the method is unique for its tolerance towards substitution patterns, as it works for both C2- and C3-substituted indoles and can be used for the first time to introduce a non-symmetrical all carbon quaternary center at the acceptor position in good yield and high diastereoselectivity.

## 2. Results and discussion

### 2.1. Screening of aminocyclopropanes and optimization

**Scheme 3:**
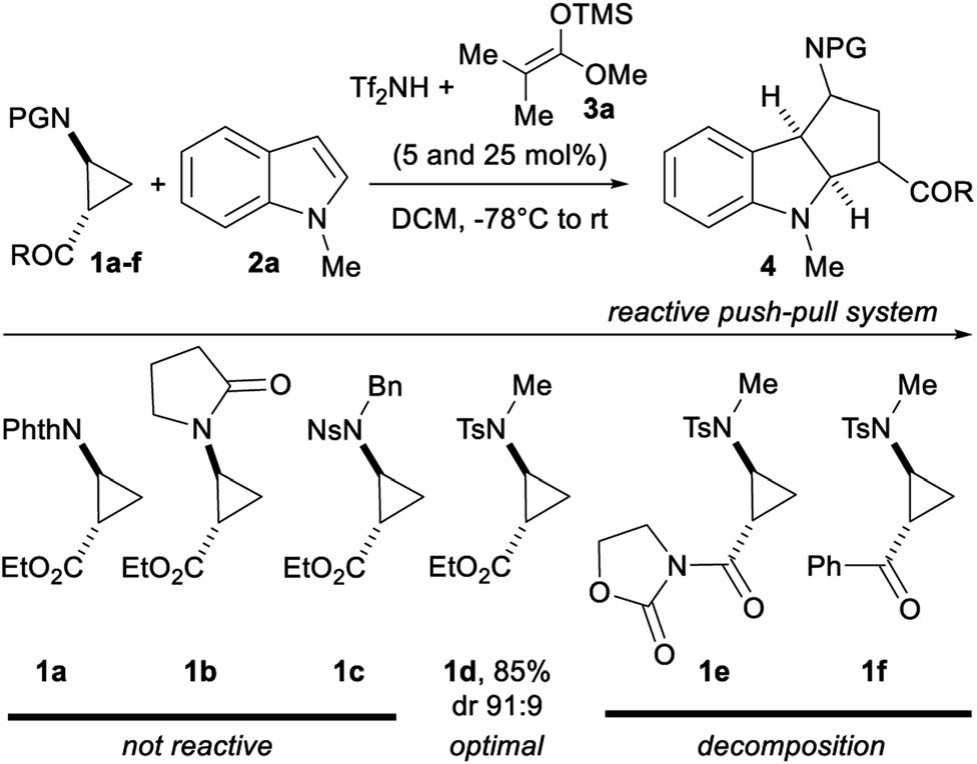
Screening of several push–pull systems for the TMS triflimide-catalyzed (3 + 2) annulation of aminocyclopropanes **1a–f** with 1-methylindole (**2a**).

Our work started with identifying a suitable push–pull system (Scheme 3). Aminocyclopropanes **1a–f** were used in the (3 + 2) annulation with 1-methylindole (**2a**) using Lewis acids as catalysts. Preliminary experiments using TMS triflate led to no reaction (see ESI†), although such conditions have been successful for alkoxycyclopropanes.^8*b*^ Compared to silyl triflates, silyl triflimides have shown superior catalytic activity.^17^ TMS triflimide was formed through the protodesilylation of trimethylsilyl ketene acetal **3a** with bistriflimide.^18^ Although the aminocyclopropyl esters protected by a phthalimide (**1a**), an amide (**1b**) or a nosyl group (**1c**) were not reactive, the tosyl protecting group (**1d**) furnished the cycloadduct **4a** in 85% yield and 91 : 9 dr. The introduction of an oxazolidinone (**1e**) or a ketone (**1f**) as the electron withdrawing group led to stability issues and decomposition in presence of TMS triflimide.

With the optimal D–A aminocyclopropyl ester **1d**, the influence of the substituents on the silicon was examined starting from silyl ketene acetals **3a–f** (Table 1). Using trimethylsilyl ketene acetal **3a** (entry 1), the reaction was completed in less than 30 minutes leading to cycloadduct **4a** in 85% yield and 91 : 9 dr. NOESY experiments allowed the determination of the relative configuration of both isomers (see ESI†). The TES group (**3b**) and other tri-*n*-alkyl silyl groups (**3c** and **3d**) improved the dr to 93 : 7 (entries 2–4). Increasing the bulkiness of the silyl groups (TBS and TIPS, **3e** and **3f**) led to a decrease of the diastereoselectivity (entries 5 and 6). The yield was slightly improved by diminishing the catalyst loading to 2.5 mol% at 0.3 mmol scale without affecting the diastereoselectivity (entry 7). Finally, cycloadduct **4a** was obtained in 87% yield and 92 : 8 dr when the reaction was performed at 0.3 M concentration (entry 8).

**Table 1:** Optimization of the (3 + 2) annulation of aminocyclopropane **1d** with 1-methylindole (**2a**)^a^

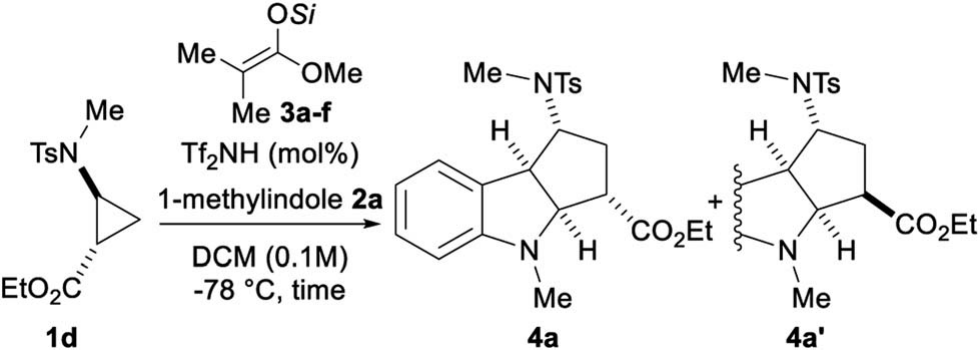					
Entry	Tf_2_NH	Si	Time (min)	Yield^b^ (%)	dr^c^
1	5	SiMe_3_ (**3a**)	30	85	91 : 9
2	5	SiEt_3_ (**3b**)	30	91	93 : 7
3	5	Si-*n*-Pr_3_ (**3c**)	30	87	93 : 7
4	5	Si-*n*-Bu_3_ (**3d**)	30	88	93 : 7
5	5	SiMe_2_*t*Bu (**3e**)	80	90	87 : 13
6	5	Si-i-Pr_3_ (**3f**)	30	91	76 : 24
**7** d	**2.5**	**SiEt**_**3**_ **(** **3b** **)**	**30**	**95**	**93 **:** 7**
8^e^	2.5	SiEt_3_ (**3b**)	30	87	92 : 8

a Reaction conditions: 0.1 mmol scale and 0.1 M, 1.05 equiv. of 1-methylindole (**2a**), 25 mol% of silyl ketene acetals **3a–f**.

b Isolated yield for the mixture of both isomers.

c dr measured from the ^1^H NMR spectrum of the isolated mixture.

d On 0.3 mmol scale.

e On 0.3 mmol scale and at 0.3 M.

### 2.2. Scope of indole derivatives^19^

**Scheme 4:**
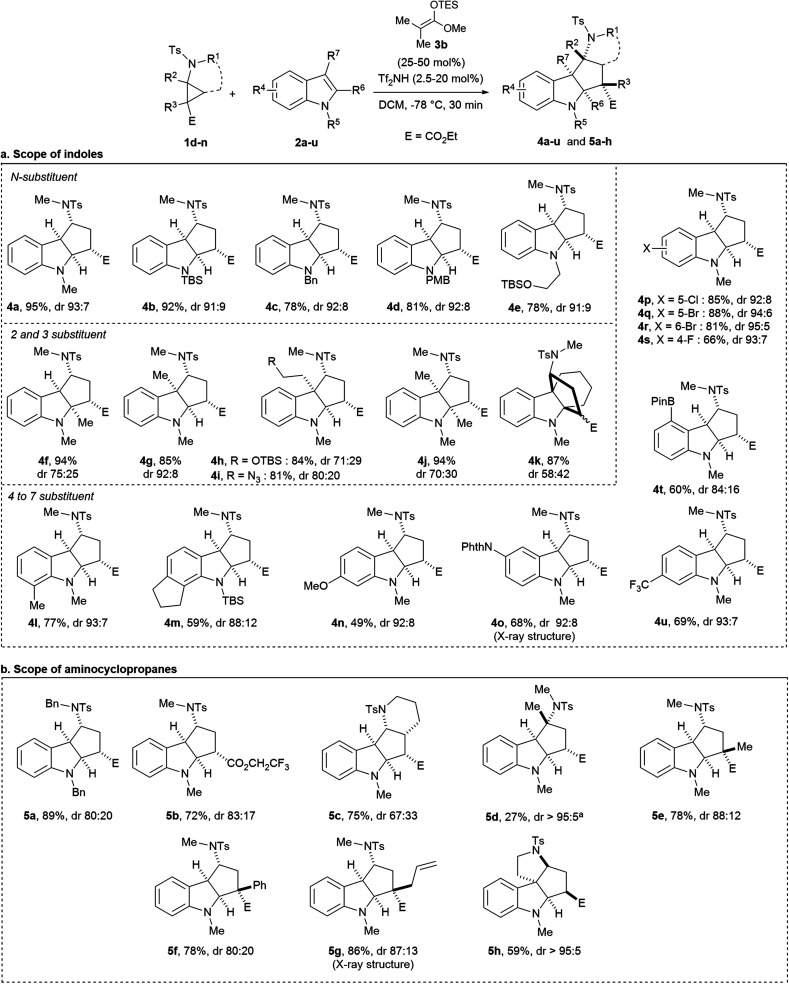
Scope of the catalytic (3 + 2) annulation of tosyl-protected aminocyclopropane **1** with indoles **2** (reaction on 0.1 to 0.3 mmol scale, yields are given for the mixture of both isomers). ^a^Reaction performed at room temperature.

The optimal conditions of entry 7 were then applied to different indole derivatives **2a–u** (Scheme 4a). Indoles **2b–d** protected by a TBS, a benzyl or a PMB group were converted to cycloadducts **4b–d** in 78–92% yield and diastereoselectivities ≥91 : 9. A functionalized *N*-alkyl substituent led to the formation of compound **4e** in similar yield and dr. Free indole gave no reaction. Indoles **2f–k** substituted at the 2 and/or 3-position leading to sterically more encumbered products **4f–k** were successful. For all substitution patterns, annulation products were obtained without ring-opening side reactions. Protected tryptophol and tryptamine gave the desired cycloadducts **4h** and **4i** with a quaternary carbon center in 84%/81% yield and 71 : 29/80 : 20 dr. Excellent yields and diastereoselectivities were obtained for alkyl substituents (**4l** and **4m**), a methoxy (**4n**), a protected nitrogen (**4o**) and halogens (**4p–s**) on the aryl ring. Other functional groups such as pinacol borane and a trifluoromethyl group were also tolerated (**4t** and **4u**). More electron withdrawing substituents such as an ester or a nitrile gave no reaction. X-ray crystal structure analysis of **4o**^20^ confirmed the relative configuration of the cycloadducts. The all-*cis* substituted product was the major diastereoisomer in all cases. The diastereoselectivity decreased with increasing substitution at C2/C3 position (no substituent: 84 : 16–95 : 5, one substituent: 71 : 29–92 : 8, two substituents: 58 : 42–70 : 30).

### 2.3. Scope of aminocyclopropanes

The scope of aminocyclopropanes **1** was then examined (Scheme 4b). These substrates were easily obtained by copper-catalyzed cyclopropanation of the corresponding enamides and diazo compounds.^21^ First, aminocyclopropane **1g** bearing a tosyl and a benzyl on the nitrogen as orthogonal protecting groups afforded cycloadduct **5a** in 89% yield and 80 : 20 dr. Replacement of the ethyl ester (E) by a trifluoroethyl ester group was tolerated (**5b**). More substituted aminocyclopropanes **1i–n** bearing quaternary stereocenters were then prepared. Bicyclo[4.1.0] aminocyclopropane **1i** led to the formation of tetracyclic compound **5c** in 75% yield and 67 : 33 dr. Aminocyclopropane **1j** bearing a fully substituted center at the donor position showed some reactivity only at room temperature, leading to product **5d** in 27% yield and >95 : 5 dr. Aminocyclopropanes fully substituted at the carbon center next to the ester group were more reactive. Alkyl, aryl and allyl substituents led to the formation of (3 + 2) cycloadducts **5e–g** bearing a carbon quaternary center in 78–86% yield and 80 : 20–88 : 12 dr.^18^ To the best of our knowledge, such indoline products bearing a quaternary stereocenter have never been accessed before *via* an annulation of D–A cyclopropanes. When an intramolecular reaction was performed with aminocyclopropane **1n**, the desired product **5h** was obtained in >95 : 5 dr, but with another relative configuration (supported by NOESY experiments, see ESI†), in agreement with the results reported by Tang and co-workers using aminocyclopropane diesters.^16^

**Scheme 5:**
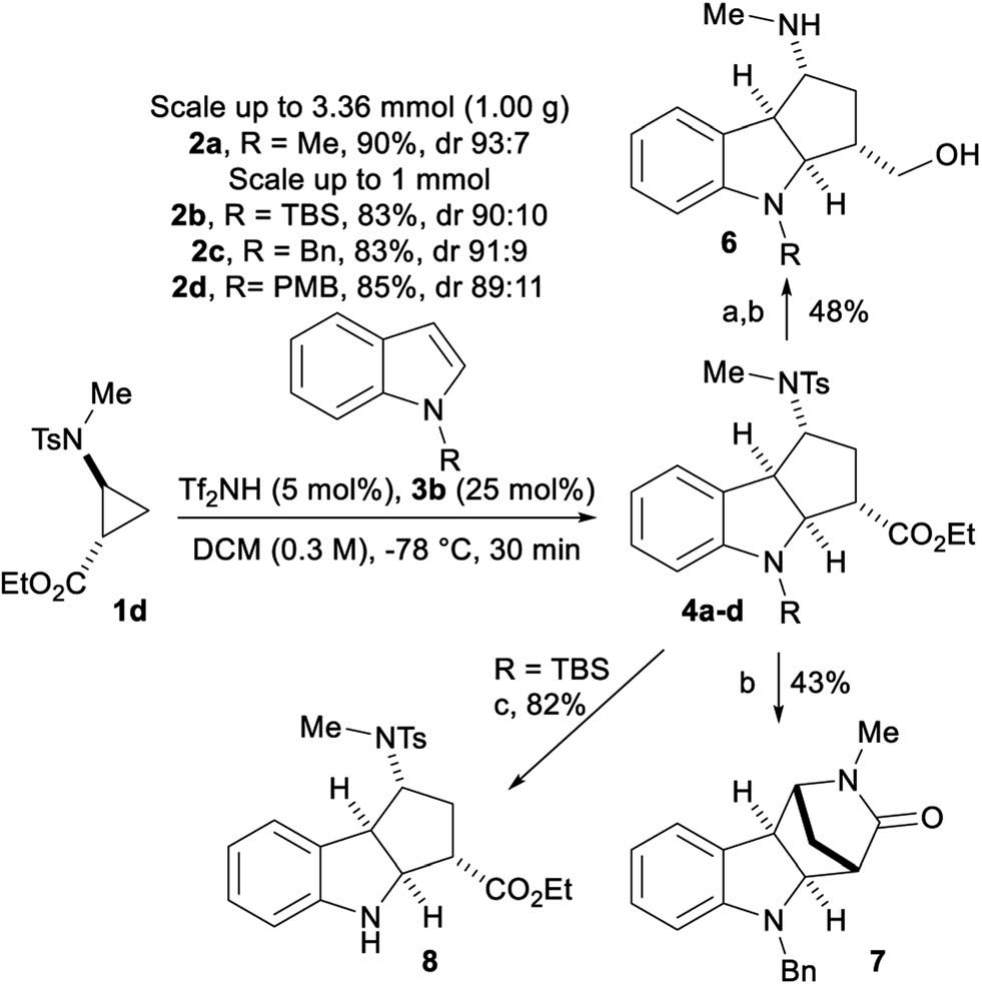
Scale up experiments and product modifications. Reaction conditions: (a) DIBALH, THF, 0 °C; (b) Li/naphthalene, THF, rt; (c) TBAF, THF, 0 °C.

We further performed the reaction with 1 mmol of aminocyclopropane **1d** with protected indoles **2b–d** and obtained similar yields and dr (Scheme 5). With **2a**, a further scale up to 1.00 g (3.36 mmol) was done, giving **4a** in 90% yield and 93 : 7 dr. After reduction of the ester on **4c** with DIBALH, the tosyl group was removed using reductive naphthalene/lithium conditions leading to amino alcohol **6**. Due to the *cis* orientation of the nitrogen and the ester, a bridgehead lactam **7** was produced in 43% yield when tosyl removal was performed directly on **4c**. Finally, the TBS protecting group was removed with TBAF producing free indole **8** in 82% yield. Unfortunately, attempts to epimerize the ester center through enolate formation followed by reprotonation were not successful.

**Scheme 6:**
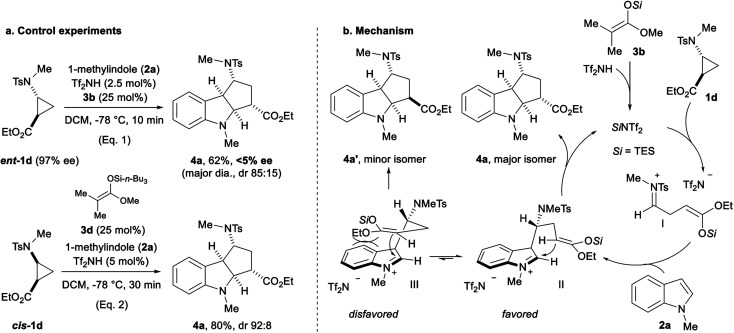
Influence of the absolute and relative configuration of the starting aminocyclopropane **1d** on the (3 + 2) annulation with 1-methylindole (**2a**) (a); speculative mechanism proposal (b).

We then attempted to gain information about the reaction mechanism by starting with enantiopure aminocyclopropane **ent-1d** (Scheme 6a, eqn (1)).^22^ Racemic cycloadduct **4a** was obtained in the TES triflimide-catalyzed (3 + 2) annulation with **2a** (eqn (1)). Moreover, using *cis*-substituted cyclopropane **cis-1d** led to the formation of **4a** with the same diastereoselectivity as observed for *trans*-substituted cyclopropane **1d** (eqn (2)). Considering these results, the formation of an open-chain reactive intermediate is probable (Scheme 6b). The protodesilylation of silyl ketene acetal **3b** produces the active TES triflimide catalyst,^17,18^ which then activates aminocyclopropane **1d** through silylation of the ester. Ring-opening leads to iminium **I**, which is attacked by indole **2a** at the most nucleophilic position to give iminium **II**. A Mannich reaction closes then the ring delivering the (3 + 2) cycloadduct **4a**. The diastereoselectivity is controlled by minimizing steric repulsions between the silyl enol ether and the indole ring (**II***vs.***III**).

## 3. Conclusion

In conclusion, a (3 + 2) annulation reaction of tosyl-protected aminocyclopropane monoesters with indoles catalyzed by triethylsilyl triflimide was disclosed. The tricyclic indoline products were obtained in excellent yields, high degrees of stereoselectivity and short reaction times (less than 30 minutes) with the formation of four stereocenters in one operation, including quaternary centers. The method gives access to complex nitrogen-substituted polycyclic indoline scaffolds of high interest for synthetic and medicinal chemistry.

## Author contributions

V. P. discovered and optimized the reaction, studied the scope, performed the functionalization of the products and the studies on the mechanism, prepared the experimental part and the first draft of the manuscript. E. G. L. R. did the required revisions after the departure of V. P. She performed the scale up of the transformation and further scope extension and functionalization attempts on the products and prepared the related experimental part. J. W. designed the overall research, supervised the work, finalized the manuscript, proofread the experimental part and coordinated the overall project.

## Conflicts of interest

There are no conflicts to declare.

## Supplementary Material

SC-012-D1SC01127H-s001

SC-012-D1SC01127H-s002
