# Supplementary material for: Catalytic (3 + 2) annulation of donor–acceptor aminocyclopropane monoesters and indoles
Source: Chem Sci. 2021 May 5;12(25):8706–12. doi: 10.1039/d1sc01127h (PMC8246098; doi:10.1039/d1sc01127h)
Supplement: SC-012-D1SC01127H-s001 [file SC-012-D1SC01127H-s001.pdf]

Electronic Supplementary Material (ESI) for Chemical Science.
This journal is © The Royal Society of Chemistry 2021

Supporting information

Table of Contents

1. General informations 2
2. Preparation of starting materials and catalysts 3
    2.1. Synthesis of silyl enol ethers 3
    2.2. Synthesis of indole derivatives 5
    2.1. Synthesis of D-A aminocyclopropanes 11
        2.1.1. Synthesis of vinyl sulfonamides 12
        2.1.2. Cyclopropanation of enamides 15
        2.1.3. Synthesis of other aminocyclopropanes (1e, 1f, 1k, 1m) from 1d 21
3. Screening of Lewis acids and optimization 24
4. (3+2) annulation of D-A aminocyclopropanes with indoles 25
    4.1. Scope of indoles 26
    4.2. Scope of aminocyclopropanes 39
    4.3. List of unsuccessful substrates 44
5. Product modifications 44
6. Enantiospecific experiment and chiral HPLC traces 46
7. X-ray crystallographic data 48
    7.1. Compound 4o 48
    7.2. Compound 5g 49
8. References 52
9. NMR spectra 53

1. General informations

All reactions were set up under a nitrogen atmosphere in oven-dried glassware using standard Schlenk techniques, unless otherwise stated. Anhydrous solvents (THF, DCM and toluene) were taken from a commercial SPS solvent dispenser (H_2O content < 10 ppm, Karl-Fischer titration). Anhydrous dimethylformamide, acetonitrile and acetone were purchased from chemical suppliers (Aldrich or Acros Organics). All reagent-grade chemicals were obtained from commercial suppliers (Acros, Aldrich, Fluka, VWR, Aplichem, Fluorochem or Merck) and were used as received unless otherwise stated. Chromatographic purification of products was accomplished using flash chromatography (FC) on SiliaFlash P60 silica gel (230 - 400 mesh). For thin layer chromatography (TLC) analysis, pre-coated TLC sheets ALUGRAM® Xtra SIL G/UV_{254} were employed, using UV light as the visualizing agent and basic aqueous potassium permanganate ($KMnO_4$) stain solutions, and heat as developing agents. The calculated experimental yields refer to chromatographically and spectroscopically (^{1}H-NMR) homogeneous materials unless otherwise stated. Compounds were described as mixtures when it was not possible, in our hands, to separate both compounds.

The NMR spectra were recorded on a Brucker DPX-400 spectrometer (400 MHz for ^{1}H, 101 MHz for ^{13}C and 376 MHz for ^{19}F) at rt using $CDCl_3$ as internal reference unless otherwise indicated. The acidity of deuterated chloroform was neutralized using basic alumina for acid-sensitive compounds, including all cycloadducts. Carbon spectra have been measured using ^{1}H-decoupling. The chemical shift (δ) for ^{1}H and ^{13}C are given in ppm relative to residual signals of the deuterated solvents ($CDCl_3$ - 7.26 ppm ^{1}H NMR and 77.16 ppm ^{13}C NMR; CD_3CN – 1.94 ppm ^{1}H NMR and 118.26 ppm ^{13}C NMR; MeOD 3.31 ppm ^{1}H NMR and 49.0 ppm ^{13}C NMR). The coupling constants (J) are expressed in Hz. The following abbreviations were used to explain the multiplicities: br = broad, s = singlet, d = doublet, t = triplet, td = triplet of doublet, q = quartet, dd = doublet of doublet, ddd = doublet of doublet of doublet, m = multiplet. Infrared spectra were recorded on a JASCO FT-IR B4100 spectrophotometer with an ATR PRO410-S and a ZnSe prisma and are reported as cm^{-1} (w = weak, m = medium, s = strong, br = broad). High resolution mass spectrometric measurements were performed by the mass spectrometry service of ISIC at the EPFL on a MICROMASS (ESI) Q-TOF Ultima API. The diffraction data for crystal structures were collected by mass spectrometry service of ISIC at the EPFL at low temperature using a SuperNova, Dual, Cu at home/near, Atlas diffractometer operating at T = 140.01(10) K. Data were measured using ω scans using Cu K_α radiation. Data reduction, scaling and absorption corrections were performed using CrysAlisPro (Rigaku, V1.171.40.84a, 2020). The structure was solved and the space group determined by the ShelXT[1] structure solution program using dual methods and refined by full matrix least squares minimisation on $\boldsymbol{F^2}$ using version 2018/3 of ShelXL.[2] All non-hydrogen atoms were refined anisotropically. Most hydrogen atom positions were calculated geometrically and refined using the riding model, but some hydrogen atoms were refined freely.

HPLC analysis on chiral stationary phase was performed on a Agilent Acquity instrument using a Daicel CHIRALPAK IA chiral columns.

Raw data for NMR, mass and IR is available at zenodo.org: https://doi.org/10.5281/zenodo.4705362

2. Preparation of starting materials and catalysts

2.1. Synthesis of silyl enol ethers

The silyl ketene acetals **3a-f** were prepared from a reported literature procedure (Scheme S1).[3]

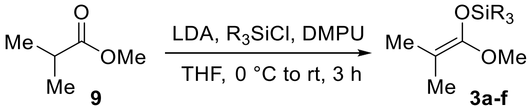

SiR_3 = TMS, TES, TBS, TIPS, Si*n*-Pr_3, Si*n*-Bu_3

Scheme S1. Synthesis of silyl ketene acetals.

General procedure A

To a solution of *i*-Pr_2NH (1.03 equiv.) in dry THF (0.4 M) cooled to 0 °C, *n*-BuLi (2.5 M solution in hexanes, 1.04 equiv.) was added dropwise and the reaction mixture was stirred at rt for 10 min. The reaction was subsequently cooled to 0 ºC and neat methyl isobutyrate **9** (1 equiv.) was added dropwise. The reaction mixture was stirred for 30 min at 0 ºC, followed by the addition of 1,3-dimethyl-3,4,5,6,-tetrahydro-2(1H)-pyrimidinone (DMPU, 2 equiv.) and the corresponding silyl chloride (1.2 equiv.). The reaction was allowed to warm up to room temperature and stirred for 3 h. The reaction mixture was then concentrated in vacuo. Pentane and sat. aqueous $NaHCO_3$ solutions were added to the residue. The aqueous layer was extracted with pentane. The organic layers were further washed with water, sat. $CuSO_4$ solution (3 x), water and brine. After drying over Na_2SO_4 and concentration, the crude product was purified by distillation under reduced pressure to afford the silyl ketene acetals **3a-f** as colorless liquids.

Characterization of silyl ketene acetals

((1-Methoxy-2-methylprop-1-en-1-yl)oxy)trimethylsilane (3a): Prepared according to the general procedure A from diisopropylamine (2.5 mL, 18 mmol, 1.03 equiv.), *n*-BuLi (2.5M, 7.3 mL, 18 mmol, 1.04 equiv.), methyl isobutyrate (2.0 mL, 17 mmol, 1.0 equiv.), DMPU (4.2 mL, 35 mmol, 2.0 equiv.) and chlorotrimethylsilane (2.3 mL, 18 mmol, 1.04 equiv.) in THF (35 mL) for 3 h. The crude oil was purified by distillation (10 mbar, 65 °C) to afford a colorless liquid (1.1 g, 6.3 mmol, 37%).

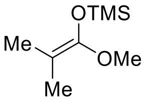

3a

Chemical Formula: $C_8H_{18}O_2Si$
Molecular Weight: 174.3150

^{1}H NMR (400 MHz, $CDCl_3$): δ 3.50 (s, 3H, OC*H*$_3$), 1.57 (s, 3H, C*H*$_3$), 1.52 (s, 3H, C*H*$_3$), 0.20 (s, 9H, TMS).
^{13}C NMR (101 MHz, $CDCl_3$): δ 149.5, 91.0, 56.6, 17.0, 16.2, 0.1.
NMR spectra are in agreement with the reported data.[3]

Triethyl((1-methoxy-2-methylprop-1-en-1-yl)oxy)silane (3b): Prepared according to the general procedure A from diisopropylamine (2.5 mL, 18 mmol, 1.03 equiv.), *n*-BuLi (2.5M, 7.3 mL, 18 mmol, 1.04 equiv.), methyl isobutyrate (2.0 mL, 17 mmol, 1.0 equiv.), DMPU (4.2 mL, 35 mmol, 2.0 equiv.) and TESCl (3.1 mL, 18 mmol, 1.04 equiv.) in THF (35 mL) for 3 h. The crude oil was purified by distillation (1 mbar, 70 – 72 °C) to afford a colorless liquid (2.6 g, 12 mmol, 68%).

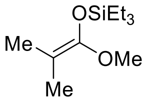

3b

Chemical Formula: $C_{11}H_{24}O_2Si$
Molecular Weight: 216.3960

^{1}H NMR (400 MHz, $CDCl_3$): δ 3.52 (s, 3H, OC*H*$_3$), 1.56 (s, 3H, C*H*$_3$), 1.54 (s, 3H, C*H*$_3$), 0.99 (t, *J* = 7.9 Hz, 9H, TES), 0.69 (q, *J* = 7.9 Hz, 6H, TES).
^{13}C NMR (101 MHz, $CDCl_3$): δ 150.0, 91.1, 57.2, 17.0, 16.3, 6.7, 5.1.
NMR spectra are in agreement with the reported data.[3]

Tri-*n*-propyl((1-methoxy-2-methylprop-1-en-1-yl)oxy)silane (3c): Prepared according to the general procedure A from diisopropylamine (3.2 mL, 23 mmol, 1.2 equiv.), *n*-BuLi (2.5 M, 8.4 mL, 21 mmol, 1.1 equiv.), methyl isobutyrate (2.2 mL, 19 mmol, 1.0 equiv.), DMPU (4.6 mL, 38 mmol, 2.0 equiv.) and *n*-Pr_3SiCl (5.0 mL, 23 mmol, 1.2 equiv.) in THF (47 mL) for 3 h. The crude oil was purified by distillation (1 mbar, 110 °C) to afford a colorless liquid (3.61 g, 13.9 mmol, 73%).

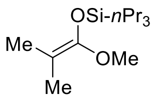

3c
Chemical Formula: $C_{14}H_{30}O_2Si$
Molecular Weight: 258.4770

^{1}H NMR (400 MHz, $CDCl_3$): δ 3.50 (s, 3H, C*H*$_3$), 1.55 (s, 3H, C*H*$_3$), 1.52 (s, 3H, C*H*$_3$), 1.46 – 1.34 (m, 6H, 3xC*H*$_2$), 0.96 (t, *J* = 7.3 Hz, 9H, 3xC*H*$_3$), 0.71 – 0.64 (m, 6H, 3xC*H*$_2$).
^{13}C NMR (101 MHz, $CDCl_3$): δ 150.0, 91.1, 57.2, 18.6, 17.0, 16.7 (2C), 16.3.
IR (ν_{max}, cm^{-1}) 2956 (s), 2924 (s), 1704 (s), 1175 (s), 1064 (s), 840 (s).
HRMS (APPI/LTQ-Orbitrap) m/z: $[M + H]^+$ Calcd for $C_{14}H_{31}O_2Si^+$ 259.2088; Found 259.2090.

Tri-*n*-butyl((1-methoxy-2-methylprop-1-en-1-yl)oxy)silane (3d): Prepared according to the general procedure A from diisopropylamine (2.9 mL, 21 mmol, 1.2 equiv.), *n*-BuLi (2.5 M, 7.7 mL, 19 mmol, 1.1 equiv.), methyl isobutyrate (2.0 mL, 17 mmol, 1 equiv.), DMPU (4.2 mL, 35 mmol, 2.0 equiv.) and Bu_3SiCl (5.6 mL, 21 mmol, 1.2 equiv.) in THF (43 mL) for 3 h. The crude oil was purified by distillation (1 mbar, 160 °C) to afford a colorless liquid (2.8 g, 9.3 mmol, 53%).

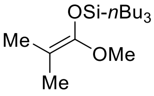

3d
Chemical Formula: $C_{17}H_{36}O_2Si$
Molecular Weight: 300.5580

^{1}H NMR (400 MHz, $CDCl_3$): δ 3.50 (s, 3H, C*H*$_3$), 1.55 (s, 3H, C*H*$_3$), 1.53 (s, 3H, C*H*$_3$), 1.40 – 1.28 (m, 12H, 6xC*H*$_2$), 0.93 – 0.85 (m, 9H, 3xC*H*$_3$), 0.72 – 0.64 (m, 6H, 3xC*H*$_2$).
^{13}C NMR (101 MHz, $CDCl_3$): δ 150.0, 91.2, 26.8, 25.3, 17.1, 16.3, 13.90, 13.87.
IR (ν_{max}, cm^{-1}) 2957 (m), 2923 (m), 2859 (m), 1705 (m), 1173 (s).
HRMS (APCI/QTOF) m/z: $[M + H]^+$ Calcd for $C_{17}H_{37}O_2Si^+$ 301.2557; Found 301.2555.

***tert*-Butyl((1-methoxy-2-methylprop-1-en-1-yl)oxy)dimethylsilane (3e)**: Prepared according to the general procedure A from diisopropylamine (2.5 mL, 18 mmol, 1.03 equiv.), *n*-BuLi (2.5M, 7.3 mL, 18 mmol, 1.04 equiv.), methyl isobutyrate (2.0 mL, 17 mmol, 1.0 equiv.), DMPU (4.2 mL, 35 mmol, 2.0 equiv.) and *tert*-butylchlorodimethylsilane (2.7 g, 18 mmol, 1.04 equiv.) in THF (35 mL) for 3 h. The crude oil was purified by distillation (1 mbar, 75 – 80 °C) to afford a colorless liquid (1.9 g, 8.7 mmol, 50%).

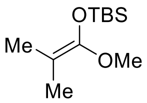

3e
Chemical Formula: $C_{11}H_{24}O_2Si$
Molecular Weight: 216.3960

^{1}H NMR (400 MHz, $CDCl_3$): δ 3.51 (s, 3H, OC*H*$_3$), 1.57 (s, 3H, C*H*$_3$), 1.53 (s, 3H, C*H*$_3$), 0.96 (s, 9H, TBS), 0.14 (s, 6H, TBS).
^{13}C NMR (101 MHz, $CDCl_3$): δ 149.9, 91.5, 57.1, 25.9, 18.2, 17.0, 16.4, -4.5.
NMR spectra are in agreement with the reported data.[3]

Triisopropyl((1-methoxy-2-methylprop-1-en-1-yl)oxy)silane (3f): Prepared according to the general procedure A from diisopropylamine (2.5 mL, 18 mmol, 1.03 equiv.), *n*-BuLi (2.5M, 7.3 mL, 18 mmol, 1.04 equiv.), methyl isobutyrate (2.0 mL, 17 mmol, 1.0 equiv.), DMPU (4.2 mL, 35 mmol, 2.0 equiv.) and chlorotriisopropylsilane (3.9 mL, 18 mmol, 1.04 equiv.) in THF (35 mL) for 3 h. The crude oil was purified by distillation (1 mbar, 100 – 115 °C) to afford a colorless liquid (2.8 g, 10 mmol, 62%).

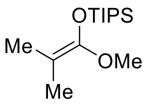

3f

Chemical Formula: $C_{14}H_{30}O_2Si$
Molecular Weight: 258.4770

^{1}H NMR (400 MHz, $CDCl_3$): δ 3.56 (s, 3H, OC*H*$_3$), 1.57 (s, 6H, 2xC*H*$_3$), 1.22 – 1.04 (m, 21H, TIPS).
^{13}C NMR (101 MHz, $CDCl_3$): δ 150.9, 91.2, 58.3, 18.0, 17.2, 16.5, 12.9.
NMR spectra are in agreement with the reported data.[3]

2.2. Synthesis of indole derivatives

The Figure S1 discloses the commercially available 1-methylindole derivatives and the TBS-protected indoles already prepared by our group. The data of **2b** and **2m** are disclosed below and were taken from our original publication.[4]

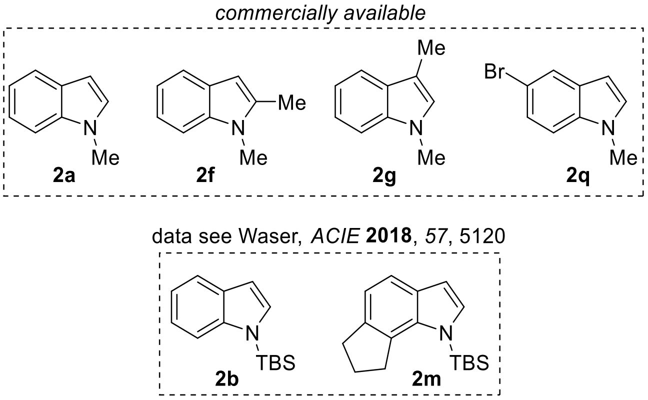

Figure S1. Commercially available 1-methylindole derivatives and TBS-protected indole reported by our group.

Most of the indole derivatives were prepared by alkylation and silylation of the corresponding free indole as depicted in Scheme S2.

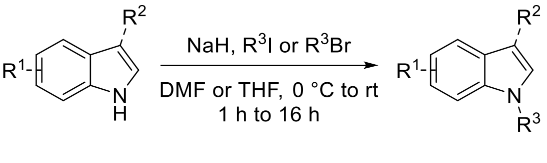

Scheme S2. Alkylation and silylation of indole derivatives.

General procedure (B) for the methylation, benzylation or alkylation of indoles

A solution of indole (1.0 equiv.) in anhydrous DMF or THF (0.3 M) was added dropwise to a stirred suspension of NaH (60% dispersion in mineral oil, 1.2 to 3.0 equiv.) in DMF or THF at 0 ºC. The mixture was allowed to slowly warm up to rt over 15 min, and iodomethane (or iodide or 4-methoxybenzyl bromide) (1.05 to 1.5 equiv.) was added dropwise at 0 °C. The resulting mixture was then allowed to reach rt and stirred for 1 h (or when full conversion was observed by TLC). The

reaction was then quenched at 0 °C with a sat. aqueous solution of NH_4Cl and extracted with diethyl ether or EtOAc. The combined organic layers were washed with water and brine, dried over anhydrous Na_2SO_4, filtered off and concentrated under reduced pressure. The crude indole was then purified by flash chromatography.

Characterization of indole derivatives

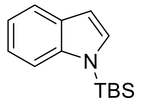

2b

Chemical Formula: $C_{14}H_{21}NSi$
Molecular Weight: 231.4140

1-(*Tert*-butyldimethylsilyl)-1H-indole (2b): Prepared according to the general procedure B from indole (0.58 g, 5.00 mmol, 1.0 equiv), NaH (0.60 g, 15.0 mmol, 3 equiv) *tert*-butylchlorodimethylsilane (0.90 g, 6.00 mmol, 1.2 equiv) in THF (7 mL) for 12 h. The crude was purified by flash chromatography using pent/EtOAc 40:1 to afford 1-(Tert butyldimethylsilyl)-1H-indole (**2b**) (1.08 g, 4.67 mmol, 93% yield) as white solid.

Rf = 0.42 (pentane).

^{1}H NMR (400 MHz, $CDCl_3$): δ 7.64 (d, *J* = 7.9 Hz, 1H, Ar*H*), 7.52 (d, *J* = 8.2 Hz, 1H, Ar*H*), 7.19 (d, *J* = 3.2 Hz, 1H, Ar*H*), 7.18 – 7.08 (m, 2H, Ar*H*), 6.62 (d, *J* = 3.1 Hz, 1H, Ar*H*), 0.94 (s, 9H, SiC(C*H*$_3$)$_3$), 0.61 (s, 6H, 2 x SiC*H*$_3$).

^{1}H NMR spectrum is in agreement with the reported data.[4]

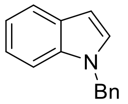

2c

Chemical Formula: $C_{15}H_{13}N$
Molecular Weight: 207.2760

1-Benzylindole (2c): Prepared according to the general procedure B from indole (2.0 g, 17 mmol, 1.0 equiv.), NaH (1.0 g, 26 mmol, 1.5 equiv.) and benzyl bromide (2.5 mL, 20 mmol, 1.2 equiv.) in DMF (34 mL) for 1 h. The crude was purified by flash chromatography using pent/Et_2O 97:3 to afford **2c** as a beige solid (3.2 g, 15 mmol, 89%).

Rf = 0.9 (pent/Et_2O 9:1).

^{1}H NMR (400 MHz, $CDCl_3$): δ 7.68 – 7.63 (m, 1H, Ar*H*), 7.40 – 7.22 (m, 4H, Ar*H*), 7.21 – 7.08 (m, 5H, Ar*H*), 6.56 (d, *J* = 3.1 Hz, 1H, Ar*H*), 5.34 (s, 2H, C*H*$_2$).

^{1}H NMR spectrum is in agreement with the reported data.[5]

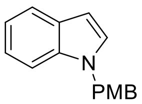

2d

Chemical Formula: $C_{16}H_{15}NO$
Molecular Weight: 237.3020

1-(4-Methoxybenzyl)-indole (2d): Prepared according to the general procedure B from indole (0.13 g, 1.1 mmol, 1.0 equiv.), NaH (52 mg, 1.3 mmol, 1.2 equiv.) and 4-methoxybenzyl bromide (0.19 mL, 1.3 mmol, 1.2 equiv.) in DMF (5.4 mL) for 1 h. The crude was purified by flash chromatography using pent/Et_2O 95:5 to afford **2d** as a colorless oil (195 mg, 822 μmol, 75%).

Rf = 0.9 (pent/Et_2O 9:1).

^{1}H NMR (400 MHz, $CDCl_3$): δ 7.69 – 7.66 (m, 1H, Ar*H*), 7.33 (d, *J* = 8.2 Hz, 1H, Ar*H*), 7.23 – 7.07 (m, 5H, Ar*H*), 6.88 – 6.82 (m, 2H, Ar*H*), 6.58 – 6.55 (m, 1H, Ar*H*), 5.27 (s, 2H, C*H*$_2$), 3.79 (s, 3H, C*H*$_3$).

^{13}C NMR (101 MHz, $CDCl_3$): δ 159.2, 136.3, 129.6, 128.9, 128.3, 128.2, 121.7, 121.1, 119.6, 114.2, 109.8, 101.6, 55.4, 49.7.

HRMS (APCI/QTOF) m/z: $[M + H]^+$ Calcd for $C_{16}H_{16}NO^+$ 238.1226; Found 238.1228.

NMR spectra are in agreement with the reported data.[6]

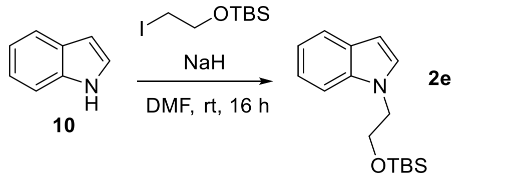

Chemical Formula: $C_{16}H_{25}NOSi$
Molecular Weight: 275.4670

1-(2-((*tert*-Butyldimethylsilyl)oxy)ethyl)-indole (2e): Prepared according to the general procedure B from indole **10** (0.20 g, 1.7 mmol, 1.0 equiv.), NaH (75 mg, 1.9 mmol, 1.1 equiv.) and iodide[7] (0.54 g, 1.9 mmol, 1.1 equiv.) in DMF (5.7 mL) for 16 h. The crude was purified by flash chromatography using pent/Et_2O 97:3 to afford **2e** as a colorless oil (396 mg, 1.44 mmol, 84%).

Rf = 0.79 (pent/Et_2O 9:1).
^{1}H NMR (400 MHz, $CDCl_3$): δ 7.66 – 7.63 (m, 1H, Ar*H*), 7.37 (d, *J* = 8.2 Hz, 1H, Ar*H*), 7.22 (ddd, *J* = 8.2, 7.0, 1.2 Hz, 1H, Ar*H*), 7.16 (d, *J* = 3.0 Hz, 1H, Ar*H*), 7.12 (ddd, *J* = 7.9, 7.0, 1.0 Hz, 1H, Ar*H*), 6.51 (d, *J* = 2.9 Hz, 1H, Ar*H*), 4.25 (t, *J* = 5.7 Hz, 2H, CH_2), 3.94 (t, *J* = 5.7 Hz, 2H, CH_2), 0.86 (s, 9H, TBS), -0.10 (s, 6H, TBS).
^{13}C NMR (101 MHz, $CDCl_3$): δ 136.2, 128.8 (2C), 121.4, 121.0, 119.3, 109.4, 101.1, 62.5, 48.8, 26.0, 18.4, -5.5.
HRMS (ESI/QTOF) m/z: $[M + H]^+$ Calcd for $C_{16}H_{26}NOSi^+$ 276.1778; Found 276.1778.
NMR spectra are in agreement with the reported data.[8]

3-(2-((*tert*-Butyldimethylsilyl)oxy)ethyl)-1-methylindole (2h)

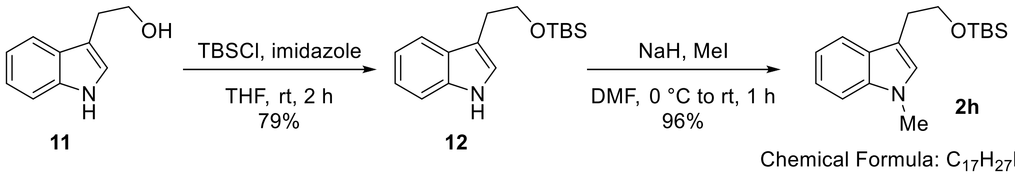

Chemical Formula: $C_{17}H_{27}NOSi$
Molecular Weight: 289.4940

Tryptophol **11** (0.50 g, 3.1 mmol, 1 equiv.) was dissolved in dry THF (10 mL). Imidazole (232 mg, 3.41 mmol, 1.1 equiv.) and *tert*-butylchlorodimethylsilane (514 mg, 3.41 mmol, 1.1 equiv.) were added in one portion at rt. The reaction mixture was stirred at rt for 2 h and was diluted with EtOAc. The organic layer was washed with water and brine, dried over Na_2SO_4, filtered off and concentrated under reduced pressure. The yellow oil was purified by flash chromatography using pent/Et_2O 90:10 to afford **12** as a colorless oil which was used directly in the next step. According to the general procedure B from indole **12** (672 mg, 2.44 mmol, 1.0 equiv.), NaH (107 mg, 2.68 mmol, 1.1 equiv.) and MeI (167 µL, 2.68 mmol, 1.1 equiv.) in DMF (8 mL) for 1 h. The crude was purified by flash chromatography using pent/Et_2O 98:2 to afford **2h** as a colorless oil (675 mg, 2.33 mmol, 75% for two steps).

Rf = 0.79 (pent/Et_2O 9:1).
^{1}H NMR (400 MHz, $CDCl_3$): δ 7.59 (d, *J* = 7.9 Hz, 1H, Ar*H*), 7.28 (d, *J* = 8.3 Hz, 1H, Ar*H*), 7.21 (t, *J* = 7.6 Hz, 1H, Ar*H*), 7.10 (t, *J* = 7.4 Hz, 1H, Ar*H*), 6.89 (s, 1H, Ar*H*), 3.86 (t, *J* = 7.5 Hz, 2H, CH_2), 3.74 (s, 3H, CH_3), 2.98 (t, *J* = 7.5 Hz, 2H, CH_2), 0.91 (s, 9H, TBS), 0.04 (s, 6H, TBS).
^{1}H NMR spectrum is in agreement with the reported data.[9]

3-(2-Bromoethyl)-1-methylindole (14)

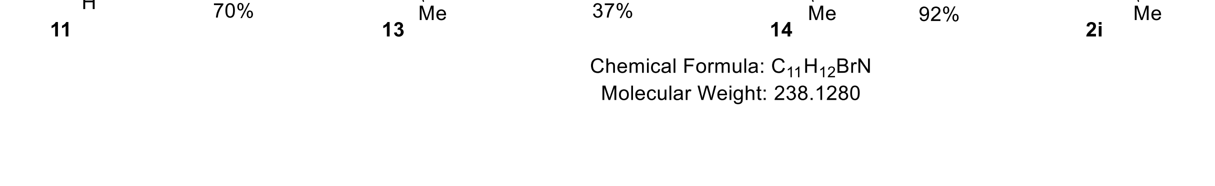

Chemical Formula: $C_{11}H_{12}BrN$
Molecular Weight: 238.1280

Prepared according to the general procedure B from tryptophol **11** (500 mg, 3.10 mmol, 1.0 equiv.), NaH (372 mg, 9.30 mmol, 3.0 equiv.) and MeI (193 µL, 3.10 mmol, 1.0 equiv.) in DMF (10 mL) for 1 h. The crude was purified by flash chromatography using pent/EtOAc 80:20 to afford alcohol **13** as a colorless oil (381 mg, 2.17 mmol, 70%). Then, in a 10 mL RBF, alcohol **13** (316 mg, 1.80 mmol, 1.0 equiv.), triphenylphosphine (473 mg, 1.80 mmol, 1.0 equiv.) and imidazole (123 mg, 1.80 mmol, 1.0 equiv.) were diluted in 18 mL of dry DCM. Br_2 (93 µL, 1.8 mmol, 1.0 equiv.) was added dropwise at 0 °C. The reaction mixture was allowed to reach room temperature and was stirred o/n. Then, a saturated aqueous solution of $Na_2S_2O_3$ was added. The aqueous layer was extracted with DCM. The combined organic layers were washed with a saturated aqueous solution of $Na_2S_2O_3$ and brine, dried over $MgSO_4$ and concentrated under reduced pressure. The crude was purified by flash chromatography using pent/Et_2O 98:2 to 97:3 to afford **14** as a colorless oil (159 mg, 667 µmol, 37%).

Rf = 0.8 (pentane/EtOAc 9:1).
^{1}H NMR (400 MHz, $CDCl_3$): δ 7.58 (d, *J* = 7.9 Hz, 1H, Ar*H*), 7.31 (d, *J* = 8.2 Hz, 1H, Ar*H*), 7.25 – 7.21 (m, 1H, Ar*H*), 7.16 – 7.10 (m, 1H, Ar*H*), 6.95 (s, 1H, Ar*H*), 3.77 (s, 3H, C*H*3), 3.62 (t, *J* = 7.7 Hz, 2H, C*H*2), 3.33 (t, *J* = 7.7 Hz, 2H, C*H*2).
^{13}C NMR (101 MHz, $CDCl_3$): δ 137.1, 127.5, 127.1, 121.9, 119.2, 118.7, 112.0, 109.5, 33.2, 32.8, 29.4.
HRMS (ESI/QTOF) m/z: $[M + H]^+$ Calcd for $C_{11}H_{13}{}^{79}BrN^+$ 238.0226; Found 238.0228.

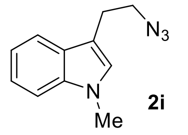
2i
Chemical Formula: $C_{11}H_{12}N_4$
Molecular Weight: 200.2450

3-(2-Azidoethyl)-1-methylindole (2i): To a solution of bromide **14** (127 mg, 533 µmol, 1 equiv.) in a mixture of acetone/water 4:1 (5 mL) was added sodium azide (104 mg, 1.60 mmol, 3.0 equiv.) and the mixture was stirred for two days at rt. DCM was added to the reaction mixture and the organic layer was separated. The aqueous layer was extracted with DCM and the combined organic layers were washed with brine, dried over Na_2SO_4 and concentrated under reduced pressure. The crude azide **2i** (98 mg, 0.49 mmol, 92%) was pure enough and did not require any purification.

Rf = 0.6 (pent/Et20 96:4).
^{1}H NMR (400 MHz, $CDCl_3$) δ 7.58 (dt, *J* = 7.9, 1.0 Hz, 1H, Ar*H*), 7.31 (d, *J* = 8.2 Hz, 1H, Ar*H*), 7.27 – 7.20 (m, 1H, Ar*H*), 7.17 – 7.08 (m, 1H, Ar*H*), 6.93 (s, 1H, Ar*H*), 3.77 (s, 3H, C*H*3), 3.56 (t, *J* = 7.3 Hz, 2H, C*H*2), 3.06 (t, *J* = 7.3 Hz, 2H, C*H*2).
^{1}H NMR spectrum is in agreement with the reported data.[10]

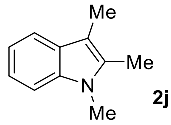
2j
Chemical Formula: $C_{11}H_{13}N$
Molecular Weight: 159.2320

1,2,3-Trimethylindole (2j): Prepared according to the general procedure B from 2,3-dimethylindole (420 mg, 2.89 mmol, 1.0 equiv.), NaH (139 mg, 3.47 mmol, 1.2 equiv.) and MeI (189 µL, 3.04 mmol, 1.05 equiv.) in DMF (7 mL) for 1 h. The crude was purified by flash chromatography using pent/Et_2O 97:3 to afford **2j** as a colorless oil (415 mg, 2.6 mmol, 90%).

Rf = 0.87 (pent/Et_2O 9:1).
^{1}H NMR (400 MHz, $CDCl_3$): δ 7.49 (d, *J* = 7.7 Hz, 1H, Ar*H*), 7.25 – 7.22 (m, 1H, Ar*H*), 7.17 – 7.12 (m, 1H, Ar*H*), 7.10 – 7.05 (m, 1H, Ar*H*), 3.65 (s, 3H), 2.35 (s, 3H, C*H*3), 2.26 (s, 3H, C*H*3).
^{1}H NMR spectrum is in agreement with the reported data.[11]

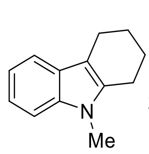

2k

Chemical Formula: $C_{13}H_{15}N$
Molecular Weight: 185.2700

9-Methyl-2,3,4,9-tetrahydrocarbazole (2k): Prepared according to the general procedure B from 1,2,3,4-tetrahydrocarbazole (300 mg, 1.75 mmol, 1.0 equiv.), NaH (84 mg, 2.10 mmol, 1.2 equiv.) and MeI (115 µL, 1.84 mmol, 1.05 equiv.) in DMF (6 mL) for 1 h. The crude was purified by flash chromatography using pent/Et_2O 98:2 to afford **2k** as a colorless oil (317 mg, 1.71 mmol, 95%).

Rf = 0.83 (pent/Et_2O 9:1).
^{1}H NMR (400 MHz, $CDCl_3$): δ 7.47 (d, *J* = 7.7 Hz, 1H, Ar*H*), 7.24 (s, 1H, Ar*H*), 7.18 – 7.12 (m, 1H, Ar*H*), 7.09 – 7.04 (m, 1H, Ar*H*), 3.62 (s, 3H, C*H*$_3$), 2.76 – 2.67 (m, 4H, 2xC*H*$_2$), 1.99 – 1.91 (m, 2H, C*H*$_2$), 1.90 – 1.82 (m, 2H, C*H*$_2$).
^{1}H NMR spectrum is in agreement with the reported data.[12]

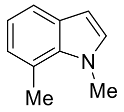

2l

Chemical Formula: $C_{10}H_{11}N$
Molecular Weight: 145.2050

1,7-Dimethylindole (2l): Prepared according to the general procedure B from 7-methylindole (400 mg, 3.05 mmol, 1.0 equiv.), NaH (146 mg, 3.66 mmol, 1.2 equiv.) and MeI (199 µL, 3.20 mmol, 1.05 equiv.) in DMF (8 mL) for 1 h. The crude was purified by flash chromatography using pent/Et_2O 95:5 to afford **2l** as a colorless oil (440 mg, 3.03 mmol, quant.).

Rf = 0.79 (pent/Et_2O 9:1).
^{1}H NMR (400 MHz, $CDCl_3$): δ 7.50 – 7.43 (m, 1H, Ar*H*), 7.02 – 6.89 (m, 3H, Ar*H*), 6.47 – 6.41 (m, 1H, Ar*H*), 4.07 (s, 3H, C*H*$_3$), 2.78 (s, 3H, C*H*$_3$).
^{1}H NMR spectrum is in agreement with the reported data.[13]

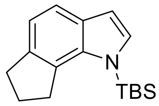

2m

Chemical Formula: $C_{17}H_{25}NSi$
Molecular Weight: 271.4790

1-(*Tert*-butyldimethylsilyl)-1,6,7,8-tetrahydrocyclopenta[g]indole (**2m**): Prepared according to the general procedure B from 1,6,7,8-tetrahydrocyclopenta[g]indole (0.47 g, 3.0 mmol, 1.0 equiv), NaH (0.36 g, 9.0 mmol, 3.0 equiv) and *tert*-butylchlorodimethylsilane (0.68 g, 4.5 mmol, 1.5 equiv) in THF (9 mL) for 12 h. The crude was purified by flash chromatography using pent/EtOAc 20:1 to afford **2m** (0.74 g, 2.7 mmol, 91%) as white solid.

Rf = 0.40 (pentane).
m. p. = 53.8 – 54.8 °C;
^{1}H NMR (400 MHz, $CDCl_3$) δ 7.45 (d, *J* = 7.8 Hz, 1H, Ar*H*), 7.22 (d, *J* = 3.2 Hz, 1H, Ar*H*), 7.08 (d, *J* = 7.8 Hz, 1H, Ar*H*), 6.61 (d, *J* = 3.2 Hz, 1H, Ar*H*), 3.18 (t, *J* = 7.2 Hz, 2H, CH_2CH_2C*H*$_2$), 3.01 (t, *J* = 7.4 Hz, 2H, C*H*$_2$$CH_2CH_2$), 2.12 (p, *J* = 7.3 Hz, 2H, CH_2C*H*$_2$$CH_2$), 0.91 (s, 9H, SiC(C*H*$_3$)$_3$), 0.62 (s, 6H, 2 x SiC*H*$_3$).
^{13}C NMR (101 MHz, CDCl3) δ 139.1, 138.6, 131.8, 131.2, 126.8, 118.7, 117.3, 105.2, 34.5, 33.3, 26.6, 26.0, 19.6, -1.0.
IR (film) $\tilde{\nu}$ 2952 (m), 2934 (m), 2891 (w), 2860 (m), 1528 (w), 1466 (m), 1411 (m), 1294 (w), 1263 (m), 1214 (w), 1133 (m), 1084 (m), 1022 (w), 838 (m), 807 (s), 720 (m).
HRMS (ESI) m/z: $[M+H]^+$ Calcd for $C_{17}H_{26}NSi^+$ 272.1829; found 272.1828.

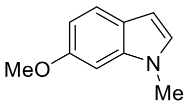

2n

Chemical Formula: $C_{10}H_{11}NO$
Molecular Weight: 161.2040

6-Methoxy-1-methylindole (2n): Prepared according to the general procedure B from 6-methoxyindole (400 mg, 2.72 mmol, 1.0 equiv.), NaH (130 mg, 3.26 mmol, 1.2 equiv.) and MeI (178 µL, 2.85 mmol, 1.05 equiv.) in DMF (7 mL) for 1 h. The crude was purified by flash chromatography using pent/Et_2O 95:5 to afford **2n** as a colorless oil (356 mg, 2.21 mmol, 81%).

Rf = 0.45 (pent/Et_2O 9:1).

^{1}H NMR (400 MHz, $CDCl_3$): δ 7.51 – 7.47 (m, 1H, Ar*H*), 6.95 (s, 1H, Ar*H*), 6.81 – 6.77 (m, 2H, Ar*H*), 6.41 (brs, 1H, Ar*H*), 3.89 (s, 3H, C*H*$_3$), 3.74 (s, 3H, C*H*$_3$).
^{1}H NMR spectrum is in agreement with the reported data.[14]

2-(1-Methyl-1H-indol-5-yl)isoindoline-1,3-dione (2o): 5-aminoindole **15** (200 mg, 1.51 mmol, 1 equiv.) and phthalimic anhydride (247 mg, 1.66 mmol, 1.1 equiv.) were heated under reflux in 5 mL of dry toluene with a dean-stark apparatus for 2 h. The mixture was then cooled to rt and the beige solid was filtered off and rinsed 3 times with toluene. The crude solid was directly used in the next step.

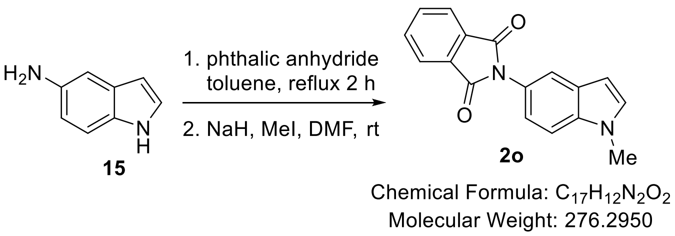

According to the general procedure A from previously prepared indole (252 mg, 961 µmol, 1.0 equiv.) NaH (57 mg, 1.4 mmol, 1.5 equiv.), MeI (90 µL, 1.4 mmol, 1.5 equiv.) in DMF (3 mL) for 1h. The crude product was purified by flash chromatography (pent/EtOAc 85:15) to afford **2o** as white solid (162 mg, 586 µmol, 39%, two steps).

Rf = 0.69 (DCM).
m. p. = 214 – 214 °C.
^{1}H NMR (400 MHz, DMSO-*d*$_6$): δ 7.98 – 7.93 (m, 2H, Ar*H*), 7.92 – 7.87 (m, 2H, Ar*H*), 7.58 (d, *J* = 1.7 Hz, 1H, Ar*H*), 7.54 (d, *J* = 8.7 Hz, 1H, Ar*H*), 7.43 (d, *J* = 3.0 Hz, 1H, Ar*H*), 7.17 (dd, *J* = 8.7, 1.9 Hz, 1H, Ar*H*), 6.51 (dd, *J* = 3.1, 0.8 Hz, 1H, Ar*H*), 3.84 (s, 3H, C*H*$_3$).
^{13}C NMR (101 MHz, DMSO-*d*$_6$): δ 167.7, 135.7, 134.5, 131.7, 130.9, 127.8, 123.3, 123.3, 120.7, 119.8, 109.8, 100.7, 32.7.
HRMS (ESI/QTOF) m/z: $[M + H]^+$ Calcd for $C_{17}H_{13}N_2O_2^+$ 277.0972; Found 277.0971.

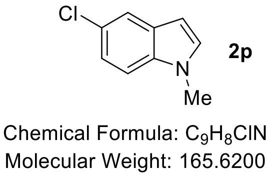

2p

5-Chloro-1-methylindole (2p): Prepared according to the general procedure B from 5-chloroindole (445 mg, 2.94 mmol, 1.0 equiv.), NaH (141 mg, 3.52 mmol, 1.2 equiv.) and MeI (192 µL, 3.08 mmol, 1.05 equiv.) in DMF (7 mL) for 1 h. The crude was purified by flash chromatography using pent/Et_2O 95:5 to afford **2p** as a colorless oil (432 mg, 2.61 mmol, 89%).

Rf = 0.67 (pent/Et_2O 9:1).
^{1}H NMR (400 MHz, $CDCl_3$): δ 7.58 (d, *J* = 2.0 Hz, 1H, Ar*H*), 7.23 (d, *J* = 8.7 Hz, 1H, Ar*H*), 7.17 (dd, *J* = 8.7, 2.0 Hz, 1H, Ar*H*), 7.07 (d, *J* = 3.0 Hz, 1H, Ar*H*), 6.42 (d, *J* = 3.0 Hz, 1H, Ar*H*), 3.78 (s, 3H, C*H*$_3$).
^{1}H NMR spectrum is in agreement with the reported data.[15]

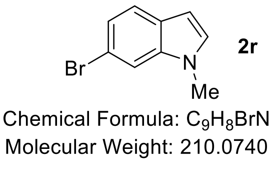

2r

6-Bromo-1-methylindole (2r): Prepared according to the general procedure B from 6-bromo-1H-indole (266 mg, 1.36 mmol, 1.0 equiv.), NaH (65 mg, 1.6 mmol, 1.2 equiv.) and MeI (88 µL, 1.42 mmol, 1.05 equiv.) in DMF (3 mL) for 1 h. The crude was purified by flash chromatography using pent/Et_2O 95:5 to afford **2r** as a colorless oil (246 mg, 1.17 mmol, 84%).

Rf = 0.71 (pent/Et_2O 9:1).
^{1}H NMR (400 MHz, $CDCl_3$): δ 7.53 – 7.46 (m, 2H, Ar*H*), 7.26 – 7.19 (m, 1H, Ar*H*), 7.03 (d, *J* = 3.1 Hz, 1H, Ar*H*), 6.47 (d, *J* = 3.0 Hz, 1H, Ar*H*), 3.75 (s, 3H, C*H*$_3$).
^{13}C NMR (101 MHz, $CDCl_3$): δ 137.6, 129.6, 127.4, 122.6, 122.2, 115.2, 112.4, 101.3, 33.0.
NMR spectra are in agreement with the reported data.[16]

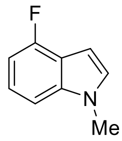

2s

Chemical Formula: C_9H_8FN
Molecular Weight: 149.1684

4-Fluoro-1-methylindole (2s): Prepared according to the general procedure B from 4-fluoroindole (303 mg, 2.24 mmol, 1.0 equiv.), NaH (108 mg, 2.69 mmol, 1.2 equiv.) and MeI (147 µL, 2.35 mmol, 1.05 equiv.) in DMF (5.6 mL) for 1 h. The crude was purified by flash chromatography using pent/Et_2O 97:3 to afford **2s** as a colorless oil (291 mg, 1.95 mmol, 87%).

Rf = 0.79 (pent/Et_2O 9:1).
^{1}H NMR (400 MHz, $CDCl_3$): δ 7.21 – 7.11 (m, 2H, Ar*H*), 7.04 (d, *J* = 3.1 Hz, 1H, Ar*H*), 6.83 (ddd, *J* = 10.4, 7.5, 1.1 Hz, 1H, Ar*H*), 6.62 (dd, *J* = 3.1, 0.8 Hz, 1H, Ar*H*), 3.80 (s, 3H, C*H*$_3$).
^{13}C NMR (101 MHz, $CDCl_3$): δ 156.5 (d, *J* = 246.8 Hz), 139.5 (d, *J* = 11.7 Hz), 128.8, 122.1 (d, *J* = 7.8 Hz), 117.5 (d, *J* = 22.6 Hz), 105.5 (d, *J* = 3.6 Hz), 104.2 (d, *J* = 19.1 Hz), 97.1, 33.2.
^{19}F NMR (376 MHz, $CDCl_3$) δ -122.2 – -122.3 (m)
IR (ν_{max}, cm^{-1}) 3032 (w), 1501 (m), 1289 (m), 1229 (s), 975 (m), 734 (s).
HRMS (ESI/QTOF) m/z: $[M + H]^+$ Calcd for $C_9H_9FN^+$ 150.0714; Found 150.0711.

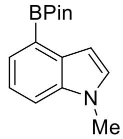

2t

Chemical Formula: $C_{15}H_{20}BNO_2$
Molecular Weight: 257,14

1-Methyl-4-(4,4,5,5-tetramethyl-1,3,2-dioxaborolan-2-yl)-indole (2t): Prepared according to the general procedure B from 4-(4,4,5,5-tetramethyl-1,3,2-dioxaborolan-2-yl)-1H-indole (299 mg, 1.23 mmol, 1 equiv.), NaH (74 mg, 1.8 mmol, 1.5 equiv.) and MeI (80 µL, 1.3 mmol, 1.05 equiv.) in THF (6 mL) for 1 h. The crude was purified by flash chromatography using pent/Et_2O 95:5 to afford **2t** as a colorless oil (226 mg, 879 µmol, 73%).

Rf = 0.64 (pentane/Et_2O 9:1).
^{1}H NMR (400 MHz, $CDCl_3$): δ 7.65 (d, *J* = 7.0 Hz, 1H, Ar*H*), 7.44 (d, *J* = 8.2 Hz, 1H, Ar*H*), 7.25 (dd, *J* = 8.2, 7.0 Hz, 1H, Ar*H*), 7.11 (d, *J* = 2.5 Hz, 1H, Ar*H*), 6.99 (d, *J* = 3.0 Hz, 1H, Ar*H*), 3.81 (s, 3H, C*H*$_3$), 1.41 (s, 12H, 4xC*H*$_3$).
^{13}C NMR (101 MHz, $CDCl_3$): δ 136.2, 133.3, 129.4, 127.5 (2C), 120.9, 112.2, 103.0, 83.5, 32.9, 25.1.
HRMS (ESI/QTOF) m/z: $[M + H]^+$ Calcd for $C_{15}H_{21}BNO_2^+$ 258.1660; Found 258.1668.

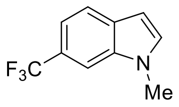

2u

Chemical Formula: $C_{10}H_8F_3N$
Molecular Weight: 199.1762

1-Methyl-6-(trifluoromethyl)indole (2u): Prepared according to the general procedure B from 6-(trifluoromethyl)indole (305 mg, 1.65 mmol, 1.0 equiv.), NaH (79 mg, 1.97 mmol, 1.2 equiv.) and MeI (108 µL, 1.73 mmol, 1.05 equiv.) in DMF (4 mL) for 1 h. The crude was purified by flash chromatography using pent/Et_2O 97:3 to afford **2u** as a colorless oil (301 mg, 1.51 mmol, 94%).

Rf = 0.79 (pent/Et_2O 9:1).
^{1}H NMR (400 MHz, $CDCl_3$): δ 7.73 (d, *J* = 8.3 Hz, 1H, Ar*H*), 7.65 – 7.63 (m, 1H, Ar*H*), 7.39 (dd, *J* = 8.3, 1.6 Hz, 1H, Ar*H*), 7.20 (d, *J* = 3.1 Hz, 1H, Ar*H*), 6.58 (dd, *J* = 3.1, 0.9 Hz, 1H, Ar*H*), 3.83 (s, 3H, C*H*$_3$).
^{13}C NMR (101 MHz, $CDCl_3$): δ 135.7, 131.6, 130.9, 125.5 (q, *J* = 271.4 Hz), 123.7 (q, *J* = 31.9 Hz), 121.3, 116.0 (q, *J* = 3.5 Hz), 106.9 (q, *J* = 4.5 Hz), 101.4, 33.0.
^{19}F NMR (376 MHz, $CDCl_3$) δ -60.4.
IR (ν_{max}, cm^{-1}) 3037 (w), 1351 (s), 1299 (s), 1160 (s), 1114 (s).
HRMS (ESI/QTOF) m/z: $[M + H]^+$ Calcd for $C_{10}H_9F_3N^+$ 200.0682; Found 200.0675.

2.1. Synthesis of D-A aminocyclopropanes

2.1.1. Synthesis of vinyl sulfonamides

The vinyl sulfonamides were prepared by a two-step procedure as shown with *N*-vinyl-*N*-methyl-*p*-toluenesulfonamide **18** in Scheme S3. This procedure was applied to other compounds.

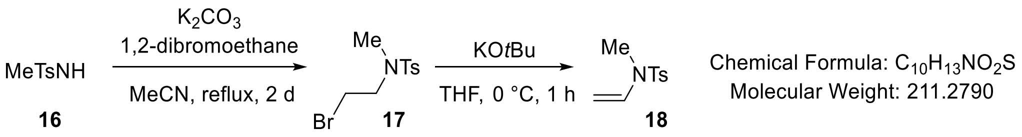

Scheme S3. Synthesis of *N*-vinyl-*N*-methyl-*p*-toluenesulfonamide **18**.

General procedure C for the gram scale synthesis of vinyl sulfonamides

A mixture of *N*-methyl-*p*-toluene sulphonamide **16** (8.46 g, 45.7 mmol, 1.0 equiv.), 1,2-dibromoethane (39.5 mL, 457 mmol, 10.0 equiv.) and K_2CO_3 (18.9 g, 137 mmol, 3.0 equiv.) in CH_3CN was refluxed for 2 or 3 days. The mixture was then filtered off and concentrated under reduced pressure. The crude product was purified by flash chromatography (pentane/EtOAc 85:15) to afford **17** as a yellow oil (11.9 g, 89%).

Then, to a stirred solution of *N*-(2-bromoethyl)-*N*-methyl-p-toluenesulfonamide **17** (11.9 g, 40.7 mmol, 1.0 equiv.) in THF (80 mL) was added *t*BuOK (6.9 g, 61 mmol, 1.5 equiv.) at 0 °C. The resulting pale-yellow solution was allowed to stir for 1 h at 0 °C before H_2O was added at 0 °C. The aqueous layer was extracted with Et_2O and the combined organic layers were washed with brine, dried over Na_2SO_4, filtered off and evaporated to dryness. The crude vinyl sulfonamide **18** was pure enough without further purification and was obtained as white crystal (7.3 g, 34 mmol, 83%).

Rf = 0.75 (pent/EtOAc 4:1).
^{1}H NMR (400 MHz, $CDCl_3$): δ 7.64 (d, *J* = 8.4 Hz, 2H, Ts), 7.30 (d, *J* = 8.0 Hz, 2H, Ts), 7.00 (dd, *J* = 15.6, 9.0 Hz, 1H, C*H*), 4.33 (dd, *J* = 9.0, 1.4 Hz, 1H, CH_2), 4.18 (dd, *J* = 15.6, 1.4 Hz, 1H, CH_2), 2.86 (s, 3H, CH_3), 2.42 (s, 3H, CH_3).
^{13}C NMR (101 MHz, $CDCl_3$): δ 143.9, 134.7, 133.7, 129.8, 126.9, 93.3, 31.3, 21.5.
HRMS (ESI/QTOF) m/z: $[M + H]^+$ Calcd for $C_{10}H_{14}NO_2S^+$ 212.0740; Found 212.0742.
NMR spectra are in agreement with the reported data.[17]

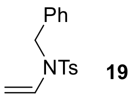

Chemical Formula: $C_{16}H_{17}NO_2S$
Molecular Weight: 287.3770

***N*-Benzyl-4-methyl-*N*-vinylbenzenesulfonamide (19)**: Prepared according to the general procedure C from *N*-benzyl-4-methylbenzenesulfonamide (2.5 g, 9.6 mmol, 1.0 equiv.) using 1,2-dibromoethane (8.3 mL, 96 mmol, 10.0 equiv.), K_2CO_3 (4.0 g, 28.7 mmol, 3.0 equiv.) in MeCN (10 mL) for 2 days. After purification of the bromide by flash chromatography using pent/EtOAc 85:15, the elimination was performed using KO*t*Bu (1.6 g, 14 mmol, 1.5 equiv.) in THF (32 mL) for 1 h. The vinyl sulfonamide **19** was obtained as white crystal without further purification (2.3 g, 8.0 mmol, 84%, two steps).

Rf = 0.86 (pent/EtOAc 9:1).
m. p. = 103.3 – 104.4 °C.
^{1}H NMR (400 MHz, $CDCl_3$): δ 7.69 – 7.65 (m, 2H, Ts), 7.31 – 7.18 (m, 7H, Ts, Ar*H*), 6.95 (dd, *J* = 15.7, 9.2 Hz, 1H, C*H*), 4.51 (s, 2H, CH_2), 4.24 (dd, *J* = 9.2, 1.5 Hz, 1H, CH_2), 4.12 (dd, *J* = 15.7, 1.5 Hz, 1H, CH_2), 2.40 (s, 3H, CH_3).
^{13}C NMR (101 MHz, $CDCl_3$): δ 144.0, 136.2, 135.6, 132.2, 130.0, 128.6, 127.5, 127.0, 126.9, 94.7, 48.9, 21.7.

IR (ν_{max}, cm^{-1}) 3060 (m), 1625 (s), 1354 (s), 1319 (s), 1163 (s).
HRMS (ESI/QTOF) m/z: $[M + H]^+$ Calcd for $C_{16}H_{18}NO_2S^+$ 288.1053; Found 288.1056.

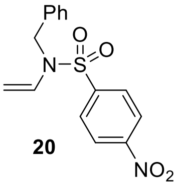

20
Chemical Formula: $C_{15}H_{14}N_2O_4S$
Molecular Weight: 318.3470

***N*-Benzyl-4-nitro-*N*-vinylbenzenesulfonamide (20)**: Prepared according to a slightly modified general procedure C from *N*-benzyl-4-nitrobenzenesulfonamide[18] (432 mg, 1.48 mmol, 1.0 equiv.), 1,2-dibromoethane (1.30 mL, 14.8 mmol, 10.0 equiv.), K_2CO_3 (613 mg, 4.43 mmol, 3.0 equiv.) in MeCN (2 mL). The crude bromide was not purified and was used directly in the elimination using *t*BuOK (332 mg, 2.96 mmol, 2.0 equiv.) in THF (10 mL) at -78 °C for 2 h. The crude was purified by flash chromatography using pent/EtOAc 90:10 to afford vinyl sulfonamide **20** as white solid (271 mg, 567 µmol, 57%).
Rf = 0.48 (pentane/EtOAc 9:1).
m. p. = 116 – 117.5 °C.
^{1}H NMR (400 MHz, $CDCl_3$): δ 8.36 – 8.30 (m, 2H, Ns), 7.97 – 7.91 (m, 2H, Ns), 7.32 – 7.21 (m, 5H, Ar*H*), 6.94 (dd, *J* = 15.7, 9.2 Hz, 1H, C*H*), 4.61 (s, 2H, CH_2), 4.42 (dd, *J* = 9.2, 1.8 Hz, 1H, CH_2), 4.32 (dd, *J* = 15.7, 1.8 Hz, 1H, CH_2).
^{13}C NMR (101 MHz, $CDCl_3$): δ 150.3, 144.7, 134.7, 131.5, 128.8, 128.3, 127.9, 127.1, 124.6, 96.8, 49.1.
IR (ν_{max}, cm^{-1}) 3107 (m), 2963 (m), 1531 (s), 1349 (s), 1165 (s), 741 (s).
HRMS (ESI/QTOF) m/z: $[M + Na]^+$ Calcd for $C_{15}H_{14}N_2NaO_4S^+$ 341.0566; Found 341.0565.

Internal vinyl sulfonamide **24** was synthesized from a literature procedure through a metathesis reaction (Scheme S4).[19]

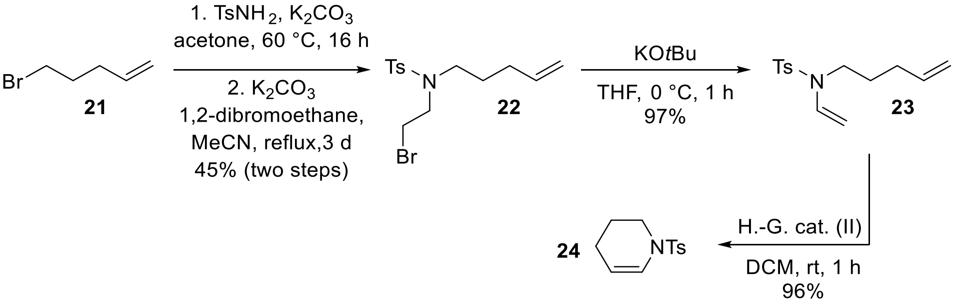

Scheme S4. Synthesis of an internal vinyl sulfonamide.

***N*-(2-Bromoethyl)-4-methyl-*N*-(pent-4-en-1-yl)benzenesulfonamide (22)**: To an oven-dried RBF was added 5-bromo-1-pentene **21** (3.0 mL, 25 mmol, 1.1 eq.), *p*-toluenesulfonamide (3.9 g, 23 mmoles, 1.0 eq.), K_2CO_3 (6.4 g, 46 mmol, 2.0 eq.) and acetone (23 mL). The round bottom flask was heated to 60 °C. Upon completion of the reaction (16 h), the solution was cooled to rt, filtered through a plug of Celite and rinsed with EtOAc, and concentrated under reduced pressure to afford the crude product. Purification of the resulting crude residue by flash column chromatography (pent/EtOAc 80:20) afforded the desired sulfonamide product (3.5 g, 15 mmol, 64%) which was directly used for the next step. According to the general procedure C from sulfonamide (3.5 g, 15 mmol, 1 equiv.), 1,2-dibromoethane (12.8 mL, 147 mmol, 10.0 equiv.) and K_2CO_3 (6.1 g, 44 mmol, 3.0 equiv.) in CH_3CN (15 mL) for 3 days. The crude product was purified by flash chromatography using pentane/EtOAc 90:10 to afford **22** as a yellow oil (3.6 g, 10 mmol, 71%)

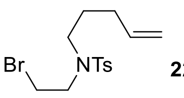

22
Chemical Formula: $C_{14}H_{20}BrNO_2S$
Molecular Weight: 346.2830

Rf = 0.76 (pentane/EtOAc 9:1).
^{1}H NMR (400 MHz, $CDCl_3$): δ 7.71 – 7.65 (m, 2H, Ts), 7.34 – 7.27 (m, 2H, Ts), 5.82 – 5.67 (m, 1H, C*H*), 5.06 – 4.94 (m, 2H, CH_2), 3.50 – 3.37 (m, 4H, CH_2CH_2Br), 3.17 – 3.09 (m, 2H, CH_2), 2.42 (s, 3H, CH_3), 2.08 – 2.01 (m, 2H, CH_2), 1.69 – 1.57 (m, 2H, CH_2).
^{13}C NMR (101 MHz, $CDCl_3$): δ 143.7, 137.2, 136.2, 129.9, 127.2, 115.6, 50.3, 49.3, 30.7, 29.6, 28.2, 21.6.
IR (ν_{max}, cm^{-1}) 3071 (m), 2935 (w), 1598 (m), 1446 (m), 1338 (s), 1156 (s), 915 (s), 730 (s).
HRMS (ESI/QTOF) m/z: $[M + H]^+$ Calcd for $C_{14}H_{21}{}^{79}BrNO_2S^+$ 346.0471; Found 346.0471.

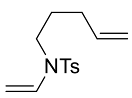
23
Chemical Formula: $C_{14}H_{19}NO_2S$
Molecular Weight: 265.3710

4-Methyl-*N*-(pent-4-en-1-yl)-*N*-vinylbenzenesulfonamide (23): Prepared according to the general procedure C from bromide **22** (3.4 g, 9.9 mmol, 1.0 equiv.) and *t*BuOK (1.5 mL, 12 mmol, 1.2 equiv.) in THF (50 mL) for 1 h. The crude vinyl sulfonamide **23** was pure enough without further purification and was obtained as a colorless oil (2.57 g, 9.68 mmol, 97%).

Rf = 0.86 (pentane/EtOAc 9:1).
^{1}H NMR (400 MHz, $CDCl_3$): δ 7.64 (d, *J* = 8.3 Hz, 2H, Ts), 7.32 – 7.25 (m, 2H, Ts), 6.87 (dd, *J* = 15.8, 9.3 Hz, 1H, C*H*), 5.84 – 5.70 (m, 1H, C*H*), 5.06 – 4.94 (m, 2H, CH_2), 4.32 (dd, *J* = 9.3, 1.4 Hz, 1H, CH_2), 4.23 (dd, *J* = 15.8, 1.3 Hz, 1H, CH_2), 3.34 – 3.25 (m, 2H, NCH_2), 2.40 (s, 3H, CH_3), 2.11 – 2.02 (m, 2H, CH_2), 1.73 – 1.63 (m, 2H, CH_2).
^{13}C NMR (101 MHz, $CDCl_3$): δ 143.8, 137.4, 136.4, 132.2, 129.9, 126.9, 115.5, 92.9, 44.4, 30.9, 26.0, 21.6.
IR (ν_{max}, cm^{-1}) 3073 (w), 2937 (w), 1626 (m), 1352 (s), 1161 (s), 972 (s), 658 (s).
HRMS (ESI/QTOF) m/z: $[M + H]^+$ Calcd for $C_{14}H_{20}NO_2S^+$ 266.1209; Found 266.1208.

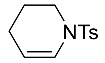
24
Chemical Formula: $C_{12}H_{15}NO_2S$
Molecular Weight: 237.3170

1-Tosyl-1,2,3,4-tetrahydropyridine (24): The H.-G. catalyst (second generation, 59 mg, 95 µmol, 2.5 mol%) was added to a solution of vinyl sulfonamide **23** (1.0 g, 3.8 mmol, 1.0 equiv.) diluted in 38 mL of degassed dry DCM (15 min by nitrogen sparging). The reaction was stirred at rt for 16 h and the solvent was evaporated. The crude was purified by flash chromatography using pent/Et_2O 90:10 to afford **24** as a colorless oil (872 mg, 3.67 mmol 96%).

Rf = 0.35 (pentane/EtOAc 9:1).
m. p. = 59.1 – 60.9 °C.
^{1}H NMR (400 MHz, $CDCl_3$): δ 7.68 – 7.64 (m, 2H, Ts), 7.32 – 7.28 (m, 2H, Ts), 6.63 (dt, *J* = 8.4, 2.0 Hz, 1H, C*H*), 4.96 (dt, *J* = 8.4, 3.9 Hz, 1H, C*H*), 3.38 – 3.34 (m, 2H, NCH_2), 2.42 (s, 3H, CH_3), 1.94 – 1.86 (m, 2H, CH_2), 1.69 – 1.61 (m, 2H, CH_2).
^{13}C NMR (101 MHz, $CDCl_3$): δ 143.7, 135.2, 129.8, 127.2, 125.2, 108.4, 44.0, 21.7, 21.1, 21.0.
IR (ν_{max}, cm^{-1}) 3019 (w), 2935 (m), 1650 (m), 1344 (s), 1165 (s), 1105 (s), 965 (s), 929 (s).
HRMS (ESI/QTOF) m/z: $[M + H]^+$ Calcd for $C_{12}H_{16}NO_2S^+$ 238.0896; Found 238.0899.

N,4-Dimethyl-*N*-(prop-1-en-2-yl)benzenesulfonamide (26)

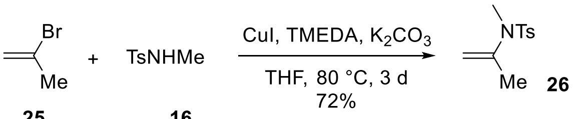

25 **16**
Chemical Formula: $C_{11}H_{15}NO_2S$
Molecular Weight: 225.3060

According to a reported procedure initially described with vinyl bromide,[20] to a microwave vial fitted with a magnetic stir bar was added *N*-methyl-*p*-toluenesulfonamide **16** (2.1 g, 11 mmol, 2.0 equiv.),

2-bromopropene **25** (500 µL, 5.63 mmol, 1.0 equiv.), copper(I) iodide (53 mg, 0.28 mmol, 5 mol%.), *N*,*N*'-dimethylethylenediamine (60 µL, 0.56 mmol, 0.1 equiv.), K_2CO_3 (1.55 g, 11.2 mmol, 2.0 equiv.) and THF (5.6 mL). The vial was tightly sealed and heated to 80 °C for 3 days while maintaining a vigorous stirring. Then, the mixture was filtered off through a pad of Celite, rinsing with EtOAc, and the filtrate was concentrated under reduced pressure. The crude product was purified by flash chromatography using pent/EtOAc 90:10 to afford **26** as a colorless oil (918 mg, 4.07 mmol, 72%).

Rf = 0.9 (pentane/EtOAc 4:1).
^{1}H NMR (400 MHz, CD_3CN): δ 7.67 – 7.63 (m, 2H, Ts), 7.40 – 7.36 (m, 2H, Ts), 4.81 – 4.79 (m, 1H, C*H*$_2$), 4.51 (s, 1H, C*H*$_2$), 2.92 (s, 3H, C*H*$_3$), 2.41 (s, 3H, C*H*$_3$), 1.91 – 1.87 (m, 3H, C*H*$_3$).
^{13}C NMR (101 MHz, CD_3CN): δ 146.0, 144.9, 135.4, 130.5, 128.5, 109.9, 37.8, 22.3, 21.5.
IR (ν_{max}, cm^{-1}) 3035 (w), 2926 (w), 1344 (s), 1158 (s), 876 (s), 684 (s).
HRMS (ESI/QTOF) m/z: [M + Na]$^+$ Calcd for $C_{11}H_{15}NNaO_2S^+$ 248.0716; Found 248.0723.

4-Methyl-*N*-(2-(1-methylindol-3-yl)ethyl)-*N*-vinylbenzenesulfonamide (29)

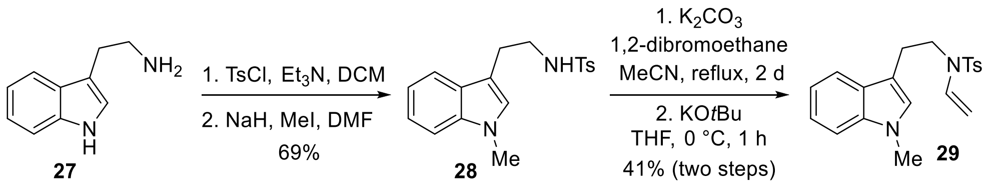

Chemical Formula: $C_{20}H_{22}N_2O_2S$
Molecular Weight: 354.4680

The indole-containing vinyl sulfonamide **28** was prepared from a reported procedure.[21] According to the general procedure C from sulfonamide **28** (2.1 g, 6.4 mmol, 1.0 equiv.), 1,2-dibromoethane (5.5 mL, 64 mmol, 10.0 equiv.) and K_2CO_3 (2.6 g, 19 mmol, 3.0 equiv.) in CH_3CN (6.4 mL) for 2 days. After purification of the bromide by flash chromatography using pent/EtOAc 80:20, the elimination was performed using *t*BuOK (645 mg, 5.75 mmol, 1.5 equiv.) in THF (20 mL) for 1 h. The vinyl sulfonamide **29** was obtained as white solid after a recrystallisation in diethyl ether (930 mg, 2.62 mmol, 41%).

Rf = 0.29 (pentane/EtOAc 9:1).
^{1}H NMR (400 MHz, $CDCl_3$) δ 7.71 – 7.65 (m, 2H, Ts), 7.62 (dt, *J* = 7.9, 1.0 Hz, 1H, Ar*H*), 7.33 – 7.21 (m, 4H, Ts, Ar*H*), 7.14 (ddd, *J* = 8.0, 6.8, 1.2 Hz, 1H, C*H*), 6.99 (dd, *J* = 15.6, 9.4 Hz, 1H, Ar*H*), 6.89 (s, 1H, Ar*H*), 4.46 – 4.41 (m, 2H, C*H*$_2$), 3.73 (s, 3H, C*H*$_3$), 3.63 – 3.56 (m, 2H, C*H*$_2$), 3.10 – 3.04 (m, 2H, C*H*$_2$), 2.39 (s, 3H, C*H*$_3$).
^{13}C NMR (101 MHz, $CDCl_3$) δ 143.8, 137.1, 136.4, 132.2, 129.9, 127.7, 126.9, 126.9, 121.8, 119.1, 118.9, 111.0, 109.4, 92.6, 45.9, 32.7, 23.3, 21.6.
HRMS (ESI/QTOF) m/z: [M + H]$^+$ Calcd for $C_{20}H_{23}N_2O_2S^+$ 355.1475; Found 355.1476.
NMR spectra are in agreement with literature data.[21]

2.1.2. Cyclopropanation of enamides

The aminocyclopropanes were synthesized by a copper-catalyzed cyclopropanation of the corresponding protected enamines or the previously prepared vinyl sulfonamides (Scheme S5).

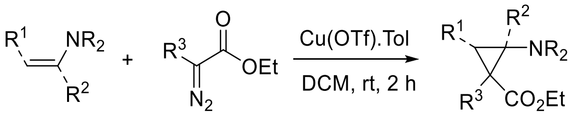

Scheme S5. Cyclopropanation of vinyl sulfonamide.

General procedure D for the cyclopropanation

A two-necked RBF was charged with Cu(OTf) toluene complex (2 mol%) in a glovebox. Outside the glovebox and under a nitrogen atmosphere, the enamine (1 equiv.) diluted in dry DCM was introduced. The diazo reagent (1.2 to 8.0 equiv.) was introduced slowly (manually or via a syringe pump) at rt or 0 °C for 1 to 2 h while the reaction was stirred moderately. When full conversion was reached, the solvent was evaporated and the crude was purified by flash chromatography.

For some tosyl-protected aminocyclopropanes, the crude diastereomeric mixture obtained after the cyclopropanation step could be converted to the pure trans isomer thanks to an isomerization under acidic condition (TsOH.H_2O). This operation was specified in the cyclopropanation procedure when accomplished.

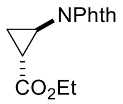
1a

Chemical Formula: $C_{14}H_{13}NO_4$
Molecular Weight: 259.2610

Ethyl 2-(1,3-dioxoisoindolin-2-yl)cyclopropane-1-carboxylate (1a): Prepared according to the general procedure D from *N*-vinylphthalimide (0.50 g, 2.9 mmol, 1.0 equiv.), EDA (87%, 1.7 mL, 14 mmol, 5.0 equiv.), Cu(OTf) toluene complex (30 mg, 58 µmol, 2 mol%) in DCM (19 mL) for 1 h. The crude product was purified by flash chromatography using pent/EtOAc 80:20 furnishing the aminocyclopropane as mixture of diastereomers (477 mg, 1.84 mmol, 63%, dr 64:36). The pure *trans* isomer **1a** was finally obtained as a colorless solid by recrystallisation in a pent/EtOAc mixture (143 mg, 551 µmol, 19%). The *cis* isomer could not be isolated in a pure fraction.

Rf = 0.45 (pent/EtOAc 4:1).
^{1}H NMR (400 MHz, $CDCl_3$) δ 7.86 – 7.80 (m, 2H, Ar*H*), 7.75 – 7.69 (m, 2H, Ar*H*), 4.21 (q, *J* = 7.1 Hz, 2H, C*H*$_2$), 3.35 – 3.29 (m, 1H, C*H*), 2.25 – 2.18 (m, 1H, C*H*), 1.76 (dt, *J* = 9.2, 5.5 Hz, 1H, C*H*$_2$), 1.64 (dt, *J* = 8.1, 5.9 Hz, 1H, C*H*$_2$), 1.31 (t, *J* = 7.1 Hz, 3H, C*H*$_3$).
^{13}C NMR (101 MHz, $CDCl_3$): δ 172.2, 168.1, 134.3, 131.6, 123.4, 61.2, 29.6, 20.0, 14.3, 13.6. NMR spectra are in agreement with the reported data.[22]

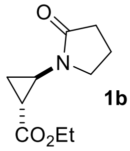
1b

Chemical Formula: $C_{10}H_{15}NO_3$
Molecular Weight: 197.2340

Ethyl 2-(2-oxopyrrolidin-1-yl)cyclopropane-1-carboxylate (1b): Prepared according to the general procedure D from *N*-vinyl-2-pyrrolidone (500 µL, 4.68 mmol, 1.0 equiv.), ethyl diazoacetate (87%, 2.30 mL, 18.8 mmol, 4.0 equiv.) and Cu(OTf) toluene complex (48 mg, 94 µmol, 2 mol%) in DCM (12 mL) for 1 h. The crude product was purified by flash chromatography using pent/EtOAc 60:40 to 100% EtOAc to afford **1b** as the *trans* product (134 mg, 679 µmol, 14%). The *cis* isomer could not be isolated in a pure fraction.

Rf = 0.32 (EtOAc).
^{1}H NMR (400 MHz, $CDCl_3$) δ 4.14 (qd, *J* = 7.2, 1.5 Hz, 2H, OC*H*$_2$CH$_3$), 3.31 (t, *J* = 7.0 Hz, 2H, C*H*$_2$), 3.17 (ddd, *J* = 8.3, 5.3, 3.1 Hz, 1H, C*H*), 2.38 (t, *J* = 8.1 Hz, 2H, C*H*$_2$), 2.04 – 1.96 (m, 2H, C*H*$_2$), 1.85 (ddd, *J* = 9.1, 5.9, 3.1 Hz, 1H, C*H*), 1.50 – 1.38 (m, 2H, C*H*$_2$), 1.27 (t, *J* = 7.2 Hz, 3H, OCH$_2$C*H*$_3$).
^{13}C NMR (101 MHz, $CDCl_3$): δ 175.9, 172.2, 60.8, 47.2, 34.0, 31.6, 19.7, 18.0, 14.2, 14.0.
NMR spectra are in agreement with the reported data.[22]

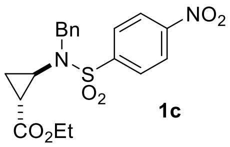

Chemical Formula: $C_{19}H_{20}N_2O_6S$
Molecular Weight: 404.4370

Ethyl-2-((*N*-benzyl-4-nitrophenyl)sulfonamido)cyclopropane-1-carboxylate (1c): Prepared according to the general procedure D from vinyl sulfonamide **20** (95 mg, 0.30 mmol, 1.0 equiv.), ethyl diazoacetate (87%, 145 µL, 1.19 mmol, 4.0 equiv.), Cu(OTf) toluene complex (3 mg, 6 µmol, 2 mol%) in DCM (1.5 mL) for 1 h. The crude was purified by flash chromatography using pent/EtOAc 85:15 to afford the *trans* cyclopropane **1c** as a viscous oil (47 mg, 0.12 mmol, 39%). The *cis* isomer was not isolated.

Rf = 0.43 (pentane/EtOAc 4:1).
^{1}H NMR (400 MHz, $CDCl_3$): δ 8.40 – 8.32 (m, 2H, Ns), 8.00 – 7.93 (m, 2H, Ns), 7.33 – 7.29 (m, 3H, Ar*H*), 7.29 – 7.24 (m, 2H, Ar*H*), 4.51 (d, *J* = 14.3 Hz, 1H, CH_2), 4.22 (d, *J* = 14.3 Hz, 1H, CH_2), 4.09 (qq, *J* = 7.1, 3.7 Hz, 2H, OCH_2CH_3), 2.45 (ddd, *J* = 7.6, 4.9, 2.8 Hz, 1H, C*H*), 1.76 (ddd, *J* = 9.3, 6.2, 2.9 Hz, 1H, CH_2), 1.40 (dt, *J* = 9.6, 5.4 Hz, 1H, CH_2), 1.35 – 1.24 (m, 1H, CH_2), 1.24 (t, *J* = 7.1 Hz, 3H, OCH_2CH_3).
^{13}C NMR (101 MHz, $CDCl_3$): δ 171.8, 150.4, 143.7, 135.2, 129.0 (2C), 128.9, 128.5, 124.5, 61.2, 55.1, 38.5, 22.6, 16.3, 14.4.
HRMS (nanochip-ESI/LTQ-Orbitrap) m/z: $[M + Na]^+$ Calcd for $C_{19}H_{20}N_2NaO_6S^+$ 427.0934; Found 427.0944.

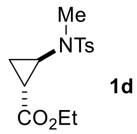

Chemical Formula: $C_{14}H_{19}NO_4S$
Molecular Weight: 297.3690

Ethyl 2-((*N*,4-dimethylphenyl)sulfonamido)cyclopropane-1-carboxylate (1d): Prepared according to the general procedure D from vinyl sulfonamide **18** (3.0 g, 14 mmol, 1.0 equiv.), ethyl diazoacetate (87%, 6.8 mL, 56 mmol, 4.0 equiv.), Cu(OTf) toluene complex (145 mg, 280 µmol, 2 mol%) in DCM (28 mL) for 1 h. At the end of the reaction TsOH.H_2O (0.1 equiv.) was added to the reaction mixture and the stirring was continued for 48 h at rt allowing a complete isomerization to the *trans* product. After removal of the solvent under reduced pressure, the crude was purified by flash chromatography using pent/EtOAc 85:15 to 75:25 to afford cyclopropane **1d** as white solid (3.0 g, 10 mmol, 72%).

Data for the trans *isomer*
Rf = 0.59 (pent/EtOAc 4:1).
m. p. = 56.6 – 57.6 °C.
^{1}H NMR (400 MHz, $CDCl_3$): δ 7.71 – 7.65 (m, 2H, Ts), 7.37 – 7.30 (m, 2H, Ts), 4.13 (qd, *J* = 7.1, 0.9 Hz, 2H, OCH_2CH_3), 2.72 (s, 3H, CH_3), 2.43 (s, 3H, CH_3), 2.27 (ddd, *J* = 7.4, 4.7, 2.7 Hz, 1H, C*H*), 2.02 (ddd, *J* = 9.0, 6.0, 2.7 Hz, 1H, C*H*), 1.54 (m, 1H, CH_2), 1.36 (dt, *J* = 7.4, 5.7 Hz, 1H, CH_2), 1.26 (t, *J* = 7.1 Hz, 3H, OCH_2CH_3).
^{13}C NMR (101 MHz, $CDCl_3$): δ 172.3, 144.1, 132.4, 129.9, 128.1, 61.1, 40.2, 37.6, 22.4, 21.7, 17.1, 14.3.
IR (ν_{max}, cm^{-1}) 2982 (m), 2931 (m), 1725 (s), 1408 (s), 1348 (s), 1173 (s).
HRMS (ESI/QTOF) m/z: $[M + Na]^+$ Calcd for $C_{14}H_{19}NNaO_4S^+$ 320.0927; Found 320.0926.

Data for the cis *isomer* (the isomerization was not performed after the cyclopropanation).
Rf = 0.28 (pentane/EtOAc 4:1).
^{1}H NMR (400 MHz, $CDCl_3$) δ 7.74 – 7.70 (m, 2H, Ts), 7.35 – 7.30 (m, 2H, Ts), 4.24 – 4.10 (m, 2H, OCH_2CH_3), 2.72 (s, 3H, CH_3), 2.42 (s, 3H, CH_3), 2.34 (td, *J* = 7.4, 5.4 Hz, 1H, C*H*), 1.89 (dt, *J* = 8.4, 6.9 Hz, 1H, C*H*), 1.72 (dt, *J* = 6.7, 5.6 Hz, 1H, CH_2), 1.28 (t, *J* = 7.1 Hz, 3H, OCH_2CH_3), 1.21 (ddd, *J* = 8.4, 7.5, 5.8 Hz, 1H, CH_2).
^{13}C NMR (101 MHz, $CDCl_3$): δ 169.6, 143.8, 133.5, 129.7, 128.1, 61.1, 38.7, 37.3, 22.1, 21.7, 14.4, 14.0.

IR (ν_{max}, cm^{-1}) 2980 (m), 1728 (s), 1346 (s), 1165 (s).
HRMS (ESI/QTOF) m/z: $[M + Na]^+$ Calcd for $C_{14}H_{19}NNaO_4S^+$ 320.0927; Found 320.0928.

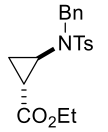

1g

Chemical Formula: $C_{20}H_{23}NO_4S$
Molecular Weight: 373.4670

Ethyl-2-((N-benzyl-4-methylphenyl)sulfonamido)cyclopropane-1-carboxylate (1g): Prepared according to the general procedure D from vinyl sulfonamide **19** (1.0 g, 3.5 mmol, 1.0 equiv.), EDA (87%, 1.7 mL, 14 mmol, 4.0 equiv.) and Cu(OTf) toluene complex (36 mg, 70 µmol, 2 mol%) in DCM (7 mL) for 1 h. At the end of the reaction, TsOH.H_2O (0.1 equiv.) was added to the reaction mixture and the stirring was continued for 72 h at rt allowing a complete isomerization to the *trans* product. After removal of the solvent under reduced pressure, the crude was purified by flash chromatography using pent/EtOAc 85:15 to afford cyclopropane **1g** as white solid (890 mg, 2.38 mmol, 69%).

Data for the trans *isomer*
Rf = 0.7 (pentane/EtOAc 4:1).
m. p. = 102 – 103.1 °C.
^{1}H NMR (400 MHz, $CDCl_3$): δ 7.75 – 7.69 (m, 2H, Ts), 7.36 – 7.32 (m, 2H, Ts), 7.32 – 7.27 (m, 5H, Ar*H*), 4.46 (d, *J* = 14.1 Hz, 1H, C*H*$_2$), 4.11 (d, *J* = 14.0 Hz, 1H, C*H*$_2$), 4.05 (qd, *J* = 7.1, 4.0 Hz, 2H, OC*H*$_2$CH$_3$), 2.45 (s, 3H, C*H*$_3$), 2.35 (ddd, *J* = 7.6, 4.9, 2.8 Hz, 1H, C*H*), 1.66 (ddd, *J* = 9.2, 6.2, 2.8 Hz, 1H, C*H*), 1.34 (dt, *J* = 9.5, 5.2 Hz, 1H, C*H*$_2$), 1.25 – 1.15 (m, 4H, C*H*$_2$, OCH$_2$C*H*$_3$).
^{13}C NMR (101 MHz, $CDCl_3$): δ 172.2, 144.0, 136.1, 134.6, 129.9, 128.9, 128.6, 128.0, 127.8, 60.8, 55.1, 38.9, 22.1, 21.7, 16.0, 14.3.
IR (ν_{max}, cm^{-1}) 3060 (m), 2987 (m), 1724 (s), 1341 (s), 1162 (s), 1094 (s), 704 (s).
HRMS (ESI/QTOF) m/z: $[M + H]^+$ Calcd for $C_{20}H_{24}NO_4S^+$ 374.1421; Found 374.1419.

Data for the cis *isomer* (the isomerization was not performed after the cyclopropanation).
Rf = 0.43 (pentane/EtOAc 4:1).
^{1}H NMR (400 MHz, $CDCl_3$): δ 7.77 – 7.73 (m, 2H, Ts), 7.36 – 7.22 (m, 7H, Ts, Ar*H*), 4.57 (d, *J* = 15.3 Hz, 1H, C*H*$_2$), 4.22 (d, *J* = 15.3 Hz, 1H, C*H*$_2$), 4.12 – 3.91 (m, 2H, OC*H*$_2$CH$_3$), 2.66 – 2.60 (m, 1H, C*H*), 2.42 (s, 3H, C*H*$_3$), 1.80 (dt, *J* = 8.5, 6.8 Hz, 1H, C*H*), 1.38 (q, *J* = 6.0 Hz, 1H, C*H*$_2$), 1.24 (t, *J* = 7.2 Hz, 3H, OCH$_2$C*H*$_3$), 1.05 (td, *J* = 8.2, 6.0 Hz, 1H, C*H*$_2$).
^{13}C NMR (101 MHz, $CDCl_3$): δ 170.0, 143.6, 136.4, 136.1, 129.6, 128.7, 128.5, 127.9, 127.7, 60.9, 53.8, 37.3, 21.9, 21.6, 14.3, 14.1.
IR (ν_{max}, cm^{-1}) 3061 (m), 2988 (m), 1724 (s), 1341 (s), 1162 (s), 1094 (s), 704 (s).
HRMS (ESI/QTOF) m/z: $[M + H]^+$ Calcd for $C_{20}H_{24}NO_4S^+$ 374.1421; Found 374.1421.

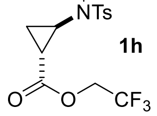

1h

Chemical Formula: $C_{14}H_{16}F_3NO_4S$
Molecular Weight: 351.3402

2,2,2-Trifluoroethyl-2-((*N*,4-dimethylphenyl)sulfonamido)cyclopropane-1-carboxylate (1h): Prepared according to the general procedure D from vinyl sulfonamide **18** (55 mg, 0.26 mmol, 1.0 equiv.), Cu(OTf) toluene complex (2.7 mg, 5.2 µmol, 2.0 mol%), 2,2,2-trifluoroethyl 2-diazoacetate (53 mg, 0.31 mmol, 1.2 equiv.) in DCM (2 mL) for 2 h. The 2,2,2-trifluoroethyl 2-diazoacetate was diluted in DCM (0.6 ml) and introduced via a syringe pump over a period of 1 h (~ 0.6 mL/h). The crude was purified by flash chromatography using pent/EtOAc 85:15 to afford the *trans* **1h** (34 mg, 97 µmol, 37%) and the *cis* isomer **1h’** (32 mg, 90 µmol, 35%) as colorless oils. The *cis* isomer can also be isomerized to the *trans* isomer using TsOH.H_2O (0.1 equiv.) in DCM for 16 h.

Data for the trans *isomer*

Rf = 0.66 (pentane/EtOAc 4:1).
^{1}H NMR (400 MHz, $CDCl_3$): δ 7.70 – 7.65 (m, 2H, Ts), 7.37 – 7.32 (m, 2H, Ts), 4.48 (qq, *J* = 8.3, 4.3 Hz, 2H, OCH_2CF_3), 2.74 (s, 3H, CH_3), 2.44 (s, 3H, CH_3), 2.37 (ddd, *J* = 7.5, 4.9, 2.7 Hz, 1H, C*H*), 2.14 (ddd, *J* = 9.4, 6.0, 2.7 Hz, 1H, C*H*), 1.64 (dt, *J* = 9.4, 5.5 Hz, 1H, CH_2), 1.45 (dt, *J* = 7.5, 5.5 Hz, 1H, CH_2).
^{13}C NMR (101 MHz, $CDCl_3$): δ 170.7, 144.3, 132.3, 130.0, 128.1, 122.9 (q, *J* = 277.4 Hz), 60.7 (q, *J* = 36.7 Hz), 40.8, 37.4, 22.0, 21.7, 17.6.
^{19}F NMR (376 MHz, $CDCl_3$): δ -73.8 (t, *J* = 8.2 Hz).
IR (ν_{max}, cm^{-1}) 3028 (w), 2978 (w), 1750 (m), 1352 (m), 1282 (m), 1163 (s), 752 (m).
HRMS (nanochip-ESI/LTQ-Orbitrap) m/z: $[M + H]^+$ Calcd for $C_{14}H_{17}F_3NO_4S^+$ 352.0825; Found 352.0813.

Data for the cis *isomer*

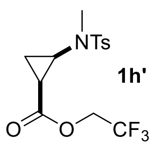
1h'

Chemical Formula: $C_{14}H_{16}F_3NO_4S$
Molecular Weight: 351.3402

Rf = 0.42 (pentane/EtOAc 4:1).
^{1}H NMR (400 MHz, $CDCl_3$): δ 7.74 – 7.69 (m, 2H, Ts), 7.36 – 7.31 (m, 2H, Ts), 4.65 – 4.53 (m, 1H, OCH_2CF_3), 4.48 – 4.36 (m, 1H, OCH_2CF_3), 2.74 (s, 3H, CH_3), 2.43 (s, 3H, CH_3), 2.42 – 2.37 (m, 1H, C*H*), 2.04 (dt, *J* = 8.4, 6.9 Hz, 1H, C*H*), 1.72 (q, *J* = 6.0 Hz, 1H, CH_2), 1.30 (td, *J* = 7.8, 6 Hz, 1H, CH_2).
^{13}C NMR (101 MHz, $CDCl_3$): δ 168.2, 144.1, 133.1, 129.8, 128.1, 123.2 (q, *J* = 277.2 Hz), 60.9 (q, *J* = 36.6 Hz), 39.2, 37.4, 21.9, 21.7, 14.4.
^{19}F NMR (376 MHz, $CDCl_3$) δ -73.6 (t, *J* = 8.5 Hz).
IR (ν_{max}, cm^{-1}) 3030 (w), 2976 (w), 1755 (m), 1348 (m), 1281 (m), 1158 (s), 697 (s).
HRMS (nanochip-ESI/LTQ-Orbitrap) m/z: $[M + H]^+$ Calcd for $C_{14}H_{17}F_3NO_4S^+$ 352.0825; Found 352.0812.

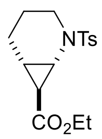
1i

Chemical Formula: $C_{16}H_{21}NO_4S$
Molecular Weight: 323.4070

Ethyl-2-tosyl-2-azabicyclo[4.1.0]heptane-7-carboxylate (1i): Prepared according to the general procedure D from vinyl sulfonamide **24** (400 mg, 1.69 mmol, 1.0 equiv.), (87%, 1.0 mL, 8.4 mmol, 5.0 equiv.), Cu(OTf) toluene complex (17 mg, 34 µmol, 2.0 mol%) in DCM (3.4 mL) for 2 h. The crude product was purified by flash chromatography using pent/EtOAc 80:20 to afford **1i** as a colorless oil (379 mg, 1.17 mmol, 70%).

Rf = 0.46 (pentane/EtOAc 4:1).
^{1}H NMR (400 MHz, $CDCl_3$): δ 7.68 (d, *J* = 8.3 Hz, 2H, Ts), 7.29 (d, *J* = 8.0 Hz, 2H, Ts), 4.19 – 4.03 (m, 2H, OCH_2CH_3), 3.40 (dd, *J* = 8.8, 2.7 Hz, 1H, C*H*), 3.40 – 3.31 (m, 1H, CH_2), 2.86 (ddd, *J* = 13.0, 11.0, 2.4 Hz, 1H, CH_2), 2.43 (s, 3H, CH_3), 1.88 – 1.66 (m, 3H, CH_2, C*H*), 1.61 – 1.52 (m, 1H, CH_2), 1.26 (t, *J* = 7.1 Hz, 3H, OCH_2CH_3), 1.24 – 1.06 (m, 2H, CH_2, C*H*).
^{13}C NMR (101 MHz, $CDCl_3$): δ 171.5, 143.6, 135.8, 129.8, 127.5, 60.7, 42.9, 38.2, 24.7, 21.7, 21.5, 20.6, 19.4, 14.4.
IR (ν_{max}, cm^{-1}) 3027 (w), 2938 (m), 1719 (m), 1343 (s), 1159 (s), 921 (m), 735 (s).
HRMS (ESI/QTOF) m/z: $[M + Na]^+$ Calcd for $C_{16}H_{21}NNaO_4S^+$ 346.1084; Found 346.1080.

Ethyl-2-((*N*,4-dimethylphenyl)sulfonamido)-2-methylcyclopropane-1-carboxylate (1j)

1j

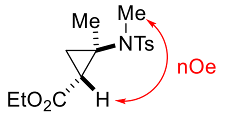

Chemical Formula: $C_{15}H_{21}NO_4S$
Molecular Weight: 311.3960

Prepared according to the general procedure D from vinyl sulfonamide **26** (250 mg, 1.11 mmol, 1.0 equiv.), ethyl diazoacetate (87%, 1.1 mL, 8.88 mmol, 8.0 equiv.) and Cu(OTf) toluene complex (12 mg, 22 µmol, 2.0 mol%) in DCM (5.5 mL) for 1 h. The crude product was purified by flash chromatography using pent/EtOAc 85:15 to afford a pure diastereomer **1j** as colorless oil (79 mg, 0.25 mmol, 23%).

Rf = 0.47 (pentane/EtOAc 4:1).
^{1}H NMR (400 MHz, $CDCl_3$): δ 7.73 – 7.69 (m, 2H, Ts), 7.32 – 7.28 (m, 2H, Ts), 4.22 – 4.09 (m, 2H, OCH_2CH_3), 2.86 (s, 3H, CH_3), 2.43 (s, 3H, CH_3), 2.04 (dd, J = 9.4, 6.9 Hz, 1H, CH), 1.64 (dd, J = 9.4, 5.7 Hz, 1H, CH_2), 1.33 (dd, J = 7.0, 5.7 Hz, 1H, CH_2), 1.27 (t, J = 7.1 Hz, 3H, OCH_2CH_3), 1.22 (s, 3H, CH_3).
^{13}C NMR (101 MHz, $CDCl_3$): δ 170.9, 143.7, 137.1, 129.8, 127.7, 61.0, 45.1, 34.5, 29.2, 23.2, 21.7, 14.5, 14.1.
IR (ν_{max}, cm^{-1}) 3067 (w), 2939 (w), 1727 (m), 1343 (s), 1163 (s), 913 (m).
HRMS (ESI/QTOF) m/z: [M + H]$^+$ Calcd for $C_{15}H_{22}NO_4S^+$ 312.1264; Found 312.1262.

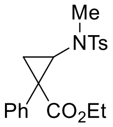

1l

Chemical Formula: $C_{20}H_{23}NO_4S$
Molecular Weight: 373.4670

Ethyl 2-((*N*,4-dimethylphenyl)sulfonamido)-1-phenylcyclopropane-1-carboxylate (1l): Prepared according to a slightly modification of the general procedure D from vinyl sulfonamide **18** (356 mg, 1.68 mmol, 2.0 equiv.), ethyl diazo(phenyl)acetate[23] (160 mg, 843 µmol, 1.0 equiv.) diluted in 0.7 mL of DCM and Cu(OTf) toluene complex (8.7 mg, 17 µmol, 2.0 mol%) in DCM (4.2 mL). Ethyl diazo(phenyl)acetate was introduced via a syringe pump (1 mL/h). The crude was purified by flash chromatography using pent/EtOAc 85:15 to afford cyclopropane **1l** in mixture of diastereomer as a colorless oil (85 mg, 0.23 mmol, 27%).

Characterized as a 60:40 mixture of inseparable diasteromers

Rf = 0.63 (pentane/EtOAc 4:1).
^{1}H NMR (400 MHz, $CDCl_3$, signals for major diastereomer): δ 7.61 – 7.57 (m, 2H, Ts), 7.32 – 7.20 (m, 7H, Ts, ArH), 4.15 – 4.06 (m, 2H, OCH_2CH_3), 2.83 – 2.79 (m, 1H, CH), 2.46 – 2.42 (m, 1H, CH_2), 2.41 (s, 3H, CH_3), 2.39 (s, 3H, CH_3), 1.77 (dd, J = 8.2, 6.2 Hz, 1H, CH_2), 1.11 (t, J = 7.1 Hz, 3H, OCH_2CH_3).
^{13}C NMR (101 MHz, $CDCl_3$, both diastereomers): δ 172.8, 169.6, 143.9, 143.8, 138.4, 133.6, 133.5, 132.6, 130.9, 130.3, 129.83, 129.81, 128.4, 128.0, 127.9, 127.8, 127.69, 127.67, 61.7, 61.6, 46.0, 45.9, 37.6, 37.3, 37.0, 35.3, 21.7, 21.6, 21.0, 20.1, 14.3, 14.2.
IR (ν_{max}, cm^{-1}) 3060 (w), 2937 (w), 1719 (s), 1345 (s), 1270 (m), 1168 (s), 725 (s).
HRMS (ESI/QTOF) m/z: [M + Na]$^+$ Calcd for $C_{20}H_{23}NNaO_4S^+$ 396.1240; Found 396.1239.

Ethyl-2-((4-methyl-*N*-(2-(1-methyl-indol-3-yl)ethyl)phenyl)sulfonamido)cyclopropane-1-carboxylate (1n)

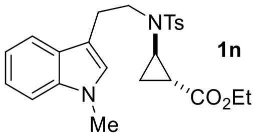

Chemical Formula: $C_{24}H_{28}N_2O_4S$
Molecular Weight: 440.5580

Prepared according to the general procedure D from vinyl sulfonamide **29** (357 mg, 1.01 mmol, 1.0 equiv.), EDA (87%, 974 µL, 8.05 mmol, 8.0 equiv.) and Cu(OTf) toluene complex (10 mg, 20 µmol, 2.0 mol%) in DCM (5 mL) for 1 h at 0 °C. After two flash chromatographies using pent/EtOAc 80:20 for the first column and toluene/EtOAc 96:4 for the second column, cyclopropane **1n** was isolated as a viscous oil (53 mg, 0.12 mmol, 12%).

Rf = 0.43 (toluene/EtOAc 95:5).

^{1}H NMR (400 MHz, $CDCl_3$): δ 7.75 – 7.69 (m, 2H, Ts), 7.56 (d, *J* = 8.0 Hz, 1H, Ar*H*), 7.33 – 7.26 (m, 3H, Ts, Ar*H*), 7.25 – 7.20 (m, 1H, Ar*H*), 7.13 – 7.08 (m, 1H, Ar*H*), 6.87 (s, 1H, Ar*H*), 4.15 (q, *J* = 7.1 Hz, 2H, OCH_2CH_3), 3.75 (s, 3H, CH_3), 3.58 – 3.48 (m, 1H, CH_2), 3.34 (dt, *J* = 13.9, 7.6 Hz, 1H, CH_2), 3.06 – 3.01 (m, 2H, CH_2), 2.52 (ddd, *J* = 7.4, 4.8, 2.8 Hz, 1H, C*H*), 2.42 (s, 3H, CH_3), 1.99 (ddd, *J* = 9.2, 6.1, 2.8 Hz, 1H, C*H*), 1.49 (dt, *J* = 9.6, 5.1 Hz, 1H, CH_2), 1.37 (dt, *J* = 7.4, 5.8 Hz, 1H, CH_2), 1.29 (t, *J* = 7.1 Hz, 3H, OCH_2CH_3).

^{13}C NMR (101 MHz, $CDCl_3$): δ 172.3, 143.9, 137.1, 135.1, 129.9, 127.8, 127.7, 127.1, 121.8, 119.1, 118.8, 111.1, 109.4, 61.1, 52.2, 39.0, 32.8, 25.0, 22.6, 21.7, 16.7, 14.4.

IR (ν_{max}, cm^{-1}) 3054 (m), 2980 (m), 2930 (m), 1725 (s), 1467 (s), 1337 (s), 1163 (s), 739 (s).

HRMS (ESI/QTOF) m/z: $[M + H]^+$ Calcd for $C_{24}H_{29}N_2O_4S^+$ 441.1843; Found 441.1844.

2.1.3. Synthesis of other aminocyclopropanes (1e, 1f, 1k, 1m) from 1d

***N*,4-dimethyl-N-((1R,2R)-2-(2-oxooxazolidine-3carbonyl)cyclopropyl)benzenesulfonamide (1e)**:

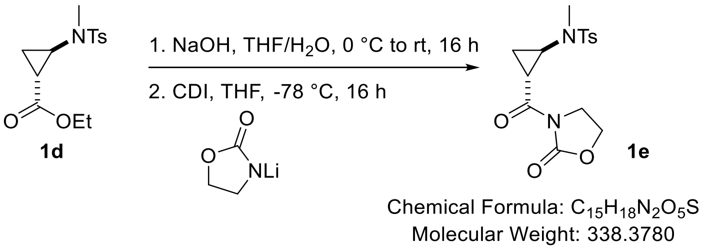

Aminocyclopropane **1d** (255 mg, 856 µmol, 1.0 equiv.) was diluted in a mixture of THF/H_2O 1:1 and NaOH (342 mg, 8.55 mmol, 10.0 equiv.) was added at 0 °C. The reaction mixture was then allowed to stir at rt for 16 h. The solvents were evaporated and the crude was diluted with water. The aqueous layer was extracted with ether and was then acidified to pH 2 with a 1 M HCl aqueous solution. The acidic aqueous layer was finally extracted with DCM and the combined organic layers were dried over Na_2SO_4, filtered and concentrated under reduced pressure. The crude product was directly used in the next step.

In a two-necked RBF, 1,1'-carbonyldiimidazole (141 mg, 870 µmol, 1.1 equiv.) was added in one portion at 0 °C to a solution of the acid (213 mg, 791 µmol, 1.0 equiv.) diluted in THF (8 mL). The reaction mixture was then stirred at rt for 1 h and then cooled to -78 °C. To this solution was added dropwise the lithiated oxazolidinone, previously prepared by the addition of *n*-BuLi (2.5 M in hexanes, 633 µL, 1.58 mmol, 2.0 equiv.) to a solution of 2-oxazolidone (138 mg, 1.58 mmol, 2.0 equiv.) at 0 °C in a minimum amount of THF. The mixture was then stirred for 16 h allowing to reach rt slowly and finally quenched with a NH_4Cl saturated aqueous solution. The aqueous layer was extracted with EtOAc and the combined organic layers were washed with brine, dried over Na_2SO_4, filtered off and concentrated under reduced pressure. The crude product was purified by flash chromatography using DCM/EtOAc 90:10 to afford cyclopropane **1e** as a white solid (125 mg, 369 µmol, 43% for two steps).

Rf = 0.55 (DCM/EtOAc 75 :25).

m. p. = 89 – 92 °C.

^{1}H NMR (400 MHz, $CDCl_3$): δ 7.74 – 7.67 (m, 2H, Ts), 7.38 – 7.31 (m, 2H, Ts), 4.41 (dd, *J* = 8.5, 7.6 Hz, 2H, CH_2), 3.99 (dd, *J* = 8.5, 7.6 Hz, 2H, CH_2), 3.45 (ddd, *J* = 9.0, 6.0, 2.7 Hz, 1H, C*H*), 2.75 (s, 3H, CH_3), 2.49 (ddd, *J* = 7.5, 5.0, 2.7 Hz, 1H, C*H*), 2.44 (s, 3H, CH_3), 1.78 (dt, *J* = 9.5, 5.0 Hz, 1H, CH_2), 1.62 – 1.55 (m, 1H, CH_2).

^{13}C NMR (101 MHz, $CDCl_3$): δ 171.6, 153.8, 144.1, 132.8, 129.9, 128.1, 62.1, 42.9, 42.3, 37.4, 21.7, 21.3, 19.4.

IR (ν_{max}, cm^{-1}) 3066 (w), 2981 (w), 1732 (s), 1352 (s), 1162 (s), 745 (m).
HRMS (ESI/QTOF) m/z: $[M + H]^+$ Calcd for $C_{15}H_{19}N_2O_5S^+$ 339.1009; Found 339.1018.

2-((*N*,4-Dimethylphenyl)sulfonamido)-*N*-methoxy-*N*-methylcyclopropane-1-carboxamide (30)

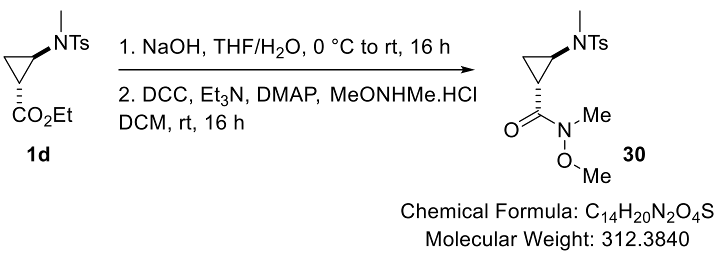

To a stirred solution of acid (step 1 see above, 0.74 mmol, 1.0 equiv.) diluted in dry DCM (4 mL) were added *N,O*-dimethylhydroxylamine hydrochloride (109 mg, 1.11 mmol, 1.5 equiv.), 4-dimethylaminopyridine (9.1 mg, 74 µmol, 0.1 equiv.) and *N,N*'-dicyclohexylcarbodiimide (230 mg, 1.11 mmol, 1.5 equiv.). Then triethylamine (155 µL, 1.11 mmol, 1.5 equiv.) was added at rt and the reaction mixture was stirred for 16 h. After evaporation of the solvent, the crude was purified by flash chromatography using pent/EtOAc 60:40 to afford the Weinred amide **30** as a colorless oil (130 mg, 416 µmol, 55% for two steps).

Rf = 0.31 (pent/EtOAc 1:1).
^{1}H NMR (400 MHz, $CDCl_3$): 7.71 – 7.65 (m, 2H, Ts), 7.36 – 7.31 (m, 2H, Ts), 3.83 (s, 3H, C*H*3), 3.20 (s, 3H, C*H*3), 2.72 (s, 3H, C*H*3), 2.69 – 2.63 (m, 1H, C*H*), 2.43 (s, 3H, C*H*3), 2.32 (ddd, *J* = 7.3, 4.5, 2.8 Hz, 1H C*H*), 1.49 – 1.41 (m, 1H, C*H*2), 1.36 – 1.29 (m, 1H, C*H*2).
^{13}C NMR (101 MHz, $CDCl_3$): δ 172.0, 144.0, 132.2, 129.9, 128.1, 62.0, 40.1, 37.7, 32.6, 21.7, 20.4, 16.6.
IR (ν_{max}, cm^{-1}) 2946 (w), 2857 (w), 1652 (s), 1451 (m), 1346 (s), 1167 (s).
HRMS (nanochip-ESI/LTQ-Orbitrap) m/z: $[M + H]^+$ Calcd for $C_{14}H_{21}N_2O_4S^+$ 313.1217; Found 313.1209.

N-2-Benzoylcyclopropyl)-*N*,4-dimethylbenzenesulfonamide (1f)

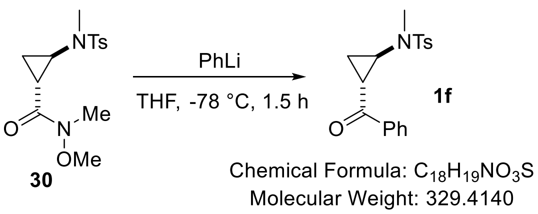

To a stirred solution of Weinreb amide **30** (192 mg, 614 µmol, 1.0 equiv.) in dry THF (5 mL) was added dropwise at -78 °C PhLi (1.9 M in dibutyl ether, 355 µL, 675 µmol, 1.1 equiv.). The reaction mixture was stirred at -78 °C for 1.5 h and quenched with a $NaHCO_3$ saturated aqueous solution. The aqueous layer was extracted with Et_2O. The combined organic layers were washed with brine, dried over Na_2SO_4 filtered off and concentrated under reduced pressure. The crude product was purified by flash chromatography using pent/EtOAc 85:15 to afford the cyclopropyl ketone **1f** as a colorless oil (155 mg, 470 µmol, 77%).

Rf = 0.61 (pentane/EtOAc 4:1).
^{1}H NMR (400 MHz, $CDCl_3$): δ 8.13 – 8.08 (m, 2H, Ar*H*), 7.63 – 7.58 (m, 3H, Ar*H*, Ts), 7.54 – 7.48 (m, 2H, Ar*H*), 7.30 – 7.26 (m, 2H, Ts), 3.27 (ddd, *J* = 8.7, 6.1, 2.7 Hz, 1H, C*H*), 2.77 (s, 3H, C*H*3), 2.45 – 2.39 (m, 4H, C*H*, C*H*3), 1.62 – 1.53 (m, 2H, C*H*2).
^{13}C NMR (101 MHz, $CDCl_3$): δ 198.0, 144.1, 137.4, 133.4, 132.0, 129.9, 128.8, 128.5, 128.0, 43.0, 37.7, 27.3, 21.6, 18.7.
IR (ν_{max}, cm^{-1}) 3063 (w), 2974 (w), 1667 (m), 1391 (m), 1348 (m), 1221 (m), 1165 (s), 722 (s).
HRMS (nanochip-ESI/LTQ-Orbitrap) m/z: $[M + Na]^+$ Calcd for $C_{18}H_{19}NNaO_3S^+$ 352.0978; Found 352.0968.

Ethyl-2-((*N*,4-dimethylphenyl)sulfonamido)-1-methylcyclopropane-1-carboxylate (1k)

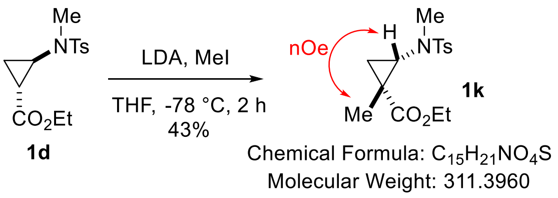

Cyclopropane **1d** (300 mg, 1.01 mmol, 1.0 equiv.) diluted in 2 mL of THF was added at -78 °C to a stirred solution of LDA, prepared from diisopropylamine (171 µL, 1.21 mmol, 1.2 equiv.) and *n*-BuLi (2.5 M in hexanes, 484 µL, 1.21 mmol, 1.2 equiv.) in THF (8 mL). The reaction mixture was stirred for 1 h at -78 °C and MeI (251 µL, 4.03 mmol, 4.0 equiv.) was added. The reaction was then stirred at -78 °C for 2 h (full conversion was observed) and quenched with a NH_4Cl saturated aqueous solution. The aqueous layer was extracted with diethyl ether and the combined organic layers were washed with brine, dried over Na_2SO_4, filtered off and concentrated under reduced pressure. The crude product was purified by flash chromatography using pent/EtOAc 85:15 to afford cyclopropane **1k** as a single isomer and a yellow oil (134 mg, 430 µmol, 43%).

Rf = 0.69 (pentane/EtOAc 4:1).
^{1}H NMR (400 MHz, $CDCl_3$): δ 7.73 – 7.68 (m, 2H, Ts), 7.37 – 7.32 (m, 2H, Ts), 4.09 (q, *J* = 7.1 Hz, 2H, OC*H*$_2$CH$_3$), 2.70 (s, 3H, C*H*$_3$), 2.44 (s, 3H, C*H*$_3$), 2.31 (dd, *J* = 8.0, 5.1 Hz, 1H, C*H*), 1.56 (dd, *J* = 8.0, 5.5 Hz, 1H, C*H*$_2$), 1.46 – 1.39 (m, 4H, C*H*$_2$, C*H*$_3$), 1.23 (t, *J* = 7.1 Hz, 3H, OCH$_2$C*H*$_3$).
^{13}C NMR (101 MHz, $CDCl_3$): δ 174.2, 144.0, 132.7, 129.9, 128.1, 61.2, 44.9, 38.3, 25.5, 23.6, 21.7, 14.3, 13.4.
IR (ν_{max}, cm^{-1}) 3058 (w), 2936 (w), 1718 (s), 1348 (m), 1296 (m), 1164 (s), 714 (s).
HRMS (ESI/QTOF) m/z: [M + Na]$^+$ Calcd for $C_{15}H_{21}NNaO_4S^+$ 334.1084; Found 334.1087.

N-(2-Allyl-2-((ethylperoxy)-l2-methyl)cyclopropyl)-*N*,4-dimethylbenzenesulfonamide (1m)

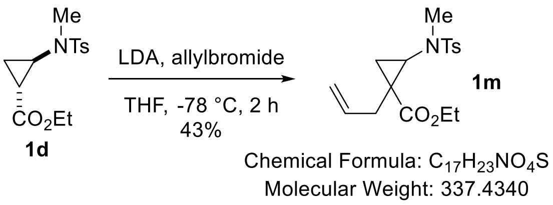

Cyclopropane **1d** (309 mg, 1.04 mmol, 1.0 equiv.) diluted in 2 mL of THF was added at -78 °C to a stirred solution of LDA, prepared from diisopropylamine (176 µL, 1.25 mmol, 1.2 equiv.) and *n*-BuLi (2.5 M in hexanes, 498 µL, 1.25 mmol, 1.2 equiv.) in THF (10 mL). The reaction mixture was stirred for 1 h at -78 °C and allyl bromide (181 µL, 2.08 mmol, 2 equiv.) was introduced. The reaction was then stirred for 2 h at -78 °C and quenched with a NH_4Cl saturated aqueous solution. The aqueous layer was extracted with diethyl ether and the combined organic layers were washed with brine, dried over Na_2SO_4, filtered off and concentrated under reduced pressure. The crude product was purified by flash chromatography using pent/EtOAc 90:10 to afford cyclopropane **1m** in mixture of diastereomers as colorless oil (152 mg, 450 µmol, 45%, dr 66:34). Separation of the mixture on PREP TLC (pent/EtOAc 90:10) allowed clean NMR characterizations for both diastereomers.

Data for the major diastereomer

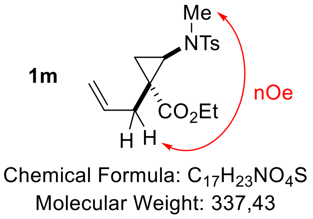

Rf = 0.7 (pentane/EtOAc 4:1).
^{1}H NMR (400 MHz, $CDCl_3$): δ 7.70 (d, *J* = 8.3 Hz, 2H, Ts), 7.35 (d, *J* = 8.0 Hz, 2H, Ts), 5.96 – 5.85 (m, 1H, C*H*), 5.14 – 5.02 (m, 2H, C*H*$_2$), 4.18 – 4.03 (m, 2H, OC*H*$_2$CH$_3$), 2.96 – 2.89 (m, 1H, C*H*$_2$), 2.71 (s, 3H, C*H*$_3$), 2.44 (s, 3H, C*H*$_3$), 2.30 (dd, *J* = 7.8, 5.3 Hz, 1H, NC*H*), 2.10 (ddt, *J* = 14.9, 6.7, 1.4 Hz, 1H, C*H*$_2$), 1.63 (ddd, *J* = 7.8, 5.7, 1.5 Hz, 1H, C*H*$_2$), 1.48 (t, *J* = 5.7 Hz, 1H, C*H*$_2$), 1.23 (t, *J* = 7.1 Hz, 3H, OC*H*$_2$C*H*$_3$).

^{13}C NMR (101 MHz, $CDCl_3$): δ 173.4, 144.1, 135.8, 132.7, 129.9, 128.1, 116.7, 61.2, 45.7, 38.4, 31.5, 29.7, 21.7, 21.5, 14.3.
IR (ν_{max}, cm^{-1}) 3076 (w), 2936 (w), 1720 (m), 1348 (s), 1165 (s), 719 (m).
HRMS (ESI/QTOF) m/z: $[M + Na]^+$ Calcd for $C_{17}H_{23}NNaO_4S^+$ 360.1240; Found 360.1247.

Data for the minor diastereomer

1m'

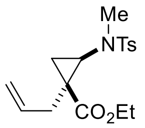

Chemical Formula: $C_{17}H_{23}NO_4S$
Molecular Weight: 337,43

Rf = 0.65 (pentane/EtOAc 4:1).
^{1}H NMR (400 MHz, $CDCl_3$): 7.72 – 7.68 (m, 2H, Ts), 7.35 – 7.31 (m, 2H, Ts), 5.87 – 5.76 (m, 1H, C*H*), 5.07 – 4.98 (m, 2H, CH_2), 4.28 – 4.09 (m, 2H, OCH_2CH$_3$), 2.77 – 2.72 (m, 1H, CH_2), 2.71 (s, 3H, CH_3), 2.44 (s, 3H, CH_3), 2.08 (dd, *J* = 7.4, 5.3 Hz, 1H, CH_2), 2.05 – 2.01 (m, 1H, C*H*), 1.95 (ddt, *J* = 14.9, 6.5, 1.4 Hz, 1H, CH_2) 1.28 (t, *J* = 7.1 Hz, 3H, OCH$_2$CH_3), 1.07 (dd, *J* = 7.4, 5.7 Hz, 1H, CH_2).
^{13}C NMR (101 MHz, $CDCl_3$): δ 170.5, 143.8, 134.6, 133.5, 129.7, 128.1, 117.2, 61.3, 44.6, 37.6, 37.2, 31.7, 21.7, 19.5, 14.4.
HRMS (ESI/QTOF) m/z: $[M + H]^+$ Calcd for $C_{17}H_{24}NO_4S^+$ 338.1421; Found 338.1419.

3. Screening of Lewis acids and optimization

Preliminary experiments showed that TMSOTf was not reactive enough in the dearomatization of 1-methylindole under catalytic conditions (Table S1).

Table S1. Screening of silyl Lewis acid.

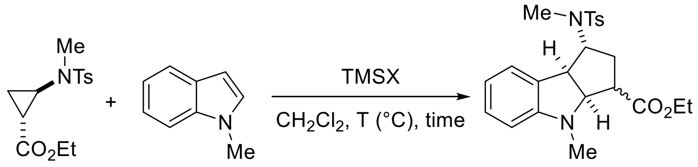

1d, 0.1M **2a**, 1.5 equiv. **4a**

Entry	X	mol%	T (°C)	Time (h)	Conv. (%)	Yield[a] (%)	dr[b]
1	OTf	20	-78 to rt	3	0	-	-
2	OTf	100	-78 to rt	3	0	-	-
3[c]	NTf_2	10	-78	2	100	79	4 :1

[a] Isolated yield; [b] determined from ^{1}H NMR spectrum; [c] $TMSNTf_2$ was formed by the pre-mixing of triflimide acid with silyl ketene acetal **3a**.

As a control experiment, triflimide did not lead to the formation of the cycloadduct (Table S2, entry 2). Other solvents than DCM, such as toluene and THF, were totally ineffective for this reaction (entry 3 and 4). Slower reaction rates were observed in DCE such that the temperature was increased to −20 °C producing the cycloadduct in a slightly lower yield and an identical diastereoselectivity (entry 5). The TBS catalyst (**3e**) did not show any influence on the diastereoselectivity and the $TIPSNTf_2$ (**3f**) was even worse leading to a 2.3:1 dr ratio (entry 6 and 7). The diastereoselectivity increased considerably with $TESNTf_2$ (**3b**) reaching an 8:1 dr ratio (entry 8). Running the same reaction at 0 °C led to no reaction (entry 9). The bulkier tri-*n*-propylsilyl moiety (**3d**) further improved the dr ratio to 9:1 (entry 10). At this point we were alerted by the influence of the DCM/Toluene ratio on the

diastereoselectivity (toluene comes from the stock solutions of Tf_2NH and silyl ketene acetal). Indeed, increasing the DCM/toluene ratio led to a dramatic drop in the diastereoselectivity (entry 11).

Table S2. First optimization of the (3+2) annulation of aminocyclopropyl carboxylate 1d.

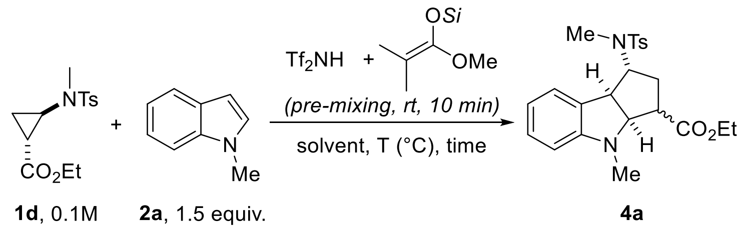

Entry	Tf_2NH[a] (mol%)	*Si*[a]	solvent	T (°C)	Time (h)	Conv. (%)	Yield[b] (%)	dr[c]
1	10	TMS	DCM	-78	2	100	79	4 :1
2	20	-	DCM	rt	16	0	-	-
3	5	TMS	Tol	rt	72	traces	-	-
4	5	TMS	THF	rt	72	0	-	-
5	5	TMS	DCE	-20	3	>95	74	4 :1
6	5	TBS	DCM	-78	2	66	52	4 :1
7	5	TIPS	DCM	-78	2	100	78	2.3 :1
8	5	TES	DCM	-78	1.5	100	87	8:1
9	5	TES	DCM	0	0.5	0	-	-
10	5	*n*Pr_3Si	DCM	-78	0.5	100	81	9:1
11[d]	5	*n*Pr_3Si	DCM/Tol	-78	0.5	100	85	4.9:1

[a] Homemade solutions of Tf_2NH and silyl ketene acetals (25 mol%) in dry toluene were prepared; [b] Isolated yield; [c] dr values were determined from the crude ^1H NMR spectrum or from the isolate fraction of both diastereomers; [d] a DCM/Tol ratio of 75:25 instead of 83:17 (entry 10) was used.

Even though stock solutions of Tf_2NH in DCE are more stable than those in DCM, we preffered to use freshly prepared stock solutions in dry DCM to avoid reproducibility issues. The influence of the silyl group on the diastereoselectivity was studied by repeating some experiments using 1.05 equivalent of 1-methylindole (see manuscript, Table 1). The diastereoselectivity followed this trend: TES>TMS>TBS>TIPS.

4. (3+2) annulation of D-A aminocyclopropanes with indoles

General procedure E for the (3+2) annulation of aminocyclopropanes

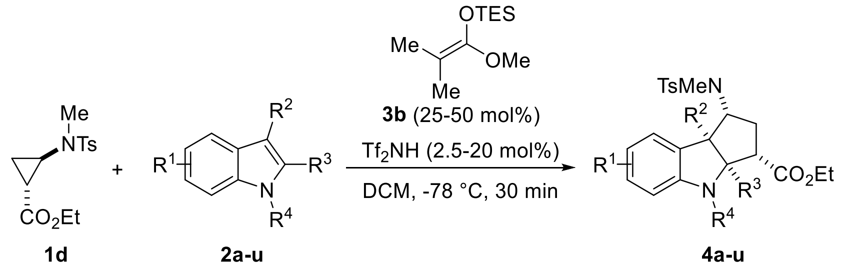

Scheme S6. (3+2) annulation of aminocyclopropane **1d** with indoles.

In an oven dried microwave vial, a freshly prepared DCM solution of bis(trifluoromethanesulfonyl)imide (2.5 to 20 mol%) was added to silyl ketene acetal **3b** (0.25 to 0.5 equiv.) diluted in 0.1 mL of dry DCM and the mixture was stirred for 10 minutes at rt. The mixture was then diluted with DCM and finally cooled to -78 °C. Aminocyclopropane **1d** (0.1 to 0.3 mmol, 1.0 equiv.) and the indole derivative (1.05 equiv.) were diluted together in DCM and added dropwise at -78 °C to the preformed catalyst. The reaction mixture was stirred at -78 °C for 30 minutes, unless otherwise stated. The reaction was quenched by addition of 10 µL of Et_3N and the solvent was concentrated under reduced pressure. The crude product was then purified by flash chromatography or PREP TLC. The dr ratio was measured from the ^{1}H NMR spectrum of the crude product or the isolated mixture of diastereoisomers by integrating the TsNC*H* proton of each diastereomer and was compared to the ratio obtained from the tosyl protons.

General procedure F for the scale up experiment (1 mmol of substrate)

In an oven dried microwave vial, a freshly prepared DCM solution of bis(trifluoromethanesulfonyl)imide (5 mol%) was added to silyl ketene acetal **3b** (0.25 mmol, 0.25 equiv.) diluted in 0.3 mL of dry DCM and the mixture was stirred for 10 minutes at rt. The mixture was then diluted with DCM to reach 3 mL and finally cooled to -78 °C. Aminocyclopropane **1d** (1 mmol, 1.0 equiv.) and the indole derivative (1.05 mmol, 1.05 equiv.) were diluted together in DCM (1.4 mL) and added dropwise at -78 °C to the preformed catalyst. The reaction mixture was stirred at -78 °C for 30 minutes. The reaction was quenched by addition of 10 µL of Et_3N and the solvent was concentrated under reduced pressure. The crude product was then purified by flash chromatography.

A gram scale experiment with aminocyclopropane **1d** and 1-Methylindole was also accomplished using the same procedure and led to a similar yield and identical dr ratio.

Notice on the handling of bis(trifluoromethanesulfonyl)imide

Bis(trifluoromethanesulfonyl)imide was purchased from Acros and was stored in a glovebox. The solution in DCM (*ca.* 0.2 M, 2-3 mL) was prepared by diluting the acid (previously weighted in a glovebox) with dry DCM in oven-dried and nitrogen-purged vial. The solution was used directly to set up a reaction by taking off the required amount. Then the solution was stored in the fridge and renewed every two days. When the solution was stored for prolongated period, a yellow color appeared with solid residues (which may arise from the degradation of the septum in highly acidic media). Such yellow solutions did not promote the desired (3+2) annultion. Furthermore, due to the volatility of DCM, it is also recommended to perform a new solution every day. The stability of bis(trifluoromethanesulfonyl)imide solution can be enhanced in DCE. From our observations, Tf2NH is not totally soluble in toluene.

4.1. Scope of indoles

Ethyl-1-((*N*,4-dimethylphenyl)sulfonamido)-4-methyl-1,2,3,3a,4,8b-hexahydrocyclopenta[b]indole-3-carboxylate (4a)

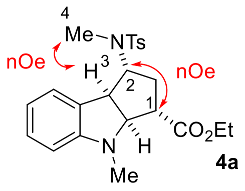

4a

Chemical Formula: $C_{23}H_{28}N_2O_4S$
Molecular Weight: 428.5470

Prepared according to the general procedure E from Tf_2NH (0.2 M, 39 µL, 7.8 µmol, 2.5 mol%), silyl enol ether **3b** (16.8 mg, 77.8 µmol, 0.25 equiv.), aminocyclopropane **1d** (92.6 mg, 311 µmol, 1.0 equiv.) and 1-methylindole **2a** (42 µL, 0.33 mmol, 1.05 equiv.) in DCM (3 mL). The crude product was purified by flash chromatography using pent/EtOAc 80:20 to afford cycloadduct **4a** as a colorless oil (127 mg, 296 µmol, 95%, dr 93:7).

For the gram scale experiment, cycloadduct **4a** (1.29 g, 3.02 mmol, 90%, dr 93:7) was obtained from Tf_2NH (0.2 M, 0.84 mL, 0.17 mmol, 5 mol%), silyl enol ether **3b** (0.18 g, 0.84 mmol, 0.25 equiv.), aminocyclopropane **1d** (1.00 g, 3.36 mmol, 1.0 equiv.) and 1-methylindole **2a** (0.44 mL, 3.5 mmol, 1.05 equiv.) in DCM (32 mL) using general procedure F.

Data for the major diastereomer

Rf = 0.3 (pentane/EtOAc 4:1).

^{1}H NMR (400 MHz, $CDCl_3$): δ 7.66 – 7.59 (m, 2H, Ts), 7.28 – 7.21 (m, 2H, Ts), 7.14 – 7.05 (m, 1H, Ar*H*), 6.89 (d, *J* = 7.0 Hz, 1H, Ar*H*), 6.57 (td, *J* = 7.4, 1.0 Hz, 1H, Ar*H*), 6.41 (d, *J* = 7.9 Hz, 1H, Ar*H*), 4.37 (ddd, *J* = 11.3, 8.4, 6.7 Hz, 1H, H-2, C*H*), 4.25 – 4.11 (m, 2H, OCH_2CH_3), 4.07 (dd, *J* = 10.1, 5.4 Hz, 1H, C*H*), 3.58 – 3.52 (m, 1H, H-3, C*H*), 2.91 (s, 3H, H-4, CH_3), 2.89 – 2.82 (m, 1H, H-1, C*H*), 2.80 (s, 3H, CH_3), 2.41 (s, 3H, CH_3), 1.95 – 1.76 (m, 2H, CH_2), 1.28 (t, *J* = 7.1 Hz, 3H, OCH_2CH_3).

^{13}C NMR (101 MHz, $CDCl_3$): δ 174.4, 150.9, 143.4, 136.6, 129.8, 129.1, 128.4, 127.2, 124.4, 118.2, 106.9, 72.9, 63.8, 61.2, 48.4, 48.0, 33.7, 31.5, 29.2, 21.6, 14.3.

IR (ν_{max}, cm^{-1}) 3057 (w), 2988 (w), 1725 (m), 1487 (m), 1340 (m), 1264 (m), 1161 (m).

HRMS (ESI/QTOF) m/z: $[M + H]^+$ Calcd for $C_{23}H_{29}N_2O_4S^+$ 429.1843; Found 429.1841.

Data for the minor diastereomer (procedure see above)

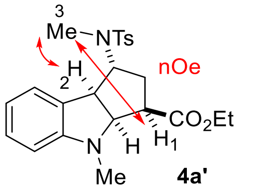

4a'

Chemical Formula: $C_{23}H_{28}N_2O_4S$
Molecular Weight: 428,54

Rf = 0.21 (pentane/EtOAc 4:1).

^{1}H NMR (400 MHz, $CDCl_3$): δ 7.72 – 7.65 (m, 2H, Ts), 7.31 – 7.26 (m, 2H, Ts), 7.15 – 7.08 (m, 2H, Ar*H*), 6.71 (t, *J* = 7.4 Hz, 1H, Ar*H*), 6.44 (d, *J* = 7.8 Hz, 1H, Ar*H*), 4.53 – 4.47 (m, 1H, C*H*), 4.26 – 4.19 (m, 1H, C*H*), 4.19 – 4.05 (m, 2H, OCH_2CH_3), 3.80 (dd, *J* = 9.3, 4.7 Hz, 1H, H-2, C*H*), 3.22 – 3.13 (m, 1H, H-1, C*H*) 2.84 (s, 3H, H-3, CH_3), 2.74 (s, 3H, CH_3), 2.42 (s, 3H, CH_3), 2.08 – 1.98 (m, 1H, CH_2), 1.61 – 1.51 (m, 1H, CH_2), 1.24 (t, *J* = 7.1 Hz, 3H, OCH_2CH_3).

^{13}C NMR (101 MHz, $CDCl_3$): δ 172.1, 152.8, 143.4, 136.3, 130.1, 129.9, 128.4, 127.4, 124.4, 118.8, 108.0, 73.2, 65.1, 60.8, 51.8, 49.3, 37.1, 30.2, 29.9, 21.7, 14.3.

HRMS (ESI/QTOF) m/z: $[M + H]^+$ Calcd for $C_{23}H_{29}N_2O_4S^+$ 429.1843; Found 429.1846.

Ethyl-4-(*tert*-butyldimethylsilyl)-1-((*N*,4-dimethylphenyl)sulfonamido)-1,2,3,3a,4,8b-hexahydrocyclopenta[b]indole-3-carboxylate (4b)

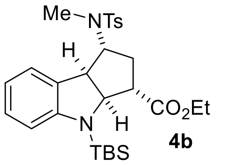

4b

Chemical Formula: $C_{28}H_{40}N_2O_4SSi$
Molecular Weight: 528.7830

Prepared according to the general procedure E from Tf_2NH (0.213 M, 72.5 µL, 15.4 µmol, 5 mol%), silyl enol ether **3b** (16.7 mg, 77.2 µmol, 0.25 equiv.), aminocyclopropane **1d** (91.8 mg, 309 µmol, 1.0 equiv.) and TBS-protected indole **2b** (75 mg, 0.32 mmol, 1.05 equiv.) in DCM (0.3 M, 1 mL). The crude product was purified by flash chromatography using pent/EtOAc 85:15 to afford cycloadduct **4b** as mixture of diastereoisomers and as a colorless oil (151 mg, 286 µmol, 92%, dr 91:9). From the general procedure F (scale up on 1 mmol), compound **4b** was obtained in 83% yield (0.84 mmol, 0.44 g, dr 90:10) from Tf_2NH (14.2 mg, 50.4 µmol, 5 mol%), silyl enol ether **3b** (54 mg, 0.25 mmol, 0.25 equiv.), aminocyclopropane **1d** (300 mg, 1.01 mmol, 1.0 equiv.) and TBS-protected indole **2b** (245 mg, 1.06 mmol, 1.05 equiv.) in DCM (0.3 M, 3.4 mL).

Rf = 0.48 (pentane/EtOAc 4:1).

^{1}H NMR (400 MHz, $CDCl_3$, signals for major diastereomer): δ 7.73 – 7.69 (m, 2H, Ts), 7.32 – 7.27 (m, 2H, Ts), 7.22 – 7.18 (m, 1H, Ar*H*), 7.04 – 6.99 (m, 1H, Ar*H*), 6.78 – 6.71 (m, 2H, Ar*H*), 4.62 (td, *J* = 8.6, 4.1 Hz, 1H, C*H*), 4.39 – 4.30 (m, 1H, C*H*), 4.10 (qd, *J* = 7.2, 2.9 Hz, 2H, OCH_2CH_3), 3.67 (dd, *J* = 9.5, 4.1 Hz, 1H, C*H*), 2.89 (s, 3H, CH_3), 2.54 (q, *J* = 9.5 Hz, 1H, C*H*), 2.42 (s, 3H, CH_3), 1.69 (t, *J* = 9.3 Hz, 2H, CH_2), 1.24 (t, *J* = 7.2 Hz, 3H, OCH_2CH_3), 0.82 (s, 9H, TBS), 0.38 (s, 3H, TBS), 0.09 (s, 3H, TBS).

^{13}C NMR (101 MHz, $CDCl_3$, signals for major diastereomer): 174.9, 150.1, 143.5, 136.6, 133.1, 129.9, 127.7, 127.3, 124.6, 119.5, 112.9, 69.6, 63.3, 60.9, 51.5, 50.7, 31.1, 29.7, 27.3, 21.7, 20.5, 14.2, -2.7, -4.5.

IR (ν_{max}, cm^{-1}) 2946 (m), 2856 (m), 1725 (s), 1468 (s), 1337 (s), 1254 (s), 1160 (s), 735 (s).

HRMS (ESI/QTOF) m/z: $[M + H]^+$ Calcd for $C_{28}H_{41}N_2O_4SSi^+$ 529.2551; Found 529.2558.

Ethyl-4-benzyl-1-((*N*,4-dimethylphenyl)sulfonamido)-1,2,3,3a,4,8b-hexahydrocyclopenta[b]indole-3-carboxylate (4c)

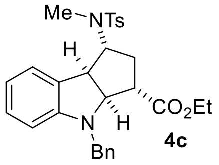

4c

Chemical Formula: $C_{29}H_{32}N_2O_4S$
Molecular Weight: 504.6450

Prepared according to the general procedure E from Tf_2NH (0.216 M, 74 µL, 16 µmol, 5 mol%), silyl enol ether **3b** (17.3 mg, 79.9 µmol, 0.25 equiv.), aminocyclopropane **1d** (95 mg, 0.32 mmol, 1.0 equiv.) and benzyl-protected indole **2c** (69.5 mg, 335 µmol, 1.05 equiv.) in DCM (0.1 M, 3 mL). The crude product was purified by flash chromatography using pent/EtOAc 85:15 to afford cycloadduct **4c** as a white solid and mixture of diastereomers (125 mg, 248 µmol, 78%, dr 92:8). From the general procedure F (scale up on 1 mmol), compound **4c** was obtained in 83% yield (0.42 g, 0.83 mmol, dr 91:9) from Tf_2NH (14 mg, 50 µmol, 5 mol%), silyl enol ether **3b** (54.2 mg, 251 µmol, 0.25 equiv.), aminocyclopropane **1d** (298 mg, 1.00 mmol, 1.0 equiv.) and benzyl-protected indole **2c** (218 mg, 1.05 µmol, 1.05 equiv.) in DCM (0.3 M, 3.3 mL).

Rf = 0.67 (pentane/EtOAc 4:1).

m. p. = 45.5 – 49.2 °C.

^{1}H NMR (400 MHz, $CDCl_3$, signals for major diastereomer): δ 7.67 – 7.63 (m, 2H, Ts), 7.32 – 7.20 (m, 7H, Ts, Ar*H*), 7.04 (t, *J* = 7.7 Hz, 1H, Ar*H*), 6.95 (d, *J* = 7.3 Hz, 1H, Ar*H*), 6.58 (t, *J* = 7.4 Hz, 1H, Ar*H*), 6.39 (d, *J* = 7.9 Hz, 1H, Ar*H*), 4.47 – 4.36 (m, 3H, CH_2, C*H*), 4.35 – 4.28 (m, 1H, C*H*), 4.02 (qd, *J* = 7.1, 1.7 Hz, 2H, OCH_2CH_3), 3.68 – 3.61 (m, 1H, C*H*), 2.89 (s, 3H, CH_3), 2.85 – 2.77 (m, 1H, C*H*), 2.42 (s, 3H, CH_3), 1.86 – 1.72 (m, 2H, CH_2), 1.15 (t, *J* = 7.1 Hz, 3H, OCH_2CH_3).

^{13}C NMR (101 MHz, $CDCl_3$, signals for major diastereomer): δ 174.1, 150.6, 143.4, 138.4, 136.5, 129.8, 128.8, 128.6, 128.4, 127.5, 127.2, 127.2, 124.5, 117.9, 106.9, 70.3, 64.2, 61.0, 51.3, 49.6, 48.0, 31.5, 29.1, 21.6, 14.1.
IR (ν_{max}, cm^{-1}) 2971 (m), 2867 (m), 1726 (s), 1486 (s), 1341 (s), 1159 (s), 738 (s).
HRMS (ESI/QTOF) m/z: $[M + H]^+$ Calcd for $C_{29}H_{33}N_2O_4S^+$ 505.2156; Found 505.2161.

Ethyl-1-((*N*,4-dimethylphenyl)sulfonamido)-4-(4-methoxybenzyl)-1,2,3,3a,4,8b-hexahydrocyclopenta[b]indole-3-carboxylate (4d)

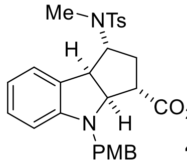

4d
Chemical Formula: $C_{30}H_{34}N_2O_5S$
Molecular Weight: 534.6710

Prepared according to the general procedure E from Tf_2NH (0.192 M, 13.3 μL, 2.6 μmol, 2.5 mol%), silyl enol ether **3b** (5.5 mg, 26 μmol, 0.25 equiv.), aminocyclopropane **1d** (30.4 mg, 102 μmol, 1.0 equiv.) and PMB-protected indole **2d** (25.5 mg, 107 μmol, 1.05 equiv.) in DCM (0.1 M, 1 mL). The crude product was purified by PREP TLC using pent/EtOAc 80:20 to afford cycloadduct **4d** as a colorless oil and mixture of diastereomers (44 mg, 82 μmol, 81%, dr 92:8). From the general procedure F (scale up on 1 mmol), compound **4d** was obtained in 85% yield (0.46 g, 0.87 mmol, dr 89:11) from Tf_2NH (0.23 M, 0.22 mL, 51 μmol, 5 mol%), silyl enol ether **3b** (55 mg, 0.25 mmol, 0.25 equiv.), aminocyclopropane **1d** (302 mg, 1.02 mmol, 1.0 equiv.) and PMB-protected indole **2d** (253 mg, 1.07 mmol, 1.05 equiv.) in DCM (0.3 M, 3.4 mL).
Rf = 0.31 (pentane/EtOAc 4:1).
^{1}H NMR (400 MHz, $CDCl_3$, signals for major diastereomer): δ 7.68 – 7.61 (m, 2H, Ts), 7.29 – 7.23 (m, 2H, Ts), 7.20 – 7.14 (m, 2H, Ar*H*), 7.05 (td, *J* = 7.7, 1.3 Hz, 1H, Ar*H*), 6.93 (d, *J* = 7.4 Hz, 1H, Ar*H*), 6.86 – 6.79 (m, 2H, Ar*H*), 6.57 (t, *J* = 7.4 Hz, 1H, Ar*H*), 6.42 (d, *J* = 7.9 Hz, 1H, Ar*H*), 4.43 – 4.24 (m, 4H, CH_2, 2xC*H*), 4.04 (qd, *J* = 7.2, 2.7 Hz, 2H, OCH_2CH_3), 3.78 (s, 3H, OCH_3), 3.65 – 3.59 (m, 1H, C*H*), 2.89 (s, 3H, CH_3), 2.83 – 2.76 (m, 1H, C*H*), 2.42 (s, 3H, CH_3), 1.82 – 1.73 (m, 2H, CH_2), 1.17 (t, *J* = 7.1 Hz, 3H, OCH_2CH_3).
^{13}C NMR (101 MHz, $CDCl_3$, signals for major diastereomer): δ 174.3, 158.9, 150.6, 143.4, 136.6, 130.3, 129.8, 128.9, 128.9, 128.4, 127.3, 124.5, 117.9, 114.0, 107.0, 70.2, 64.3, 61.1, 55.4, 50.7, 49.6, 48.0, 31.7, 29.1, 21.6, 14.2.
IR (ν_{max}, cm^{-1}) 3008 (w), 2905 (w), 1725 (m), 1340 (m), 1247 (m), 1162 (m), 910 (s).
HRMS (ESI/QTOF) m/z: $[M + H]^+$ Calcd for $C_{30}H_{35}N_2O_5S^+$ 535.2261; Found 535.2267.

Ethyl-4-(2-((tert-butyldimethylsilyl)oxy)ethyl)-1-((*N*,4-dimethylphenyl)sulfonamido)-1,2,3,3a,4,8b-hexahydrocyclopenta[b]indole-3-carboxylate (4e)

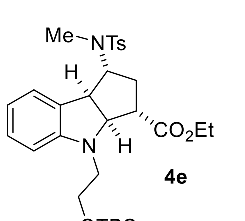

4e
Chemical Formula: $C_{30}H_{44}N_2O_5SSi$
Molecular Weight: 572.8360

Prepared according to the general procedure E from Tf_2NH (0.213 M, 70.8 μL, 15.1 μmol, 5 mol%), silyl enol ether **3b** (16.3 mg, 75.4 μmol, 0.25 equiv.), aminocyclopropane **1d** (89.7 mg, 302 μmol, 1.0 equiv.) and indole **2e** (87.2 mg, 317 μmol, 1.05 equiv.) in DCM (0.3 M, 1 mL). The crude product was purified by flash chromatography using pent/EtOAc 90:10 to afford cycloadduct **4e** as a colorless oil and mixture of diastereomers (135 mg, 236 μmol, 78%, dr 91:9).
Rf = 0.66 (pentane/EtOAc 4:1).
^{1}H NMR (400 MHz, $CDCl_3$, signals for major diastereomer): δ 7.65 – 7.60 (m, 2H, Ts), 7.27 – 7.22 (m, 2H, Ts), 7.05 (td, *J* = 7.7, 1.3 Hz, 1H, Ar*H*), 6.87 (d, *J* = 7.3 Hz, 1H, Ar*H*), 6.52 (td, *J*

= 7.4, 1.0 Hz, 1H, Ar*H*), 6.41 (d, *J* = 7.9 Hz, 1H, Ar*H*), 4.36 – 4.28 (m, 2H, 2xC*H*), 4.22 – 4.12 (m, 2H, OC*H*$_2$CH$_3$), 3.78 – 3.68 (m, 2H, OC*H*$_2$), 3.60 – 3.53 (m, 1H, C*H*), 3.32 (t, *J* = 6.4 Hz, 2H, C*H*$_2$), 2.90 (s, 3H, C*H*$_3$), 2.88 – 2.84 (m, 1H, C*H*), 2.42 (s, 3H, C*H*$_3$), 1.89 – 1.72 (m, 2H, C*H*$_2$), 1.28 (t, *J* = 7.1 Hz, 3H, OCH$_2$C*H*$_3$), 0.87 (s, 9H, *t*Bu), 0.02 (s, 3H, C*H*$_3$), 0.02 (s, 3H, C*H*$_3$).

^{13}C NMR (101 MHz, CDCl$_3$, signals for major diastereomer): δ 174.5, 150.3, 143.4, 136.6, 129.8, 128.7, 128.3, 127.3, 124.4, 117.5, 106.3, 71.1, 64.1, 61.1, 60.8, 49.7, 49.6, 48.0, 31.3, 29.1, 26.0, 21.6, 18.4, 14.3, -5.25, -5.27.

IR (ν_{max}, cm^{-1}) 3042 (w), 2939 (m), 1728 (s), 1342 (s), 1254 (s), 1162 (s), 1096 (s), 740 (s).

HRMS (ESI/QTOF) m/z: [M + H]$^+$ Calcd for $C_{30}H_{45}N_2O_5SSi^+$ 573.2813; Found 573.2825.

Ethyl-1-((*N*,4-dimethylphenyl)sulfonamido)-3a,4-dimethyl-1,2,3,3a,4,8b-hexahydrocyclopenta[b]indole-3-carboxylate (4f)

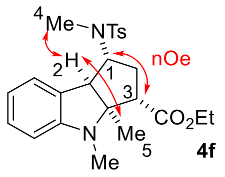

Chemical Formula: $C_{24}H_{30}N_2O_4S$
Molecular Weight: 442.5740

Prepared according to the general procedure E from Tf$_2$NH (0.213 M, 36 µL, 7.7 µmol, 2.5 mol%), silyl enol ether **3b** (16.6 mg, 76.6 µmol, 0.25 equiv.), aminocyclopropane **1d** (91.1 mg, 306 µmol, 1.0 equiv.) and indole **2f** (46.7 mg, 322 µmol, 1.05 equiv.) in DCM (0.3 M, 1 mL). The crude product was purified by flash chromatography using pent/EtOAc 80:20 to afford cycloadduct **4f** as a colorless oil and mixture of diastereoisomers (127 mg, 287 µmol, 94%, dr 75:25). Some of the mixture was purified further by PREP TLC using pent/EtOAc 80:20 allowing the isolation and clean NMR characterization of each diastereomer.

Data for the major diastereomer

Rf = 0.42 (pentane/EtOAc 4:1).

^{1}H NMR (400 MHz, CDCl$_3$): δ 7.63 – 7.59 (m, 2H, Ts), 7.25 – 7.20 (m, 2H, Ts), 7.10 (td, *J* = 7.7, 1.3 Hz, 1H, Ar*H*), 6.95 – 6.90 (m, 1H, Ar*H*), 6.53 (td, *J* = 7.4, 1.0 Hz, 1H, Ar*H*), 6.31 (d, *J* = 7.8 Hz, 1H, Ar*H*), 4.31 – 4.11 (m, 3H, H-1, C*H*, OC*H*$_2$CH$_3$), 3.07 (d, *J* = 8.8 Hz, 1H, H-2, C*H*), 2.98 – 2.91 (m, 4H, H-3, H-C*H*, C*H*$_3$), 2.82 (s, 3H, C*H*$_3$), 2.41 (s, 3H, C*H*$_3$), 2.05 (q, *J* = 12.4 Hz, 1H, C*H*$_2$), 1.63 – 1.54 (m, 1H, C*H*$_2$), 1.30 (t, *J* = 7.1 Hz, 3H, OCH$_2$C*H*$_3$), 1.16 (s, 3H, C*H*$_3$).

^{13}C NMR (101 MHz, CDCl$_3$): δ 172.9, 149.7, 143.3, 136.6, 129.8, 128.5, 127.7, 127.2, 124.5, 117.2, 105.6, 74.9, 62.6, 60.9, 56.0, 52.1, 30.0, 29.1, 28.5, 21.6, 19.3, 14.4.

IR (ν_{max}, cm^{-1}) 3043 (w), 1728 (m), 1487 (m), 1339 (m), 1154 (s), 910 (s), 730 (s).

HRMS (ESI/QTOF) m/z: [M + H]$^+$ Calcd for $C_{24}H_{31}N_2O_4S^+$ 443.1999; Found 443.2002.

Data for the minor diastereomer

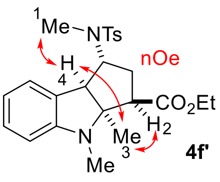

Rf = 0.40 (pentane/EtOAc 4:1).

^{1}H NMR (400 MHz, CDCl$_3$): δ 7.71 – 7.65 (m, 2H, Ts), 7.30 – 7.24 (m, 2H, Ts), 7.11 – 7.03 (m, 2H, Ar*H*), 6.62 (td, *J* = 7.4, 1.0 Hz, 1H, Ar*H*), 6.29 (d, *J* = 7.8 Hz, 1H, Ar*H*), 4.55 (dt, *J* = 7.5, 6.0 Hz, 1H, C*H*), 4.07 (qd, *J* = 7.2, 2.1 Hz, 2H, OC*H*$_2$CH$_3$), 3.34 (d, *J* = 5.5 Hz, H-4, 1H, C*H*), 2.91 – 2.82 (m, 4H, H-1, H-2, C*H*, C*H*$_3$), 2.64 (s, 3H, C*H*$_3$), 2.41 (s, 3H, C*H*$_3$), 1.94 (dt, *J* = 13.9, 7.6 Hz, 1H, C*H*$_2$), 1.66 – 1.54 (m, 2H, C*H*$_2$), 1.44 (s, 3H, H-3, C*H*$_3$), 1.19 (t, *J* = 7.2 Hz, 3H, OCH$_2$C*H*$_3$).

^{13}C NMR (101 MHz, CDCl$_3$): δ 172.3, 151.4, 143.4, 136.4, 129.8, 128.9, 128.4, 127.4, 124.4, 117.8, 106.3, 65.1, 60.8, 59.3, 54.4, 30.4, 30.2, 30.1, 29.8, 23.4, 21.7, 14.2.

HRMS (ESI/QTOF) m/z: [M + H]$^+$ Calcd for $C_{24}H_{31}N_2O_4S^+$ 443.1999; Found 443.1998.

Ethyl-1-((*N*,4-dimethylphenyl)sulfonamido)-4,8b-dimethyl-1,2,3,3a,4,8b-hexahydrocyclopenta[b]indole-3-carboxylate (4g)

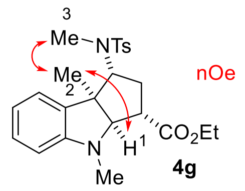

Chemical Formula: $C_{24}H_{30}N_2O_4S$
Molecular Weight: 442.5740

Prepared according to the general procedure E from Tf_2NH (0.222 M, 10.6 µL, 2.4 µmol, 2.5 mol%), silyl enol ether **3b** (5.1 mg, 24 µmol, 0.25 equiv.), aminocyclopropane **1d** (27.9 mg, 93.8 µmol, 1.0 equiv.) and indole **2g** (14.3 mg, 98.5 µmol, 1.05 equiv.) in DCM (0.1 M, 1 mL). The crude product was purified by PREP TLC using pent/EtOAc 80:20 to afford cycloadduct **4g** as a colorless oil and mixture of diastereomers (35 mg, 80 µmol, 85%, dr 92:8).

Rf = 0.5 (pentane/EtOAc 4:1).

^{1}H NMR (400 MHz, $CDCl_3$, signals for major diastereomer): δ 7.64 (d, *J* = 8.3 Hz, 2H, Ts), 7.32 (dd, *J* = 7.4, 1.3 Hz, 1H, Ar*H*), 7.30 – 7.24 (m, 2H, Ts), 7.14 (td, *J* = 7.7, 1.3 Hz, 1H, Ar*H*), 6.74 (td, *J* = 7.4, 1.0 Hz, 1H, Ar*H*), 6.43 (d, *J* = 7.8 Hz, 1H, Ar*H*), 4.47 (dd, *J* = 10.6, 7.2 Hz, 1H, C*H*), 4.23 – 4.08 (m, 2H, OCH_2CH_3), 3.71 (d, *J* = 5.7 Hz, 1H, H-1, C*H*), 2.86 (s, 3H, H-3 CH_3), 2.81 (s, 3H, CH_3), 2.71 (ddd, *J* = 10.5, 8.3, 5.7 Hz, 1H, C*H*), 2.41 (s, 3H, CH_3), 1.97 (dt, *J* = 13.1, 10.5 Hz, 1H, CH_2), 1.64 (dt, *J* = 13.1, 7.8 Hz, 1H, CH_2), 1.34 (s, 3H, H-2, CH_3), 1.26 (t, *J* = 7.1 Hz, 3H, OCH_2CH_3).

^{13}C NMR (101 MHz, $CDCl_3$, signals for major diastereomer): δ 174.7, 149.1, 143.4, 135.8, 135.5, 129.8, 128.3, 127.2, 123.8, 118.2, 106.8, 80.2, 64.3, 61.1, 55.4, 46.5, 33.1, 32.5, 29.8, 21.6, 21.4, 14.3.

IR (ν_{max}, cm^{-1}) 3046 (m), 1729 (m), 1602 (m), 1484 (m), 1340 (s), 1160 (s), 733 (s).

HRMS (ESI/QTOF) m/z: $[M + H]^+$ Calcd for $C_{24}H_{31}N_2O_4S^+$ 443.1999; Found 443.2008.

Ethyl-8b-(2-((tert-butyldimethylsilyl)oxy)ethyl)-1-((*N*,4-dimethylphenyl)sulfonamido)-4-methyl-1,2,3,3a,4,8b-hexahydrocyclopenta[b]indole-3-carboxylate (4h)

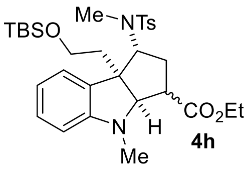

Chemical Formula: $C_{31}H_{46}N_2O_5SSi$
Molecular Weight: 586.8630

Prepared according to the general procedure E from Tf_2NH (0.229 M, 66 µL, 15 µmol, 5 mol%), silyl enol ether **3b** (16.3 mg, 75.4 µmol, 0.25 equiv.), aminocyclopropane **1d** (89.7 mg, 302 µmol, 1.0 equiv.) and indole **2h** (91.7 mg, 317 µmol, 1.05 equiv.) in DCM (0.3 M, 1 mL). The crude product was purified by flash chromatography using pent/EtOAc 80:20 to afford cycloadduct **4h** as a colorless oil and inseparable mixture of diastereoisomers (150 mg, 256 µmol, 84%, dr 71:29).

Characterized as mixture of diastereomers

Rf = 0.8 (pentane/EtOAc 4:1).

^{1}H NMR (400 MHz, $CDCl_3$, signals for major diastereomer): δ 7.65 – 7.59 (m, 2H, Ts), 7.31 – 7.22 (m, 3H, Ts, Ar*H*), 7.16 – 7.08 (m, 1H, Ar*H*), 6.76 – 6.70 (m, 1H, Ar*H*), 6.40 – 6.35 (m, 1H, Ar*H*), 4.45 (dd, *J* = 10.3, 7.1 Hz, 1H, C*H*), 4.21 – 4.08 (m, 2H, OCH_2CH_3), 4.02 (d, *J* = 5.9 Hz, 1H, C*H*), 3.50 – 3.33 (m, 2H, OCH_2), 2.84 (s, 3H, CH_3), 2.79 (s, 3H, CH_3), 2.66 (ddd, *J* = 10.3, 8.3, 5.9 Hz, 1H, C*H*), 2.40 (s, 3H, CH_3), 2.22 – 2.11 (m, 1H, CH_2), 1.95 – 1.77 (m, 2H, CH_2), 1.61 – 1.49 (m, 1H, CH_2), 1.25 (t, *J* = 7.1 Hz, 3H, OCH_2CH_3), 0.82 (s, 9H, TBS), -0.05 (s, 3H, TBS), -0.08 (s, 3H, TBS).

^{1}H NMR (400 MHz, $CDCl_3$, selected signals for minor diastereomer): δ 4.58 (dd, *J* = 8.3, 1.7 Hz, 1H, C*H*), 4.26 – 4.22 (m, 1H, C*H*), 3.11 (dt, *J* = 12.7, 7.0 Hz, 1H, C*H*), 2.80 (s, 3H, CH_3), 2.75 (s, 3H, CH_3), 2.39 (s, 3H, CH_3), 2.30 (ddd, *J* = 14.1, 7.1, 5.0 Hz, 1H, CH_2), 2.07 – 1.97 (m, 1H, CH_2), 1.73 (dt, *J* = 14.5, 7.4 Hz, 1H, CH_2), 1.39 – 1.31 (m, 1H, CH_2), 0.83 (s, 9H, TBS), -0.04 (s, 3H, TBS), -0.06 (s, 3H, TBS).

^{13}C NMR (101 MHz, $CDCl_3$, signal for both diastereomers): δ 174.6, 171.8, 152.5, 149.8, 143.5, 143.4, 136.3, 135.6, 133.4, 132.1, 129.9, 129.8, 128.5, 127.3 (2C), 127.0, 124.4, 123.7,

118.7, 117.9, 107.7, 106.4, 77.1, 76.5, 67.1, 65.6, 61.1, 60.8, 60.69, 60.65, 60.1, 57.7, 50.0, 47.3, 38.0, 37.3, 36.5, 33.0, 32.9, 31.8, 29.5, 28.5, 26.13, 26.07, 21.64, 21.62, 18.4, 18.3, 14.3, -5.1, -5.2 (2C), -5.3.
IR (ν_{max}, cm^{-1}) 3053 (w), 2940 (w), 1727 (m), 1482 (m), 1343 (m), 1162 (s), 1092 (m), 735 (s).
HRMS (ESI/QTOF) m/z: $[M + H]^+$ Calcd for $C_{31}H_{47}N_2O_5SSi^+$ 587.2969; Found 587.2980.

Ethyl-8b-(2-azidoethyl)-1-((*N*,4-dimethylphenyl)sulfonamido)-4-methyl-1,2,3,3a,4,8b-hexahydrocyclopenta[b]indole-3-carboxylate (4i)

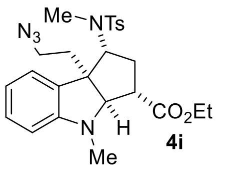

4i
Chemical Formula: $C_{25}H_{31}N_5O_4S$
Molecular Weight: 497.6140

Prepared according to the general procedure E from Tf_2NH (0.229 M, 133 µL, 30.4 µmol, 20 mol%), silyl enol ether **3b** (16.5 mg, 76.1 µmol, 0.5 equiv.), aminocyclopropane **1d** (45.3 mg, 152 µmol, 1.0 equiv.) and indole **2i** (32 mg, 0.16 mmol, 1.05 equiv.) in DCM (0.15 M, 1 mL). The crude product was purified by flash chromatography using pent/EtOAc 80:20 to afford cycloadduct **4i** as a colorless oil and mixture of diastereomers (61 mg, 0.12 mmol, 81%, dr 80:20). Half of the mixture was purified further by PREP TLC using pent/EtOAc 80:20 allowing the isolation and clean NMR characterization of the major diastereomer. The minor isomer was not obtained as a pure fraction but a ^1H NMR is still provided.

Data for the major diastereomer

Rf = 0.59 (pentane/EtOAc 4:1).
^{1}H NMR (400 MHz, $CDCl_3$): δ 7.67 – 7.62 (m, 2H, Ts), 7.36 (dd, *J* = 7.3, 1.2 Hz, 1H, Ar*H*), 7.31 – 7.27 (m, 2H, Ts), 7.16 (td, *J* = 7.8, 1.3 Hz, 1H, Ar*H*), 6.77 (td, *J* = 7.5, 1.0 Hz, 1H, Ar*H*), 6.41 (d, *J* = 7.8 Hz, 1H, Ar*H*), 4.59 (dd, *J* = 9.1, 7.6 Hz, 1H, C*H*), 4.22 – 4.07 (m, 2H, OCH_2CH_3), 3.95 (d, *J* = 6.4 Hz, 1H, C*H*), 3.13 – 3.07 (m, 2H, CH_2), 2.82 (s, 3H, CH_3), 2.80 (s, 3H, CH_3), 2.64 (ddd, *J* = 9.9, 8.6, 6.4 Hz, 1H, C*H*), 2.41 (s, 3H, CH_3), 2.23 – 2.15 (m, 1H, CH_2), 1.86 – 1.77 (m, 2H, CH_2), 1.68 – 1.59 (m, 1H, CH_2), 1.24 (t, *J* = 7.1 Hz, 3H, OCH_2CH_3).
^{13}C NMR (101 MHz, $CDCl_3$): δ 174.6, 149.5, 143.7, 135.5, 131.7, 129.9, 129.0, 127.3, 124.4, 118.4, 106.8, 77.4, 65.4, 61.3, 57.7, 47.9, 46.8, 33.6, 32.8, 32.6, 30.0, 21.7, 14.3.
IR (ν_{max}, cm^{-1}) 3045 (w), 2978 (m), 2095 (s), 1727 (s), 1484 (m), 1341 (s), 1165 (s), 741 (s).
HRMS (ESI/QTOF) m/z: $[M + H]^+$ Calcd for $C_{25}H_{32}N_5O_4S^+$ 498.2170; Found 498.2174.

^{1}H NMR for the minor diastereomer

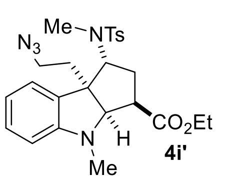

4i'
Chemical Formula: $C_{25}H_{31}N_5O_4S$
Molecular Weight: 497.6140

^{1}H NMR (400 MHz, $CDCl_3$, signals for the minor isomer) δ 7.67 – 7.61 (m, 2H, Ts), 7.30 – 7.26 (m, 2H, Ts), 7.21 (dd, *J* = 7.4, 1.3 Hz, 1H, Ar*H*), 7.13 (td, *J* = 7.7, 1.3 Hz, 1H, Ar*H*), 6.77 (td, *J* = 7.4, 1.0 Hz, 1H, Ar*H*), 6.39 (d, *J* = 7.9 Hz, 1H, Ar*H*), 4.65 (dd, *J* = 8.1, 1.6 Hz, 1H, C*H*), 4.22 – 3.99 (m, 3H, C*H*, OCH_2CH_3), 3.18 – 3.08 (m, 2H, CH_2), 2.95 – 2.85 (m, 1H, C*H*), 2.80 (s, 6H, 2xCH_3), 2.44 – 2.32 (m, 4H, CH_3, CH_2), 2.10 – 1.98 (m, 1H, CH_2), 1.71 (dt, *J* = 14.2, 7.9 Hz, 1H, CH_2), 1.41 – 1.31 (m, 1H, CH_2), 1.25 (t, *J* = 7.1 Hz, 3H, OCH_2CH_3).

Ethyl-1-((*N*,4-dimethylphenyl)sulfonamido)-3a,4,8b-trimethyl-1,2,3,3a,4,8b-hexahydrocyclopenta[b]indole-3-carboxylate (4j)

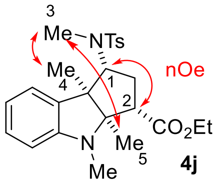

Chemical Formula: $C_{25}H_{32}N_2O_4S$
Molecular Weight: 456.6010

Prepared according to the general procedure E from Tf_2NH (0.211 M, 12.7 µL, 2.68 µmol, 2.5 mol%), silyl enol ether **3b** (5.8 mg, 27 µmol, 0.25 equiv.), aminocyclopropane **1d** (31.8 mg, 107 µmol, 1.0 equiv.) and indole **2j** (17.9 mg, 112 µmol, 1.05 equiv.) in DCM (0.1 M, 1 mL). The crude product was purified by flash chromatography using pent/EtOAc 85:15 to afford cycloadduct **4j** as as a colorless oil and mixture of diastereoisomers (46 mg, 0.10 mmol, 94%, dr 70:30). Half of the mixture was purified further by PREP TLC using pent/EtOAc 80:20 allowing the isolation and clean NMR characterization of each diastereomer.

Data for the major diastereomer

Rf = 0.45 (pentane/EtOAc 4:1).

^{1}H NMR (400 MHz, $CDCl_3$): δ 7.67 – 7.60 (m, 2H, Ts), 7.43 – 7.36 (m, 1H, Ar*H*), 7.29 – 7.22 (m, 2H, Ts), 7.14 (td, *J* = 7.7, 1.3 Hz, 1H, Ar*H*), 6.75 (td, *J* = 7.4, 1.0 Hz, 1H, Ar*H*), 6.37 (dd, *J* = 7.8, 1.0 Hz, 1H, Ar*H*), 4.55 (dd, *J* = 11.7, 7.9 Hz, H-1, 1H, C*H*), 4.22 – 4.07 (m, 2H, OCH_2CH_3), 2.91 – 2.83 (m, 4H, H-2, H-3, C*H*, CH_3), 2.77 (s, 3H, CH_3), 2.40 (s, 3H, CH_3), 2.09 (dt, *J* = 13.1, 11.9 Hz, 1H, CH_2), 1.47 (dt, *J* = 13.1, 7.6 Hz, 1H, CH_2), 1.26 (t, *J* = 7.1 Hz, 3H, OCH_2CH_3), 1.19 (s, 3H, H-4/5, CH_3), 1.12 (s, 3H, H-4/5, CH_3).

^{13}C NMR (101 MHz, $CDCl_3$): δ 173.8, 147.7, 143.3, 136.01, 135.98, 129.8, 128.3, 127.2, 123.9, 118.1, 106.3, 78.2, 63.6, 60.8, 58.2, 49.3, 32.7, 29.1, 28.6, 21.6, 17.2, 14.31, 14.29.

IR (ν_{max}, cm^{-1}) 3038 (w), 1727 (m), 1489 (m), 1339 (s), 1158 (s), 734 (s).

HRMS (ESI/QTOF) m/z: $[M + H]^+$ Calcd for $C_{25}H_{33}N_2O_4S^+$ 457.2156; Found 457.2161.

Data for the minor diastereomer

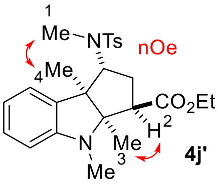

Rf = 0.42 (pentane/EtOAc 4:1).

^{1}H NMR (400 MHz, $CDCl_3$): δ 7.65 – 7.58 (m, 2H, Ts), 7.29 – 7.21 (m, 3H, Ts, Ar*H*), 7.09 (td, *J* = 7.6, 1.3 Hz, 1H, Ar*H*), 6.71 (td, *J* = 7.4, 1.0 Hz, 1H, Ar*H*), 6.29 (m, 1H, Ar*H*), 4.52 (dd, *J* = 8.9, 3.1 Hz, 1H, C*H*), 4.09 (qd, *J* = 7.2, 2.9 Hz, 2H, OCH_2CH_3), 2.80 (s, 3H, H-1, CH_3), 2.78 – 2.70 (m, 1H, H-2, C*H*), 2.61 (s, 3H, CH_3), 2.39 (s, 3H, CH_3), 2.10 (m, 1H, CH_2), 1.44 (ddd, *J* = 14.4, 6.8, 3.0 Hz, 1H, CH_2), 1.37 (s, 3H, H-3, CH_3), 1.35 (s, 3H, H-4, CH_3), 1.26 (t, *J* = 7.2 Hz, 3H, OCH_2CH_3).

^{13}C NMR (101 MHz, $CDCl_3$): δ 172.3, 150.1, 143.3, 136.4, 136.0, 129.8, 128.3, 127.1, 123.5, 117.9, 105.9, 79.5, 66.8, 60.9, 60.6, 54.1, 31.9, 30.5, 28.3, 21.6, 19.2, 18.5, 14.3.

HRMS (ESI/QTOF) m/z: $[M + H]^+$ Calcd for $C_{25}H_{33}N_2O_4S^+$ 457.2156; Found 457.2163.

Ethyl-12-((*N*,4-dimethylphenyl)sulfonamido)-9-methyl-1,2,3,4-tetrahydro-9H-4a,9a-propanocarbazole-10-carboxylate (4k)

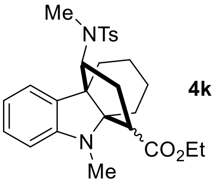

Chemical Formula: $C_{27}H_{34}N_2O_4S$
Molecular Weight: 482.6390

Prepared according to the general procedure E from Tf_2NH (0.23M, 45.3 µL, 10.4 µmol, 10 mol%), silyl enol ether **3b** (9.0 mg, 42 µmol, 0.4 equiv.), aminocyclopropane **1d** (31 mg, 0.10 mmol, 1 equiv.) and indole **2k** (20.3 mg, 109 µmol, 1.05 equiv.) in DCM (0.1 M, 1 mL). The crude product was purified by flash chromatography using pent/EtOAc 85:15 to afford cycloadduct **4k** as a mixture of diastereoisomers and as a white solid (43.7 mg, 90.5 µmol, 87%, dr 58:42). Half of the mixture was purified further by PREP TLC using pent/EtOAc 80:20 allowing the isolation and clean NMR characterization of each diastereomer.

NMR data for major diastereomer

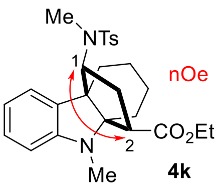

4k

Rf = 0.35 (pentane/EtOAc 4:1).
m. p. = 50.9 – 54.1 °C.
^{1}H NMR (400 MHz, $CDCl_3$): δ 7.62 – 7.57 (m, 2H, Ts), 7.30 (dd, *J* = 7.4, 1.2 Hz, 1H, Ar*H*), 7.25 – 7.21 (m, 2H, Ts), 7.14 (td, *J* = 7.6, 1.2 Hz, 1H, Ar*H*), 6.71 (td, *J* = 7.4, 1.0 Hz, 1H, Ar*H*), 6.35 (dd, *J* = 7.8, 1.0 Hz, 1H, Ar*H*), 4.43 (dd, *J* = 12.4, 7.1 Hz, 1H, H-1, C*H*), 4.25 – 4.10 (m, 2H, OCH_2CH_3), 2.96 (dd, *J* = 11.5, 7.4 Hz, H-2, 1H, C*H*), 2.91 (s, 3H, CH_3), 2.79 (s, 3H, CH_3), 2.39 (s, 3H, CH_3), 2.27 (td, *J* = 12.7, 11.5 Hz, 1H, CH_2), 1.92 – 1.76 (m, 2H, CH_2), 1.66 (ddd, *J* = 14.1, 8.2, 5.6 Hz, 1H, CH_2), 1.56 – 1.49 (m, 1H, CH_2), 1.49 – 1.39 (m, 2H, CH_2), 1.29 (t, *J* = 7.1 Hz, 3H, OCH_2CH_3), 1.28 – 1.18 (m, 2H, CH_2), 1.17 – 1.05 (m, 1H, CH_2).
^{13}C NMR (101 MHz, $CDCl_3$): δ 173.3, 149.0, 143.3, 135.6, 133.0, 129.7, 128.3, 127.4, 123.8, 117.4, 105.8, 77.3, 64.1, 61.0, 58.0, 50.2, 33.4, 28.5, 27.9, 25.9, 24.5, 21.6, 19.1, 18.6, 14.4.
IR (ν_{max}, cm^{-1}) 3053 (w), 2941 (m), 1725 (s), 1487 (m), 1341 (m), 1157 (s), 740 (s).
HRMS (ESI/QTOF) m/z: $[M + H]^+$ Calcd for $C_{27}H_{35}N_2O_4S^+$ 483.2312; Found 483.2314.

NMR data for minor diastereomer[1]

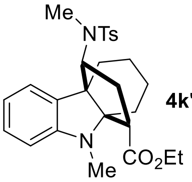

4k'

Rf = 0.32 (pentane/EtOAc 4:1).
^{1}H NMR (400 MHz, $CDCl_3$): δ 7.61 (d, *J* = 8.3 Hz, 2H, Ts), 7.27 – 7.20 (m, 3H, Ts, Ar*H*), 7.11 (td, *J* = 7.6, 1.3 Hz, 1H, Ar*H*), 6.71 (td, *J* = 7.4, 1.0 Hz, 1H, Ar*H*), 6.29 (d, *J* = 7.9 Hz, 1H, Ar*H*), 4.54 (dd, *J* = 8.8, 4.8 Hz, 1H, C*H*), 4.22 – 4.06 (m, 2H, OCH_2CH_3), 2.89 (dd, *J* = 10.2, 7.6 Hz, 1H, C*H*), 2.82 (s, 3H, CH_3), 2.60 (s, 3H, CH_3), 2.39 (s, 3H, CH_3), 2.17 – 2.02 (m, 2H, CH_2), 1.98 – 1.79 (m, 2H, CH_2), 1.70 – 1.52 (m, 3H, CH_2), 1.45 – 1.30 (m, 1H, CH_2), 1.28 (t, *J* = 7.2 Hz, 3H, OCH_2CH_3), 1.24 – 1.15 (m, 1H, CH_2), 1.09 – 0.92 (m, 1H, CH_2).
^{13}C NMR (101 MHz, $CDCl_3$): δ 172.6, 151.5, 143.2, 136.3, 133.2, 129.8, 128.3, 127.2, 123.4, 117.6, 105.7, 79.2, 67.0, 60.9, 60.7, 52.0, 32.6, 30.4, 27.7, 27.6, 26.2, 21.6, 18.3, 18.1, 14.3.
HRMS (ESI/QTOF) m/z: $[M + H]^+$ Calcd for $C_{27}H_{35}N_2O_4S^+$ 483.2312; Found 483.2314.

Ethyl-1-((*N*,4-dimethylphenyl)sulfonamido)-4,5-dimethyl-1,2,3,3a,4,8b-hexahydrocyclopenta[b]indole-3-carboxylate (4l)

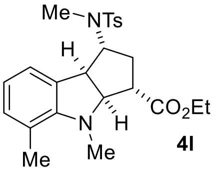

4l

Chemical Formula: $C_{24}H_{30}N_2O_4S$
Molecular Weight: 442.5740

Prepared according to the general procedure E from Tf_2NH (0.222 M, 24.5 µL, 5.5 µmol, 5 mol%), silyl enol ether **3b** (5.9 mg, 27 µmol, 0.25 equiv.), aminocyclopropane **1d** (32.4 mg, 109 µmol, 1.0 equiv.) and indole **2l** (16.6 mg, 114 µmol, 1.05 equiv.) in DCM (0.1 M, 1 mL). The crude product was purified by PREP TLC using pent/EtOAc 80:20 to afford cycloadduct **4l** as a colorless oil (37.3 mg, 84.3 µmol, 77%, dr 93:7).
Rf = 0.4 (pentane/EtOAc 4:1).
^{1}H NMR (400 MHz, $CDCl_3$): δ 7.65 – 7.60 (m, 2H, Ts), 7.27 – 7.22 (m, 2H, Ts), 6.88 (d, *J* = 7.5 Hz, 1H, Ar*H*), 6.84 (d, *J* = 7.3 Hz, 1H, Ar*H*), 6.61 (t, *J* = 7.4 Hz, 1H, Ar*H*), 4.32 – 4.10 (m, 3H, OCH_2CH_3, C*H*), 3.90 (dd, *J* = 10.4, 6.0 Hz, 1H, C*H*), 3.68 – 3.60 (m, 1H, C*H*), 2.96 (s, 3H, CH_3), 2.89 (s, 3H, CH_3), 2.85 – 2.77 (m, 1H, C*H*), 2.41 (s, 3H, CH_3), 2.33 (s, 3H, CH_3), 1.84 – 1.67 (m, 2H, CH_2), 1.28 (t, *J* = 7.1 Hz, 3H, OCH_2CH_3).
^{13}C NMR (101 MHz, $CDCl_3$): δ 174.6, 149.7, 143.3, 136.6, 131.5, 130.9, 129.8, 127.2, 122.3, 120.8, 120.0, 74.2, 64.3, 61.0, 50.0, 47.8, 39.3, 30.6, 29.2, 21.6, 19.5, 14.3.
IR (ν_{max}, cm^{-1}) 3033 (w), 2863 (w), 1729 (m), 1470 (m), 1339 (m), 1160 (s), 978 (m).

[1] The NOESY experiment did not allow us to assign the relative configuration of the minor isomer. The stereochemistry was thus assumed as disclosed as compared to other cycloadducts.

HRMS (ESI/QTOF) m/z: $[M + H]^+$ Calcd for $C_{24}H_{31}N_2O_4S^+$ 443.1999; Found 443.1999.

Ethyl-9-(tert-butyldimethylsilyl)-6-((*N*,4-dimethylphenyl)sulfonamido)-2,3,5b,6,7,8,8a,9-octahydro-1H-dicyclopenta[b,g]indole-8-carboxylate (4m)

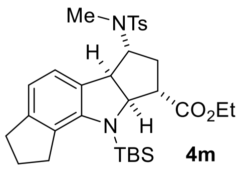

4m
Chemical Formula: $C_{31}H_{44}N_2O_4SSi$
Molecular Weight: 568.8480

Prepared according to the general procedure E from Tf_2NH (0.192 M, 13.2 µL, 2.53 µmol, 2.5 mol%), silyl enol ether **3b** (5.5 mg, 25 µmol, 0.25 equiv.), aminocyclopropane **1d** (30.1 mg, 101 µmol, 1.0 equiv.) and indole **2m** (28.9 mg, 106 µmol, 1.05 equiv.) in DCM (0.1 M, 1 mL). The crude product was purified by flash chromatography using pent/Et_2O 85:15 providing cycloadduct as a mixture of diastereomers (34 mg, 60 µmol, 59%, dr 88:12). The mixture was further purified by PREP TLC using pent/EtOAc 80:20 to afford cycloadduct **4m** as pure isomer and white solid. The minor isomer was not obtained in a pure fraction.
Rf = 0.48 (pentane/EtOAc 4:1).
m. p. = 136.4 – 138.5 °C.
^{1}H NMR (400 MHz, $CDCl_3$): δ 7.70 (d, *J* = 8.3 Hz, 2H, Ts), 7.30 – 7.25 (m, 2H, Ts), 7.09 (d, *J* = 7.4 Hz, 1H, Ar*H*), 6.78 (d, *J* = 7.4 Hz, 1H, Ar*H*), 4.60 (td, *J* = 8.6, 3.0 Hz, 1H, C*H*), 4.26 (t, *J* = 8.5 Hz, 1H, C*H*), 4.11 (q, *J* = 7.1 Hz, 2H, OCH_2CH_3), 3.55 (d, *J* = 7.6 Hz, 1H, C*H*), 2.90 (s, 3H, CH_3), 2.89 – 2.69 (m, 4H, 2xCH_2), 2.48 – 2.37 (m, 4H, CH_3, C*H*), 2.13 – 2.03 (m, 1H, CH_2), 1.97 – 1.85 (m, 1H, CH_2), 1.78 – 1.67 (m, 1H, CH_2), 1.65 – 1.55 (m, 1H, CH_2), 1.25 (t, *J* = 7.1 Hz, 3H, OCH_2CH_3), 0.75 (s, 9H, *t*Bu), 0.32 (s, 3H, CH_3), 0.11 (s, 3H, CH_3).
^{13}C NMR (101 MHz, $CDCl_3$): δ 174.9, 146.3, 145.6, 143.5, 136.5, 133.3, 129.9, 129.3, 127.3, 122.5, 117.1, 71.8, 63.0, 60.8, 50.5, 50.4, 33.6, 33.2, 29.9, 29.8, 26.8, 26.2, 21.6, 20.1, 14.3, -1.3, -2.6.
IR (ν_{max}, cm^{-1}) 3058 (w), 2948 (w), 1725 (m), 1433 (m), 1339 (m), 1162 (s), 1014 (m), 910 (m).
HRMS (ESI/QTOF) m/z: $[M + H]^+$ Calcd for $C_{31}H_{45}N_2O_4SSi^+$ 569.2864; Found 569.2875.

Ethyl-1-((*N*,4-dimethylphenyl)sulfonamido)-6-methoxy-4-methyl-1,2,3,3a,4,8b-hexahydrocyclopenta[b]indole-3-carboxylate (4n)

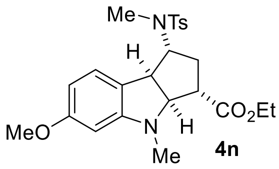

4n
Chemical Formula: $C_{24}H_{30}N_2O_5S$
Molecular Weight: 458.5730

Prepared according to the general procedure E from Tf_2NH (0.229 M, 66 µL, 15 µmol, 5 mol%), silyl enol ether **3b** (16.4 mg, 75.7 µmol, 0.25 equiv.), aminocyclopropane **1d** (90.1 mg, 303 µmol, 1.0 equiv.) and indole **2n** (51.3 mg, 318 µmol, 1.05 equiv.) in DCM (0.3 M, 1 mL). The crude product was purified by flash chromatography using pent/EtOAc 75:25 to afford cycloadduct **4n** as a colorless oil (68 mg, 0.15 mmol, 49%, dr 92:8).
Rf = 0.4 (pentane/EtOAc 4:1).
^{1}H NMR (400 MHz, $CDCl_3$): δ 7.65 – 7.61 (m, 2H, Ts), 7.27 – 7.23 (m, 2H, Ts), 6.79 (dd, *J* = 8.1, 0.9 Hz, 1H, Ar*H*), 6.09 (dd, *J* = 8.0, 2.3 Hz, 1H, Ar*H*), 5.96 (d, *J* = 2.3 Hz, 1H, Ar*H*), 4.36 – 4.27 (m, 1H, C*H*), 4.22 – 4.12 (m, 2H, OCH_2CH_3), 4.08 (dd, *J* = 10.1, 5.5 Hz, 1H, C*H*), 3.76 (s, 3H, CH_3), 3.51 – 3.44 (m, 1H, C*H*), 2.89 (s, 3H, CH_3), 2.85 – 2.78 (m, 1H, C*H*), 2.77 (s, 3H, CH_3), 2.41 (s, 3H, CH_3), 1.92 – 1.73 (m, 2H, CH_2), 1.27 (t, *J* = 7.1 Hz, 3H, OCH_2CH_3).
^{13}C NMR (101 MHz, $CDCl_3$): δ 174.4, 161.0, 152.4, 143.3, 136.6, 129.8, 127.2, 124.6, 121.6, 101.9, 94.2, 73.3, 63.9, 61.1, 55.5, 48.5, 47.4, 33.3, 31.4, 29.2, 21.6, 14.3.
IR (ν_{max}, cm^{-1}) 3032 (w), 2925 (w), 1728 (m), 1614 (m), 1493 (m), 1337 (s), 1162 (s).
HRMS (ESI/QTOF) m/z: $[M + H]^+$ Calcd for $C_{24}H_{31}N_2O_5S^+$ 459.1948; Found 459.1954.

Ethyl-1-((*N*,4-dimethylphenyl)sulfonamido)-7-(1,3-dioxoisoindolin-2-yl)-4-methyl-1,2,3,3a,4,8b-hexahydrocyclopenta[b]indole-3-carboxylate (4o)

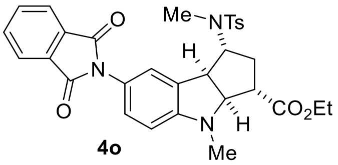

4o

Chemical Formula: $C_{31}H_{31}N_3O_6S$
Molecular Weight: 573.6640

Prepared according to the general procedure E from Tf_2NH (0.218 M, 48.3 µL, 10.5 µmol, 10 mol%), silyl enol ether **3b** (11.4 mg, 52.6 µmol, 0.5 equiv.), aminocyclopropane **1d** (31.3 mg, 105 µmol, 1.0 equiv.) and indole **2o** (30.5 mg, 111 µmol, 1.05 equiv.) in DCM (0.07 M, 1.5 mL). The crude product was purified by PREP TLC using DCM to afford cycloadduct **4o** as a yellow solid (41.3 mg, 72.0 µmol, 68%, dr 92:8).

Rf = 0.2 (DCM).

m. p. = 95 – 98 °C.

^{1}H NMR (400 MHz, $CDCl_3$): δ 7.97 – 7.91 (m, 2H, Ar*H*), 7.80 – 7.74 (m, 2H, Ar*H*), 7.66 – 7.62 (m, 2H, Ts), 7.21 – 7.16 (m, 2H, Ts), 7.10 (dd, *J* = 8.3, 2.1 Hz, 1H, Ar*H*), 6.94 – 6.91 (m, 1H, Ar*H*), 6.45 (d, *J* = 8.4 Hz, 1H, Ar*H*), 4.47 – 4.38 (m, 1H, C*H*), 4.26 – 4.12 (m, 3H, C*H*, OC*H*$_2$CH$_3$), 3.68 – 3.61 (m, 1H, C*H*), 2.87 (s, 3H, C*H*$_3$), 2.85 – 2.77 (m, 4H, C*H*$_3$, C*H*), 2.21 (s, 3H, C*H*$_3$), 1.95 – 1.77 (m, 2H, C*H*$_2$), 1.28 (t, *J* = 7.1 Hz, 3H, OCH$_2$C*H*$_3$).

^{13}C NMR (101 MHz, $CDCl_3$): δ 174.2, 167.9, 150.9, 143.3, 136.5, 134.3, 132.1, 129.7, 127.6, 127.5, 127.4, 123.6, 123.2, 121.2, 106.3, 72.6, 63.8, 61.2, 48.8, 47.8, 33.1, 31.4, 29.2, 21.4, 14.3.

IR (ν_{max}, cm^{-1}) 3056 (w), 2941 (w), 1721 (s), 1496 (m), 1341 (s), 1161 (s), 731 (s).

HRMS (ESI/QTOF) m/z: $[M + H]^+$ Calcd for $C_{31}H_{32}N_3O_6S^+$ 574.2006; Found 574.2016.

Ethyl-7-chloro-1-((*N*,4-dimethylphenyl)sulfonamido)-4-methyl-1,2,3,3a,4,8b-hexahydrocyclopenta[b]indole-3-carboxylate (4p)

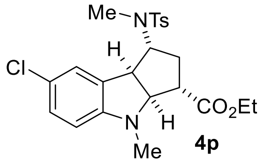

4p

Chemical Formula: $C_{23}H_{27}ClN_2O_4S$
Molecular Weight: 462.9890

Prepared according to the general procedure E from Tf_2NH (0.211 M, 12.3 µL, 2.6 µmol, 2.5 mol%), silyl enol ether **3b** (5.6 mg, 26 µmol, 0.25 equiv.), aminocyclopropane **1d** (30.9 mg, 104 µmol, 1.0 equiv.) and indole **2p** (18.1 mg, 109 µmol, 1.05 equiv.) in DCM (0.1 M, 1 mL). The crude product was purified by PREP TLC using pent/EtOAc 80:20 to afford cycloadduct **4p** as a colorless oil (41 mg, 88 µmol, 85%, dr 92:8).

Rf = 0.34 (pentane/EtOAc 4:1).

^{1}H NMR (400 MHz, $CDCl_3$): δ 7.61 (d, *J* = 8.3 Hz, 2H, Ts), 7.29 – 7.22 (m, 2H, Ts), 7.00 (dd, *J* = 8.4, 2.2 Hz, 1H, Ar*H*), 6.60 – 6.59 (m, 1H, Ar*H*), 6.26 (d, *J* = 8.4 Hz, 1H, Ar*H*), 4.34 – 4.26 (m, 1H, C*H*), 4.24 – 4.13 (m, 2H, OC*H*$_2$CH$_3$), 4.07 (dd, *J* = 10.1, 5.1 Hz, 1H, C*H*), 3.46 (t, *J* = 9.4 Hz, 1H, C*H*), 2.91 (s, 3H, C*H*$_3$), 2.89 – 2.80 (m, 1H, C*H*), 2.75 (s, 3H, C*H*$_3$), 2.41 (s, 3H, C*H*$_3$), 1.98 – 1.89 (m, 2H, C*H*$_2$), 1.28 (t, *J* = 7.1 Hz, 3H, OCH$_2$C*H*$_3$).

^{13}C NMR (101 MHz, $CDCl_3$): δ 174.2, 149.8, 143.7, 136.5, 130.6, 129.9, 128.2, 127.2, 124.4, 122.3, 107.4, 73.2, 63.5, 61.2, 48.2, 47.5, 33.5, 32.1, 29.0, 21.8, 14.3.

IR (ν_{max}, cm^{-1}) 3059 (w), 2820 (m), 1729 (s), 1487 (m), 1338 (s), 1159 (s), 809 (s).

HRMS (ESI/QTOF) m/z: $[M + H]^+$ Calcd for $C_{23}H_{28}ClN_2O_4S^+$ 463.1453; Found 463.1458.

Ethyl-7-bromo-1-((*N*,4-dimethylphenyl)sulfonamido)-4-methyl-1,2,3,3a,4,8b-hexahydrocyclopenta[b]indole-3-carboxylate (4q)

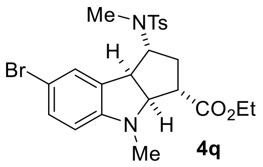

4q

Chemical Formula: $C_{23}H_{27}BrN_2O_4S$
Molecular Weight: 507.4430

Prepared according to the general procedure E from Tf_2NH (0.213 M, 73.4 μL, 15.6 μmol, 5 mol%), silyl enol ether **3b** (16.9 mg, 78.2 μmol, 0.25 equiv.), aminocyclopropane **1d** (93 mg, 0.31 mmol, 1.0 equiv.) and indole **2q** (69 mg, 0.33 mmol, 1.05 equiv.) in DCM (0.3 M, 1 mL). The crude product was purified by flash chromatography using pent/EtOAc 75:25 to afford cycloadduct **4q** as a colorless oil (139 mg, 274 μmol, 88%, dr 94:6).

Rf = 0.3 (pentane/EtOAc 4:1).

^{1}H NMR (400 MHz, $CDCl_3$): δ 7.63 – 7.59 (m, 2H, Ts), 7.28 – 7.25 (m, 2H, Ts), 7.14 (dd, *J* = 8.3, 2.1 Hz, 1H, Ar*H*), 6.77 (dd, *J* = 2.1, 0.9 Hz, 1H, Ar*H*), 6.22 (d, *J* = 8.3 Hz, 1H, Ar*H*), 4.34 – 4.26 (m, 1H, C*H*), 4.22 – 4.13 (m, 2H, OCH_2CH_3), 4.07 (dd, *J* = 10.1, 5.2 Hz, 1H, C*H*), 3.47 (t, *J* = 9.4 Hz, 1H, C*H*), 2.90 (s, 3H, CH_3), 2.88 – 2.80 (m, 1H, C*H*), 2.75 (s, 3H, CH_3), 2.42 (s, 3H, CH_3), 1.98 – 1.90 (m, 2H, CH_2), 1.27 (t, *J* = 7.2 Hz, 3H, OCH_2CH_3).

^{13}C NMR (101 MHz, $CDCl_3$): δ 174.1, 150.2, 143.6, 136.5, 131.1, 131.1, 129.9, 127.2, 127.1, 109.2, 108.0, 73.0, 63.5, 61.2, 48.2, 47.5, 33.4, 31.9, 29.0, 21.8, 14.3.

IR (ν_{max}, cm^{-1}) 3045 (m), 1726 (s), 1485 (s), 1342 (s), 1162 (s), 978 (s), 804 (s).

HRMS (ESI/QTOF) m/z: $[M + H]^+$ Calcd for $C_{23}H_{28}{}^{79}BrN_2O_4S^+$ 507.0948; Found 507.0945.

Ethyl-6-bromo-1-((*N*,4-dimethylphenyl)sulfonamido)-4-methyl-1,2,3,3a,4,8b-hexahydrocyclopenta[b]indole-3-carboxylate (4r)

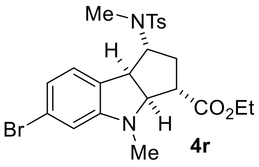

4r

Chemical Formula: $C_{23}H_{27}BrN_2O_4S$
Molecular Weight: 507.4430

Prepared according to the general procedure E from Tf_2NH (0.173 M, 14.7 μL, 2.5 μmol, 2.5 mol%), silyl enol ether **3b** (5.5 mg, 25 μmol, 0.25 equiv.), aminocyclopropane **1d** (30.2 mg, 102 μmol, 1.0 equiv.) and indole **2r** (22.4 mg, 107 μmol, 1.05 equiv.) in DCM (0.1 M, 1 mL). The crude product was purified by PREP TLC using pent/EtOAc 75:25 to afford cycloadduct **4r** as a colorless oil (41 mg, 82 μmol, 81%, dr 95:5).

Rf = 0.33 (pentane/EtOAc 4:1).

^{1}H NMR (400 MHz, $CDCl_3$): δ 7.62 – 7.58 (m, 2H, Ts), 7.26 – 7.22 (m, 2H, Ts), 6.70 (dd, *J* = 7.8, 0.9 Hz, 1H, Ar*H*), 6.62 (dd, *J* = 7.7, 1.7 Hz, 1H, Ar*H*), 6.47 (d, *J* = 1.7 Hz, 1H, Ar*H*), 4.28 (ddd, *J* = 11.5, 8.7, 6.5 Hz, 1H, C*H*), 4.22 – 4.12 (m, 2H, OCH_2CH_3), 4.09 (dd, *J* = 10.1, 5.3 Hz, 1H, C*H*), 3.50 – 3.42 (m, 1H, C*H*), 2.89 (s, 3H, CH_3), 2.85 – 2.78 (m, 1H, C*H*), 2.77 (s, 3H, CH_3), 2.42 (s, 3H, CH_3), 1.93 – 1.73 (m, 2H, CH_2), 1.27 (t, *J* = 7.1 Hz, 3H, OCH_2CH_3).

^{13}C NMR (101 MHz, $CDCl_3$): δ 174.1, 152.2, 143.6, 136.5, 129.9, 128.0, 127.2, 125.4, 122.3, 120.4, 109.5, 72.7, 63.6, 61.2, 48.5, 47.4, 33.0, 31.3, 29.1, 21.7, 14.3.

IR (ν_{max}, cm^{-1}) 3058 (w), 2947 (w), 1727 (m), 1601 (w), 1488 (w), 1339 (w), 1160 (m), 733 (s).

HRMS (ESI/QTOF) m/z: $[M + H]^+$ Calcd for $C_{23}H_{28}{}^{79}BrN_2O_4S^+$ 507.0948; Found 507.0958.

Ethyl (1R,3S,3aS,8bR)-1-((*N*,4-dimethylphenyl)sulfonamido)-8-fluoro-4-methyl-1,2,3,3a,4,8b-hexahydrocyclopenta[b]indole-3-carboxylate (4s)

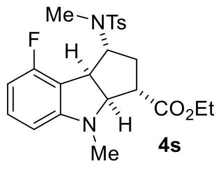

4s

Chemical Formula: $C_{23}H_{27}FN_2O_4S$
Molecular Weight: 446.5374

Prepared according to the general procedure E from Tf_2NH (0.173 M, 14.8 μL, 2.6 μmol, 2.5 mol%), silyl enol ether **3b** (5.5 mg, 26 μmol, 0.25 equiv.), aminocyclopropane **1d** (30.4 mg, 102 μmol, 1.0 equiv.) and indole **2s** (16.0 mg, 107 μmol, 1.05 equiv.) in DCM (0.1 M, 1 mL). The crude product was purified by PREP TLC using

pent/EtOAc 75:25 to afford cycloadduct **4s** as a colorless oil (30.3 mg, 67.9 μmol, 66%, dr 93:7).

Rf = 0.37 (pentane/EtOAc 4:1).

^{1}H NMR (400 MHz, $CDCl_3$): δ 7.61 – 7.55 (m, 2H, Ts), 7.20 – 7.15 (m, 2H, Ts), 7.05 – 6.97 (m, 1H, Ar*H*), 6.21 – 6.13 (m, 2H, Ar*H*), 4.38 (q, *J* = 9.3 Hz, 1H, C*H*), 4.26 – 4.13 (m, 2H, OC*H*$_2$CH$_3$), 4.07 (dd, *J* = 9.7, 4.2 Hz, 1H, C*H*), 3.65 (t, *J* = 9.6 Hz, 1H, C*H*), 2.99 – 2.89 (m, 4H, C*H*, C*H*$_3$), 2.77 (s, 3H, C*H*$_3$), 2.39 (s, 3H, C*H*$_3$), 2.07 – 1.98 (m, 2H, C*H*$_2$), 1.29 (t, *J* = 7.1 Hz, 3H, OCH$_2$C*H*$_3$).

^{13}C NMR (101 MHz, $CDCl_3$): δ 174.1, 159.5 (d, *J* = 245.8 Hz), 154.0 (d, *J* = 9.1 Hz), 143.0, 136.9, 130.2 (d, *J* = 8.8 Hz), 129.6, 127.2, 114.2 (d, *J* = 21.2 Hz), 105.3 (d, *J* = 21.2 Hz), 102.8 (d, *J* = 2.6 Hz), 73.9, 62.9, 61.3, 47.7, 45.2, 33.7, 32.5, 28.4, 21.6, 14.3.

^{19}F NMR (376 MHz, $CDCl_3$): δ -118.6 (dd, *J* = 8.8, 5.6 Hz).

IR (ν_{max}, cm^{-1}) 3058 (w), 2952 (m), 1728 (s), 1475 (m), 1338 (s), 1158 (s), 974 (s), 730 (s).

HRMS (ESI/QTOF) m/z: $[M + H]^+$ Calcd for $C_{23}H_{28}FN_2O_4S^+$ 447.1748; Found 447.1758.

Ethyl-1-((*N*,4-dimethylphenyl)sulfonamido)-4-methyl-8-(4,4,5,5-tetramethyl-1,3,2-dioxaborolan-2-yl)-1,2,3,3a,4,8b-hexahydrocyclopenta[b]indole-3-carboxylate (4t)

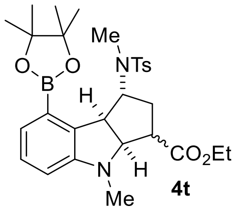

4t

Chemical Formula: $C_{29}H_{39}BN_2O_6S$
Molecular Weight: 554.5090

Prepared according to the general procedure E from Tf_2NH (0.23 M, 89.2 μL, 20.5 μmol, 20 mol%), silyl enol ether **3b** (11.1 mg, 51.3 μmol, 0.5 equiv.), aminocyclopropane **1d** (30.5 mg, 103 μmol, 1.0 equiv.) and indole **2t** (27.7 mg, 108 μmol, 1.05 equiv.) in DCM (0.1 M, 1 mL). The crude product was purified by PREP TLC using pent/EtOAc 80:20 to afford cycloadduct **4t** as a white solid and an inseparable mixture of diasteromers (34.1 mg, 61.5 μmol, 60%, dr 84:16).

Characterized as mixture of diastereomers

Rf = 0.34 (pentane/EtOAc 4:1).

m. p. = 189 – 191 °C.

^{1}H NMR (400 MHz, $CDCl_3$, signals for the major isomer): δ 7.54 – 7.49 (m, 2H, Ts), 7.19 – 7.11 (m, 4H, Ts, Ar*H*), 6.54 (dd, *J* = 7.1, 1.8 Hz, 1H, Ar*H*), 4.27 – 4.09 (m, 3H, OC*H*$_2$CH$_3$, C*H*), 3.85 (dd, *J* = 8.2, 2.2 Hz, 1H, C*H*), 3.76 (dd, *J* = 9.8, 8.2 Hz, 1H, C*H*), 3.01 (td, *J* = 8.9, 2.2 Hz, 1H, C*H*), 2.94 (s, 3H, C*H*$_3$), 2.73 (s, 3H, C*H*$_3$), 2.37 (s, 3H, C*H*$_3$), 1.99 (ddd, *J* = 12.9, 11.5, 9.2 Hz, 1H, C*H*$_2$), 1.75 (ddd, *J* = 12.9, 8.5, 7.0 Hz, 1H, C*H*$_2$), 1.33 (s, 6H, 2xC*H*$_3$), 1.31 – 1.23 (m, 9H, 2xC*H*$_3$, OCH$_2$C*H*$_3$).

^{13}C NMR (101 MHz, $CDCl_3$, signals for the major isomer): δ 174.7, 150.7, 142.9, 136.7, 136.2, 129.7, 129.6, 127.6, 127.3, 125.7, 110.1, 83.5, 75.7, 63.6, 61.1, 48.0, 46.5, 34.3, 29.9, 29.2, 26.0, 24.2, 21.6, 14.3.

IR (ν_{max}, cm^{-1}) 3056 (w), 2981 (m), 1728 (m), 1345 (s), 1155 (s), 1111 (s), 973 (m).

HRMS (ESI/QTOF) m/z: $[M + H]^+$ Calcd for $C_{29}H_{40}BN_2O_6S^+$ 555.2695; Found 555.2709.

Ethyl-1-((*N*,4-dimethylphenyl)sulfonamido)-4-methyl-6-(trifluoromethyl)-1,2,3,3a,4,8b-hexahydrocyclopenta[b]indole-3-carboxylate (4u)

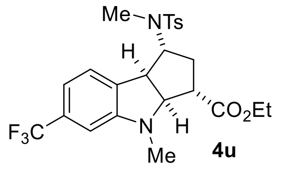

4u

Chemical Formula: $C_{24}H_{27}F_3N_2O_4S$
Molecular Weight: 496.5452

Prepared according to the general procedure E from Tf_2NH (0.173 M, 114 µL, 19.7 µmol, 20 mol%), silyl enol ether **3b** (10.7 mg, 49.3 µmol, 0.5 equiv.), aminocyclopropane **1d** (29.3 mg, 98.5 µmol, 1.0 equiv.) and indole **2u** (20.6 mg, 103 µmol, 1.05 equiv.) in DCM (0.1 M, 1 mL). The crude product was purified by PREP TLC using pent/EtOAc 75:25 to afford cycloadduct **4u** as a colorless oil (33.6 mg, 67.7 µmol, 69%, dr 93:7).

Rf = 0.26 (pentane/EtOAc 4:1).

^{1}H NMR (400 MHz, $CDCl_3$): δ 7.62 – 7.58 (m, 2H, Ts), 7.25 – 7.21 (m, 2H, Ts), 6.91 (d, *J* = 7.6 Hz, 1H, Ar*H*), 6.77 – 6.73 (m, 1H, Ar*H*), 6.52 (brs, 1H, Ar*H*), 4.30 (ddd, *J* = 11.6, 8.8, 6.5 Hz, 1H, C*H*), 4.24 – 4.11 (m, 3H, OCH_2CH_3, C*H*), 3.54 (t, *J* = 9.5 Hz, 1H, C*H*), 2.92 (s, 3H, CH_3), 2.87 – 2.79 (m, 4H, C*H*, CH_3), 2.41 (s, 3H, CH_3), 1.95 – 1.76 (m, 2H, CH_2), 1.28 (t, *J* = 7.1 Hz, 3H, OCH_2CH_3).

^{13}C NMR (101 MHz, $CDCl_3$): δ 174.0, 151.2, 143.6, 136.4, 132.7, 130.8 (q, *J* = 31.5 Hz), 129.9, 127.1, 124.7 (q, *J* = 272.2 Hz), 124.2, 114.7 (q, *J* = 3.9 Hz), 102.5 (q, *J* = 3.4 Hz), 72.6, 63.6, 61.3, 48.5, 47.6, 33.0, 31.3, 29.1, 21.6, 14.3.

^{19}F NMR (376 MHz, $CDCl_3$): δ -62.4.

IR (ν_{max}, cm^{-1}) 3060 (w), 2946 (w), 1728 (m), 1316 (m), 1270 (m), 1161 (s), 1119 (s).

HRMS HRMS (ESI/QTOF) m/z: $[M + H]^+$ Calcd for $C_{24}H_{28}F_3N_2O_4S^+$ 497.1716; Found 497.1727.

4.2. Scope of aminocyclopropanes

Ethyl-4-benzyl-1-((*N*-benzyl-4-methylphenyl)sulfonamido)-1,2,3,3a,4,8b-hexahydrocyclopenta[b]indole-3-carboxylate (5a)

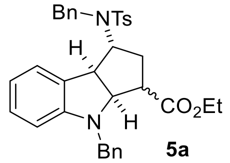

5a

Chemical Formula: $C_{35}H_{36}N_2O_4S$
Molecular Weight: 580.7430

Prepared according to the general procedure E from Tf_2NH (0.219 M, 70.6 µL, 15.5 µmol, 5 mol%), silyl enol ether **3b** (16.7 mg, 77.3 µmol, 0.25 equiv.), aminocyclopropane **1g** (116 mg, 309 µmol, 1.0 equiv.) and benzyl-protected indole **1c** (67.3 mg, 325 µmol, 1.05 equiv.) in DCM (0.3 M, 1 mL). The crude product was purified by flash chromatography using pent/EtOAc 85:15 to afford cycloadduct **5a** as a white solid and an inseparable mixture of diastereoisomers (159 mg, 274 µmol, 89%, dr 80:20).

Characterized as mixture of diastereomers

Rf = 0.57 (pentane/EtOAc 4:1).

^{1}H NMR (400 MHz, $CDCl_3$, signals for major isomer): δ 7.68 – 7.61 (m, 2H, Ts), 7.48 – 7.44 (m, 2H, Ts), 7.37 – 7.12 (m, 10H, Ar*H*), 6.99 (td, *J* = 7.7, 1.3 Hz, 1H, Ar*H*), 6.74 (d, *J* = 7.3 Hz, 1H, Ar*H*), 6.49 (td, *J* = 7.4, 1.0 Hz, 1H, Ar*H*), 6.32 (d, *J* = 7.8 Hz, 1H, Ar*H*), 4.75 (d, *J* = 15.8 Hz, 1H, Bn), 4.38 – 4.26 (m, 3H, Bn), 4.23 – 4.12 (m, 2H, 2xC*H*), 4.00 (qd, *J* = 7.1, 2.0 Hz, 2H, OCH_2CH_3), 3.66 – 3.58 (m, 1H, C*H*), 2.73 (ddd, *J* = 11.6, 7.4, 5.9 Hz, 1H, C*H*), 2.42 (s, 3H, CH_3), 1.98 – 1.83 (m, 2H, CH_2), 1.13 (t, *J* = 7.1 Hz, 3H, OCH_2CH_3).

^{13}C NMR (101 MHz, $CDCl_3$, signals for major isomer): δ 174.1, 150.5, 143.5, 138.4, 137.8, 137.7, 129.9, 128.9, 128.8, 128.6, 128.3, 128.2, 127.9, 127.4, 127.3, 127.1, 124.5, 117.7, 106.8, 70.4, 65.4, 61.0, 51.1, 49.2, 48.5, 48.2, 33.0, 21.6, 14.1.

IR (ν_{max}, cm^{-1}) 3034 (w), 2980 (w), 1726 (m), 1487 (m), 1338 (m), 1156 (s), 911 (m), 734 (s).

HRMS (ESI/QTOF) m/z: $[M + H]^+$ Calcd for $C_{35}H_{37}N_2O_4S^+$ 581.2469; Found 581.2469.

2,2,2-Trifluoroethyl-1-((*N*,4-dimethylphenyl)sulfonamido)-4-methyl-1,2,3,3a,4,8b-hexahydrocyclopenta[b]indole-3-carboxylate (5b)

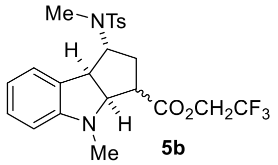

5b

Chemical Formula: $C_{23}H_{25}F_3N_2O_4S$
Molecular Weight: 482.5182

Prepared according to the general procedure E from Tf_2NH (0.219 M, 50.3 µL, 11 µmol, 10 mol%), silyl enol ether **3b** (6.0 mg, 27 µmol, 0.25 equiv.), aminocyclopropane **1h** (38.7 mg, 110 µmol, 1.0 equiv.) and 1-methylindole **2a** (14.9 µL, 116 µmol, 1.05 equiv.) in DCM (0.1 M, 1 mL). The crude product was purified by PREP TLC using pent/EtOAc 80:20 to afford cycloadduct **5b** as a colorless oil and an inseparable mixture of diastereoisomers (38.3 mg, 79.4 µmol, 72%, dr 83:17).

Characterized as mixture of diastereomers

Rf = 0.39 (pentane/EtOAc 4:1).

^{1}H NMR (400 MHz, $CDCl_3$, signals for major isomer): δ 7.65 – 7.60 (m, 2H, Ts), 7.27 – 7.22 (m, 2H, Ts), 7.10 (td, *J* = 7.8, 1.3 Hz, 1H, Ar*H*), 6.87 (d, *J* = 7.4 Hz, 1H, Ar*H*), 6.56 (td, *J* = 7.4, 1.0 Hz, 1H, Ar*H*), 6.41 (d, *J* = 7.8 Hz, 1H, Ar*H*), 4.66 – 4.34 (m, 3H, OCH_2CF_3, C*H*), 4.08 (dd, *J* = 10.1, 5.3 Hz, 1H, C*H*), 3.60 – 3.53 (m, 1H, C*H*), 3.00 – 2.93 (m, 1H, C*H*), 2.92 (s, 3H, CH_3), 2.79 (s, 3H, CH_3), 2.42 (s, 3H, CH_3), 1.99 – 1.81 (m, 2H, CH_2).

^{13}C NMR (101 MHz, $CDCl_3$, signals for major isomer): δ 172.9, 150.9, 143.5, 136.5, 129.9, 128.7, 128.5, 127.2, 124.4, 122.9 (q, *J* = 277.3 Hz), 118.2, 106.9, 72.9, 63.7, 60.7 (q, *J* = 36.6 Hz), 48.0, 47.9, 33.5, 31.5, 29.2, 21.6.

^{19}F NMR (376 MHz, $CDCl_3$): δ -73.7.

IR (ν_{max}, cm^{-1}) 3045 (m), 2973 (m), 1754 (m), 1338 (m), 1281 (s), 1155 (s), 975 (m), 739 (s).

HRMS (ESI/QTOF) m/z: $[M + H]^+$ Calcd for $C_{23}H_{26}F_3N_2O_4S^+$ 483.1560; Found 483.1555.

Ethyl-6-methyl-1-tosyl-1,2,3,4,4a,5,5a,6,10b,10c-decahydropyrido[2',3':3,4]cyclopenta[1,2-b]indole-5-carboxylate (5c)

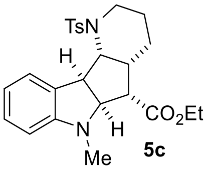

5c

Chemical Formula: $C_{25}H_{30}N_2O_4S$
Molecular Weight: 454.5850

Prepared according to the general procedure E from Tf_2NH (0.236 M, 129 µL, 30.5 µmol, 0.1 equiv.), silyl enol ether **3b** (16.5 mg, 76.1 µmol, 0.25 equiv.), aminocyclopropane **1i** (98.5 mg, 305 µmol, 1.0 equiv.) and 1-methylindole **2a** (41.1 µL, 320 µmol, 1.05 equiv.) in DCM (0.3 M, 1 mL). The crude product was purified by flash chromatography using pent/EtOAc 80:20 to afford cycloadduct **5c** as a mixture of diastereomers and a colorless oil (104 mg, 229 µmol, 75%, dr 67:33). Half of the mixture was further purified by PREP TLC using pent/EtOAc 80:20 allowing the isolation and clean NMR characterization of each diastereomer.

Data for the major diastereomer

Rf = 0.6 (pentane/EtOAc 4:1).

^{1}H NMR (400 MHz, $CDCl_3$): δ 7.81 – 7.76 (m, 2H, Ts), 7.34 – 7.29 (m, 2H, Ts), 7.09 (td, *J* = 7.7, 1.3 Hz, 1H, Ar*H*), 6.97 (d, *J* = 7.4 Hz, 1H, Ar*H*), 6.54 (td, *J* = 7.4, 1.0 Hz, 1H, Ar*H*), 6.38 (dd, *J* = 8.0, 0.9 Hz, 1H, Ar*H*), 4.59 (t, *J* = 10.3 Hz, 1H, C*H*), 4.24 – 4.17 (m, 3H, OCH_2CH_3, C*H*), 4.07 – 4.00 (m, 1H, CH_2), 3.10 – 3.03 (m, 1H, C*H*), 2.83 – 2.73 (m, 4H, CH_3, CH_2), 2.44 (s, 3H, CH_3), 2.40 (dd, *J* = 11.3, 5.6 Hz, 1H, C*H*), 2.16 – 2.05 (m, 2H, C*H*, CH_2), 1.57 – 1.47 (m, 2H, CH_2), 1.29 (t, *J* = 7.1 Hz, 3H, OCH_2CH_3), 1.26 – 1.18 (m, 1H, CH_2).

^{13}C NMR (101 MHz, $CDCl_3$): δ 173.7, 151.6, 143.5, 139.3, 129.9, 129.6, 128.5, 127.6, 125.8, 117.5, 106.4, 72.6, 72.2, 61.0, 54.4, 50.3, 47.2, 45.7, 33.7, 30.4, 24.2, 21.7, 14.4.
IR (ν_{max}, cm^{-1}) 3054 (w), 2937 (m), 1729 (s), 1603 (m), 1490 (m), 1308 (s), 1158 (s), 738 (s).
HRMS (ESI/QTOF) m/z: $[M + H]^+$ Calcd for $C_{25}H_{31}N_2O_4S^+$ 455.1999; Found 455.1997.

Data for the minor diastereomer

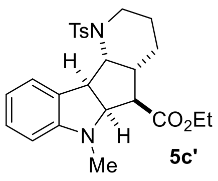

Chemical Formula: $C_{25}H_{30}N_{2}O_{4}S$
Molecular Weight: 454,58

Rf = 0.36 (pentane/EtOAc 4:1).
^{1}H NMR (400 MHz, $CDCl_3$): δ 7.78 – 7.72 (m, 2H, Ts), 7.70 (d, *J* = 7.5 Hz, 1H, Ar*H*), 7.37 – 7.34 (m, 2H, Ts), 7.09 (t, *J* = 7.5 Hz, 1H, Ar*H*), 6.71 (td, *J* = 7.5, 1.1 Hz, 1H, Ar*H*), 6.37 (dd, *J* = 7.9, 1.0 Hz, 1H, Ar*H*), 4.65 – 4.58 (m, 1H, C*H*), 4.26 – 4.05 (m, 3H, OCH_2CH_3, C*H*), 3.37 (ddd, *J* = 12.7, 8.2, 4.1 Hz, 1H, CH_2), 2.84 – 2.74 (m, 5H, C*H*, CH_3, CH_2), 2.47 – 2.39 (m, 4H, C*H*, CH_3), 2.21 – 2.08 (m, 1H, C*H*), 1.92 – 1.82 (m, 1H, CH_2), 1.44 – 1.24 (m, 5H, CH_3, CH_2), 0.71 – 0.59 (m, 1H, CH_2).
^{13}C NMR (101 MHz, $CDCl_3$): δ 171.0, 154.7, 144.0, 133.3, 129.8, 128.3, 128.2, 128.2, 127.3, 118.2, 107.3, 69.6, 63.2, 60.5, 54.5, 50.6, 45.9, 38.7, 37.6, 24.7, 21.7, 21.7, 14.4.
IR (ν_{max}, cm^{-1}) 3035 (w), 2938 (m), 1729 (s), 1603 (m), 1490 (m), 1308 (s), 1158 (s), 737 (s).
HRMS (ESI/QTOF) m/z: $[M + H]^+$ Calcd for $C_{25}H_{31}N_2O_4S^+$ 455.1999; Found 455.1993.

Ethyl-1-((*N*,4-dimethylphenyl)sulfonamido)-1,4-dimethyl-1,2,3,3a,4,8b-hexahydrocyclopenta[b]indole-3-carboxylate (5d)

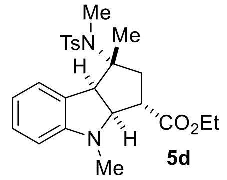

Chemical Formula: $C_{24}H_{30}N_2O_4S$
Molecular Weight: 442.5740

Prepared according to the general procedure E from Tf_2NH (0.23 M, 153 µL, 35.2 µmol, 20 mol%), silyl enol ether **3b** (19 mg, 88 µmol, 0.5 equiv.), aminocyclopropane **1j** (54.8 mg, 176 µmol, 1.0 equiv.) and 1-methylindole **2a** (23.8 µL, 185 µmol, 1.05 equiv.) in DCM (0.3 M, 0.6 mL) at rt for 1 h. The crude product was purified by PREP TLC using pent/Et_2O 70:30 to afford cycloadduct **5d** as a colorless oil (21 mg, 47 µmol, 27%, dr > 95:5).
Rf = 0.52 (pentane/EtOAc 4:1).
^{1}H NMR (400 MHz, $CDCl_3$): δ 7.78 – 7.71 (m, 2H, Ts), 7.30 (d, *J* = 8.0 Hz, 2H, Ts), 7.18 – 7.08 (m, 2H, Ar*H*), 6.65 (td, *J* = 7.4, 1.0 Hz, 1H, Ar*H*), 6.41 (d, *J* = 7.8 Hz, 1H, Ar*H*), 4.33 (d, *J* = 10.9 Hz, 1H, C*H*), 4.27 – 4.10 (m, 3H, C*H*, OCH_2CH$_3$), 3.03 (s, 3H, CH_3), 2.99 – 2.88 (m, 1H, C*H*), 2.77 (s, 3H, CH_3), 2.67 – 2.56 (m, 1H, CH_2), 2.48 (dd, *J* = 13.1, 7.2 Hz, 1H, CH_2), 2.42 (s, 3H, CH_3), 1.28 (t, *J* = 7.1 Hz, 3H, OCH$_2$CH_3), 1.19 (s, 3H, CH_3).
^{13}C NMR (101 MHz, $CDCl_3$): δ 174.2, 143.3, 138.9, 129.7, 128.8, 128.0, 127.4, 127.1, 126.3, 117.7, 107.1, 73.1, 71.3, 61.1, 55.6, 49.2, 43.5, 34.5, 34.2, 21.6, 21.6, 14.3.
IR (ν_{max}, cm^{-1}) 3037 (m), 2949 (m), 1726 (s), 1488 (s), 1313 (s), 1157 (s), 916 (s).
HRMS (ESI/QTOF) m/z: $[M + Na]^+$ Calcd for $C_{24}H_{30}N_2NaO_4S^+$ 465.1818; Found 465.1827.

Ethyl-1-((*N*,4-dimethylphenyl)sulfonamido)-3,4-dimethyl-1,2,3,3a,4,8b-hexahydrocyclopenta[b]indole-3-carboxylate (5e)

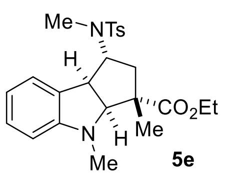

Chemical Formula: $C_{24}H_{30}N_2O_4S$
Molecular Weight: 442.5740

Prepared according to the general procedure E from Tf_2NH (0.212 M, 173 µL, 36.6 µmol, 20 mol%), silyl enol ether **3b** (19.8 mg, 91.5 µmol, 0.5 equiv.), aminocyclopropane **1k** (57 mg, 0.18 mmol, 1.0 equiv.) and 1-methylindole **2a** (24.7 µL, 192 µmol, 1.05 equiv.) in DCM (0.3 M, 0.6 mL). The crude product was purified by flash chromatography using pent/EtOAc 85:15 to afford cycloadduct **5e**

as a colorless oil and an inseparable mixture of diastereomers (62.8 mg, 142 μmol, 78%, dr 88:12).

Characterized as mixture of diastereomers

Rf = 0.44 (pentane/EtOAc 4:1).

^{1}H NMR (400 MHz, $CDCl_3$, signals for major diastereomer): δ 7.68 – 7.63 (m, 2H, Ts), 7.30 – 7.26 (m, 2H, Ts), 7.08 (t, *J* = 7.7 Hz, 1H, Ar*H*), 6.95 (d, *J* = 7.3 Hz, 1H, Ar*H*), 6.56 (td, *J* = 7.4, 1.0 Hz, 1H, Ar*H*), 6.36 (d, *J* = 8.0 Hz, 1H, Ar*H*), 4.40 (ddd, *J* = 12.3, 8.5, 6.1 Hz, 1H, C*H*), 4.21 – 4.12 (m, 3H, C*H*, OCH_2CH_3), 3.73 (dd, *J* = 11.5, 8.5 Hz, 1H, C*H*), 2.88 (s, 3H, CH_3), 2.84 (s, 3H, CH_3), 2.43 (s, 3H, CH_3), 2.08 (t, *J* = 12.4 Hz, 1H, CH_2), 1.31 – 1.24 (m, 4H, OCH_2CH_3, CH_2), 1.12 (s, 3H, CH_3).

^{13}C NMR (101 MHz, $CDCl_3$, signals for major diastereomer): δ 177.0, 152.1, 143.4, 136.5, 129.9, 129.0, 128.4, 127.3, 124.2, 117.3, 105.7, 73.8, 63.1, 61.2, 52.2, 47.9, 38.0, 34.8, 29.1, 21.6, 19.0, 14.3.

IR (ν_{max}, cm^{-1}) 3050 (m), 2936 (m), 1719 (s), 1492 (m), 1337 (s), 1159 (s), 1094 (s), 736 (s).

HRMS (ESI/QTOF) m/z: $[M + H]^+$ Calcd for $C_{24}H_{31}N_2O_4S^+$ 443.1999; Found 443.2003.

Ethyl-1-((*N*,4-dimethylphenyl)sulfonamido)-4-methyl-3-phenyl-1,2,3,3a,4,8b-hexahydrocyclopenta[b]indole-3-carboxylate (5f)

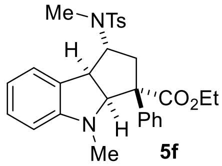

5f

Chemical Formula: $C_{29}H_{32}N_2O_4S$
Molecular Weight: 504.6450

Prepared according to the general procedure E from Tf_2NH (0.23 M, 146 μL, 33.5 μmol, 20 mol%), silyl enol ether **3b** (18.1 mg, 83.7 μmol, 0.5 equiv.), aminocyclopropane **1l** (62.5 mg, 167 μmol, 1.0 equiv.) and 1-methylindole **2a** (22.6 μL, 176 μmol, 1.05 equiv.) in DCM (0.3 M, 0.6 mL). The crude product was purified by flash chromatography using pent/EtOAc 85:15 and PREP TLC using pent/EtOAc 80:20 to afford cycloadduct **5f** (major diastereomer, 56 mg, 0.11 mmol, 66% and minor diastereomer 10 mg, 20 μmol, 12%, dr 80:20) as a colorless oil.

Data for major diastereomer

Rf = 0.38 (pentane/EtOAc 4:1).

^{1}H NMR (400 MHz, $CDCl_3$): δ 7.66 – 7.61 (m, 2H, Ts), 7.33 – 7.23 (m, 5H, Ts, Ar*H*), 7.23 – 7.18 (m, 2H, Ar*H*), 7.02 (td, *J* = 7.6, 1.2 Hz, 1H, Ar*H*), 6.77 (d, *J* = 7.3 Hz, 1H, Ar*H*), 6.54 (td, *J* = 7.4, 1.0 Hz, 1H, Ar*H*), 6.25 (d, *J* = 7.9 Hz, 1H, Ar*H*), 4.61 (d, *J* = 9.4 Hz, 1H, C*H*), 4.44 (q, *J* = 7.3 Hz, 1H, C*H*), 4.27 – 4.16 (m, 2H, OCH_2CH_3), 3.82 (dd, *J* = 9.4, 6.7 Hz, 1H, C*H*), 2.86 (s, 3H, CH_3), 2.45 (dd, *J* = 7.6, 2.2 Hz, 2H, CH_2), 2.42 (s, 3H, CH_3), 2.17 (s, 3H, CH_3), 1.24 (t, *J* = 7.1 Hz, 3H, OCH_2CH_3).

^{13}C NMR (101 MHz, $CDCl_3$): δ 176.0, 152.7, 143.5, 139.0, 135.9, 129.8, 129.7, 128.3, 128.2, 127.6, 127.5, 127.3, 123.7, 118.0, 107.6, 77.4, 64.9, 61.9, 61.7, 49.4, 38.8, 36.5, 30.3, 21.7, 14.1.

IR (ν_{max}, cm^{-1}) 3053 (m), 2932 (m), 1718 (m), 1491 (m), 1232 (m), 911 (m), 734 (s).

HRMS (ESI/QTOF) m/z: $[M + H]^+$ Calcd for $C_{29}H_{33}N_2O_4S^+$ 505.2156; Found 505.2163.

Data for minor diastereomer

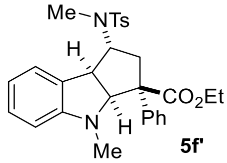

5f'

Chemical Formula: $C_{29}H_{32}N_2O_4S$
Molecular Weight: 504,64

Rf = 0.33 (pentane/EtOAc 4:1).

^{1}H NMR (400 MHz, $CDCl_3$): δ 7.76 – 7.72 (m, 2H, Ts), 7.38 – 7.23 (m, 7H, Ts, Ar*H*), 7.11 (td, *J* = 7.6, 1 Hz, 1H, Ar*H*), 7.02 – 6.98 (m, 1H, Ar*H*), 6.63 (td, *J* = 7.4, 1.0 Hz, 1H, Ar*H*), 6.46 (d, *J* = 7.9 Hz, 1H, Ar*H*), 4.92 (ddd, *J* = 11.9, 9.0, 6.1 Hz, 1H, C*H*), 4.57 (d, *J* = 10.9 Hz, 1H, C*H*), 4.02 (qd, *J* = 7.1, 1.8 Hz, 2H, OCH_2CH_3), 3.83

– 3.75 (m, 1H, C*H*), 2.94 (s, 3H, C*H*3), 2.82 (s, 3H, C*H*3), 2.44 – 2.37 (m, 4H, C*H*3, C*H*2), 1.83 – 1.74 (m, 1H, C*H*2), 1.09 (t, *J* = 7.1 Hz, 3H, OCH2C*H*3).
^{13}C NMR (101 MHz, $CDCl_3$): δ 172.9, 152.2, 143.4, 143.3, 136.7, 129.8, 129.6, 128.6, 128.3, 127.5, 127.2, 127.1, 124.2, 118.6, 107.9, 76.7, 63.9, 62.2, 61.3, 49.1, 40.3, 37.3, 29.0, 21.7, 14.0.
IR (ν_{max}, cm^{-1}) 3037 (m), 2926 (m), 1719 (m), 1492 (s), 1229 (s), 912 (s), 737 (s).
HRMS (ESI/QTOF) m/z: [M + H]$^+$ Calcd for $C_{29}H_{33}N_2O_4S^+$ 505.2156; Found 505.2175.

Ethyl-3-allyl-1-((*N*,4-dimethylphenyl)sulfonamido)-4-methyl-1,2,3,3a,4,8b-hexahydrocyclopenta[b]indole-3-carboxylate (5g)

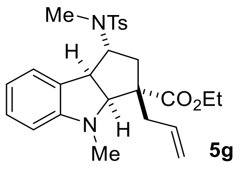

Chemical Formula: $C_{26}H_{32}N_2O_4S$
Molecular Weight: 468.6120

Prepared according to the general procedure E from Tf_2NH (0.23 M, 271 µL, 62.2 µmol, 20 mol%), silyl enol ether **3b** (33.7 mg, 156 µmol, 0.5 equiv.), aminocyclopropane **1m** (105 mg, 311 µmol, 1.0 equiv.) and 1-methylindole **2a** (42 µL, 0.33 mmol, 1.05 equiv.) in DCM (0.3 M, 1 mL). The crude product was purified by flash chromatography using pent/EtOAc 85:15 to afford cycloadduct **5g** as a white solid (126 mg, 269 µmol, 86%, dr 87:13).
Rf = 0.17 (pentane/EtOAc 9:1).
m. p. = 153.3 – 155 °C.
^{1}H NMR (400 MHz, $CDCl_3$): δ 7.66 – 7.61 (m, 2H, Ts), 7.28 – 7.22 (m, 2H, Ts), 7.14 – 7.08 (m, 2H, Ar*H*), 6.63 (td, *J* = 7.4, 1.0 Hz, 1H Ar*H*), 6.43 – 6.39 (m, 1H Ar*H*), 5.60 – 5.46 (m, 1H, C*H*), 5.10 – 5.01 (m, 2H, C*H*2), 4.33 (ddd, *J* = 12.7, 8.7, 5.9 Hz, 1H, C*H*), 4.18 (qd, *J* = 7.1, 0.9 Hz, 2H, OC*H*2CH3), 4.03 (d, *J* = 11.6 Hz, 1H, C*H*), 3.75 (dd, *J* = 11.6, 8.7 Hz, 1H, C*H*), 2.91 (s, 3H, C*H*3), 2.88 (s, 3H, C*H*3), 2.65 – 2.55 (m, 1H, C*H*2), 2.42 (s, 3H, C*H*3), 2.04 – 1.93 (m, 2H, C*H*2), 1.40 (dd, *J* = 12.9, 5.9 Hz, 1H, C*H*2), 1.29 (t, *J* = 7.1 Hz, 3H, OCH2C*H*3).
^{13}C NMR (101 MHz, $CDCl_3$): δ 175.9, 152.5, 143.4, 136.3, 134.0, 129.8, 129.1, 128.5, 127.3, 124.4, 118.6, 117.8, 106.3, 74.8, 62.5, 61.3, 56.2, 48.0, 35.8, 35.7, 32.4, 29.1, 21.6, 14.4.
IR (ν_{max}, cm^{-1}) 3050 (m), 2919 (m), 1719 (m), 1493 (m), 1339 (s), 1213 (s), 912 (s), 737 (s).
HRMS (ESI/QTOF) m/z: [M + H]$^+$ Calcd for $C_{26}H_{33}N_2O_4S^+$ 469.2156; Found 469.2167.

Ethyl-6-methyl-3-tosyl-1,2,3,3a,4,5,5a,6-octahydropyrrolo[3',2':2,3]cyclopenta[1,2-b]indole-5-carboxylate (5h)

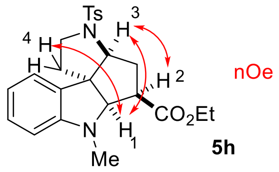

Chemical Formula: $C_{24}H_{28}N_2O_4S$
Molecular Weight: 440.5580

Prepared according to the general procedure E from Tf_2NH (0.227 M, 25 µL, 5.7 µmol, 5.0 mol%), silyl enol ether **3b** (6.2 mg, 29 µmol, 0.25 equiv.) and aminocyclopropane **1n** (50.4 mg, 114 µmol, 1.0 equiv.) in DCM (0.1 M, 1 mL) for 2 h at -78 °C. The crude product was purified by PREP TLC using tol/EtOAc 95:5 to afford cycloadduct **5h** as a colorless oil (29.8 mg, 67.6 µmol, 59%, dr > 95:5).
Rf = 0.29 (toluene/EtOAc 95:5).
^{1}H NMR (400 MHz, $CDCl_3$): δ 7.75 – 7.69 (m, 2H, Ts), 7.35 – 7.29 (m, 2H, Ts), 7.10 (td, *J* = 7.7, 1.3 Hz, 1H, Ar*H*), 7.04 (dd, *J* = 7.3, 1.3 Hz, 1H, Ar*H*), 6.70 (td, *J* = 7.4, 1.0 Hz, 1H, Ar*H*), 6.50 (d, *J* = 7.9 Hz, 1H, Ar*H*), 4.78 (d, *J* = 4.3 Hz, 1H, H-3, C*H*), 3.96 (dd, *J* = 12.9, 6.4 Hz, 1H, C*H*2), 3.58 (dq, *J* = 10.8, 7.1 Hz, 1H, OC*H*2CH3), 3.18 (dq, *J* = 10.8, 7.1 Hz, 1H, OC*H*2CH3), 3.04 (s, 1H, H-1, C*H*), 2.93 (td, *J* = 12.3, 4.8 Hz, 1H, C*H*2), 2.69 (s, 3H, C*H*3), 2.68 – 2.54 (m, 2H, H-2 C*H*, C*H*2), 2.43 (s, 3H, C*H*3), 2.22 (dd, *J* = 12.8, 4.4 Hz, H-4, 1H, C*H*2), 2.02 (td, *J* = 12.2, 6.4 Hz, 1H, H-4, C*H*2), 1.55 – 1.44 (m, 1H, C*H*2), 0.88 (t, *J* = 7.1 Hz, 3H, OCH2C*H*3).

^{13}C NMR (101 MHz, $CDCl_3$): δ 172.5, 153.1, 143.7, 136.3, 130.9, 130.0, 128.8, 127.3, 123.9, 118.2, 108.9, 84.7, 60.5, 59.6, 56.5, 51.2, 39.9, 34.6, 32.7, 32.3, 21.7, 13.8.
IR (ν_{max}, cm^{-1}) 3052 (m), 2947 (m), 1736 (s), 1469 (s), 1342 (s), 1161 (s), 911 (s), 728 (s).
HRMS (nanochip-ESI/LTQ-Orbitrap) m/z: $[M + H]^+$ Calcd for $C_{24}H_{29}N_2O_4S^+$ 441.1843; Found 441.1843.

4.3. List of unsuccessful substrates

The Scheme S7 presents the uncuccessful partners attempted with aminocyclopropane **1d** using general procedure E.

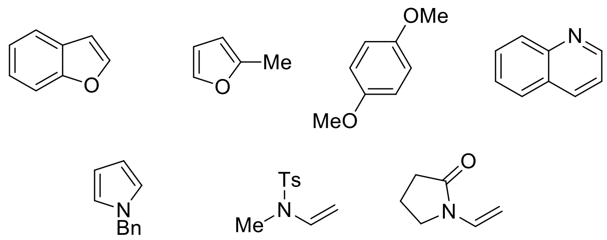

Scheme S7. List of unsuccessful substrates in the (3+2) annulation.

5. Product modifications

Characterization of product 6-8

***N*-(4-Benzyl-3-(hydroxymethyl)-1,2,3,3a,4,8b-hexahydrocyclopenta[b]indol-1-yl)-*N*,4-dimethylbenzenesulfonamide (31)**

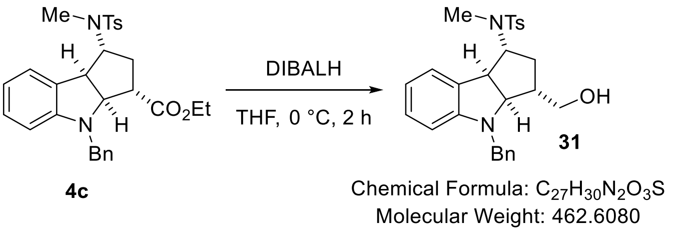

In a 10 mL RBF, the cycloadduct **4c** (203 mg, 402 µmol, 1.0 equiv.) was diluted in dry THF (4 mL) and the mixture was cooled to 0 °C. Diisobutylaluminium hydride (1.2 M, 670 µL, 805 µmol, 2.0 equiv.) was added dropwise and the reaction mixture was stirred for 2 h. The reaction was quenched with a saturated $NaHCO_3$ aqueous solution and the aqueous layer was extracted with EtOAc. The combined organic layers were washed with brine, dried over Na_2SO_4, filtered off and concentrated under reduced pressure. The crude product was purified by flash chromatography using pent/EtOAc 60:40 to afford alcohol **31** as a white solid (153 mg, 331 µmol, 82%).

Rf = 0.23 (pentane/EtOAc 3:2).
m. p. = 62 – 64 °C.
^{1}H NMR (400 MHz, $CDCl_3$): δ 7.66 – 7.60 (m, 2H, Ts), 7.34 – 7.21 (m, 7H, Ts, Ar*H*), 7.02 (td, *J* = 7.7, 1.3 Hz, 1H, Ar*H*), 6.89 (d, *J* = 7.3 Hz, 1H, Ar*H*), 6.54 (td, *J* = 7.4, 0.9 Hz, 1H, Ar*H*), 6.35 (d, *J* = 7.7 Hz, 1H, Ar*H*), 4.51 – 4.29 (m, 3H, C*H*, C*H*$_2$), 3.87 (dd, *J* = 10.3, 4.8 Hz, 1H, C*H*), 3.59 – 3.43 (m, 3H, C*H*, C*H*$_2$), 2.88 (s, 3H, C*H*$_3$), 2.41 (s, 3H, C*H*$_3$), 2.28 – 2.16 (m, 1H, C*H*), 1.65 – 1.57 (m, 1H, C*H*$_2$), 1.48 – 1.35 (m, 2H, C*H*$_2$, OH).
^{13}C NMR (101 MHz, $CDCl_3$): δ 151.2, 143.2, 139.0, 136.7, 129.8, 129.4, 128.7, 128.3, 127.4, 127.3, 127.1, 124.4, 117.7, 107.0, 70.8, 65.1, 64.1, 51.8, 48.4, 46.9, 30.7, 29.1, 21.6.

IR (ν_{max}, cm^{-1}) 3542 (w), 3055 (w), 2942 (w), 1603 (m), 1487 (s), 1334 (s), 1157 (s), 738 (s).
HRMS (ESI/QTOF) m/z: $[M + H]^+$ Calcd for $C_{27}H_{31}N_2O_3S^+$ 463.2050; Found 463.2046.

4-Benzyl-1-(methylamino)-1,2,3,3a,4,8b-hexahydrocyclopenta[b]indol-3-yl)methanol (6)

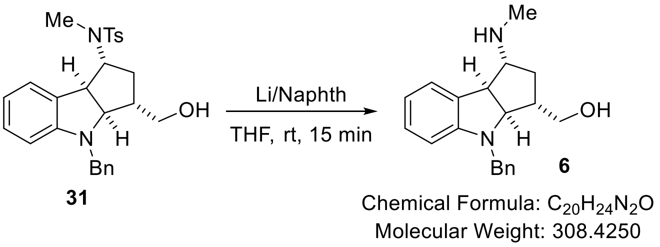

A Li/naphthalene solution in THF (0.5 M, 4.4 mL, 8.0 equiv.) was added dropwise at rt to alcohol **31** (127 mg, 275 µmol, 1.0 equiv.) diluted in THF (3 mL) under a vigorous stirring. After the addition (full conversion was observed), the reaction was quenched with a 1 M HCl aqueous solution. The aqueous layer was washed with DCM, then basified to pH>10, and finally extracted with DCM. The combined organic layers were washed with brine, dried over Na_2SO_4, filtered off and concentrated under reduced pressure. The crude product was purified by flash chromatography using DCM/MeOH 90:10 to afford amino alcohol **6** as a viscous oil (50.1 mg, 162 µmol, 59%).

Rf = 0.65 (DCM/MeOH 9:1).
^{1}H NMR (400 MHz, MeOD): δ 7.33 – 7.26 (m, 4H, Ar*H*), 7.26 – 7.19 (m, 1H, Ar*H*), 7.15 (d, *J* = 7.3 Hz, 1H, Ar*H*), 6.99 (t, *J* = 7.7 Hz, 1H, Ar*H*), 6.61 (t, *J* = 7.4 Hz, 1H, Ar*H*), 6.36 (d, *J* = 7.9 Hz, 1H, Ar*H*), 4.48 (d, *J* = 15.9 Hz, 1H, C*H*$_2$), 4.31 (d, *J* = 15.9 Hz, 1H, C*H*$_2$), 4.10 (d, *J* = 8.9 Hz, 1H, C*H*), 3.68 – 3.61 (m, 1H, C*H*), 3.60 – 3.48 (m, 2H, C*H*$_2$), 3.29 – 3.24 (m, 1H, C*H*), 2.61 (s, 3H, C*H*$_3$), 2.41 – 2.33 (m, 1H, C*H*), 2.31 – 2.21 (m, 1H, C*H*$_2$), 1.73 – 1.65 (m, 1H, C*H*$_2$).
^{13}C NMR (101 MHz, $CDCl_3$): δ 151.9, 138.8, 129.9, 128.7, 128.5, 127.3, 127.2, 124.9, 117.5, 106.5, 74.0, 69.0, 64.8, 53.6, 52.0, 46.6, 34.8, 33.1.
IR (ν_{max}, cm^{-1}) 3392 (w), 3054 (m), 2943 (w), 1602 (m), 1487 (s), 1353 (m), 741 (s).
HRMS (ESI/QTOF) m/z: $[M + H]^+$ Calcd for $C_{20}H_{25}N_2O^+$ 309.1961; Found 309.1958.

5-Benzyl-2-methyl-1,2,4,4a,5,9b-hexahydro-3H-1,4-methanopyrido[4,3-b]indol-3-one (7)

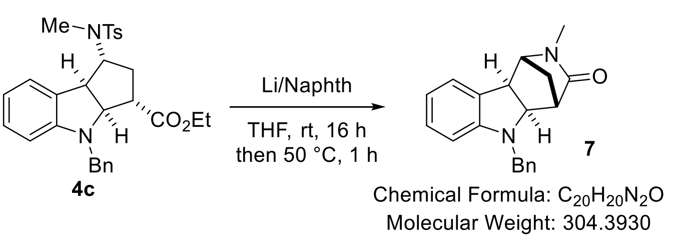

A 0.5 M Li/naphthalene solution in THF was prepared by adding Li sticks to naphthalene diluted in dry THF. The heterogeneous solution was sonicated for 1 h at rt or until an intense dark/green color appeared. This Li/naphthalene solution (0.5 M, 3.1 mL, 8.0 equiv.) was added dropwise at rt to the cycloadduct **4c** (98.4 mg, 195 µmol, 1.0 equiv.) diluted in THF. The mixture was then stirred at rt for 16 h and at 50 °C for 1 h. The reaction was then diluted with DCM (5 mL) and quenched with a 1 M HCl aqueous solution (10 mL). The organic layer was extracted with DCM (3x 15mL) and the combined organic layers were washed with a 1 M HCl aqueous solution and brine, dried over Na_2SO_4, filtered off and concentrated under reduced pressure. The crude product was purified by flash chromatography using pent/EtOAc 50:50 to afford lactam **7** as a yellow oil (25.8 mg, 84.8 µmol, 43%).

Rf = 0.18 (pentane/EtOAc 3:2).
^{1}H NMR (400 MHz, $CDCl_3$): δ 7.34 – 7.20 (m, 5H, Ar*H*), 7.09 – 7.00 (m, 2H, Ar*H*), 6.62 (td, *J* = 7.4, 0.9 Hz, 1H, Ar*H*), 6.38 (d, *J* = 7.8 Hz, 1H, Ar*H*), 4.51 – 4.36 (m, 2H, C*H*$_2$), 4.14 (dt, *J*

= 8.3, 1.2 Hz, 1H, C*H*), 3.83 (d, *J* = 8.3 Hz, 1H, C*H*), 3.74 (s, 1H, C*H*), 2.85 (s, 1H, C*H*), 2.82 (s, 3H, CH_3), 1.87 – 1.78 (m, 2H, CH_2).
^{13}C NMR (101 MHz, $CDCl_3$): δ 176.2, 154.5, 138.5, 128.8, 128.8, 127.4, 127.2, 126.4, 124.3, 117.0, 106.0, 68.6, 66.3, 51.7, 51.3, 50.1, 34.1, 28.2.
IR (ν_{max}, cm^{-1}) 3054 (m), 2929 (m), 1697 (s), 1491 (s), 1319 (m), 962 (m), 915 (m), 738 (s).
HRMS (ESI/QTOF) m/z: $[M + Na]^+$ Calcd for $C_{20}H_{20}N_2NaO^+$ 327.1468; Found 327.1470.

Ethyl-1-((*N*,4-dimethylphenyl)sulfonamido)-1,2,3,3a,4,8b-hexahydrocyclopenta[b]indole-3-carboxylate (8)

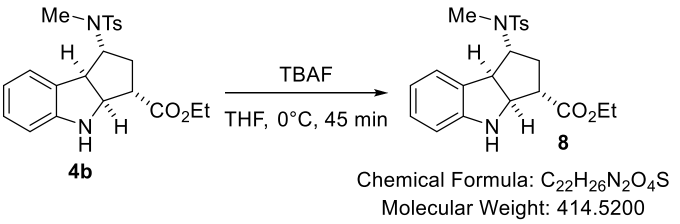

The cycloadduct **4b** (106 mg, 200 µmol, 1.0 equiv.) was diluted in dry THF (2 mL) and TBAF (1 M, 240 µL, 240 µmol, 1.2 equiv.) was added dropwise at 0 °C. The reaction mixture was stirred for 45 min at 0 °C and was quenched with a saturated $NaHCO_3$ aqueous solution. The aqueous layer was extracted with DCM and the combined organic layers were washed with brine, dried over Na_2SO_4, filtered off and concentrated under reduced pressure. The crude product was purified by flash chromatography using pent/EtOAc 65:35 to afford the free indole **8** as a colorless oil (68.1 mg, 164 µmol, 82%).

Rf = 0.3 (pentane/EtOAc 4:1).
^{1}H NMR (400 MHz, $CDCl_3$): δ 7.70 – 7.66 (m, 2H, Ts), 7.30 – 7.27 (m, 2H, Ts), 7.15 (d, *J* = 7.5 Hz, 1H, Ar*H*), 7.06 (t, *J* = 7.6 Hz, 1H, Ar*H*), 6.72 (t, *J* = 7.4 Hz, 1H, Ar*H*), 6.62 (d, *J* = 7.8 Hz, 1H, Ar*H*), 4.51 (dt, *J* = 11.0, 6.6 Hz, 1H, C*H*), 4.26 (brs, 1H, N*H*), 4.22 – 4.12 (m, 3H, OCH_2CH_3, C*H*), 3.66 (dd, *J* = 10.2, 6.3 Hz, 1H, C*H*), 2.89 (s, 3H, CH_3), 2.62 (m, 1H, C*H*), 2.42 (s, 3H, CH_3), 1.83 – 1.72 (m, 1H, CH_2), 1.70 – 1.60 (m, 1H, CH_2), 1.27 (t, *J* = 7.1 Hz, 3H, OCH_2CH_3).
^{13}C NMR (101 MHz, $CDCl_3$): δ 173.7, 148.7, 143.5, 136.5, 129.9, 129.4, 128.4, 127.3, 125.1, 119.7, 110.0, 65.2, 63.8, 61.2, 50.6, 49.6, 29.5, 29.3, 21.7, 14.4.
IR (ν_{max}, cm^{-1}) 3388 (m), 3021 (w), 2939 (m), 1728 (s), 1476 (m), 1334 (s), 1161 (s), 738 (s).
HRMS (ESI/QTOF) m/z: $[M + H]^+$ Calcd for $C_{22}H_{27}N_2O_4S^+$ 415.1686; Found 415.1689.

6. Enantiospecific experiment and chiral HPLC traces

The enantioenriched tosyl-protected aminocyclopropane ***ent*-1d** was prepared using the enantioselective cyclopropanation described by Iwasa and co-workers.[24]

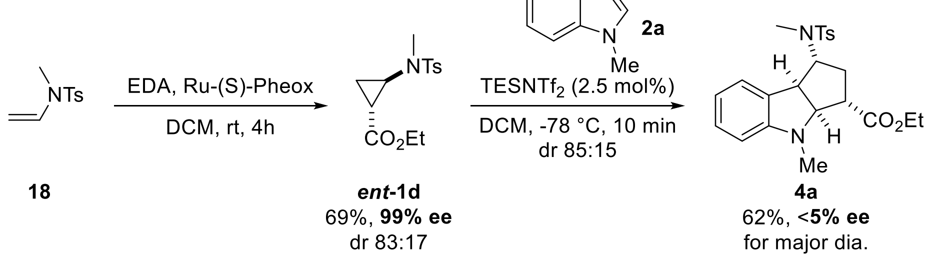

Scheme S8. Enantioselective cyclopropanation and enantiospecific experiment.

Enantioselective cyclopropanation

A 10 ml two-necked RBF was charged with Ru(II)-(S)-Pheox complex Ru-Pheox (1.3 mg, 2.0 µmol, 1 mol%) in a glovebox. Under a nitrogen atmosphere, the vinyl sulfonamide **18** (211 mg, 1.00 mmol, 5.0 equiv.) diluted in dry DCM (0.5 mL) was introduced. EDA (87%, 24.2 µL, 200 µmol, 1.0 equiv.) diluted in DCM (1.5 mL) was introduced via a syringe pump over a period of 3-4 h (~0.5 mL/h) at rt. After evaporation of the solvent, the crude was purified by flash chromatography using pent/EtOAc using pent/EtOAc 85:15 furnishing the *trans* product *ent*-**1d** (41 mg, 0.14 mmol, 69%, 97% ee, dr 83:17). The *cis* isomer was not isolated on this scale.

Enantiospecific experiment

According to the general procedure E from Tf_2NH (0.227 M, 8.52 µL, 1.93 µmol, 2.5 mol%), silyl enol ether **3b** (4.18 mg, 19.3 µmol, 0.25 equiv.), enantioenriched aminocyclopropane ***ent*-1d** (23 mg, 77 µmol, 1 equiv.) and 1-methylindole **2a** (10.4 µL, 81.2 µmol, 1.05 equiv.) in DCM (0.1 M, 0.8 mL). The crude product was purified by PREP TLC using pent/EtOAc 80:20 to afford cycloadduct **4a** as a colorless oil (major diastereomer: 20.4 mg, 47.6 µmol, 62%, <5% ee). The minor isomer was not isolated.

HPLC traces

Daicel Chiralpak IA column: 90:10 hexane/IPA, flow rate 1 mL/min, λ = 230 nm

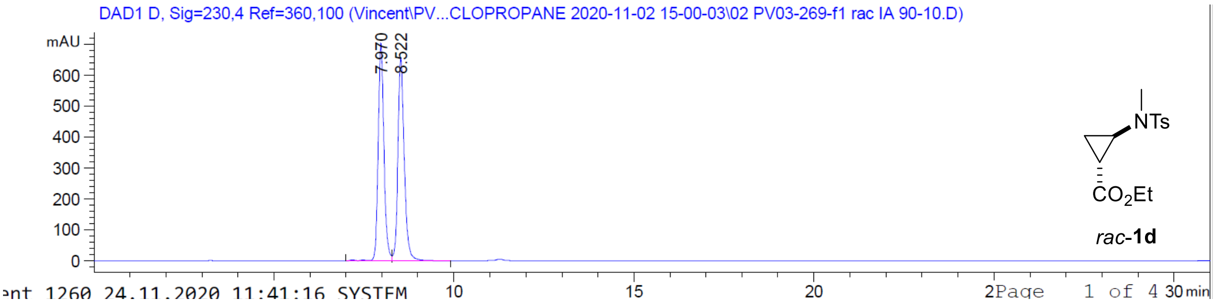

Peak #	RetTime [min]	Type	Width [min]	Area [mAU*s]	Height [mAU]	Area %
1	7.970	VV R	0.1666	7807.34717	703.13269	49.3676
2	8.522	VB	0.1849	8007.36426	653.51941	50.6324

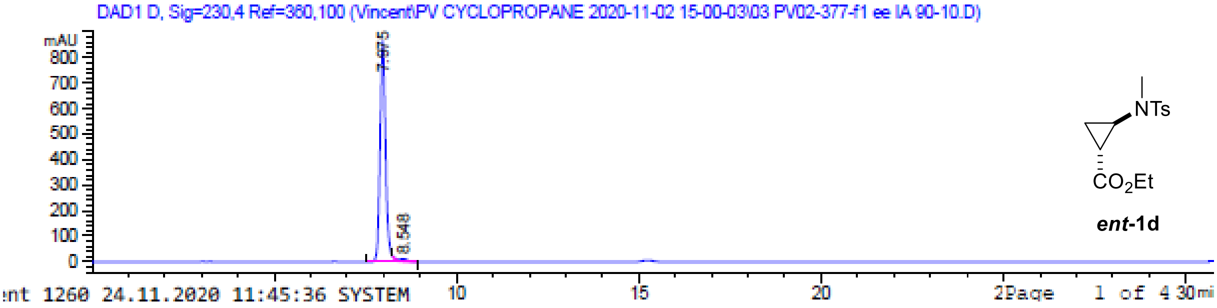

Peak #	RetTime [min]	Type	Width [min]	Area [mAU*s]	Height [mAU]	Area %
1	7.975	BV R	0.1695	9584.33496	863.37537	98.4002
2	8.548	VB E	0.2227	155.81909	9.95064	1.5998

Daicel Chiralpak IA column: 80:20 hexane/IPA, flow rate 1 mL/min, λ = 254 nm

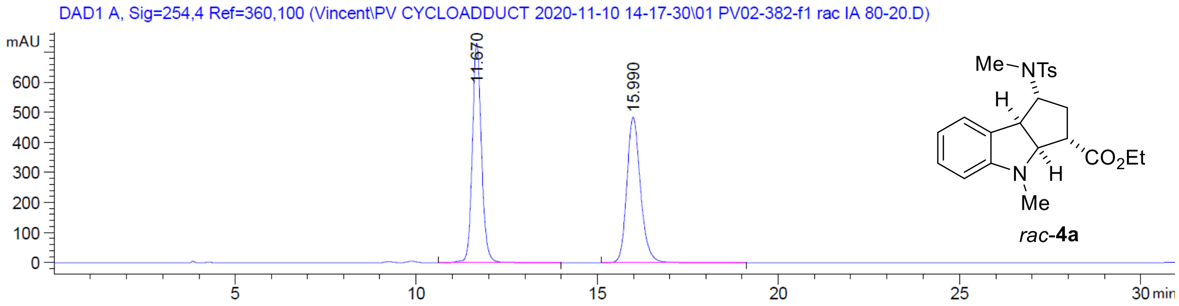

Peak #	RetTime [min]	Type	Width [min]	Area [mAU*s]	Height [mAU]	Area %
1	11.670	BB	0.2692	1.28307e4	729.47156	50.2810
2	15.990	BB	0.4001	1.26873e4	483.62991	49.7190

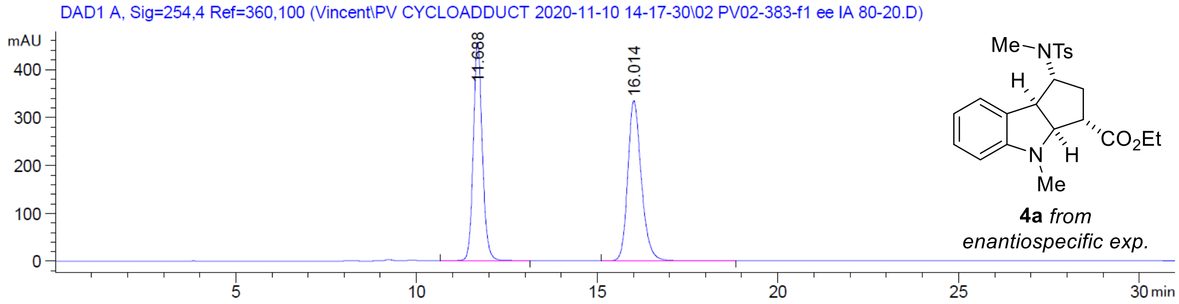

Peak #	RetTime [min]	Type	Width [min]	Area [mAU*s]	Height [mAU]	Area %
1	11.688	BB	0.2653	7936.32617	455.44235	47.5916
2	16.014	BB	0.3973	8739.58691	334.00238	52.4084

7. X-ray crystallographic data

7.1. Compound 4o

The crystal suitable for X-ray-measurement for compound **4o** was obtained by evaporation from EtOAc.

Experimental. Single clear intense yellow prism crystals of **4o** were used as supplied. A suitable crystal with dimensions 0.54 × 0.23 × 0.13 mm^3 was selected and mounted on a SuperNova, Dual, Cu at home/near, AtlasS2 diffractometer. The crystal was kept at a steady T = 140.00(10) K during data collection. The structure was solved with the ShelXT (Sheldrick, 2015) solution program using dual methods and by using Olex2 (Dolomanov et al., 2009) as the graphical interface. The model was refined with ShelXL 2018/3 (Sheldrick, 2015) using full matrix least squares minimisation on $\boldsymbol{F}^2$.

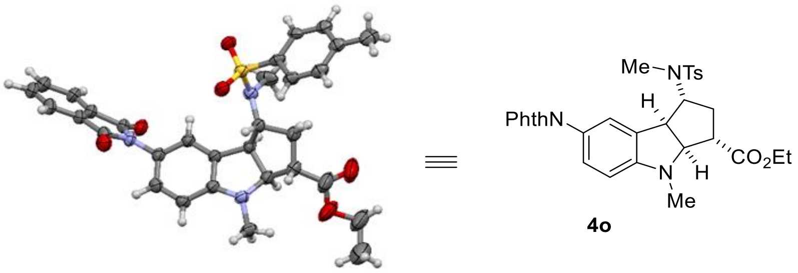

Figure S2. X-ray structure for compound **4o** (CCDC number: 2054864).

**Compound**	**4o**
Formula	$C_{31}H_{31}N_3O_6S$
$D_{calc.}$/ g cm^{-3}	1.227
μ/mm^{-1}	1.304
Formula Weight	573.65
Colour	clear intense yellow
Shape	prism
Size/mm^3	0.54×0.23×0.13
T/K	140.00(10)
Crystal System	monoclinic
Space Group	$P2_1/n$
a/Å	20.0965(8)
b/Å	5.88104(18)
c/Å	26.5867(10)
α/°	90
β/°	98.870(4)
γ/°	90
V/Å^3	3104.65(19)
Z	4
Z'	1
Wavelength/Å	1.54184
Radiation type	Cu K_α
Θ_{min}/°	3.365
Θ_{max}/°	76.763
Measured Refl's.	32676
Indep't Refl's	6437
Refl's I≥2 σ(I)	6031
R_{int}	0.0311
Parameters	542
Restraints	731
Largest Peak	0.627
Deepest Hole	-0.615
GooF	1.074
wR_2 (all data)	0.1849
wR_2	0.1829
R_1 (all data)	0.0780
R_1	0.0753

7.2. Compound 5g

The crystal suitable for X-ray-measurement for compound **5g** was obtained by diffusion (pentane/EtOAc).

Experimental. Single clear pale colourless needle crystals of **5g** were used as supplied. A suitable crystal with dimensions 0.83 × 0.05 × 0.03 mm^3 was selected and mounted on a SuperNova, Dual, Cu at home/near, Atlas diffractometer. The crystal was kept at a steady T = 140.01(10) K during data collection. The structure was solved with the ShelXT (Sheldrick, 2015) solution program using dual methods and by using Olex2 (Dolomanov et al., 2009) as the graphical interface. The model was refined with ShelXL 2018/3 (Sheldrick, 2015) using full matrix least squares minimisation on $\boldsymbol{F}^2$.

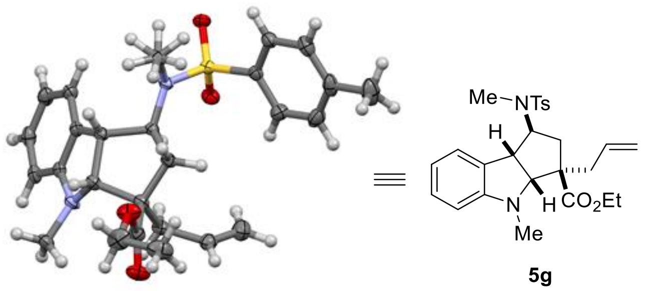

5g

Figure S3. X-ray structure for compound **5g** (CCDC number: 2054865).

**Compound**	**5g**
Formula	$C_{26}H_{32}N_2O_4S$
$D_{calc.}$/ g cm^{-3}	1.307
μ/mm^{-1}	1.494
Formula Weight	468.59
Colour	clear pale colourless
Shape	needle
Size/mm^3	0.83×0.05×0.03
T/K	140.01(10)
Crystal System	monoclinic
Space Group	$P2_1/c$
a/Å	6.36120(9)
b/Å	23.6708(4)
c/Å	15.8181(2)
α/°	90
β/°	91.6343(14)
γ/°	90
V/Å^3	2380.84(7)
Z	4
Z'	1
Wavelength/Å	1.54184
Radiation type	Cu K_α
Θ_{min}/°	3.361
Θ_{max}/°	72.575
Measured Refl's.	20793
Indep't Refl's	4656
Refl's I≥2 σ(I)	4063
R_{int}	0.0278
Parameters	303
Restraints	0
Largest Peak	0.459
Deepest Hole	-0.306
GooF	1.032
wR_2 (all data)	0.0928
wR_2	0.0880
R_1 (all data)	0.0414
R_1	0.0347

8. References

1. G.M. Sheldrick, SHELXT – Integrated space-group and crystal-structure determination. *Acta Cryst A* **2015**, *71*, 3–8.
2. G.M. Sheldrick, Crystal structure refinement with SHELXL. *Acta Cryst C* **2015**, *71*, 3–8.
3. T.Gatzenmeier, P. S. J. Kaib, J. B. Lingnau, R. Goddard, B. List, *Angew. Chem. Int. Ed*. **2018**, *57*, 2464–2468.
4. D. Perrotta, M.-M. Wang, J. Waser, *Angew. Chem. Int. Ed*. **2018**, *57*, 5120–5123.
5. K. Nemoto, S. Tanaka, M. Konno, S. Onozawa, M. Chiba, Y. Tanaka, Y. Sasaki, R. Okubo, T. Hattori, *Tetrahedron*, **2016**, *72*, 734–745.
6. U. Yadav, A. P. Sakla, R. Tokala, S. T. Nyalam, A. Khurana, C. S. Digwal, V. Talla, C. Godugu, N. Shankaraiah, A. Kamal, *ChemistrySelect*, **2020**, *5*, 4356–4363.
7. The iodide was prepared following a reported procedure: K. Speck, R. Wildermuth, T. Magauer, *Angew. Chem. Int. Ed*. **2016**, *55*, 14131–14135.
8. C. Bressy, D. Alberico, M. Lautens, *J. Am. Chem. Soc.*, **2005**, *127*, 13148–13149.
9. L. M. Repka, J. Ni, S. E. Reisman, *J. Am. Chem. Soc.*, **2010**, *132*, 14418–14420; M. C. DiPoto, R. P. Hughes, J. Wu, *J. Am. Chem. Soc.*, **2015**, *137*,14861–14864.
10. Y. Liao, Q. Lu, G. Chen, Y. Yu, C. Li, X. Huang, *ACS Catal.*, 2017, 7, 7529–7534.
11. T. R. Pradhan, H. W. Kim, J. K. Park, *Org. Lett.*, **2018**, *20*, 5286–5290.
12. J. Berges, B. Garcia, K. Muniz, *Angew. Chem. Int. Ed*. **2018**, *57*, 15891–15895.
13. X.-H. Xu, G.-K. Liu, A. Azuma, E. Tokunaga, N. Shibata, *Org. Lett.*, 2011, 13, 4854–4857.
14. B. S. Lane, M. A. Brown, D. Sames, *J. Am. Chem. Soc.*, **2005**, *127*, 8050–8057.
15. T.-H. Ding, J.-P. Qu, Y.-B. Kang, *Org. Lett.* **2020**, *22*, 3084–3088.
16. T. Stahl, K. Muether, Y. Ohki, K. Tatsumi, M. Oestreich, *J. Am. Chem. Soc.* **2013**, *135*, 10978–10981.
17. C. A. Sperger, J. E. Tungen, A. Fiksdahl, *Eur. J. Org. Chem.*, **2011**, 3719–3722.
18. Prepared following a reported procedure: L. Andna, L. Miesch, *Org. Biomol. Chem.*, **2019**, *17*, 5688–5692.
19. a) S. S. Kinderman, J. H. van Maarseveen, H. E. Schoemaker, H. Hiemstra, F. P. J. T. Rutjes, *Org. Lett.*, **2001**, *3*, 2045–2048; b) J. B. Feltenberger, R. Hayashi, Y. Tang, E. S. C. Babiash, R. P. Hsung, *Org. Lett.*, **2009**, *11*, 3666–3669.
20. J. B. Feltenberger, R. Hayashi, Y. Tang, E. S. C. Babiash, R. P. Hsung, *Org. Lett.*, **2009**, *11*, 3666–3669.
21. J. Zhu, Y.-J. Cheng, X.-K. Kuang, L. Wang, Z.-B. Zheng, Y. Tang, *Angew. Chem. Int. Ed*. **2016**, *55*, 9224–9228.
22. F. de Nanteuil, J. Waser, *Angew. Chem. Int. Ed.* **2011**, *50*, 12075–12079.
23. The diazo was prepared following a reported procedure: J. Yang, P. Ruan, W. Yang, X. Feng, X. Liu, *Chem. Sci*., **2019**, *10*, 10305–10309.
24. S. Chanthamath, D. T. Nguyen, K. Shibatomi, S. Iwasa, *Org. Lett*., **2013**, *15*, 772–775.

9. NMR spectra

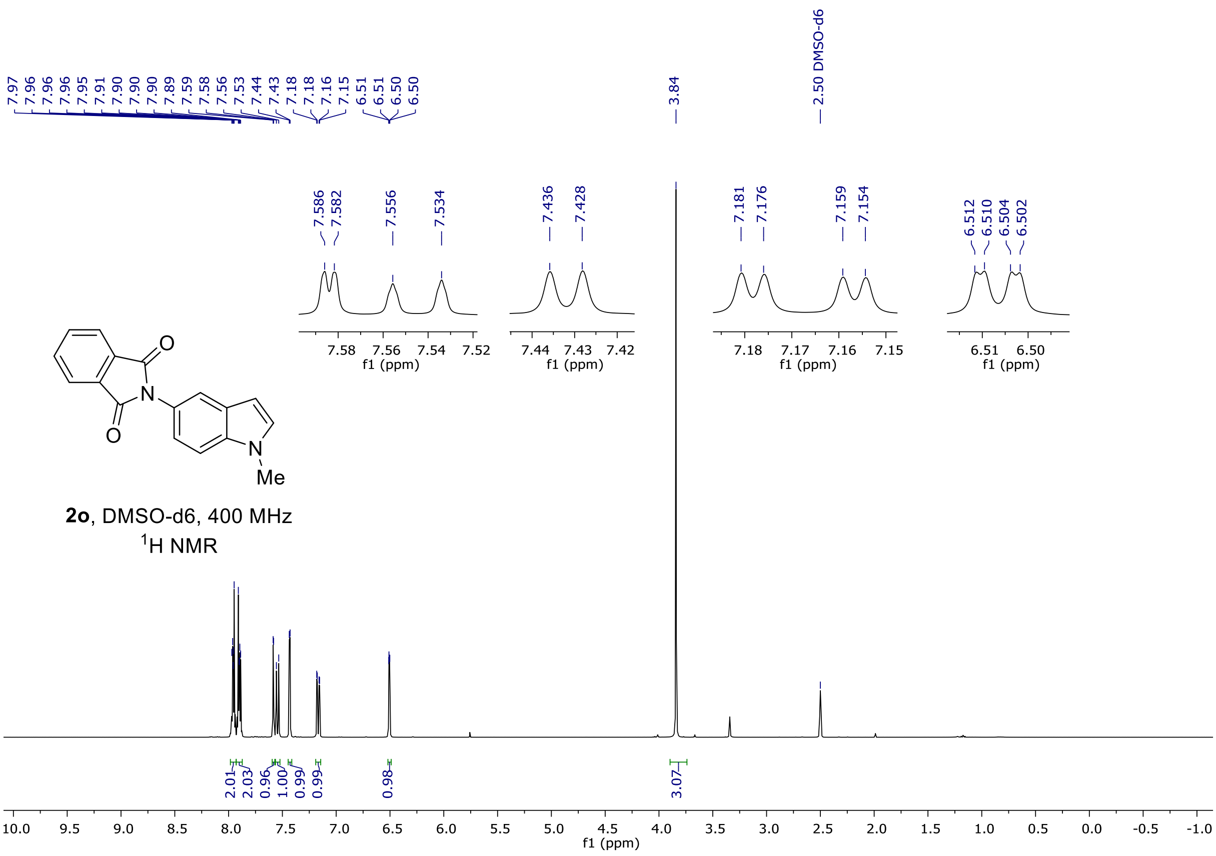

2o, DMSO-d6, 400 MHz
^{1}H NMR

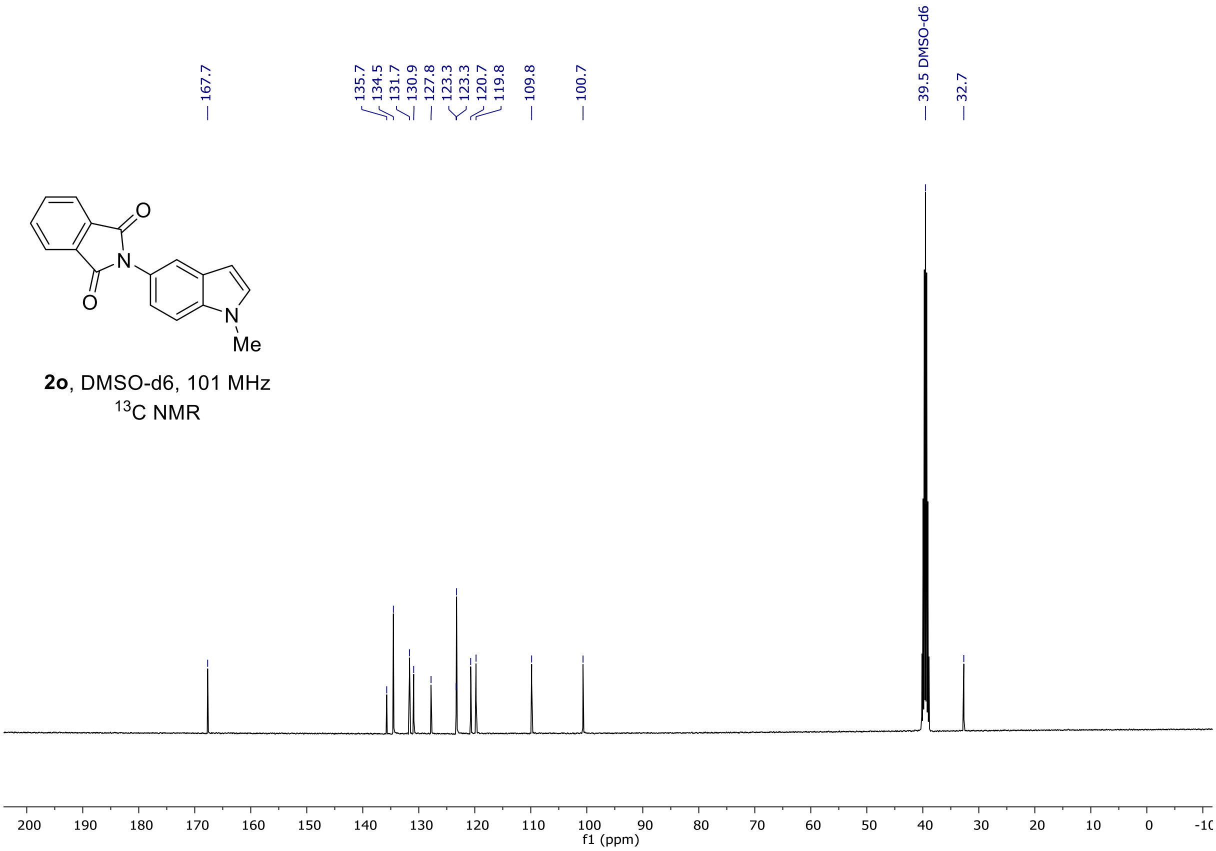

2o, DMSO-d6, 101 MHz
^{13}C NMR

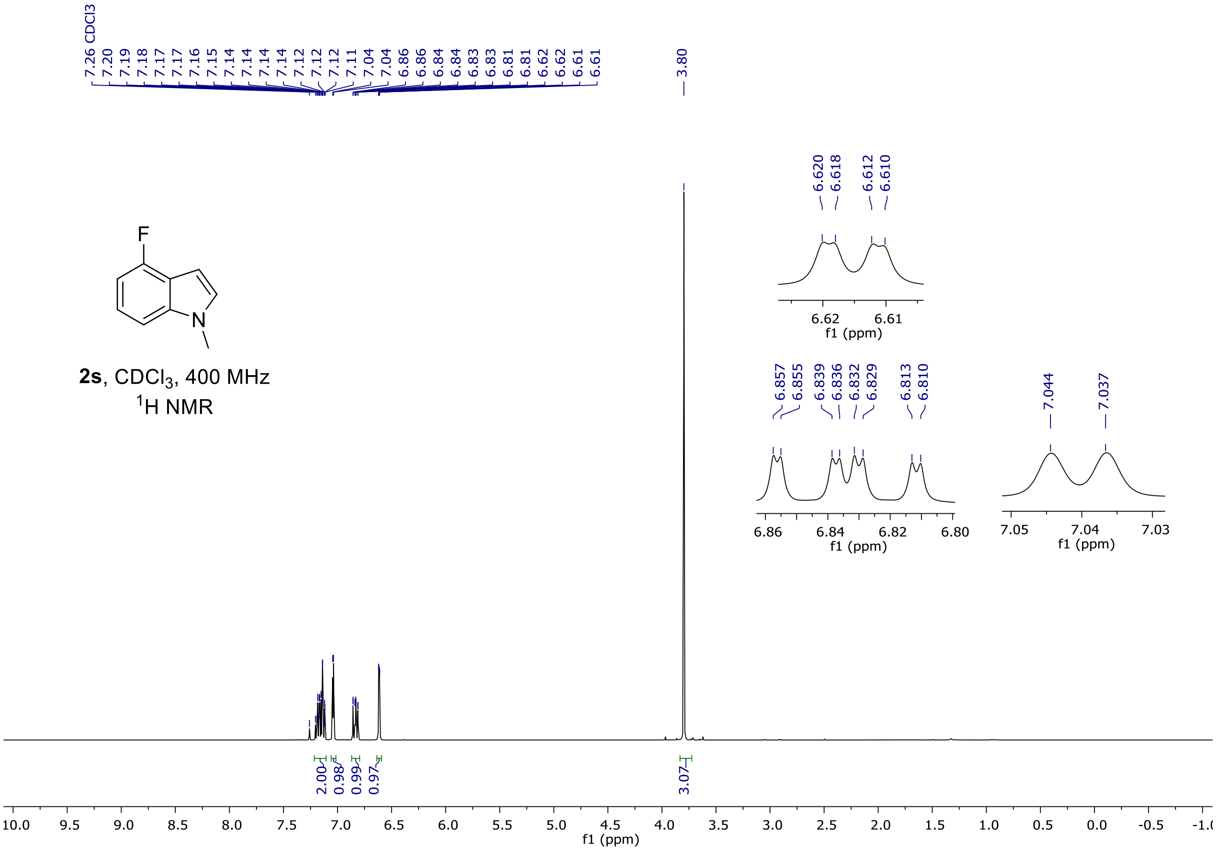
7.26 CDCl3
7.20
7.19
7.18
7.17
7.17
7.16
7.15
7.14
7.14
7.14
7.14
7.12
7.12
7.12
7.11
7.04
7.04
6.86
6.86
6.84
6.84
6.83
6.83
6.81
6.81
6.62
6.62
6.61
6.61
3.80
F
N
2s, CDCl3, 400 MHz
1H NMR
6.620
6.618
6.612
6.610
6.62
6.61
f1 (ppm)
6.857
6.855
6.839
6.836
6.832
6.829
6.813
6.810
6.86
6.84
6.82
6.80
f1 (ppm)
7.044
7.037
7.05
7.04
7.03
f1 (ppm)
2.00
0.98
0.99
0.97
3.07
10.0
9.5
9.0
8.5
8.0
7.5
7.0
6.5
6.0
5.5
5.0
4.5
4.0
3.5
3.0
2.5
2.0
1.5
1.0
0.5
0.0
-0.5
f1 (ppm)

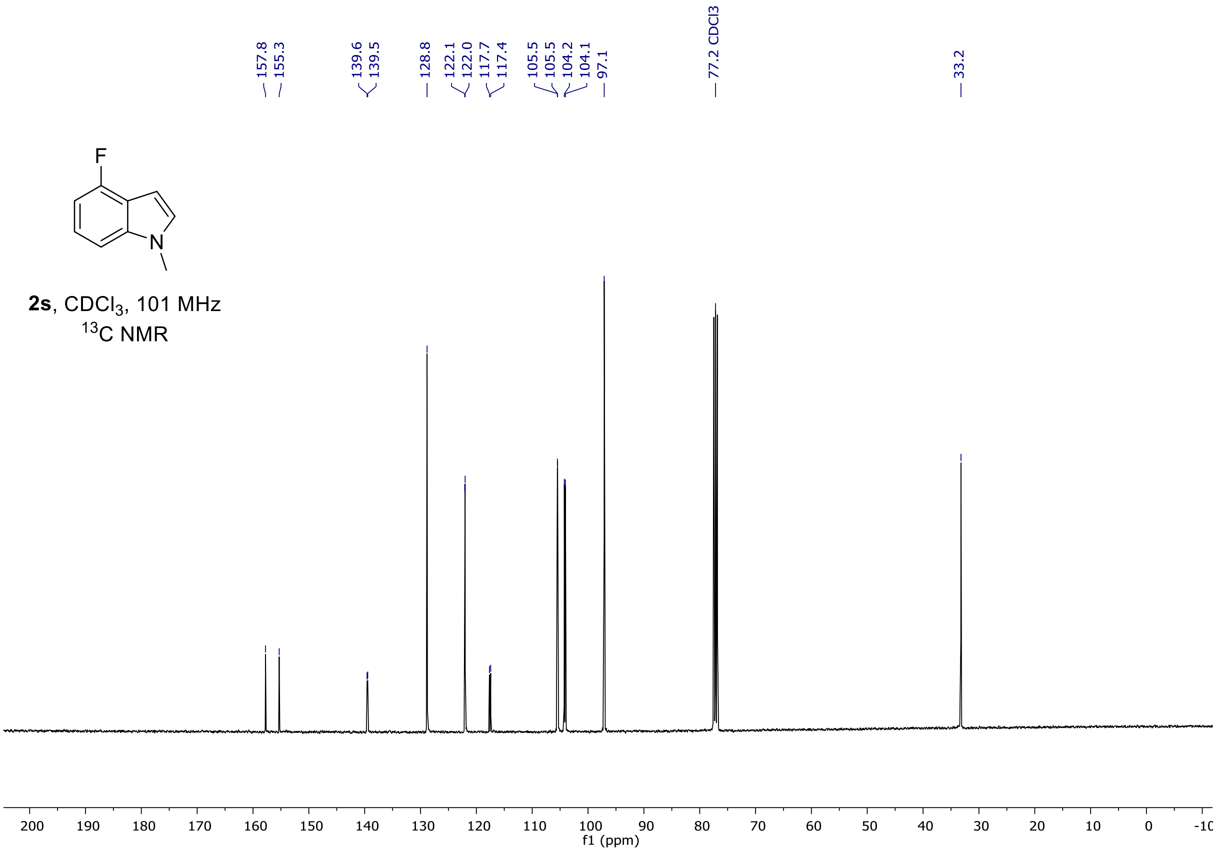
157.8
155.3
139.6
139.5
128.8
122.1
122.0
117.7
117.4
105.5
105.5
104.2
104.1
97.1
77.2 CDCl3
33.2
F
N
2s, CDCl3, 101 MHz
13C NMR
200
190
180
170
160
150
140
130
120
110
100
90
80
70
60
50
40
30
20
10
0
f1 (ppm)

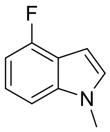

2s, $CDCl_3$, 376 MHz
^{19}F NMR

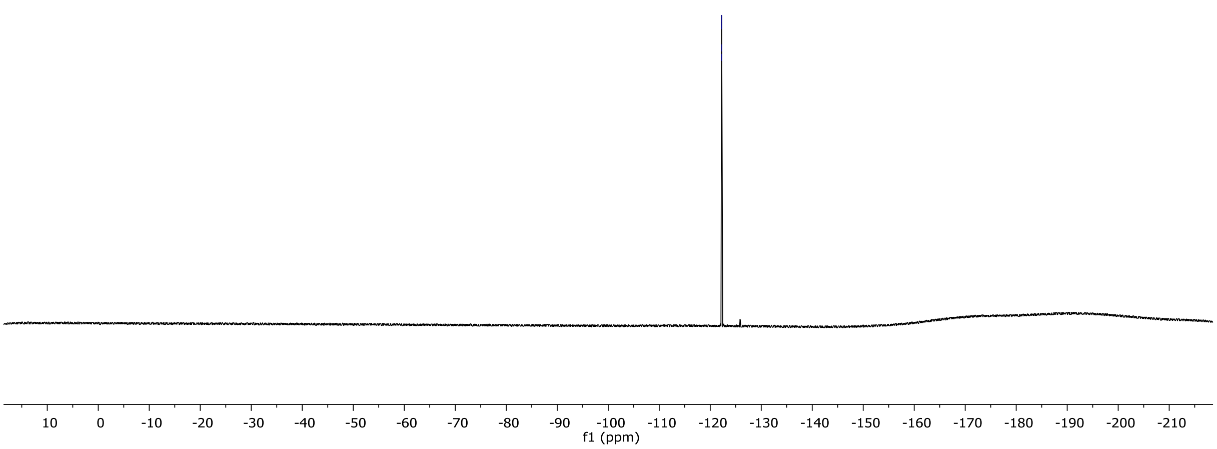

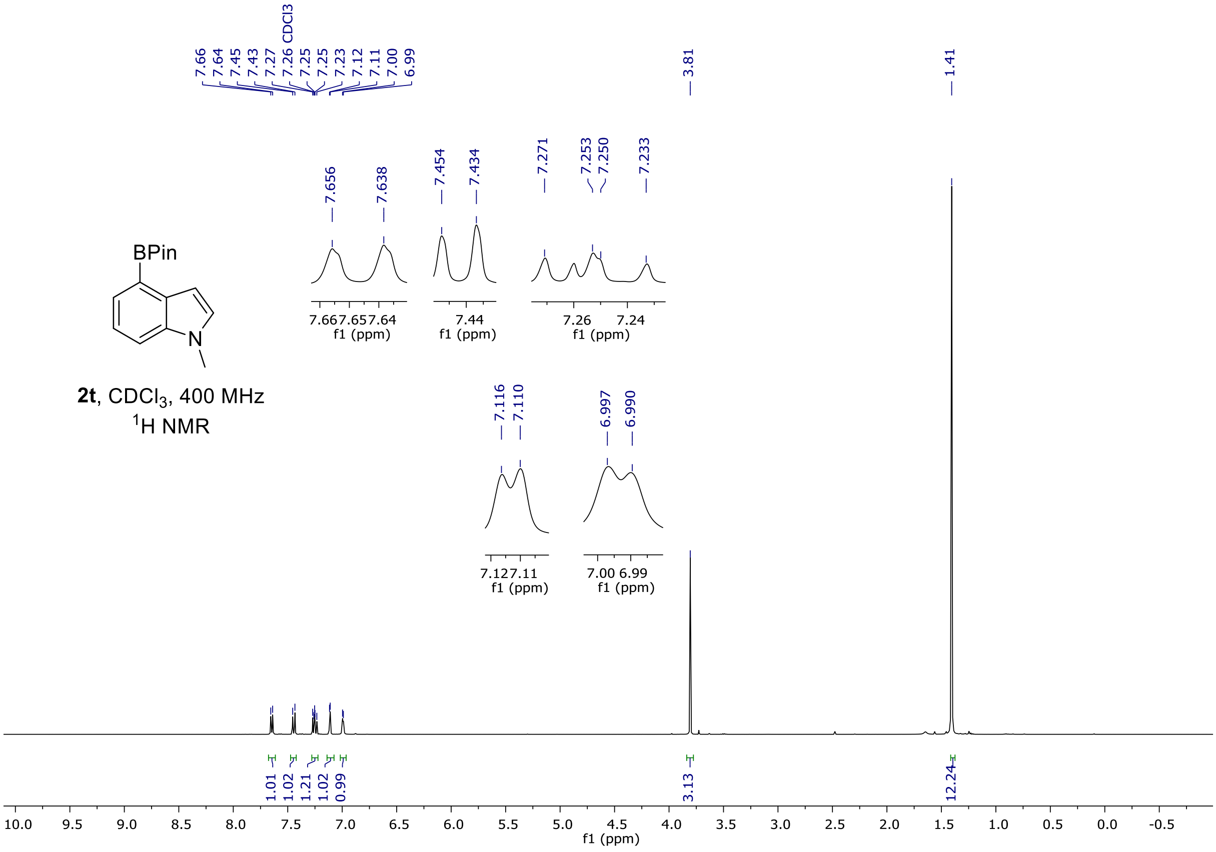

2t, $CDCl_3$, 400 MHz
^1H NMR

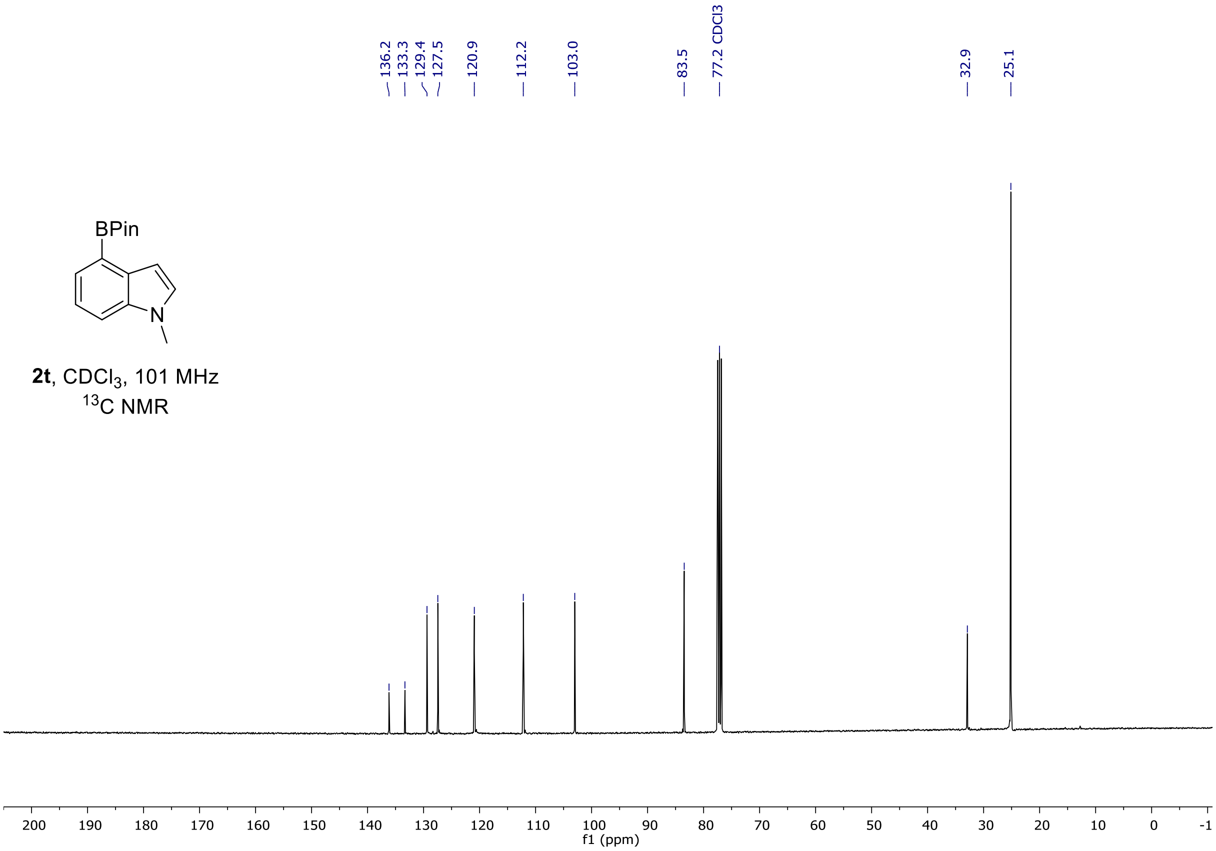

2t, $CDCl_3$, 101 MHz
^{13}C NMR

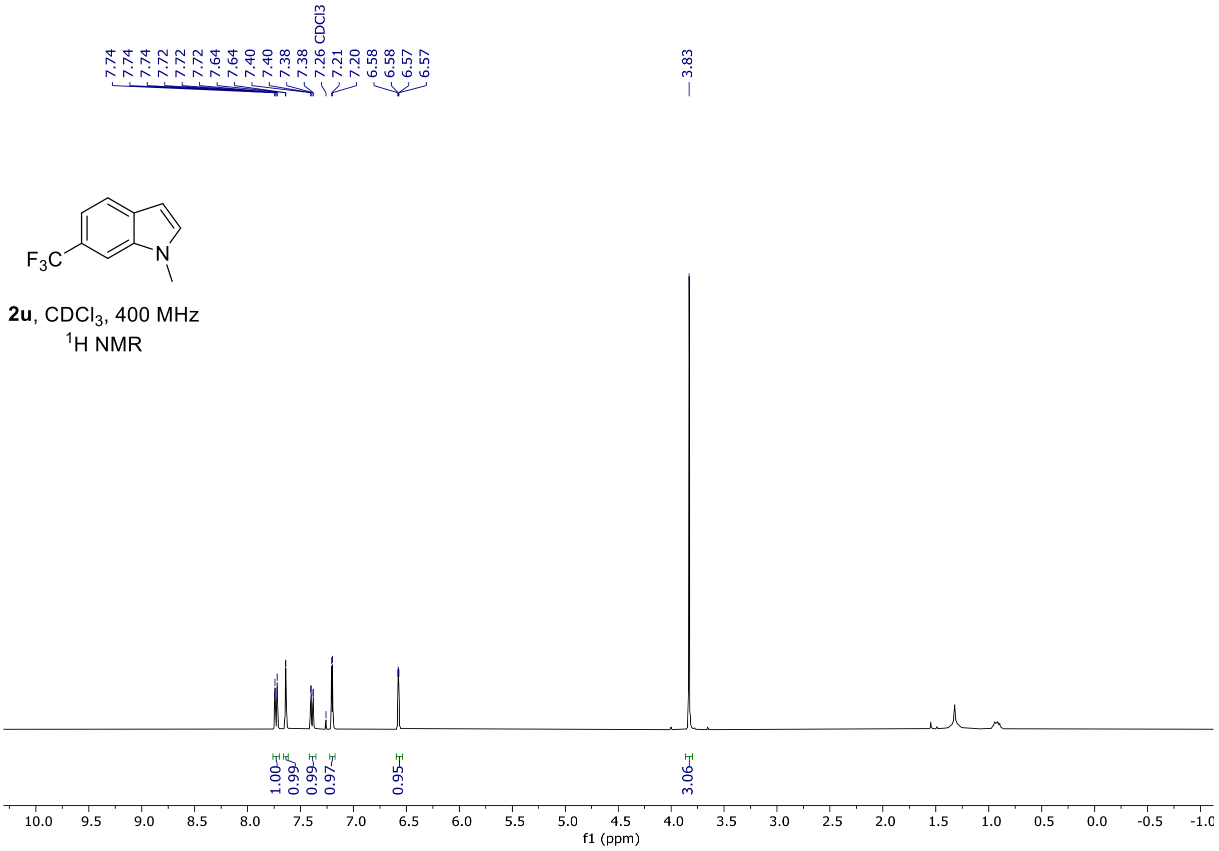
7.74
7.74
7.74
7.72
7.72
7.72
7.64
7.40
7.38
7.38
7.26 CDCl3
7.21
7.20
6.58
6.58
6.57
6.57
3.83
F3C
N
2u, CDCl3, 400 MHz
1H NMR
1.00
0.99
0.99
0.97
0.95
3.06
10.0 9.5 9.0 8.5 8.0 7.5 7.0 6.5 6.0 5.5 5.0 4.5 4.0 3.5 3.0 2.5 2.0 1.5 1.0 0.5 0.0 -0.5 -1.0
f1 (ppm)

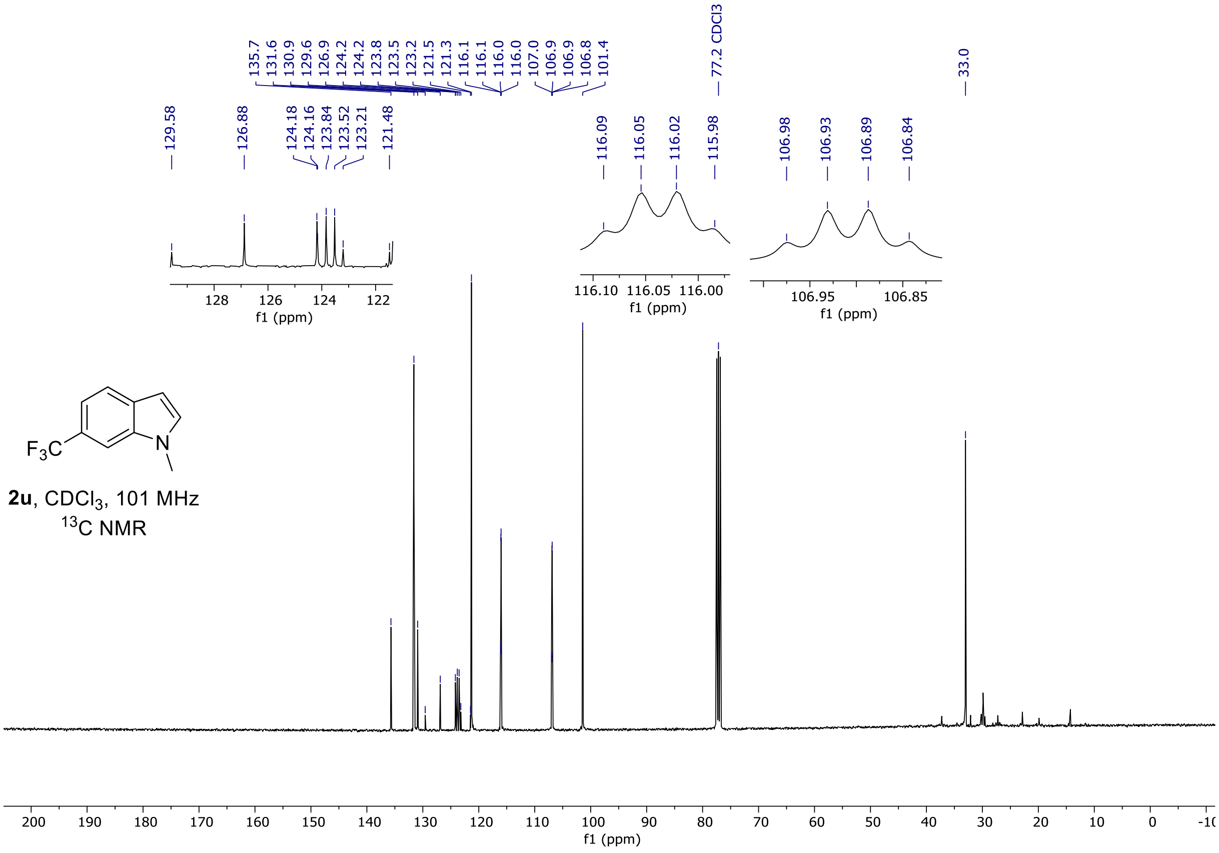
135.7
131.6
130.9
129.6
126.9
124.2
124.2
123.8
123.5
123.2
121.5
121.3
116.1
116.1
116.0
116.0
107.0
106.9
106.9
106.8
101.4
77.2 CDCl3
33.0
129.58
126.88
124.18
124.16
123.84
123.52
123.21
121.48
128 126 124 122
f1 (ppm)
116.09
116.05
116.02
115.98
116.10 116.05 116.00
f1 (ppm)
106.98
106.93
106.89
106.84
106.95 106.85
f1 (ppm)
F3C
N
2u, CDCl3, 101 MHz
13C NMR
200 190 180 170 160 150 140 130 120 110 100 90 80 70 60 50 40 30 20 10 0 -10
f1 (ppm)

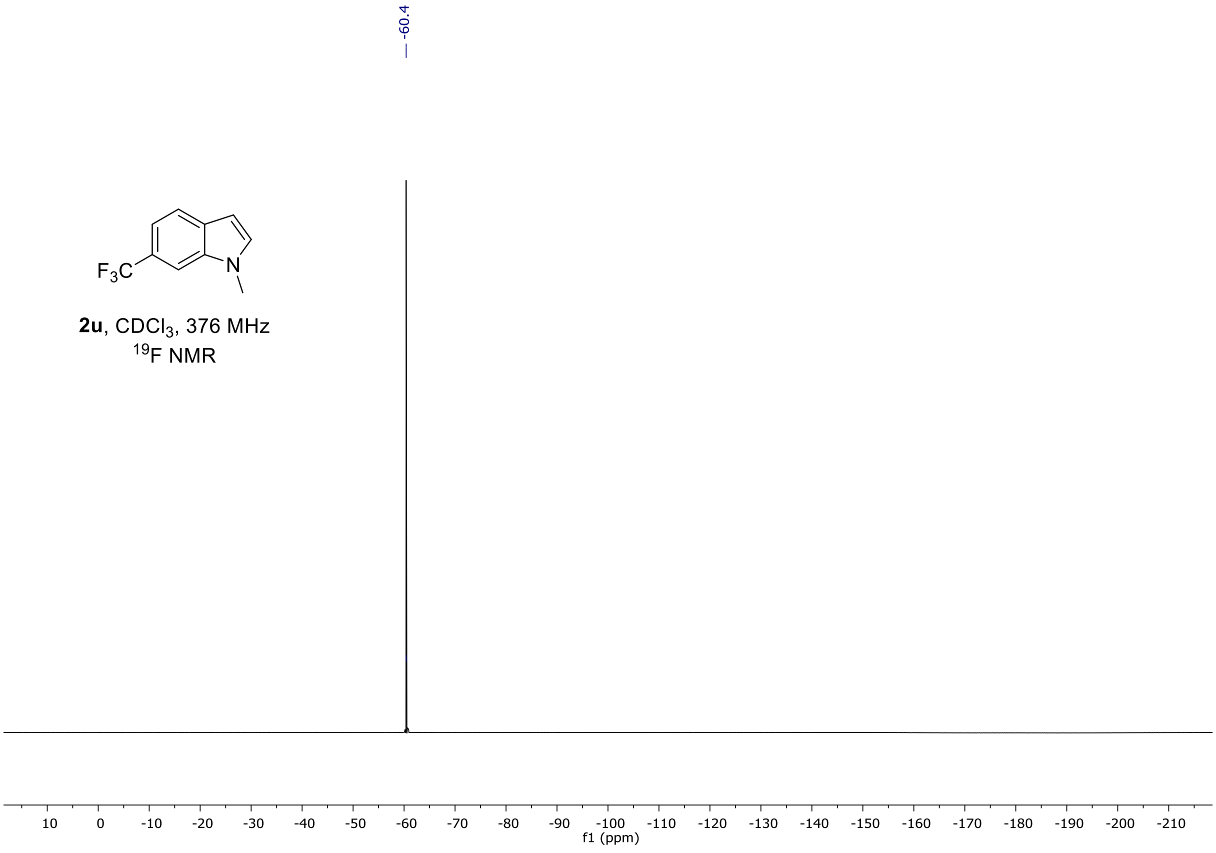

2u, $CDCl_3$, 376 MHz
^{19}F NMR

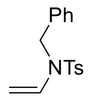

19, $CDCl_3$, 400 MHz
^1H NMR

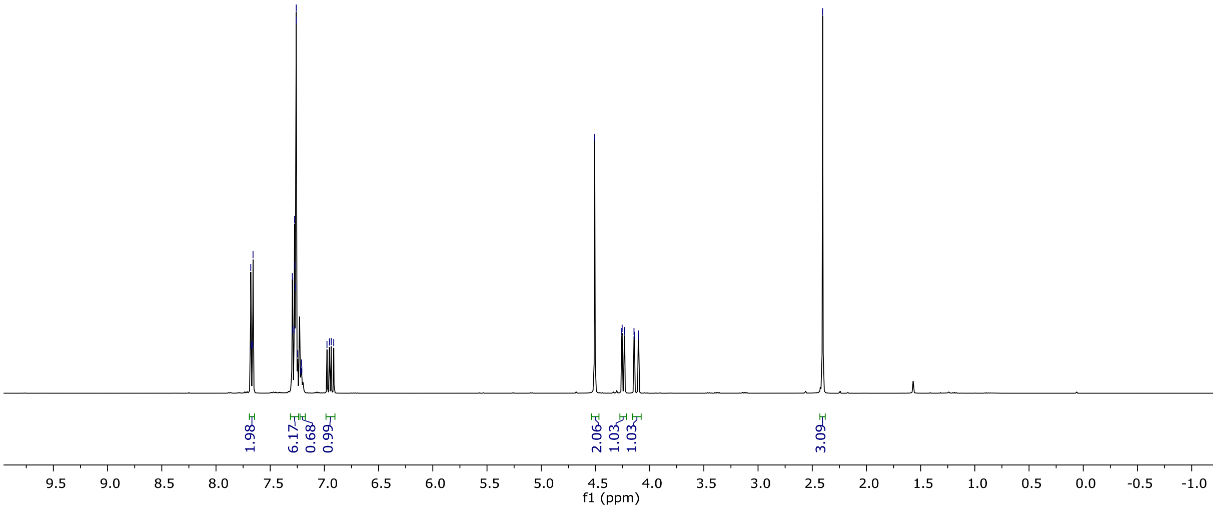

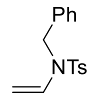

19, $CDCl_3$, 101 MHz
^{13}C NMR

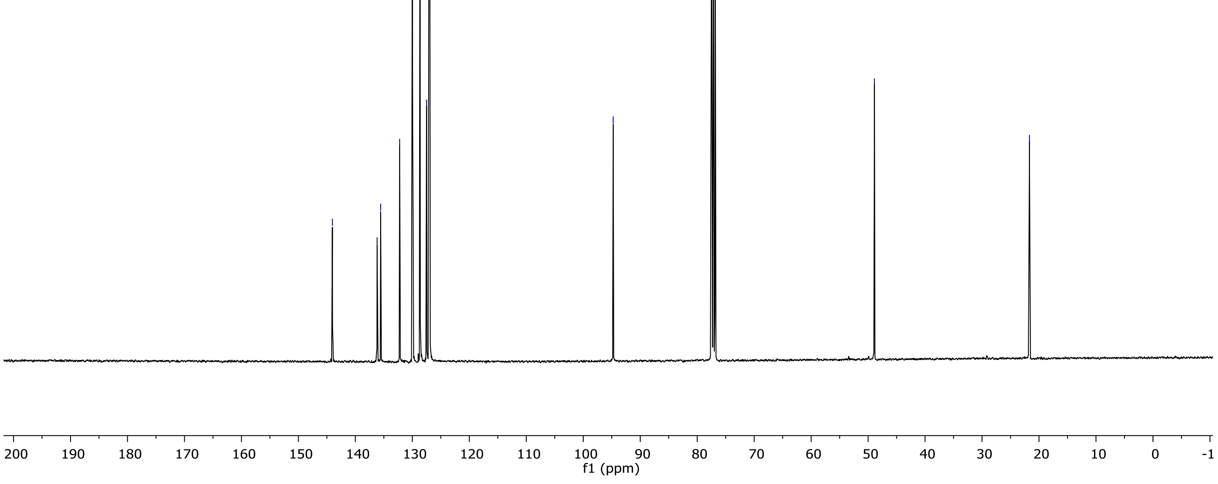

20, $CDCl_3$, 400 MHz
^1H NMR

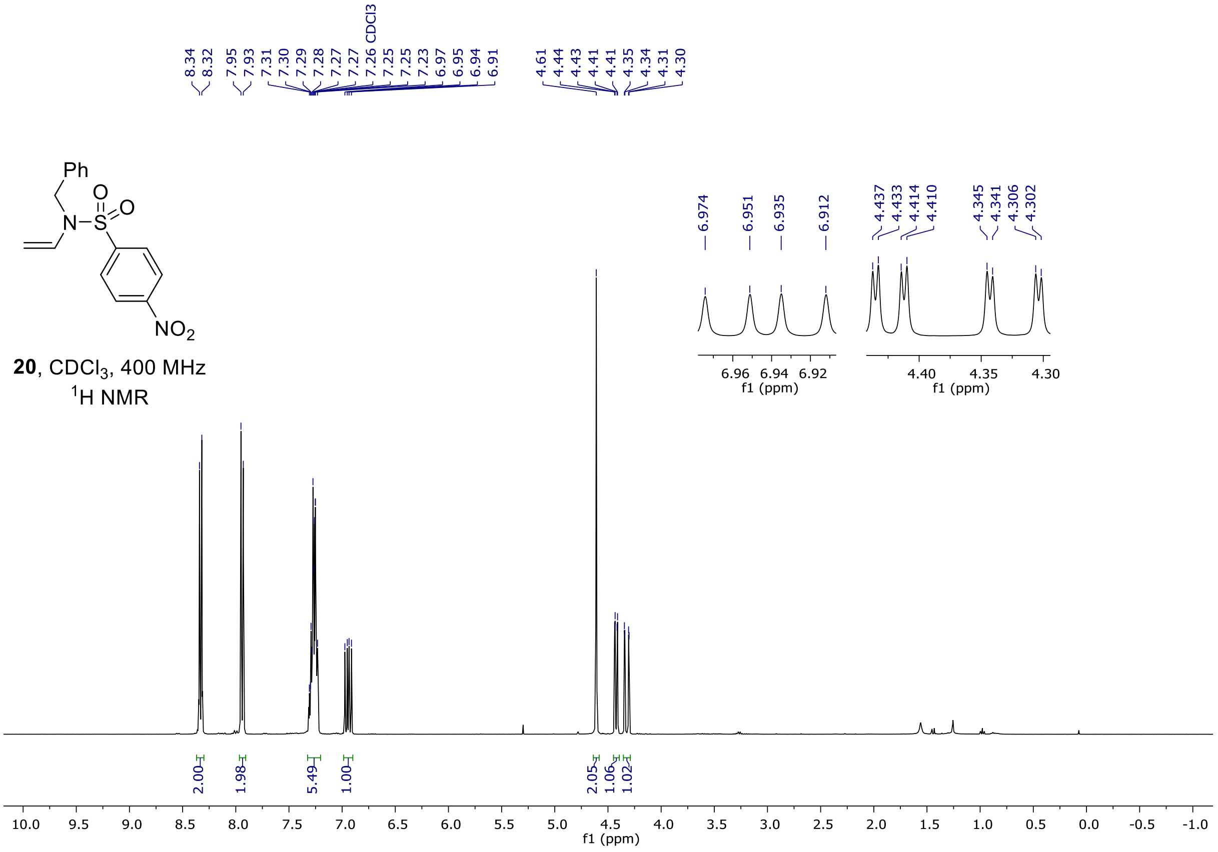

20, $CDCl_3$, 101 MHz
^{13}C NMR

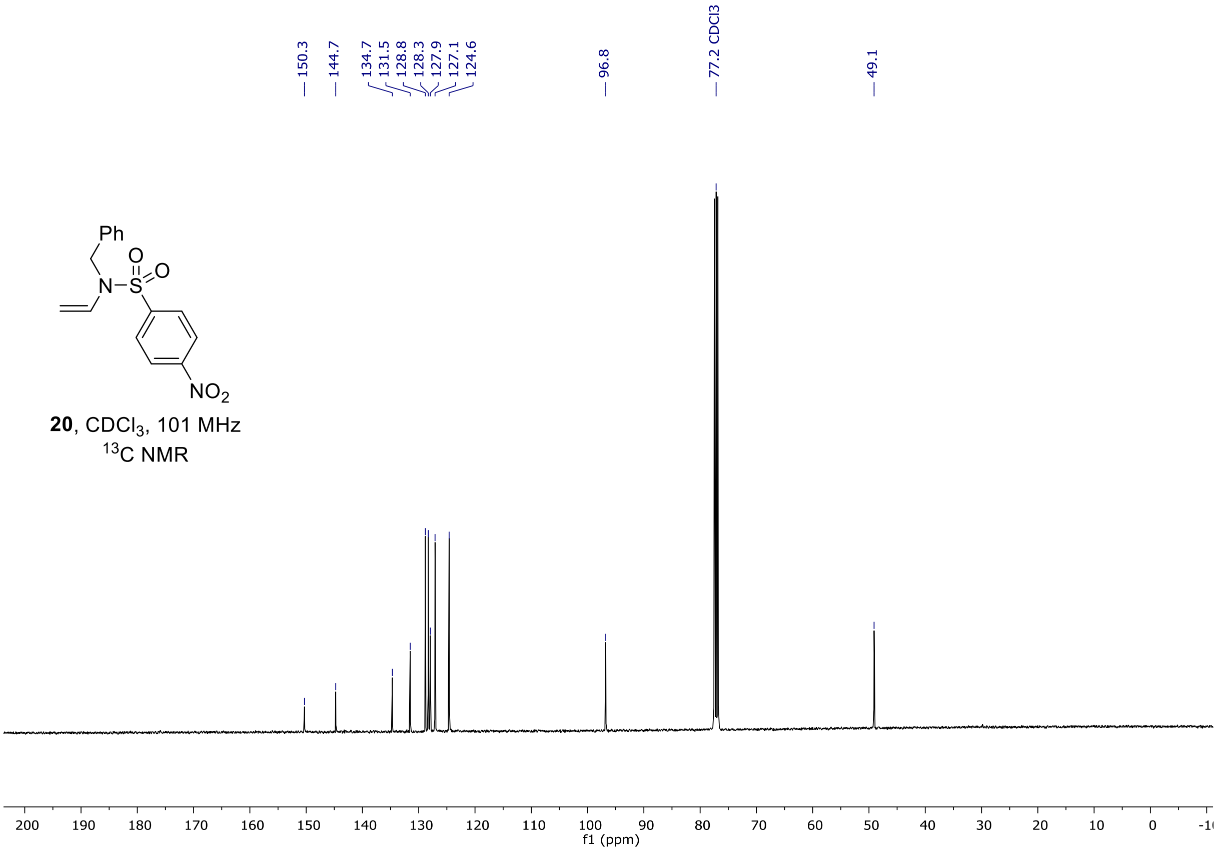

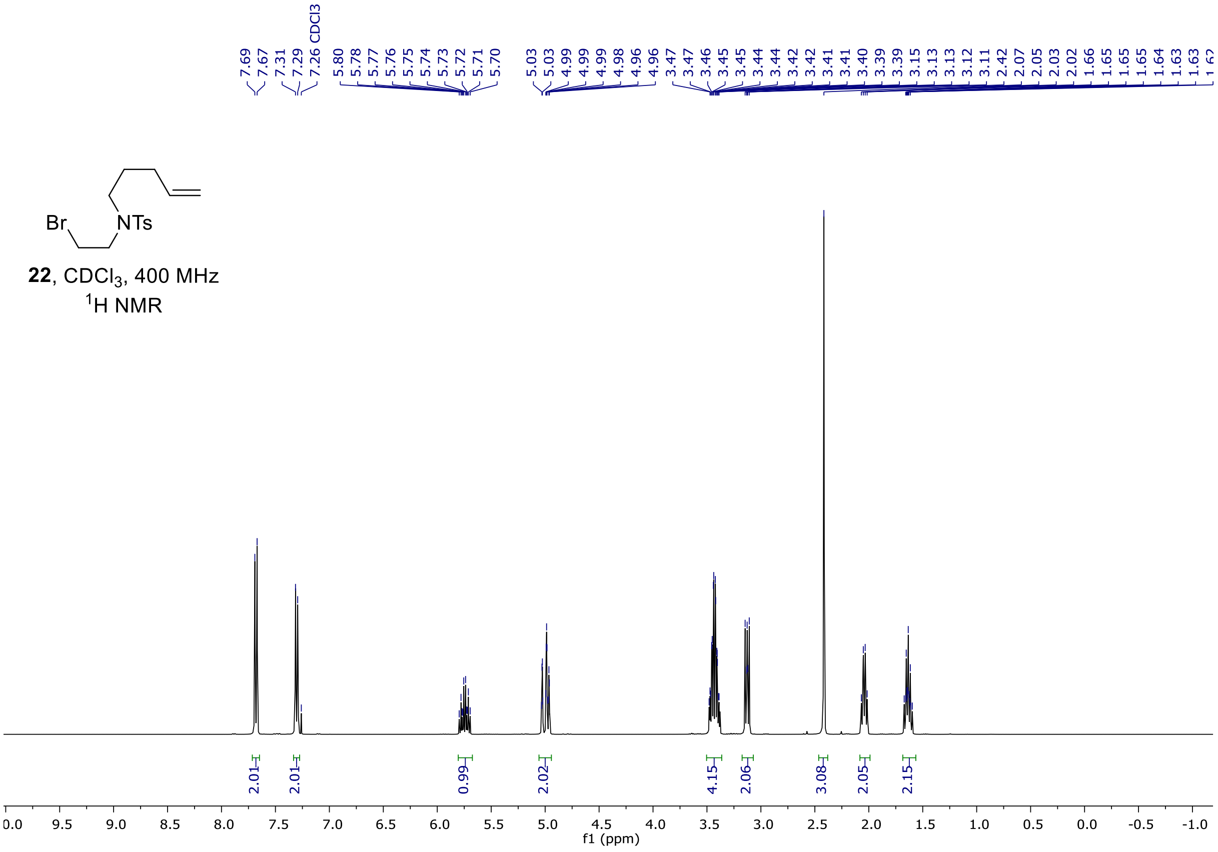
7.69
7.67
7.31
7.29
7.26 CDCl3
5.80
5.78
5.77
5.76
5.75
5.74
5.73
5.72
5.71
5.70
5.03
5.03
4.99
4.99
4.99
4.98
4.96
4.96
3.47
3.47
3.46
3.45
3.45
3.44
3.44
3.42
3.42
3.41
3.41
3.40
3.39
3.39
3.15
3.13
3.13
3.12
3.11
2.42
2.07
2.05
2.03
2.02
1.66
1.65
1.65
1.64
1.63
1.63
Br
NTs
22, CDCl3, 400 MHz
1H NMR
2.01
2.01
0.99
2.02
4.15
2.06
3.08
2.05
2.15
f1 (ppm)

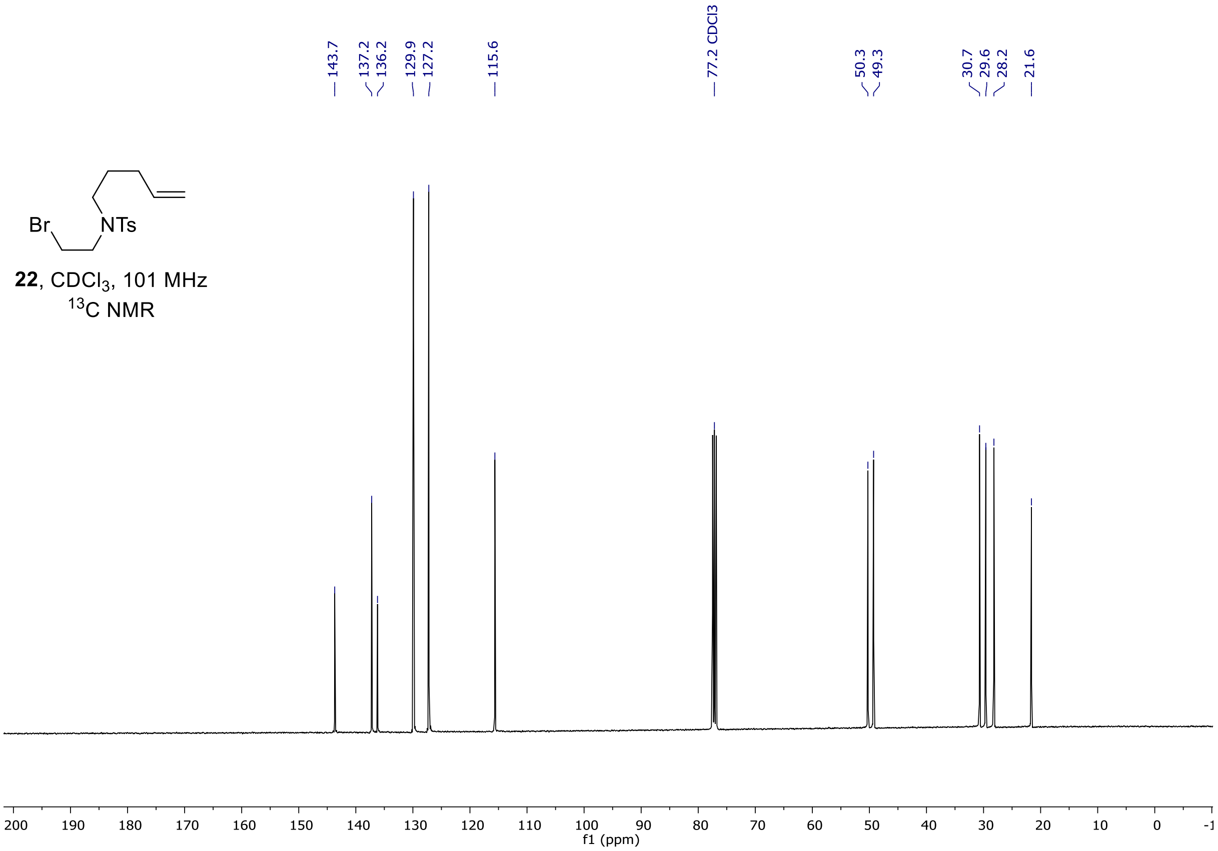
143.7
137.2
136.2
129.9
127.2
115.6
77.2 CDCl3
50.3
49.3
30.7
29.6
28.2
21.6
Br
NTs
22, CDCl3, 101 MHz
13C NMR
f1 (ppm)

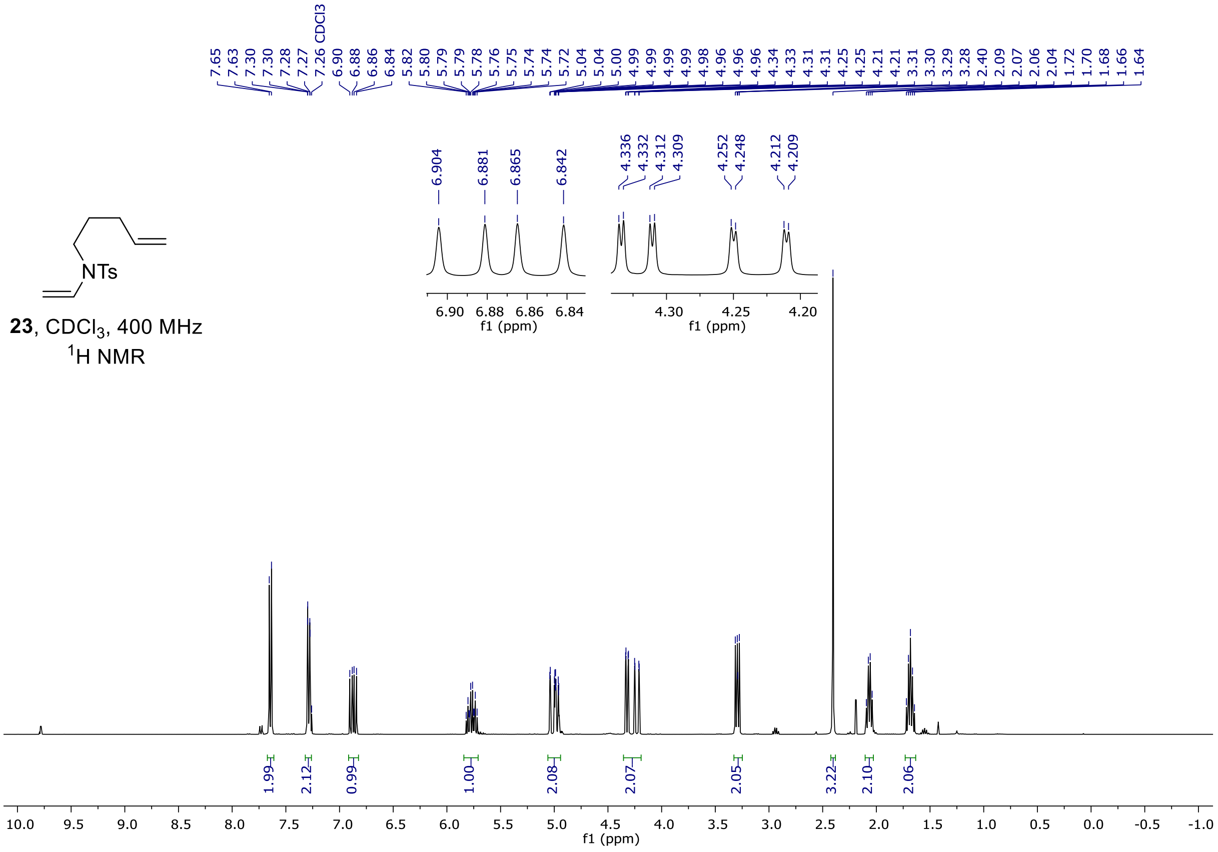
NTs
23, CDCl3, 400 MHz
1H NMR
f1 (ppm)

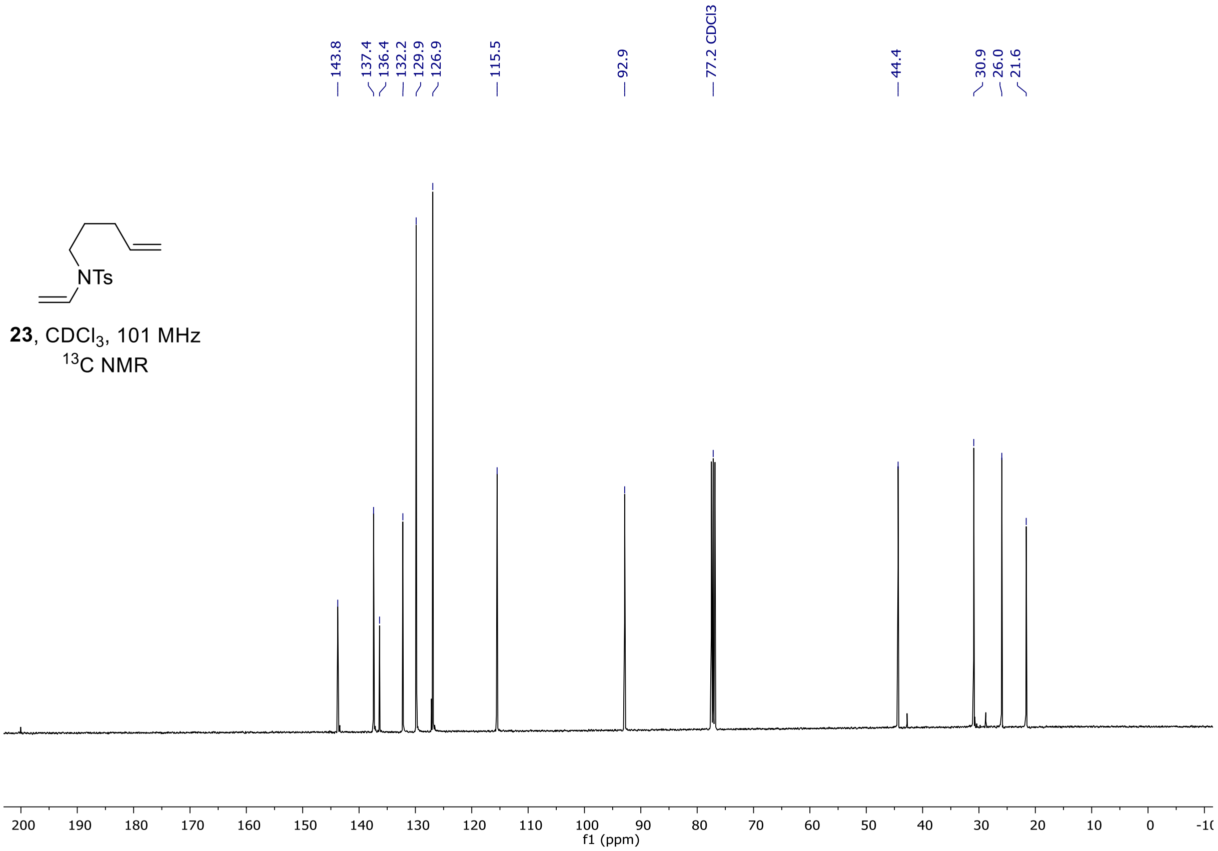
NTs
23, CDCl3, 101 MHz
13C NMR
143.8
137.4
136.4
132.2
129.9
126.9
115.5
92.9
77.2 CDCl3
44.4
30.9
26.0
21.6
f1 (ppm)

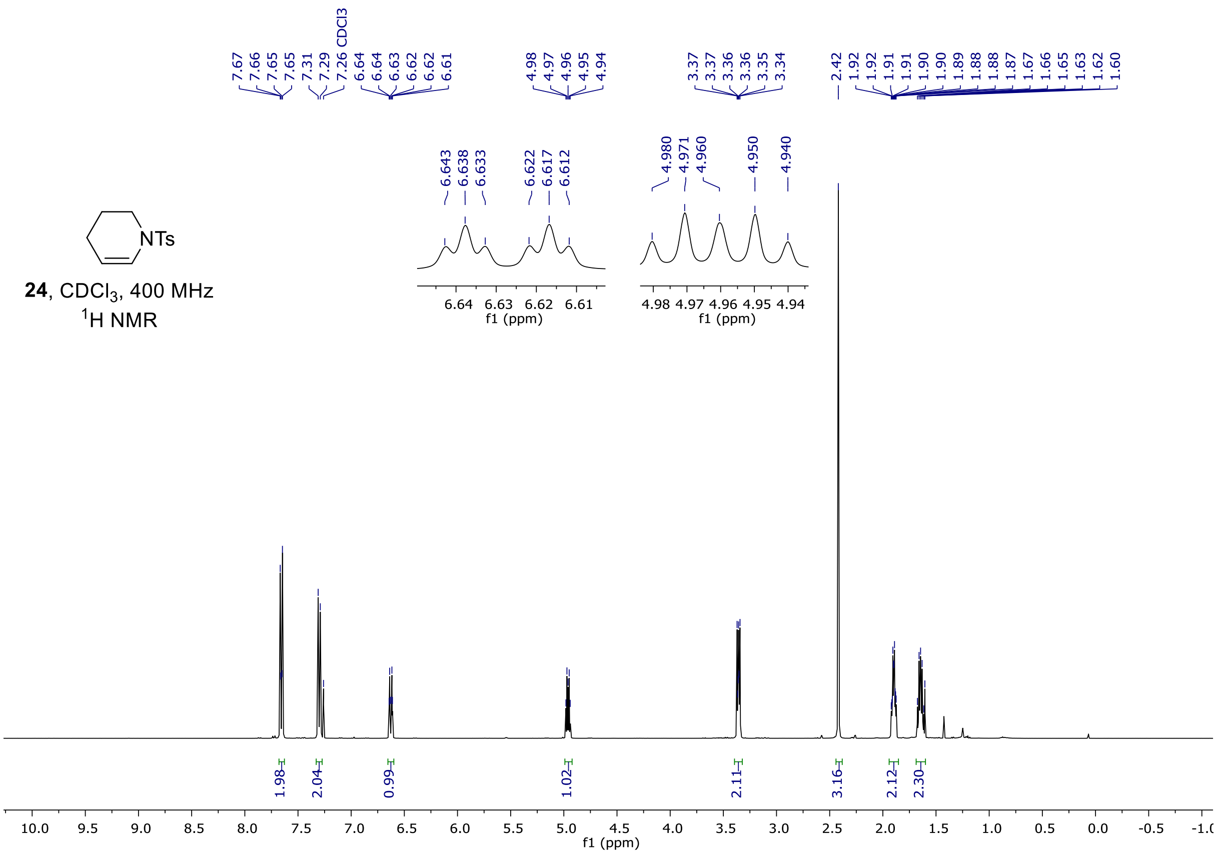

24, $CDCl_3$, 400 MHz
^1H NMR

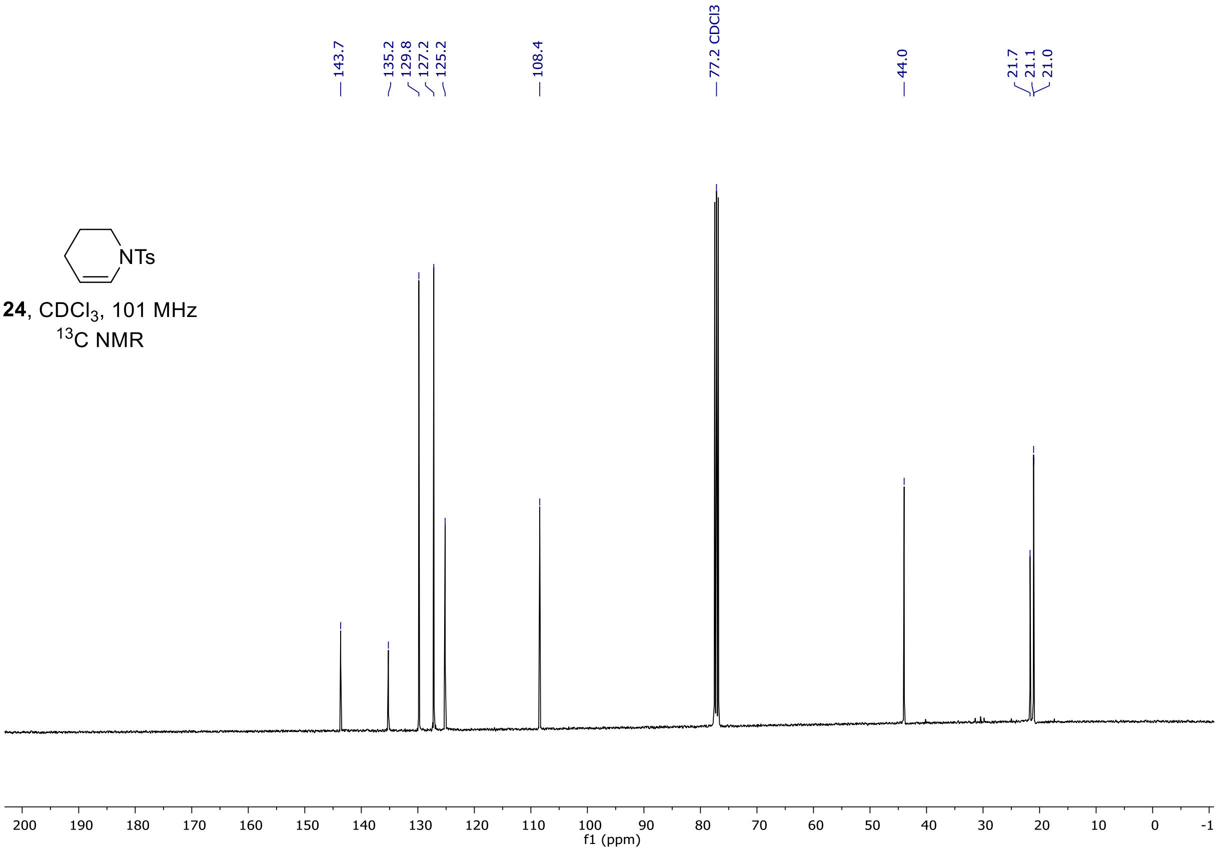

24, $CDCl_3$, 101 MHz
^{13}C NMR

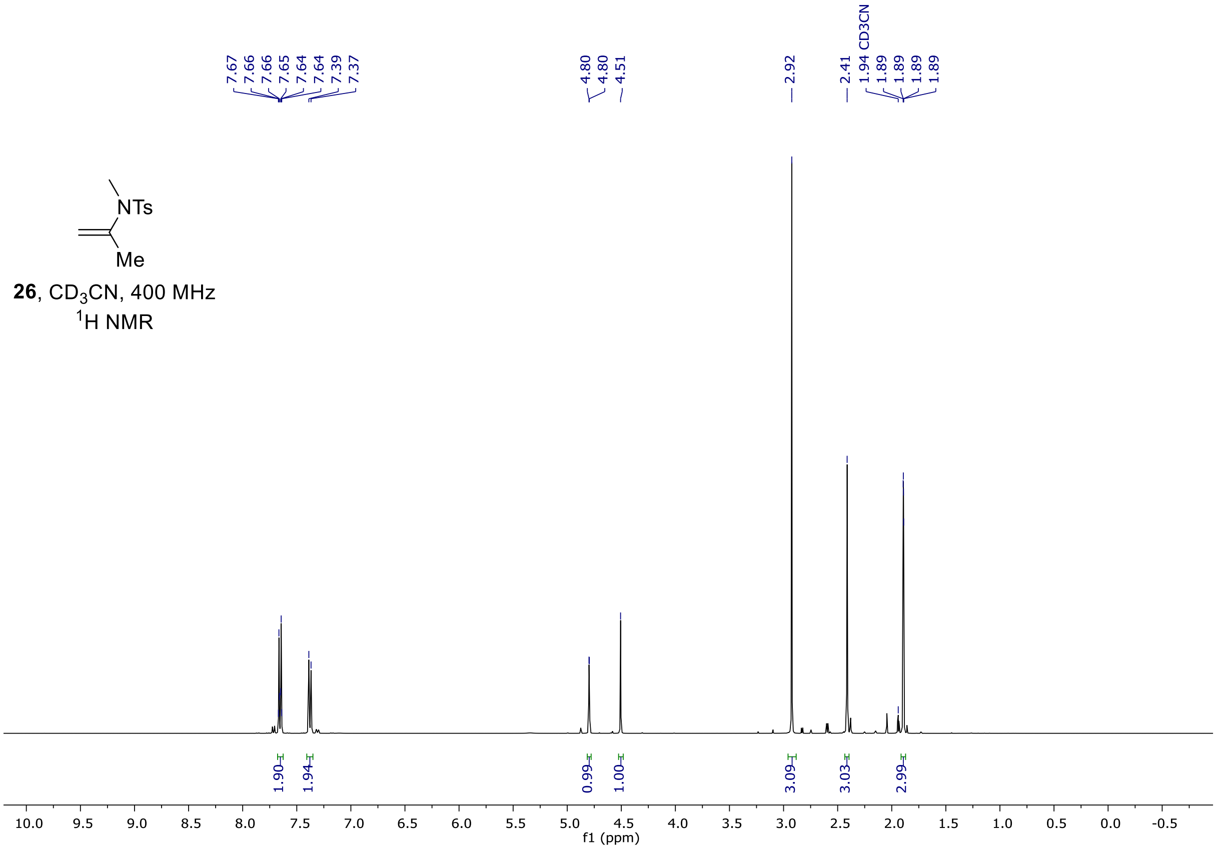

26, CD_3CN, 400 MHz

^{1}H NMR

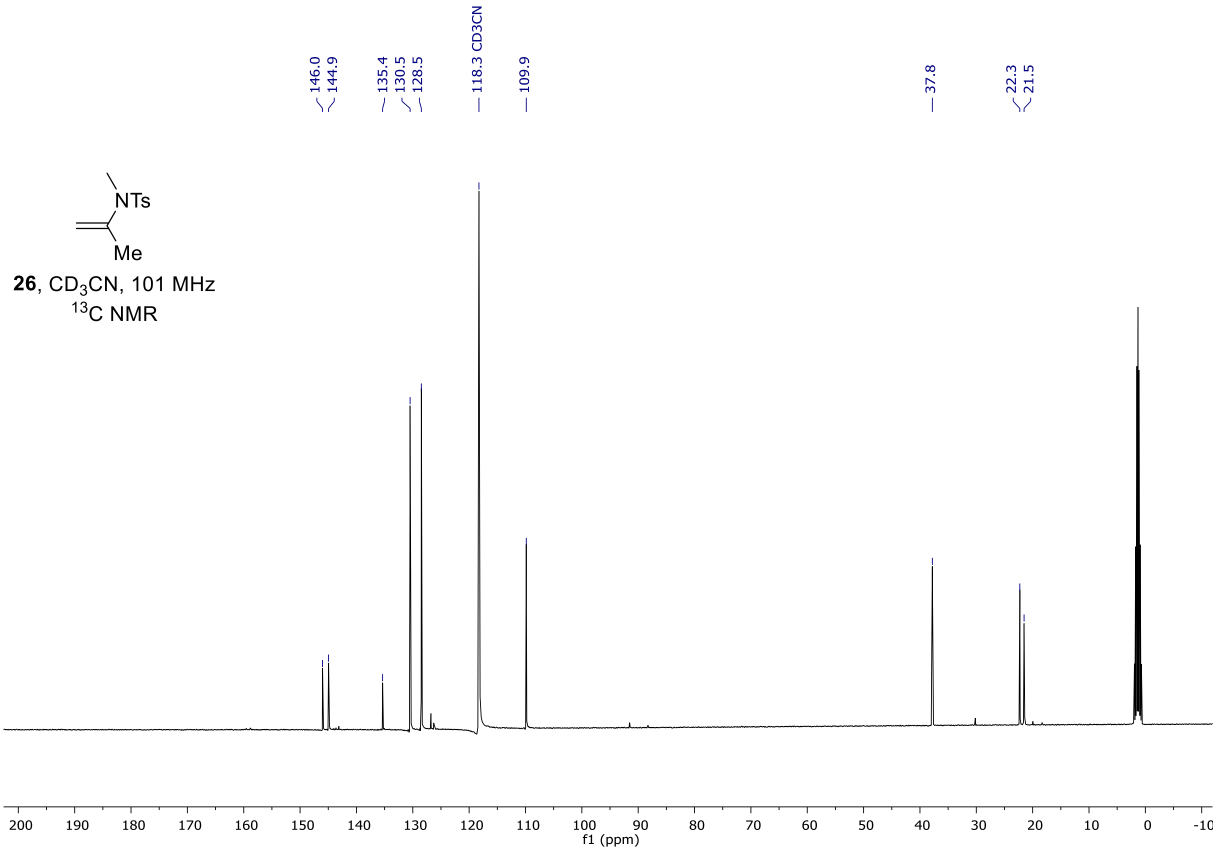

26, CD_3CN, 101 MHz

^{13}C NMR

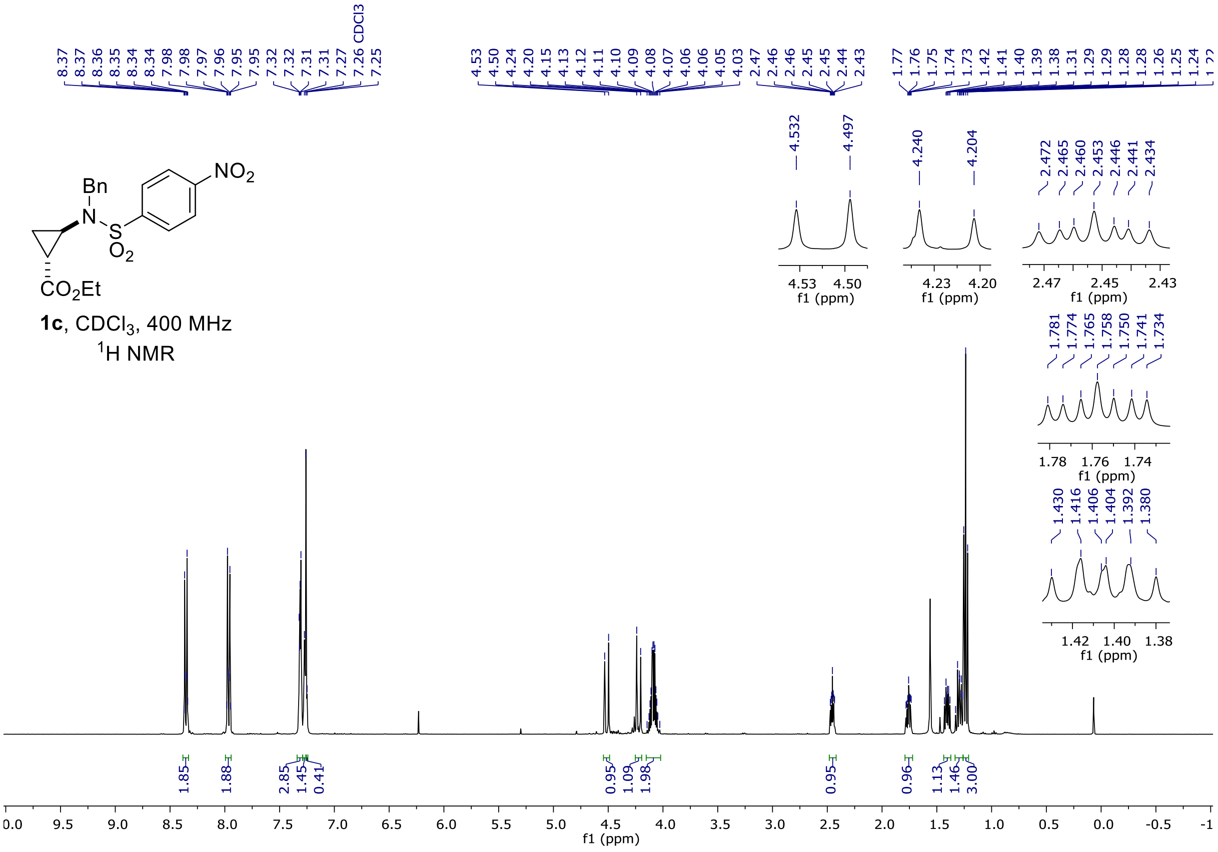

1c, CDCl3, 400 MHz
1H NMR
f1 (ppm)

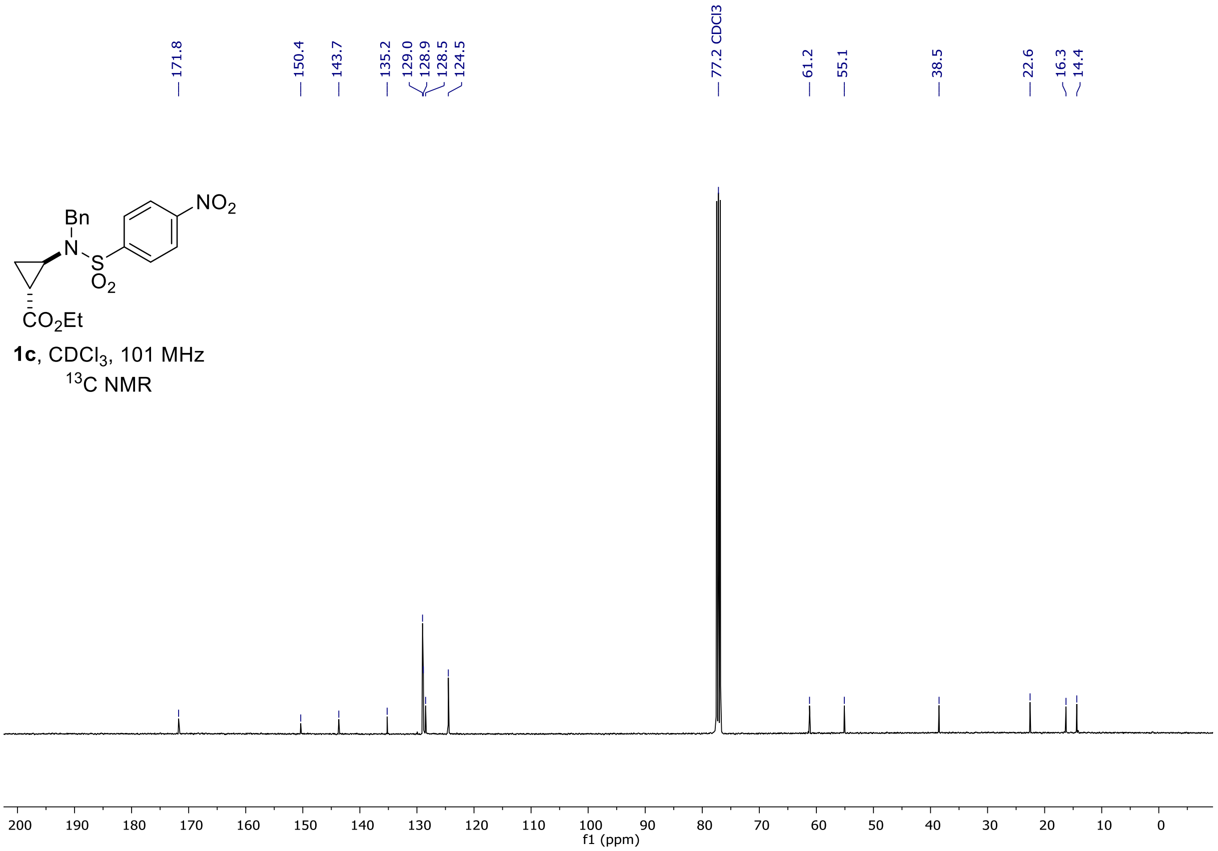

1c, CDCl3, 101 MHz
13C NMR
f1 (ppm)

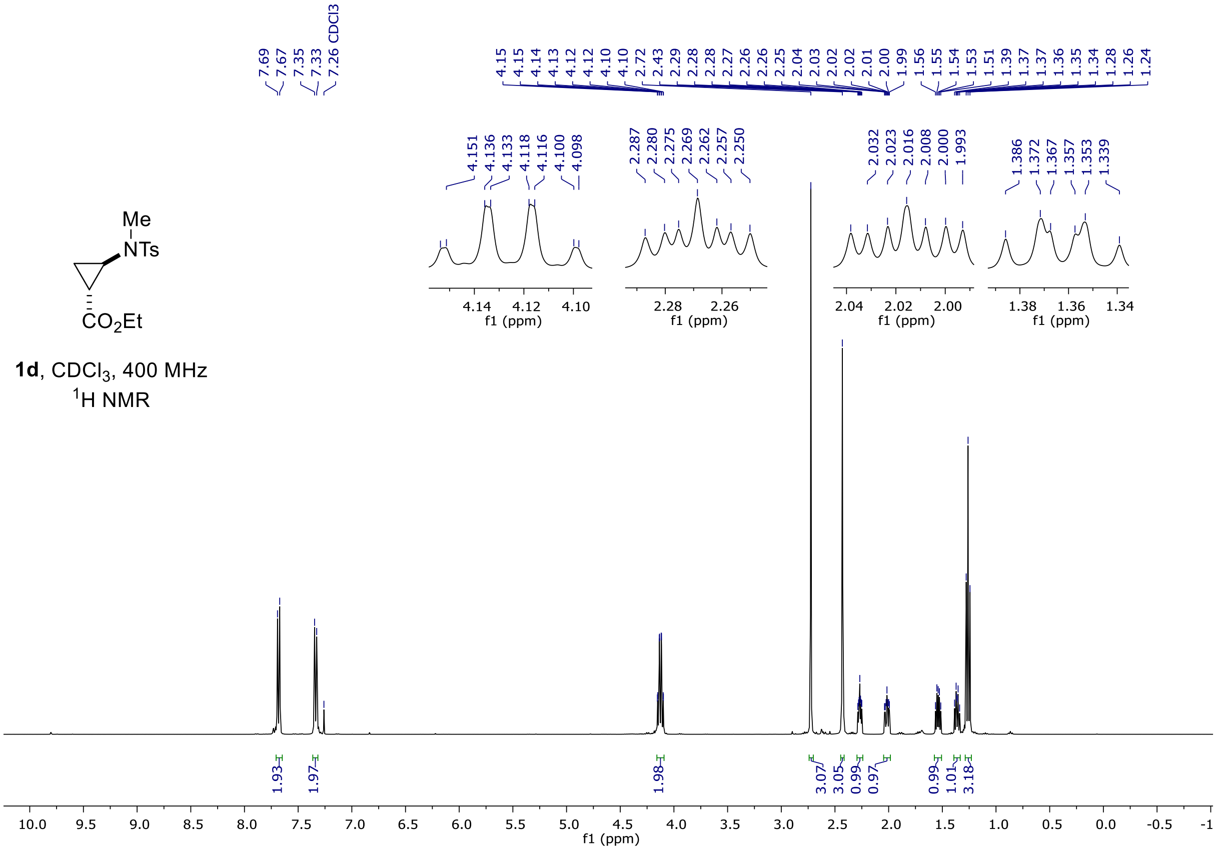

Me
NTs
CO2Et
1d, CDCl3, 400 MHz
1H NMR
7.69
7.67
7.35
7.33
7.26 CDCl3
4.15 4.15 4.14 4.13 4.12 4.12 4.10 4.10 2.72 2.43 2.29 2.28 2.28 2.27 2.26 2.26 2.25 2.04 2.03 2.02 2.02 2.01 2.00 1.99 1.56 1.55 1.54 1.53 1.51 1.39 1.37 1.36 1.35 1.34 1.28 1.26 1.24
4.151 4.136 4.133 4.118 4.116 4.100 4.098
2.287 2.280 2.275 2.269 2.262 2.257 2.250
2.032 2.023 2.016 2.008 2.000 1.993
1.386 1.372 1.367 1.357 1.353 1.339
1.93
1.97
1.98
3.07
3.05
0.99
0.97
0.99
1.01
3.18
f1 (ppm)

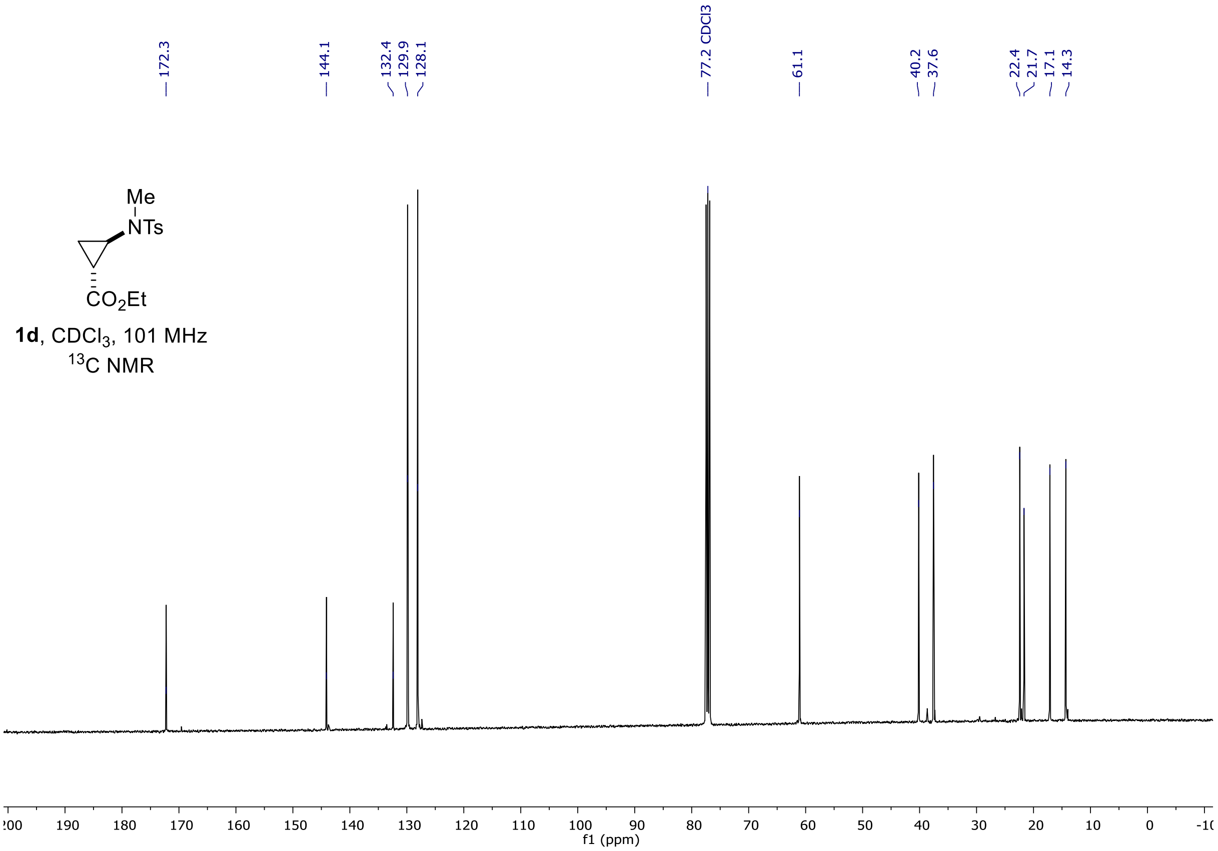

Me
NTs
CO2Et
1d, CDCl3, 101 MHz
13C NMR
172.3
144.1
132.4
129.9
128.1
77.2 CDCl3
61.1
40.2
37.6
22.4
21.7
17.1
14.3
f1 (ppm)

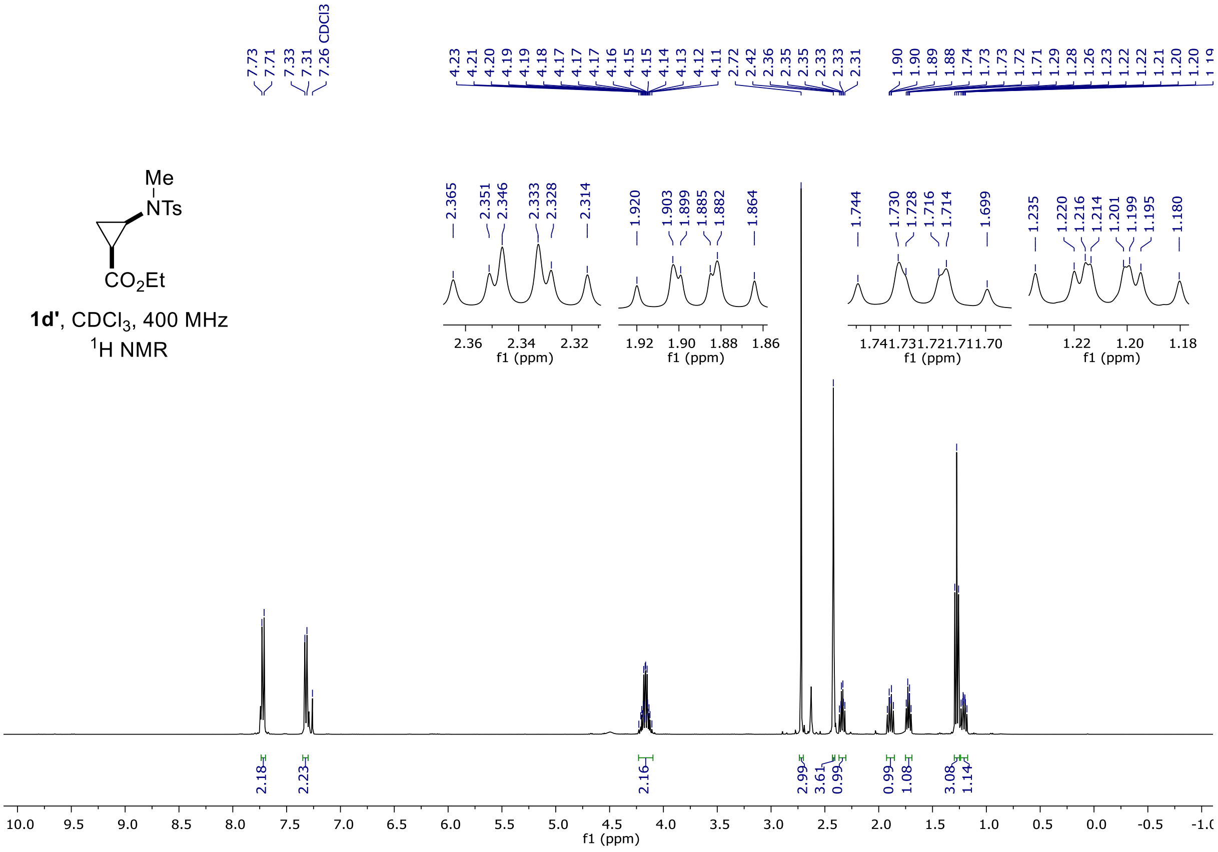
Me
NTs
CO2Et
1d', CDCl3, 400 MHz
1H NMR
f1 (ppm)

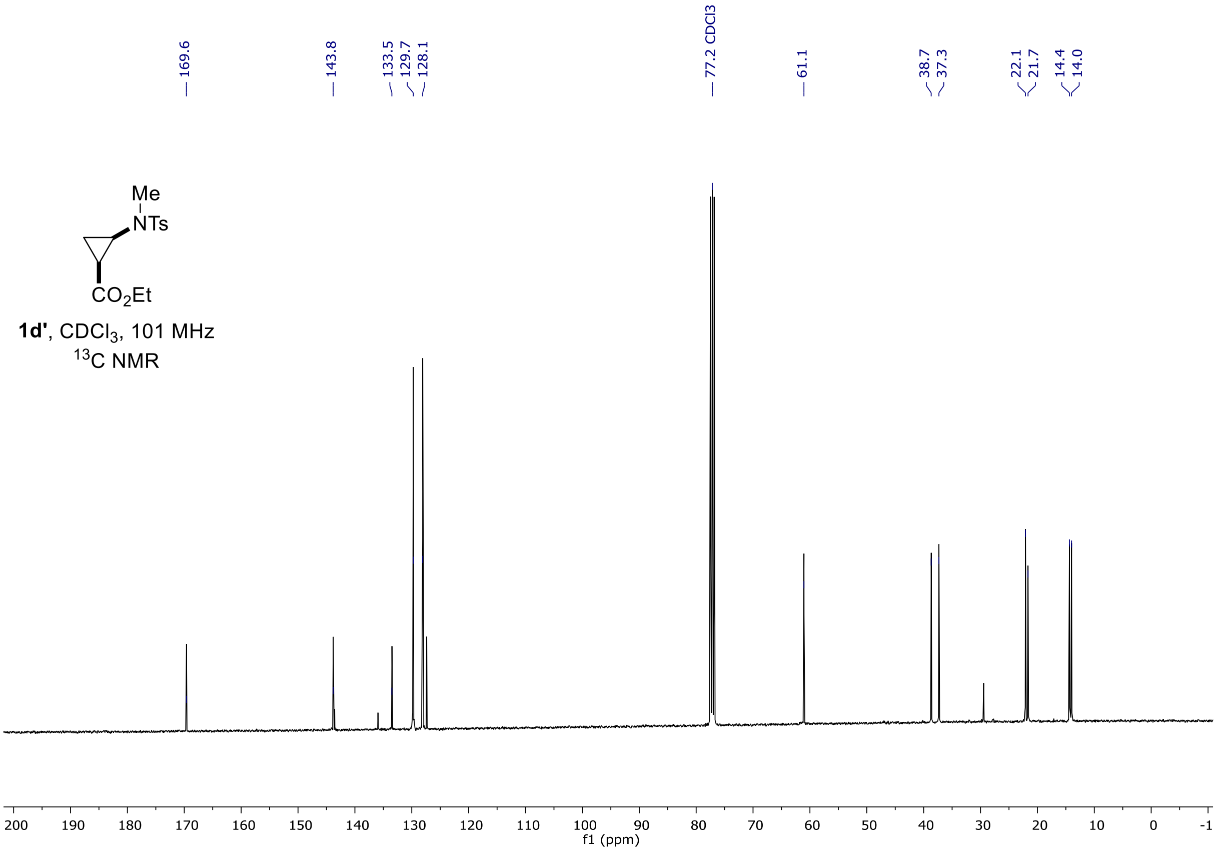
169.6
143.8
133.5
129.7
128.1
77.2 CDCl3
61.1
38.7
37.3
22.1
21.7
14.4
14.0
Me
NTs
CO2Et
1d', CDCl3, 101 MHz
13C NMR
f1 (ppm)

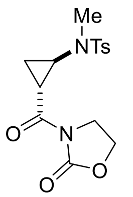

1e, $CDCl_3$, 400 MHz
^1H NMR

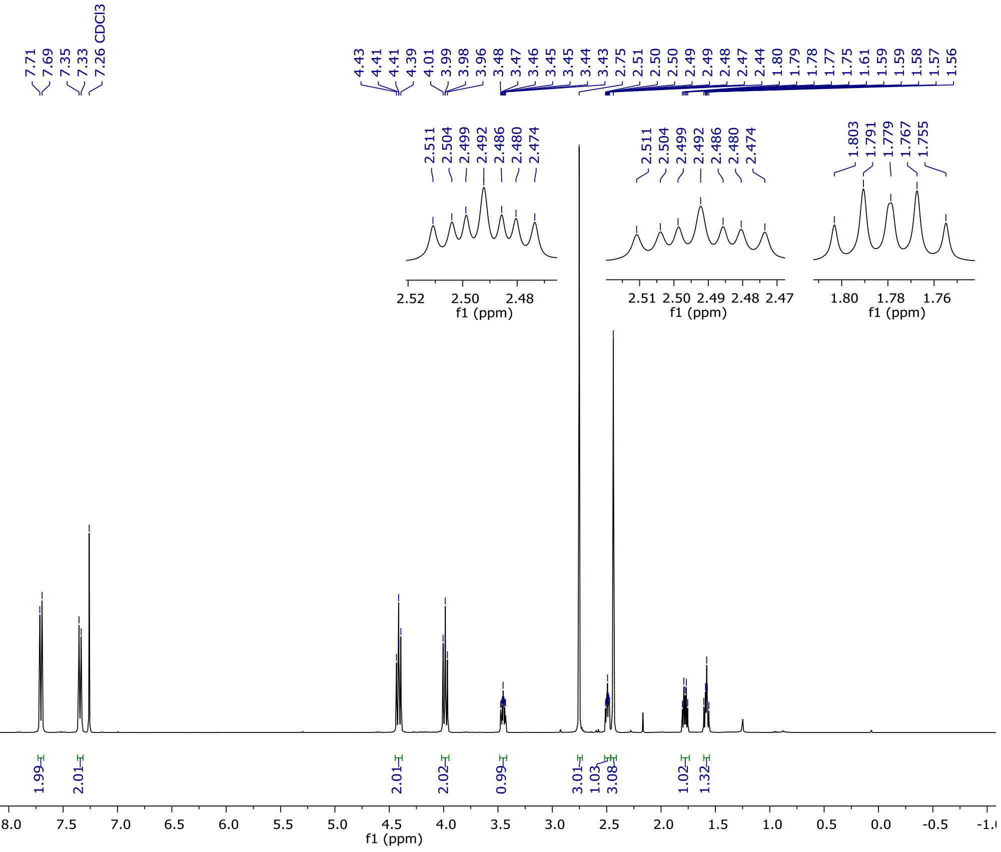

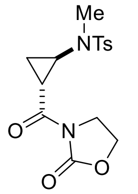

1e, $CDCl_3$, 101 MHz
^{13}C NMR

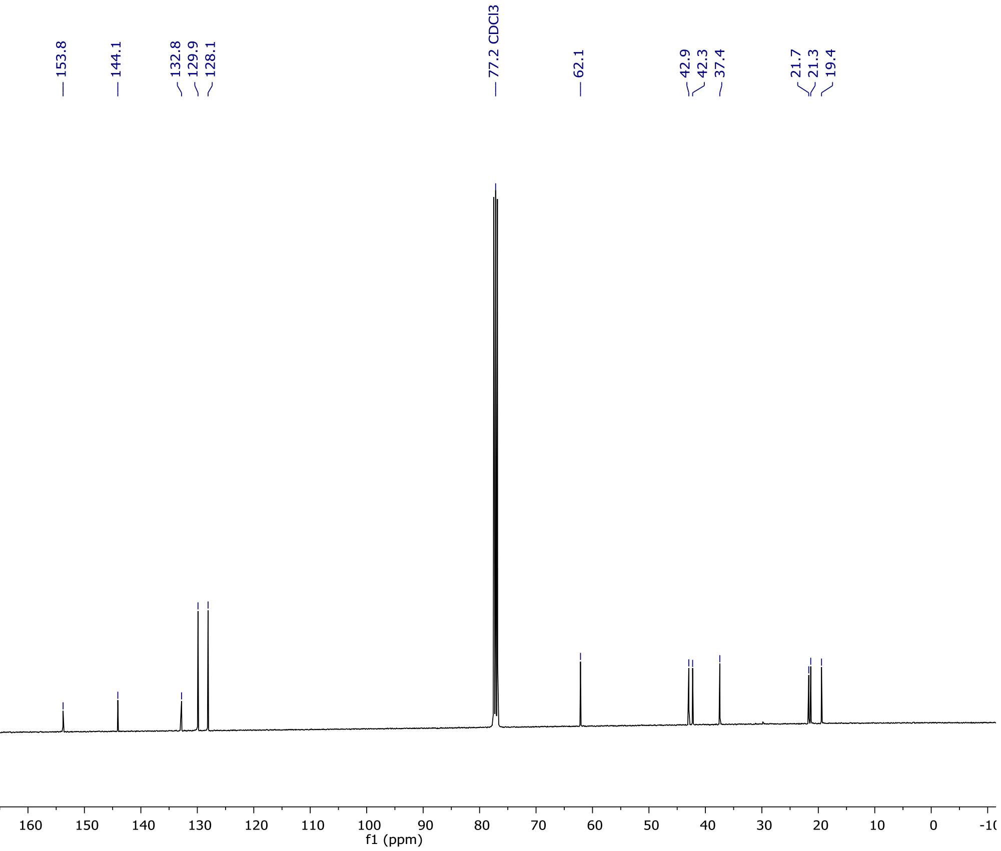

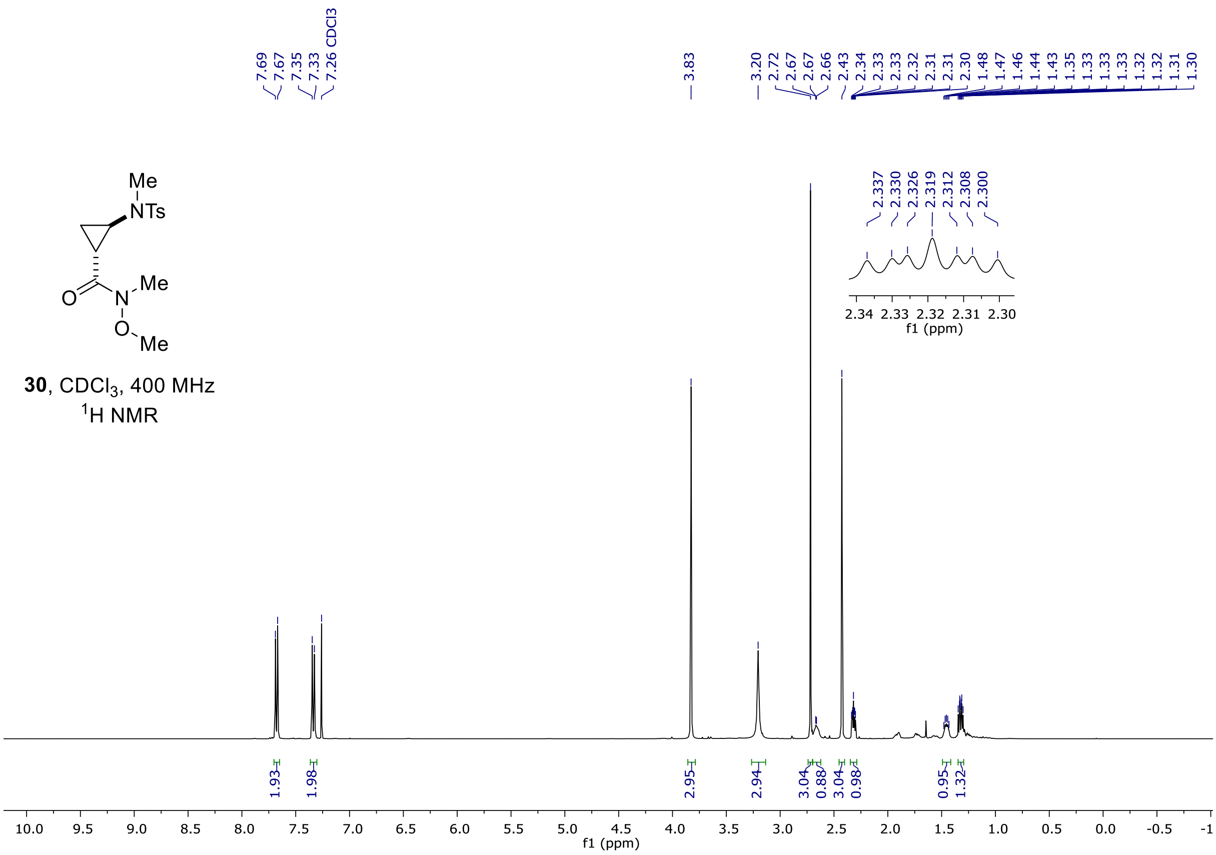
7.69
7.67
7.35
7.33
7.26 CDCl3
3.83
3.20
2.72
2.67
2.67
2.66
2.43
2.34
2.33
2.33
2.32
2.31
2.31
2.30
1.48
1.47
1.46
1.44
1.43
1.35
1.33
1.33
1.33
1.32
1.32
1.31
1.30
2.337
2.330
2.326
2.319
2.312
2.308
2.300
2.34 2.33 2.32 2.31 2.30
f1 (ppm)
Me
NTs
O
N
Me
O
Me
30, CDCl3, 400 MHz
1H NMR
1.93
1.98
2.95
2.94
3.04
0.88
3.04
0.98
0.95
1.32
10.0 9.5 9.0 8.5 8.0 7.5 7.0 6.5 6.0 5.5 5.0 4.5 4.0 3.5 3.0 2.5 2.0 1.5 1.0 0.5 0.0 -0.5 -1
f1 (ppm)

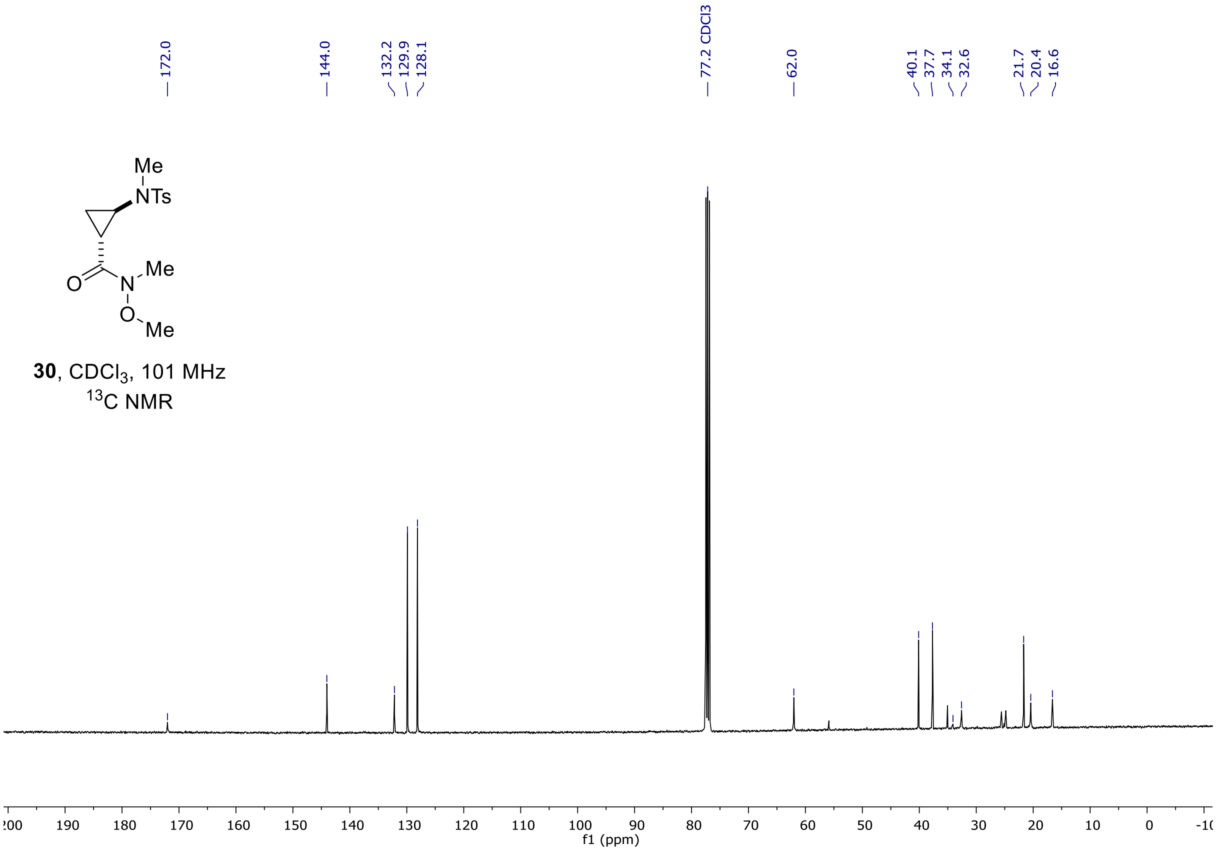
172.0
144.0
132.2
129.9
128.1
77.2 CDCl3
62.0
40.1
37.7
34.1
32.6
21.7
20.4
16.6
Me
NTs
O
N
Me
O
Me
30, CDCl3, 101 MHz
13C NMR
200 190 180 170 160 150 140 130 120 110 100 90 80 70 60 50 40 30 20 10 0 -10
f1 (ppm)

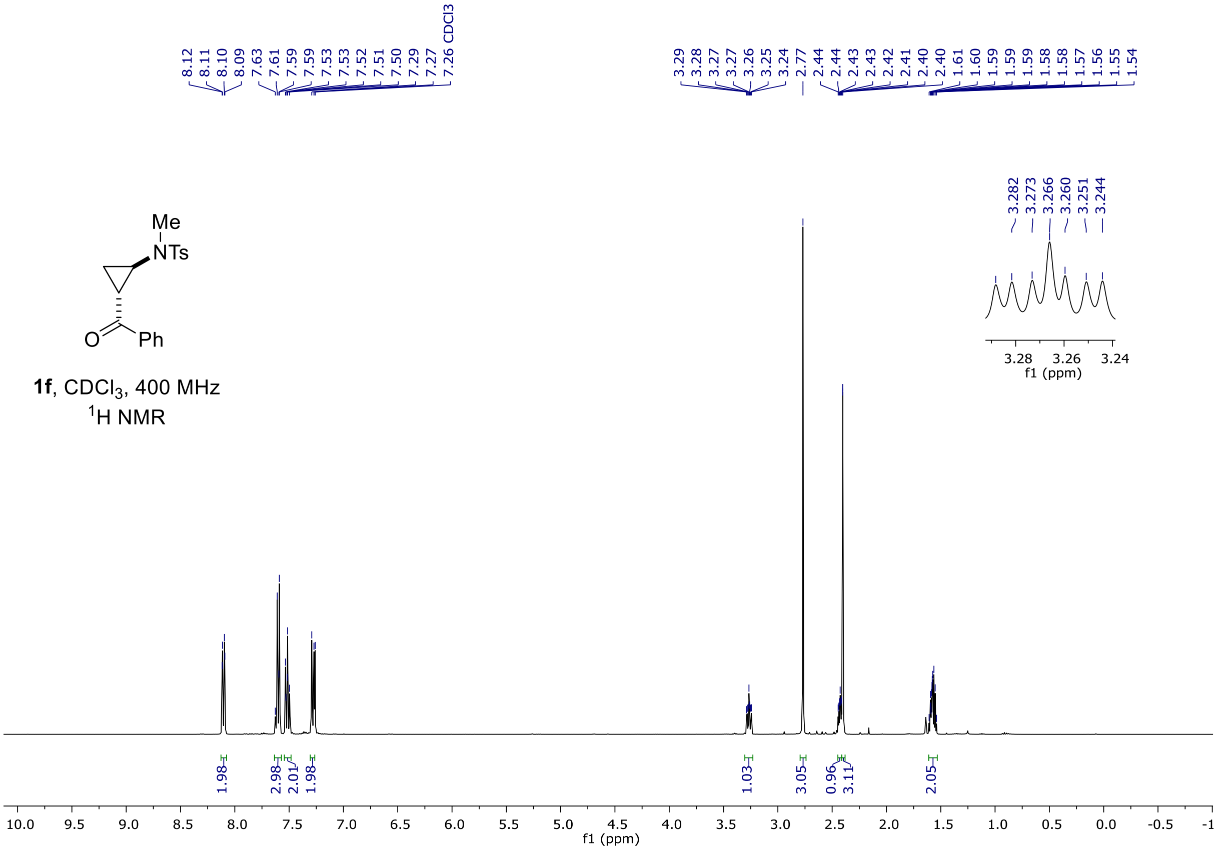
8.12 8.11 8.10 8.09 7.63 7.61 7.59 7.59 7.53 7.53 7.52 7.51 7.50 7.29 7.27 7.26 CDCl3
3.29 3.28 3.27 3.27 3.26 3.25 3.24 2.77 2.44 2.44 2.43 2.43 2.42 2.41 2.40 2.40 1.61 1.60 1.59 1.59 1.59 1.58 1.58 1.57 1.56 1.55 1.54
3.282 3.273 3.266 3.260 3.251 3.244
Me
NTs
O
Ph
1f, CDCl3, 400 MHz
1H NMR
1.98 2.98 2.01 1.98 1.03 3.05 0.96 3.11 2.05
f1 (ppm)

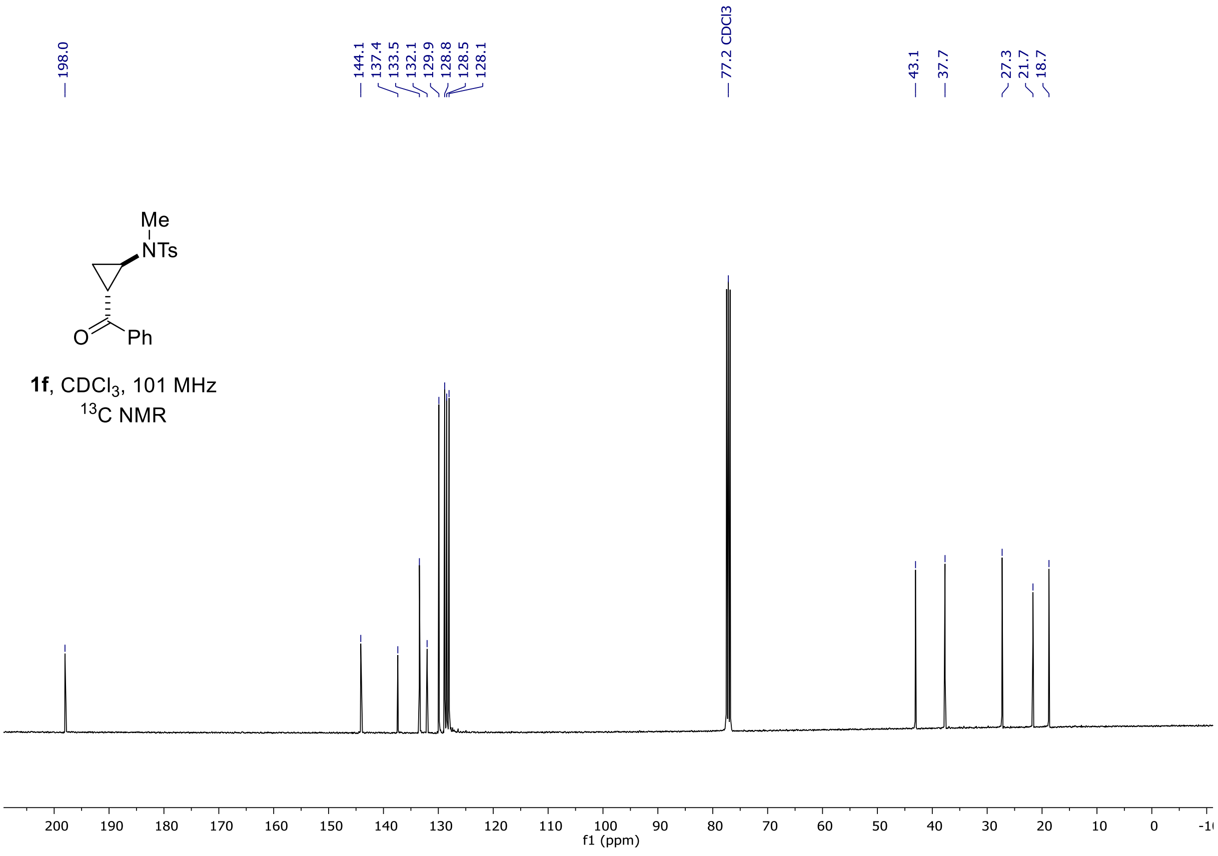
198.0
144.1 137.4 133.5 132.1 129.9 128.8 128.5 128.1
77.2 CDCl3
43.1 37.7 27.3 21.7 18.7
Me
NTs
O
Ph
1f, CDCl3, 101 MHz
13C NMR
f1 (ppm)

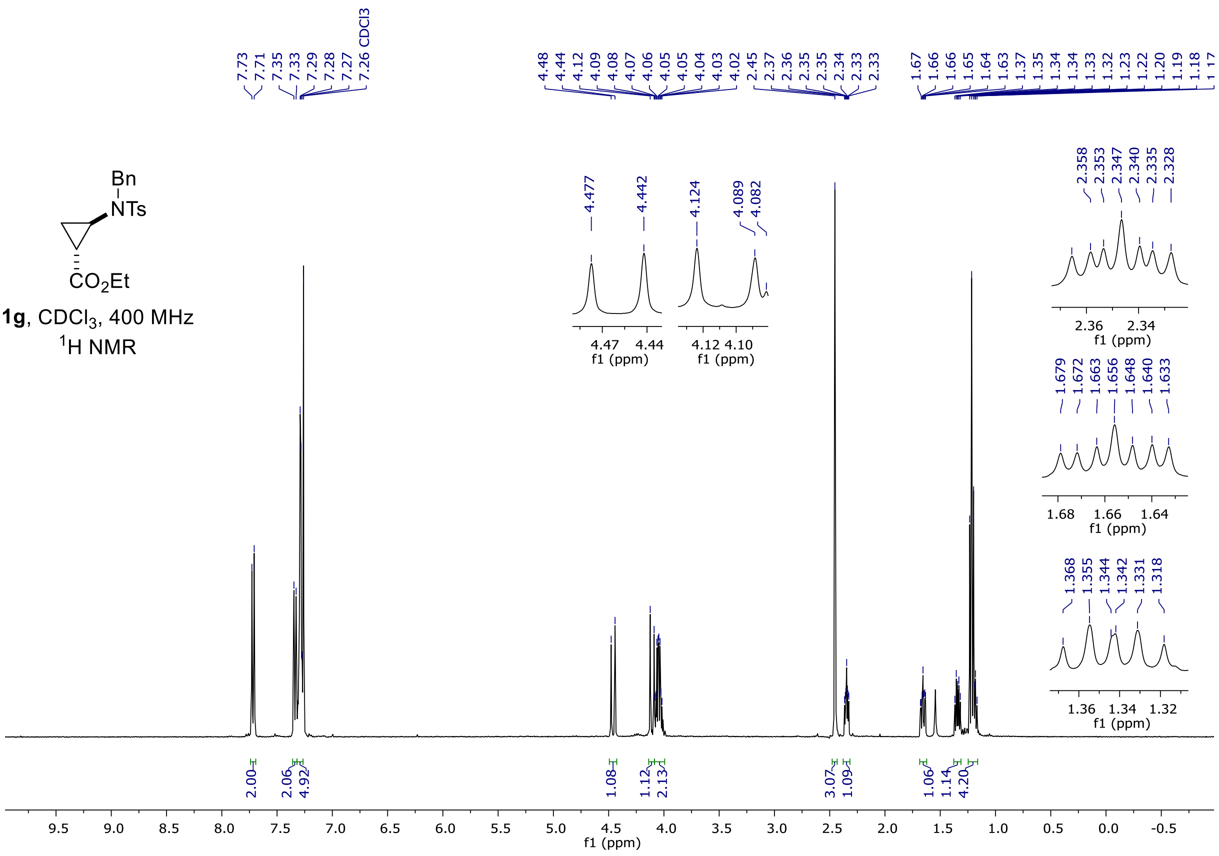

1g, $CDCl_3$, 400 MHz
^1H NMR

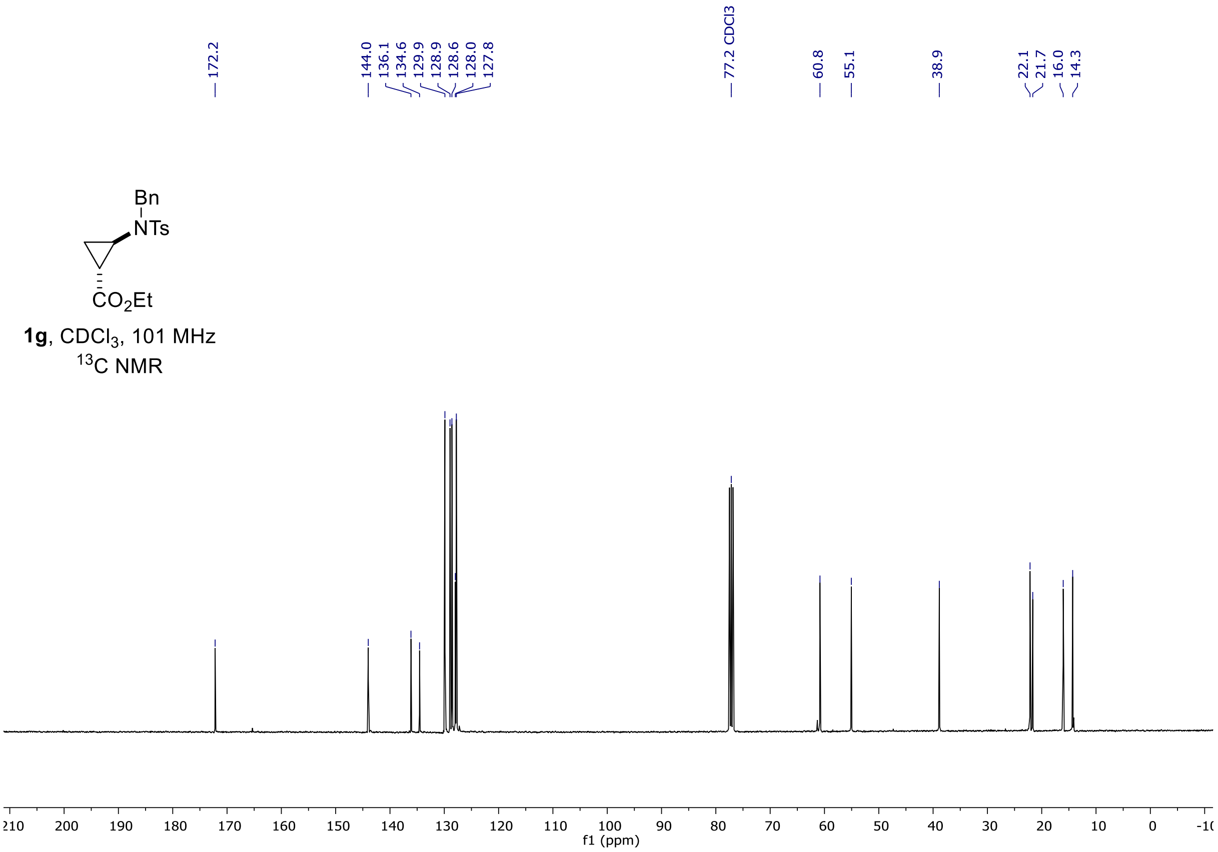

1g, $CDCl_3$, 101 MHz
^{13}C NMR

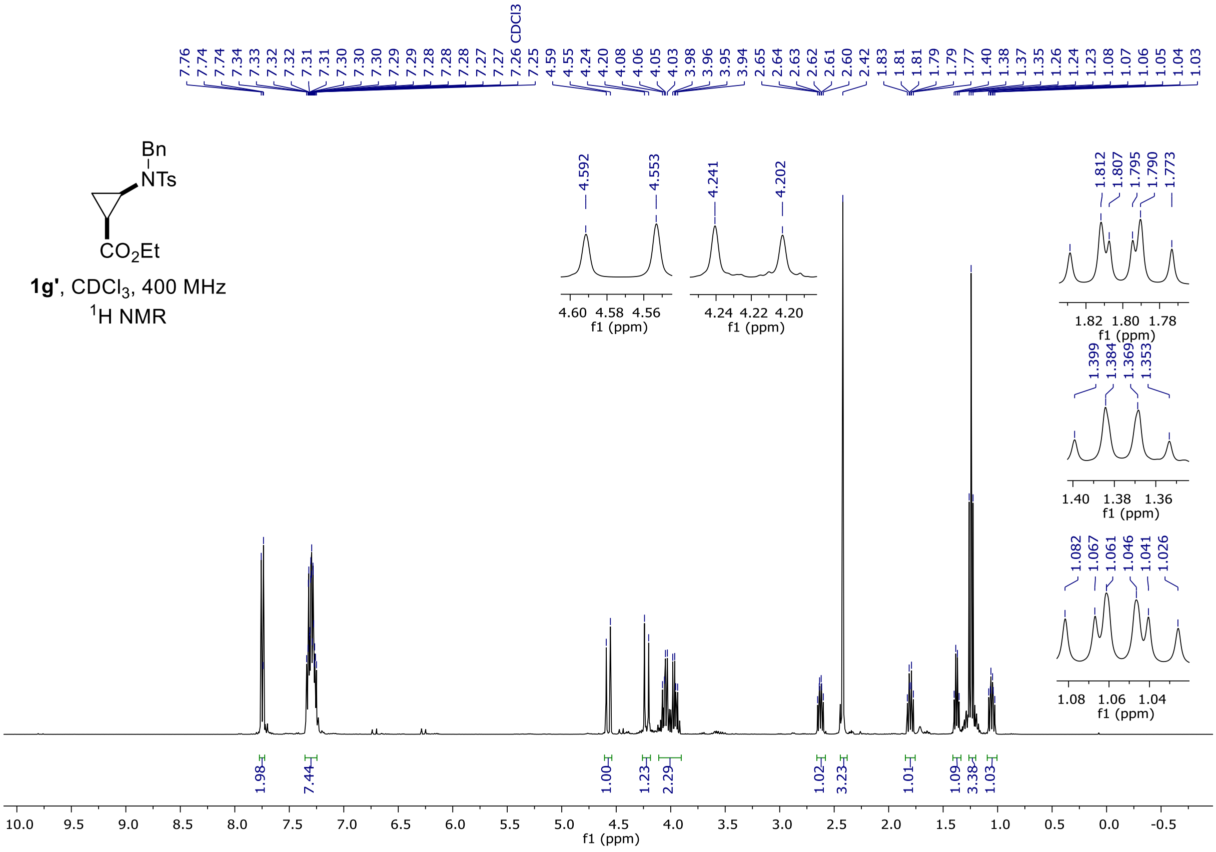

Bn
NTs
CO2Et
1g', CDCl3, 400 MHz
1H NMR
7.76 7.74 7.74 7.34 7.33 7.32 7.32 7.31 7.31 7.30 7.30 7.30 7.29 7.29 7.28 7.28 7.28 7.27 7.27 7.26 CDCl3 7.25 4.59 4.55 4.24 4.20 4.08 4.06 4.05 4.03 3.98 3.96 3.95 3.94 2.65 2.64 2.63 2.62 2.61 2.60 2.42 1.83 1.81 1.81 1.79 1.79 1.77 1.40 1.38 1.37 1.35 1.26 1.24 1.23 1.08 1.07 1.06 1.05 1.04 1.03
4.592 4.553 4.241 4.202
1.812 1.807 1.795 1.790 1.773
1.399 1.384 1.369 1.353
1.082 1.067 1.061 1.046 1.041 1.026
1.98 7.44 1.00 1.23 2.29 1.02 3.23 1.01 1.09 3.38 1.03
f1 (ppm)

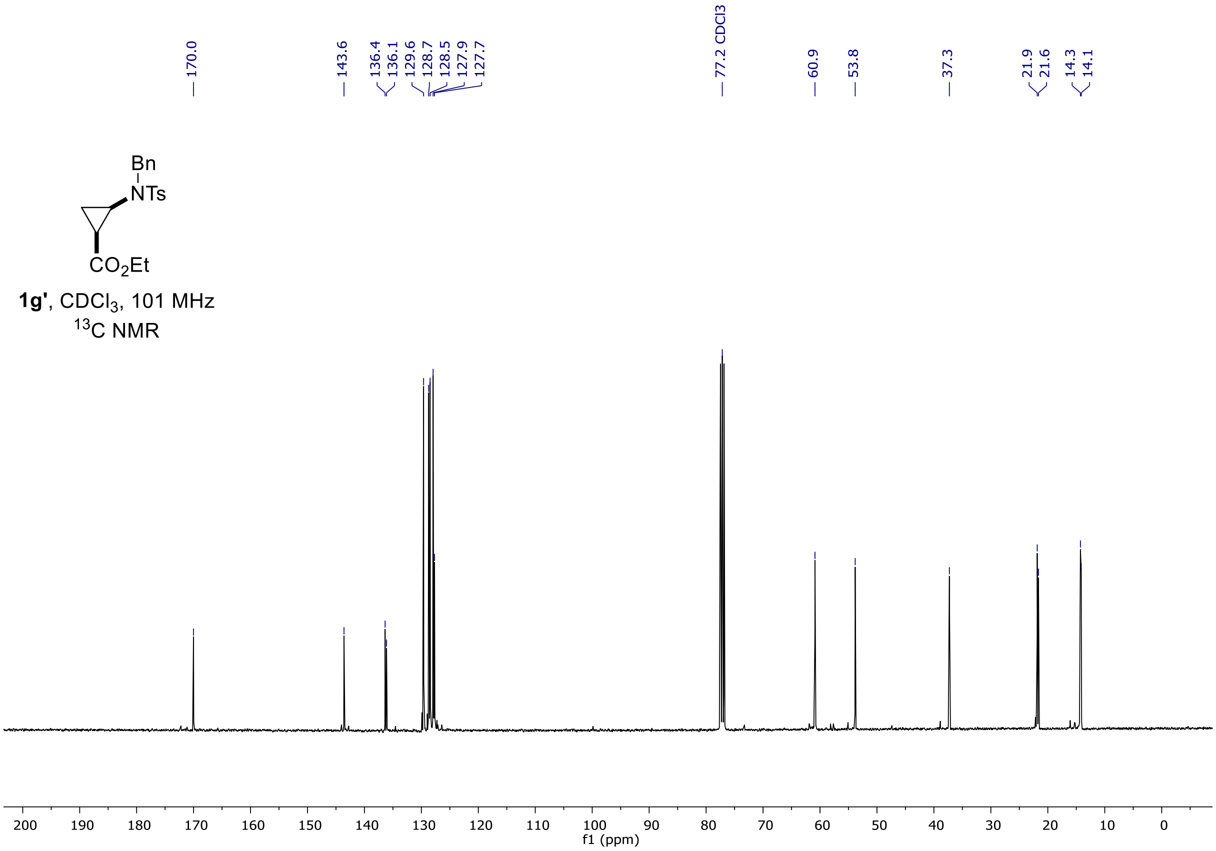

170.0 143.6 136.4 136.1 129.6 128.7 128.5 127.9 127.7 77.2 CDCl3 60.9 53.8 37.3 21.9 21.6 14.3 14.1
Bn
NTs
CO2Et
1g', CDCl3, 101 MHz
13C NMR
f1 (ppm)

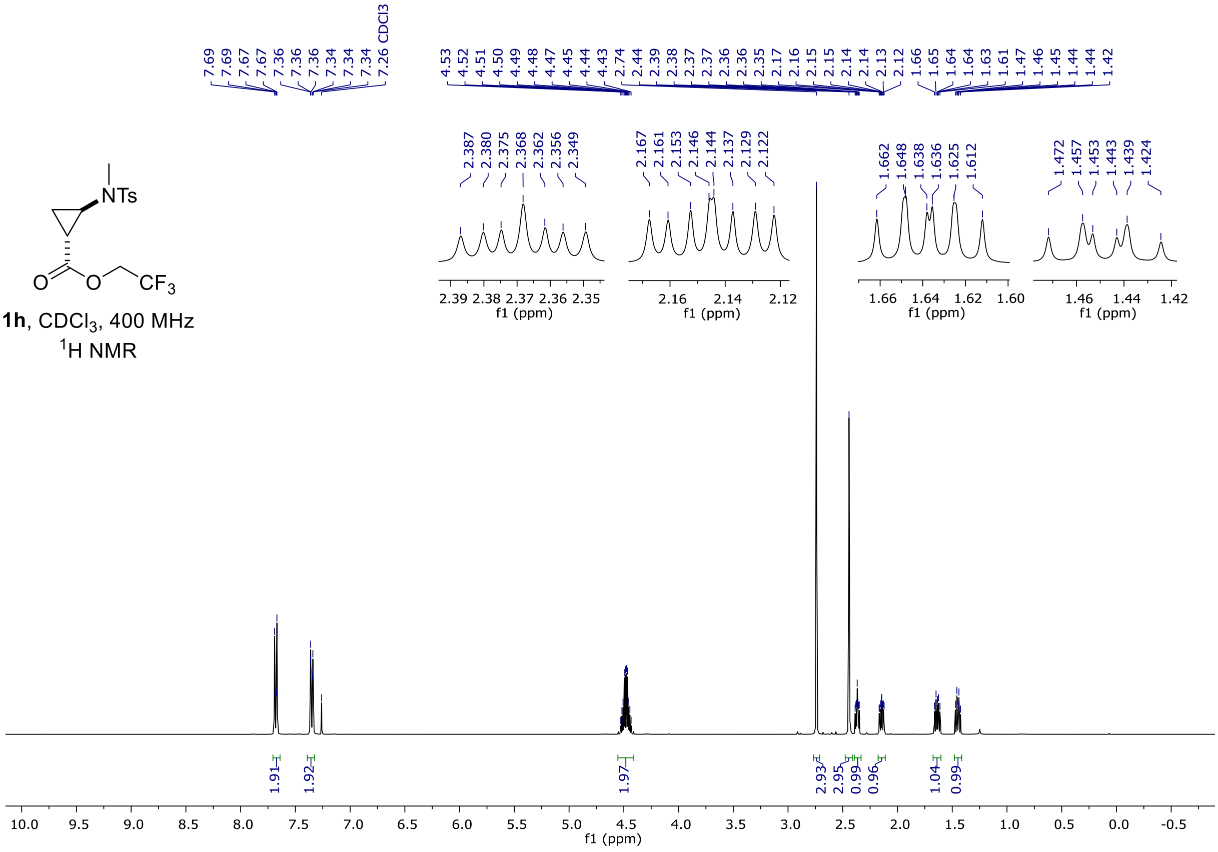

1h, $CDCl_3$, 400 MHz
^{1}H NMR

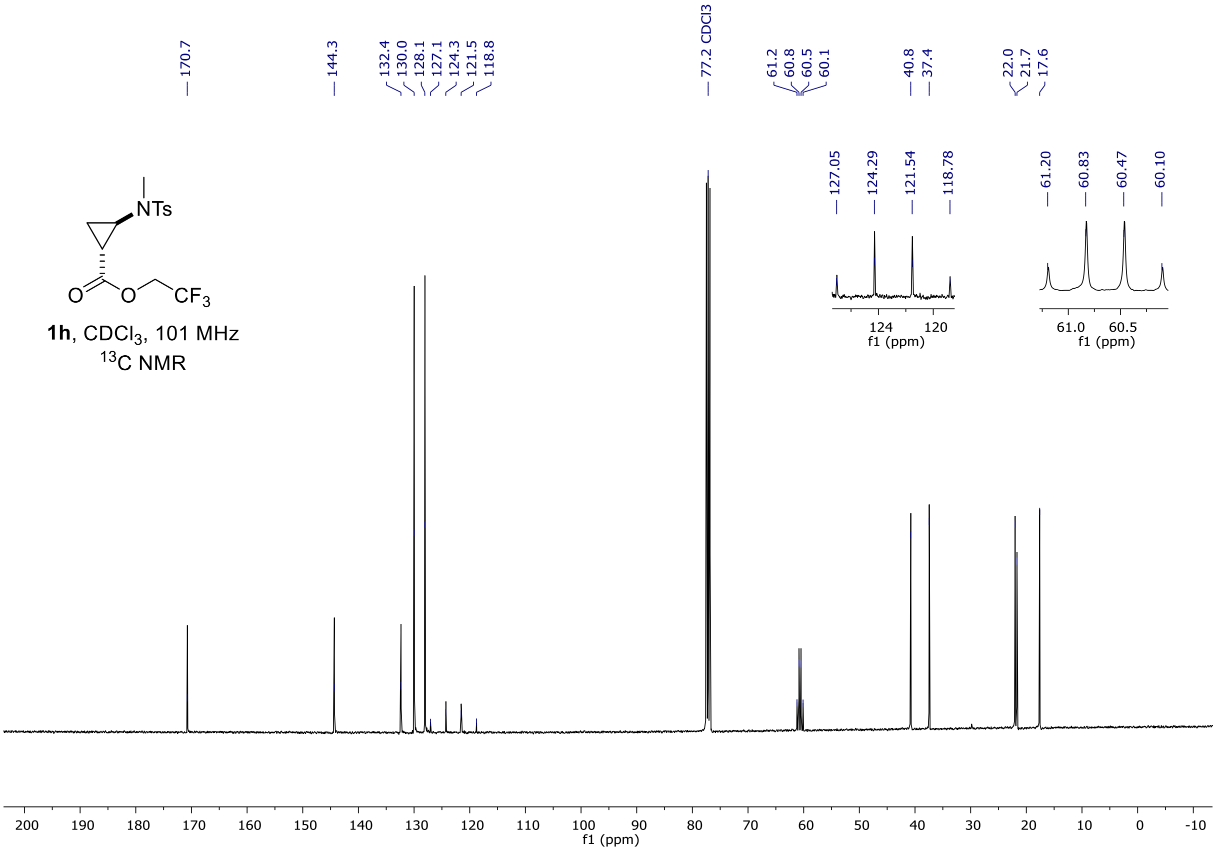

1h, $CDCl_3$, 101 MHz
^{13}C NMR

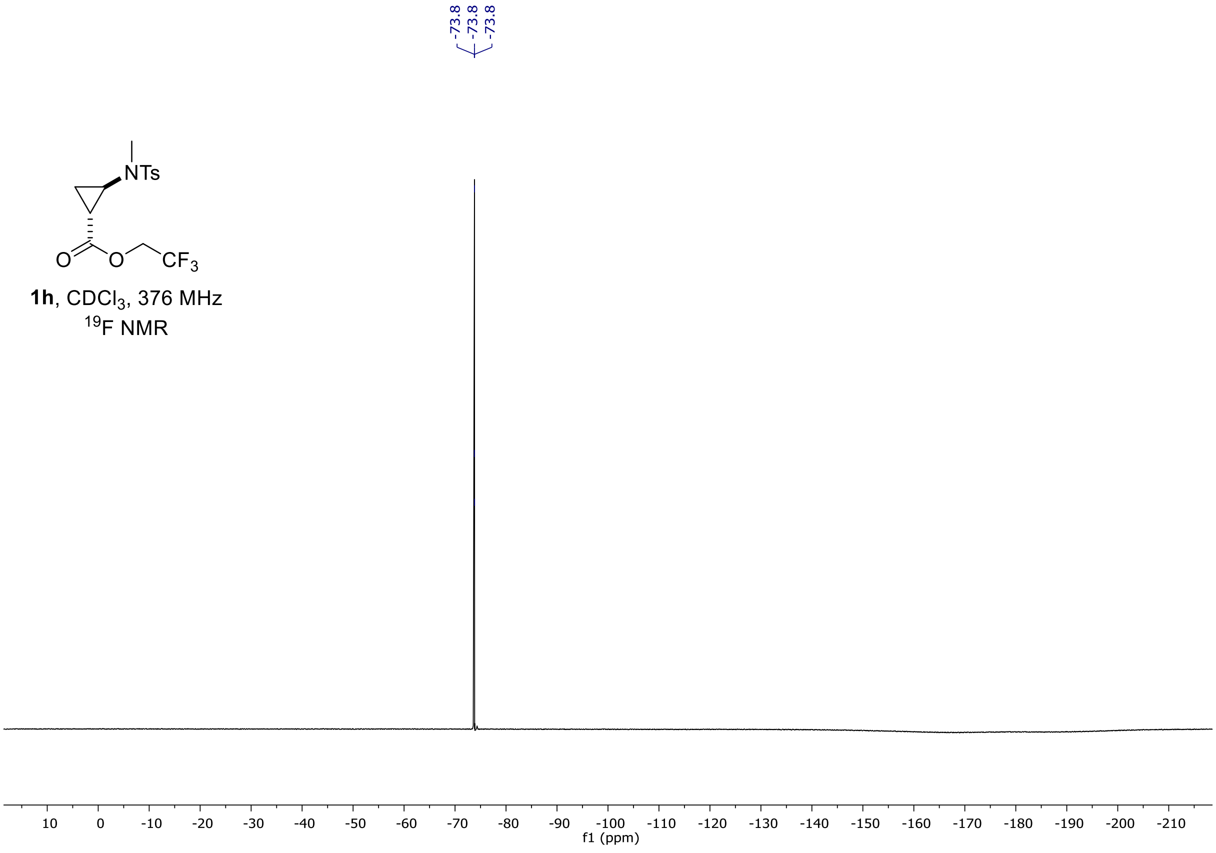

1h, $CDCl_3$, 376 MHz
^{19}F NMR

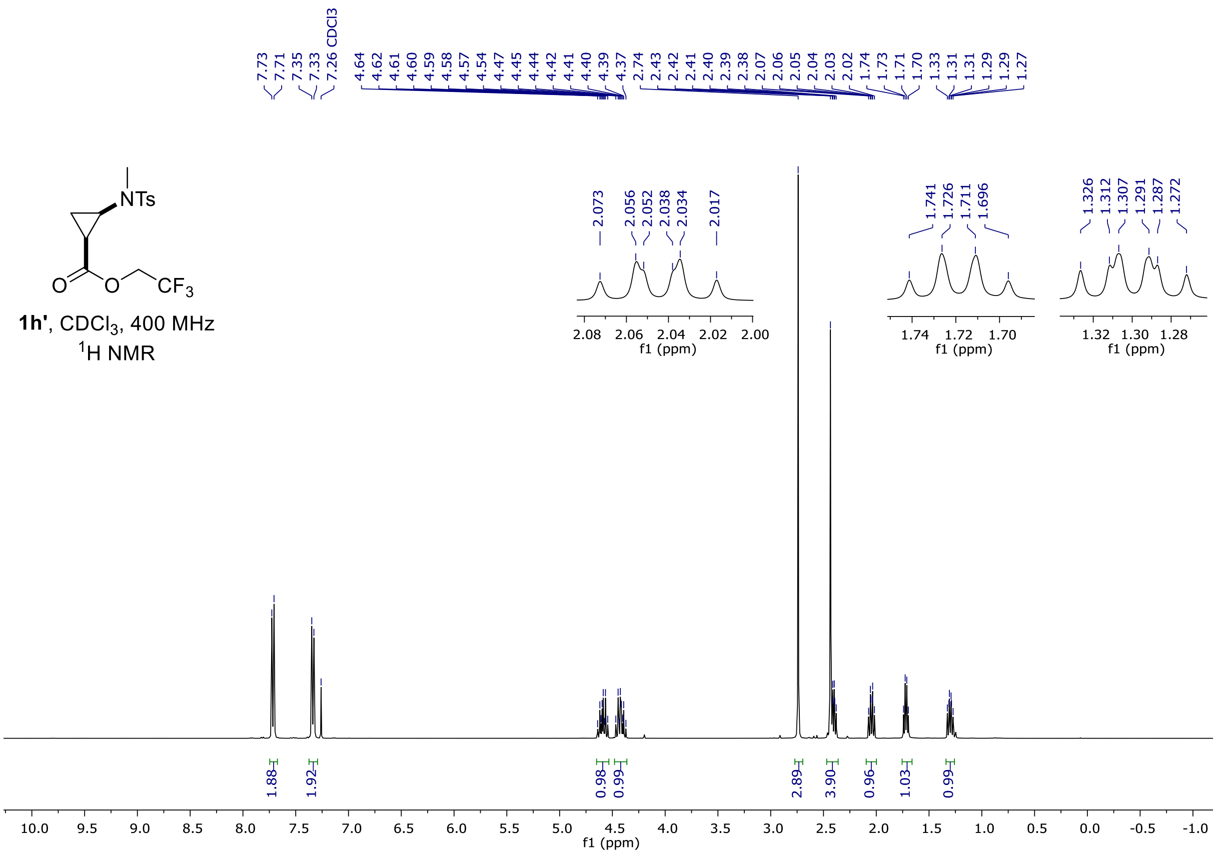
1h', CDCl3, 400 MHz
1H NMR
7.73
7.71
7.35
7.33
7.26 CDCl3
4.64
4.62
4.61
4.60
4.59
4.58
4.57
4.54
4.47
4.45
4.44
4.42
4.41
4.40
4.39
4.37
2.74
2.43
2.42
2.41
2.40
2.39
2.38
2.07
2.06
2.05
2.04
2.03
2.02
1.74
1.73
1.71
1.70
1.33
1.31
1.31
1.29
1.29
1.27
2.073
2.056
2.052
2.038
2.034
2.017
1.741
1.726
1.711
1.696
1.326
1.312
1.307
1.291
1.287
1.272
1.88
1.92
0.98
0.99
2.89
3.90
0.96
1.03
0.99
f1 (ppm)

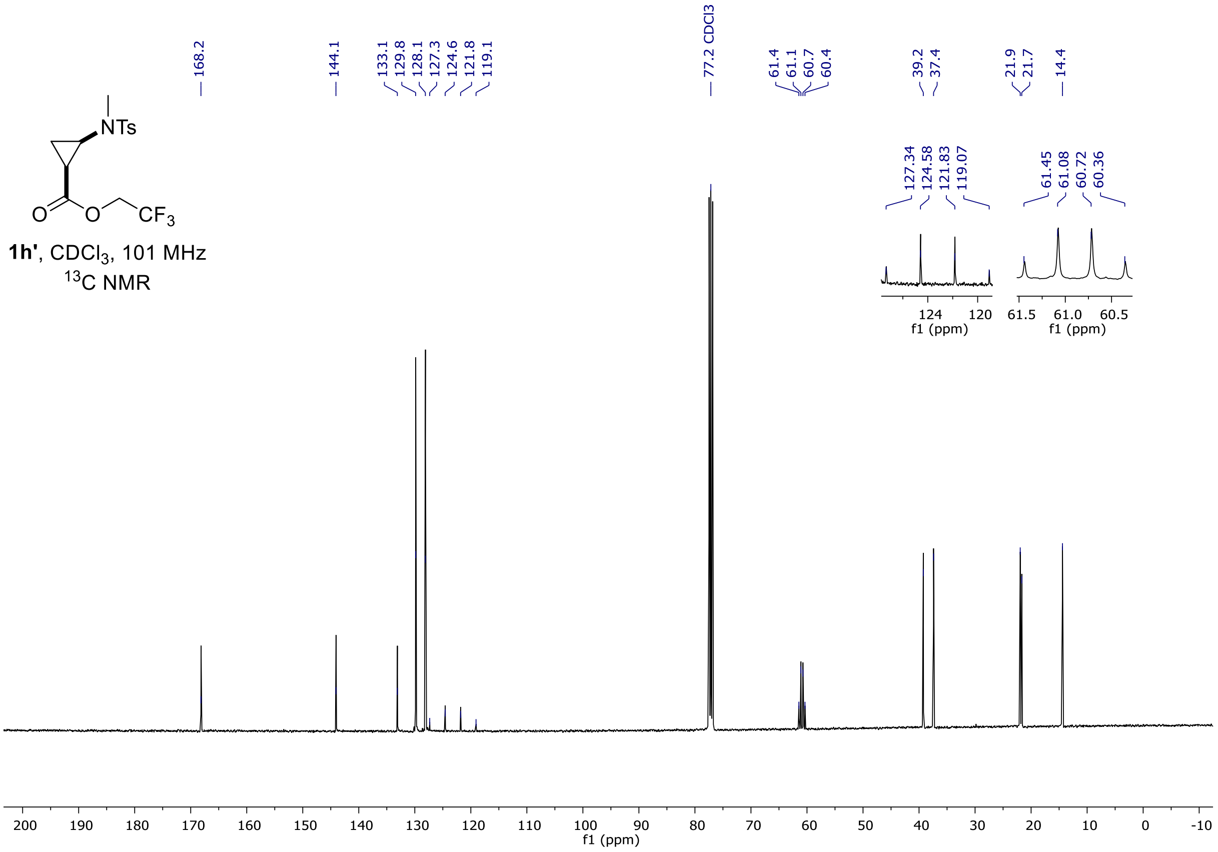
1h', CDCl3, 101 MHz
13C NMR
168.2
144.1
133.1
129.8
128.1
127.3
124.6
121.8
119.1
77.2 CDCl3
61.4
61.1
60.7
60.4
39.2
37.4
21.9
21.7
14.4
127.34
124.58
121.83
119.07
61.45
61.08
60.72
60.36
f1 (ppm)

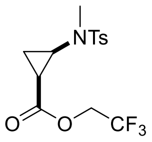

1h', $CDCl_3$, 376 MHz
^{19}F NMR

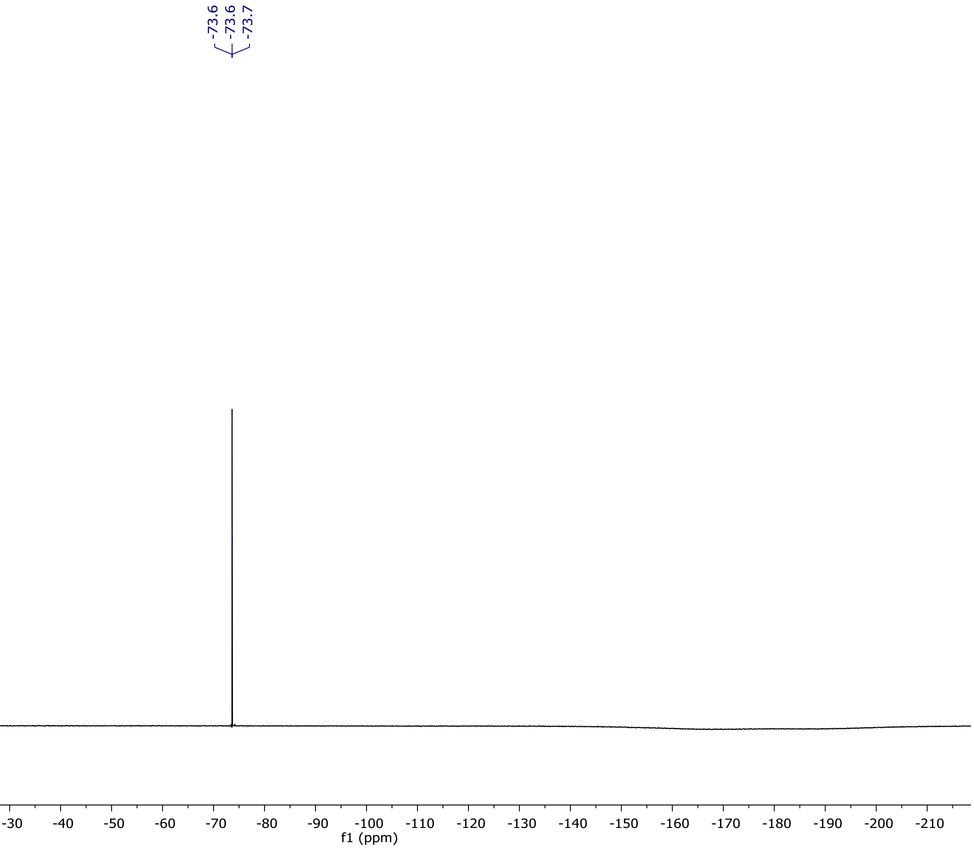

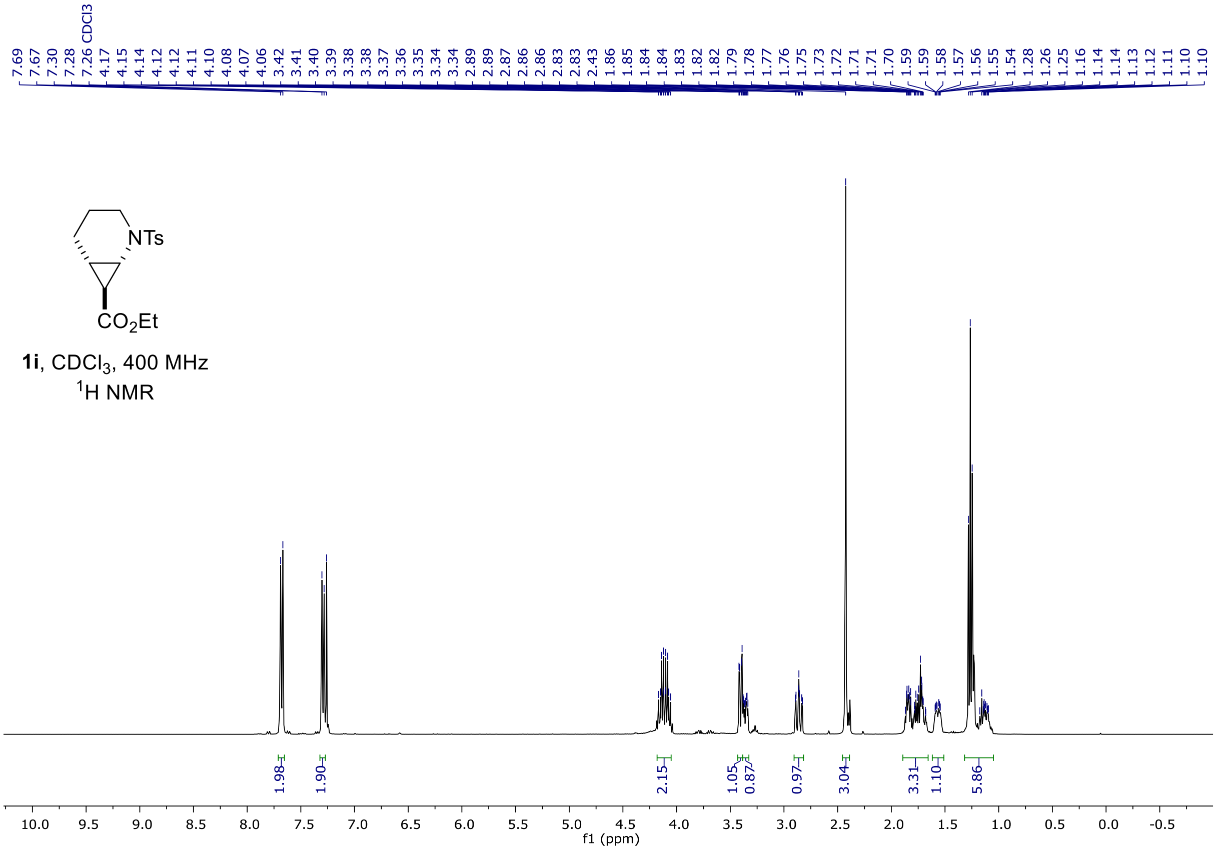
7.69 7.67 7.30 7.28 7.26 CDCl3 4.17 4.15 4.14 4.12 4.12 4.11 4.10 4.08 4.07 4.06 3.42 3.41 3.40 3.39 3.38 3.38 3.37 3.36 3.35 3.34 3.34 2.89 2.89 2.87 2.86 2.86 2.83 2.43 1.86 1.85 1.84 1.84 1.83 1.82 1.79 1.78 1.77 1.76 1.75 1.73 1.72 1.71 1.71 1.70 1.59 1.59 1.58 1.57 1.56 1.55 1.54 1.28 1.26 1.25 1.16 1.14 1.14 1.13 1.12 1.11 1.10 1.10
NTs
CO2Et
1i, CDCl3, 400 MHz
1H NMR
1.98 1.90 2.15 1.05 0.87 0.97 3.04 3.31 1.10 5.86
10.0 9.5 9.0 8.5 8.0 7.5 7.0 6.5 6.0 5.5 5.0 4.5 4.0 3.5 3.0 2.5 2.0 1.5 1.0 0.5 0.0 -0.5
f1 (ppm)

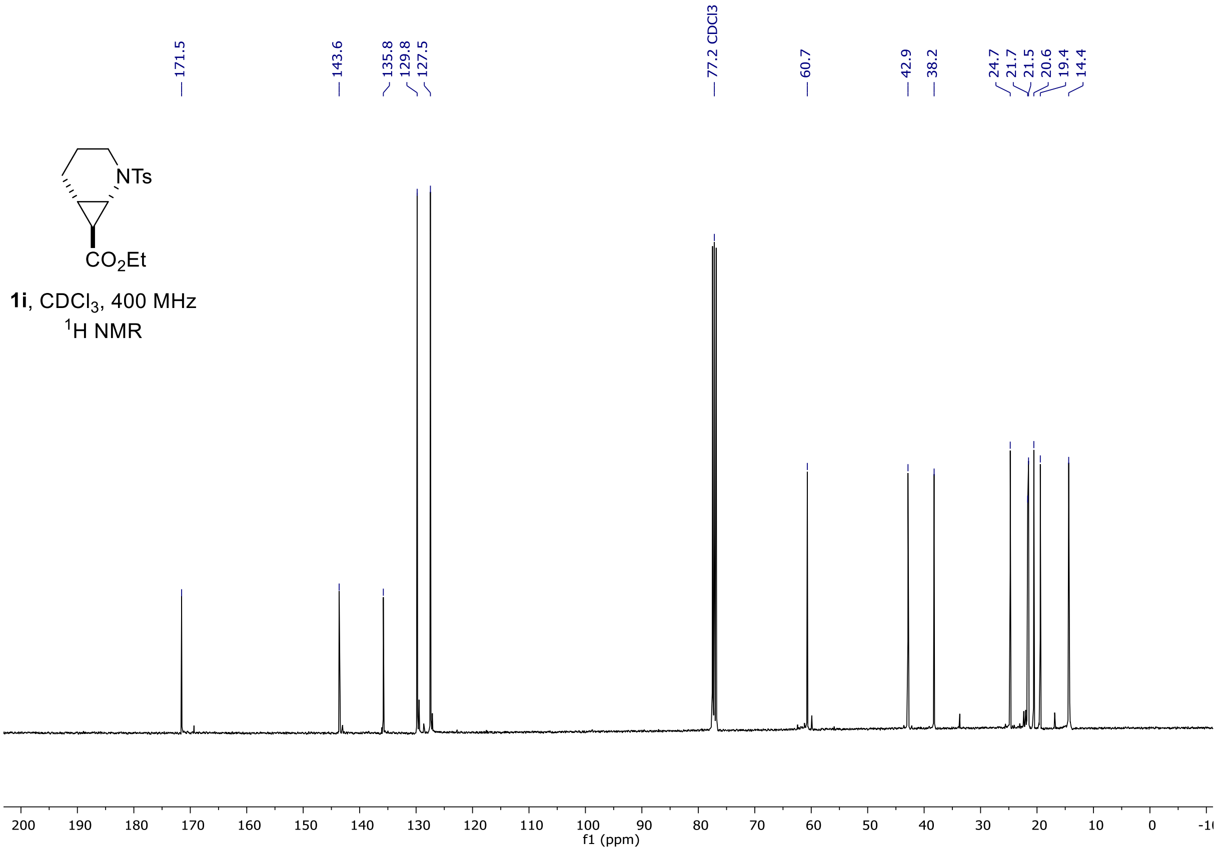
171.5 143.6 135.8 129.8 127.5 77.2 CDCl3 60.7 42.9 38.2 24.7 21.7 21.5 20.6 19.4 14.4
NTs
CO2Et
1i, CDCl3, 400 MHz
1H NMR
200 190 180 170 160 150 140 130 120 110 100 90 80 70 60 50 40 30 20 10 0
f1 (ppm)

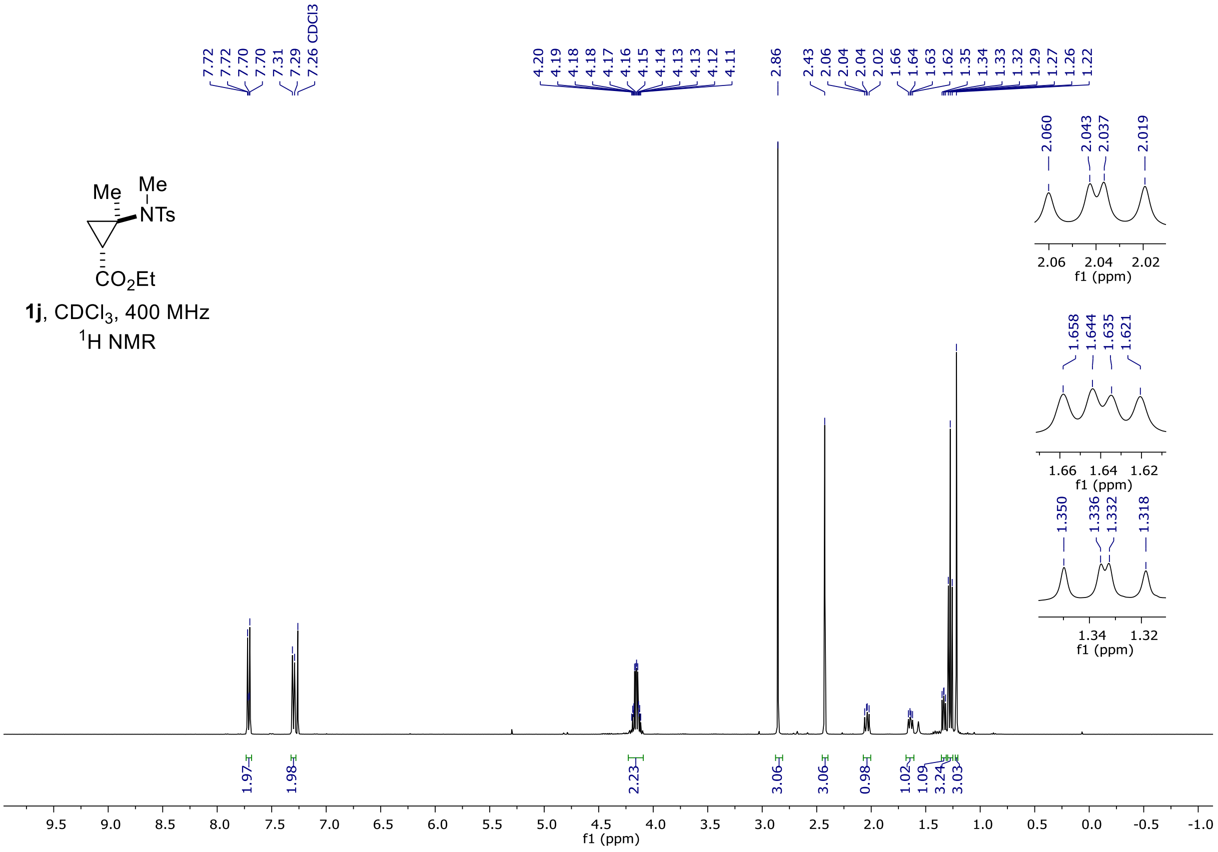
Me
Me
NTs
CO_2Et
1j, $CDCl_3$, 400 MHz
^1H NMR
7.72
7.72
7.70
7.70
7.31
7.29
7.26 CDCl3
4.20
4.19
4.18
4.18
4.17
4.16
4.15
4.14
4.13
4.13
4.12
4.11
2.86
2.43
2.06
2.04
2.04
2.02
1.66
1.64
1.63
1.62
1.35
1.34
1.33
1.32
1.29
1.27
1.26
1.22
2.060
2.043
2.037
2.019
1.658
1.644
1.635
1.621
1.350
1.336
1.332
1.318
1.97
1.98
2.23
3.06
3.06
0.98
1.02
1.09
3.24
3.03
f1 (ppm)

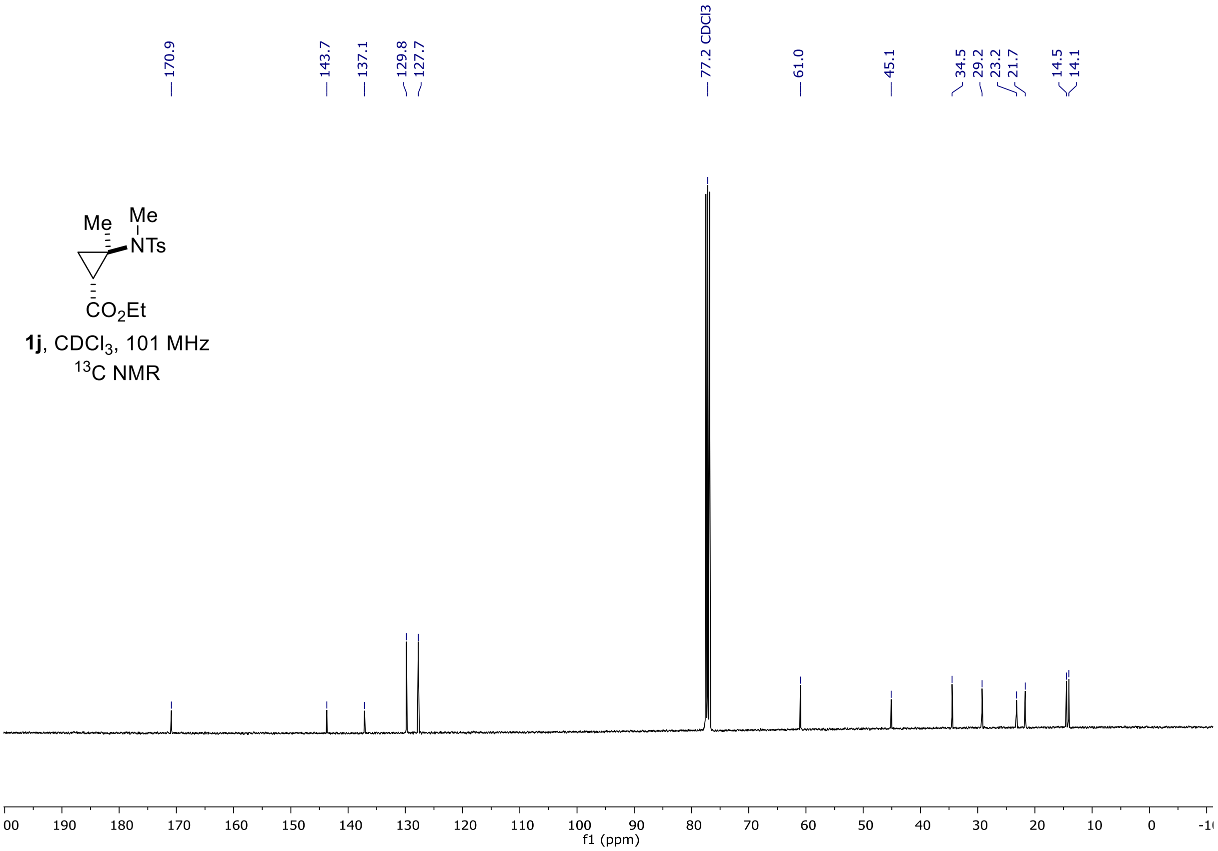
Me
Me
NTs
CO_2Et
1j, $CDCl_3$, 101 MHz
^{13}C NMR
170.9
143.7
137.1
129.8
127.7
77.2 CDCl3
61.0
45.1
34.5
29.2
23.2
21.7
14.5
14.1
f1 (ppm)

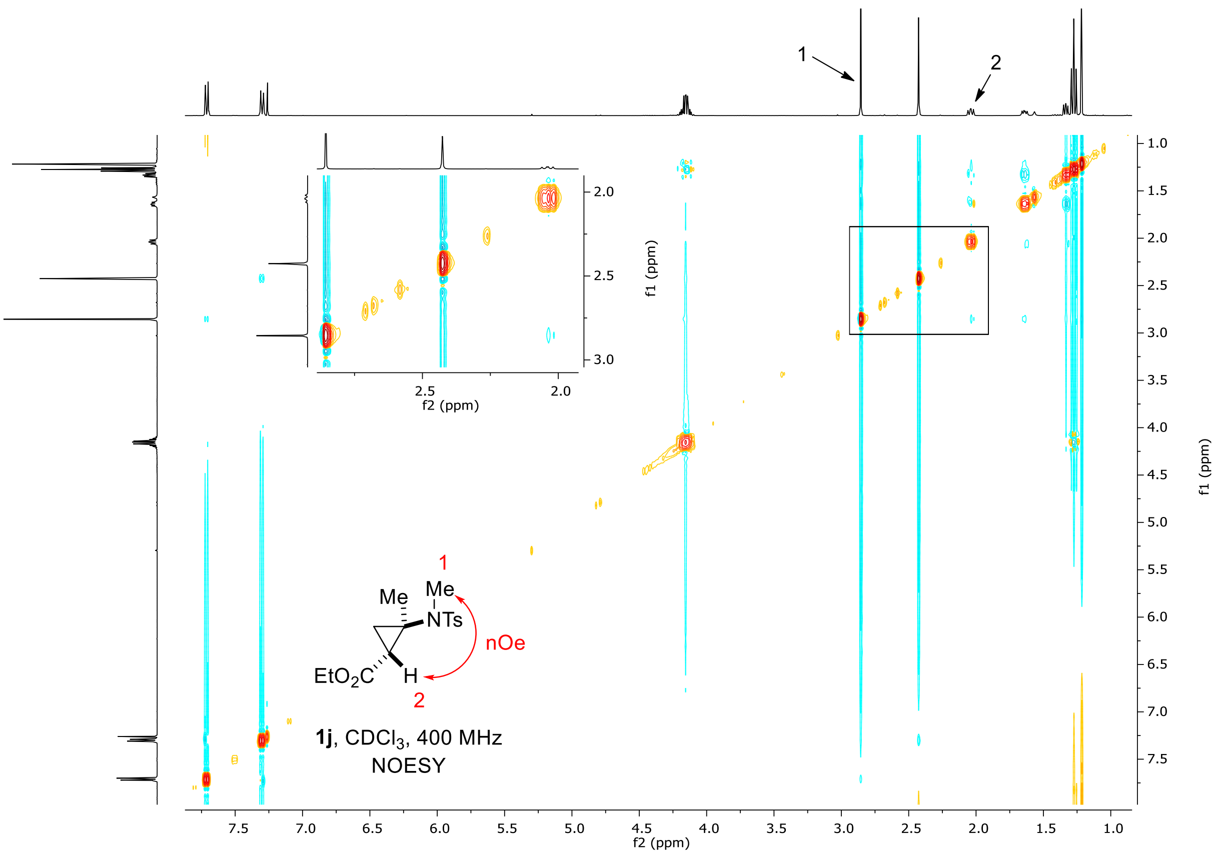

1
2
f1 (ppm)
f2 (ppm)
Me
Me
NTs
nOe
EtO2C
H
1j, CDCl3, 400 MHz
NOESY

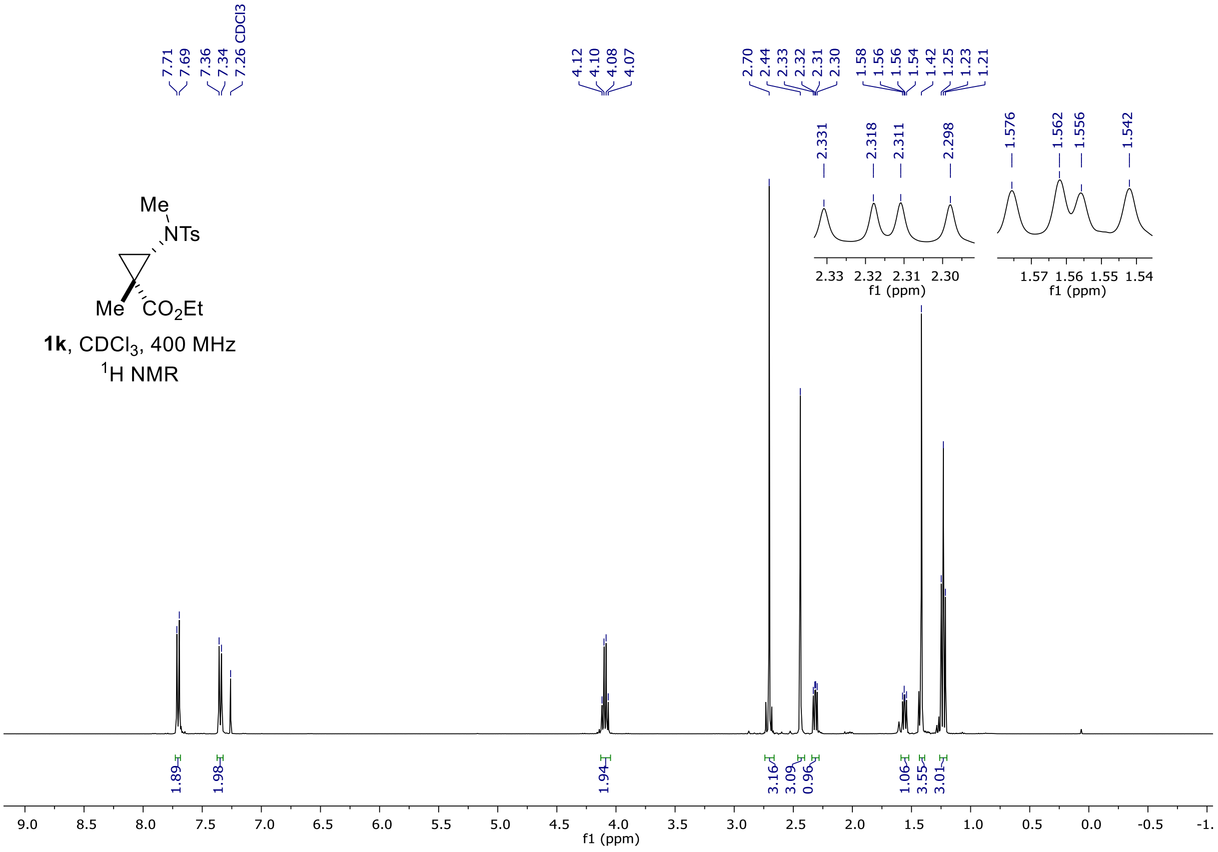

1k, $CDCl_3$, 400 MHz
^1H NMR

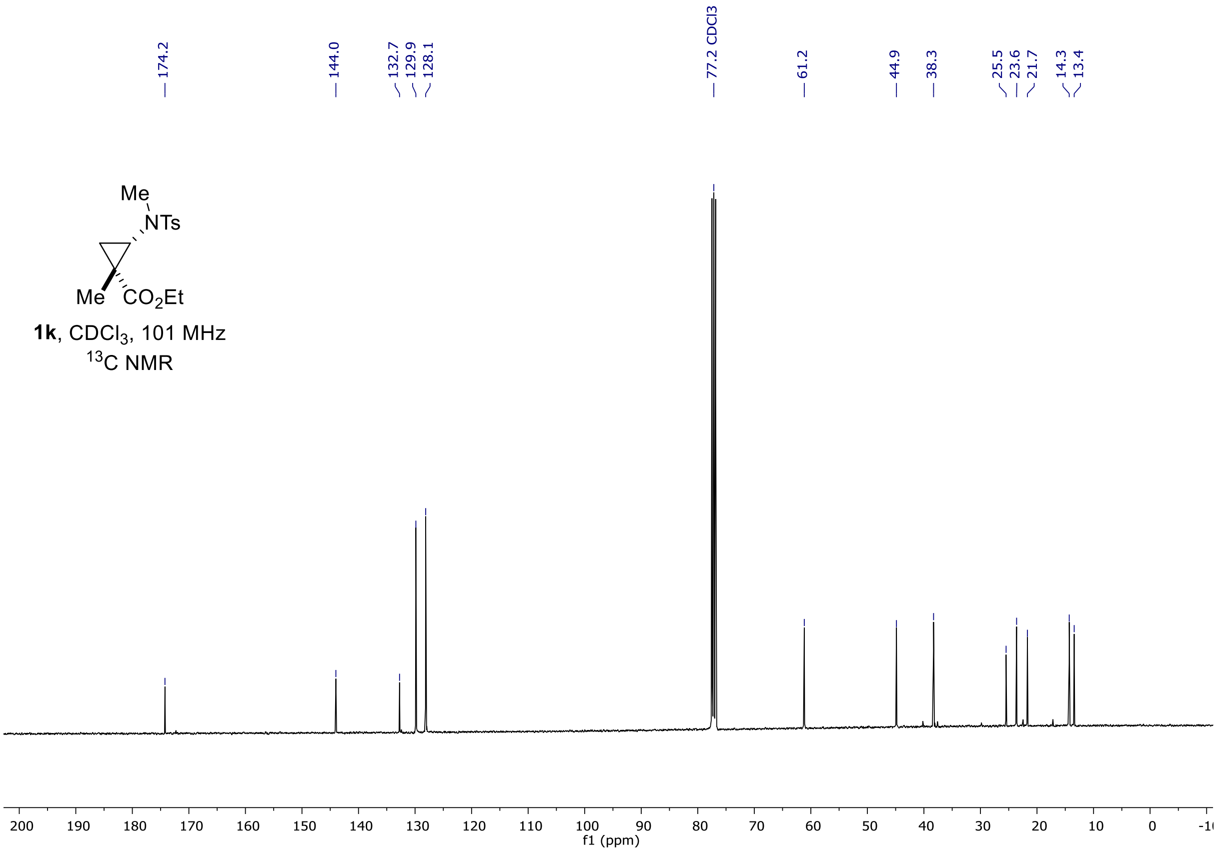

1k, $CDCl_3$, 101 MHz
^{13}C NMR

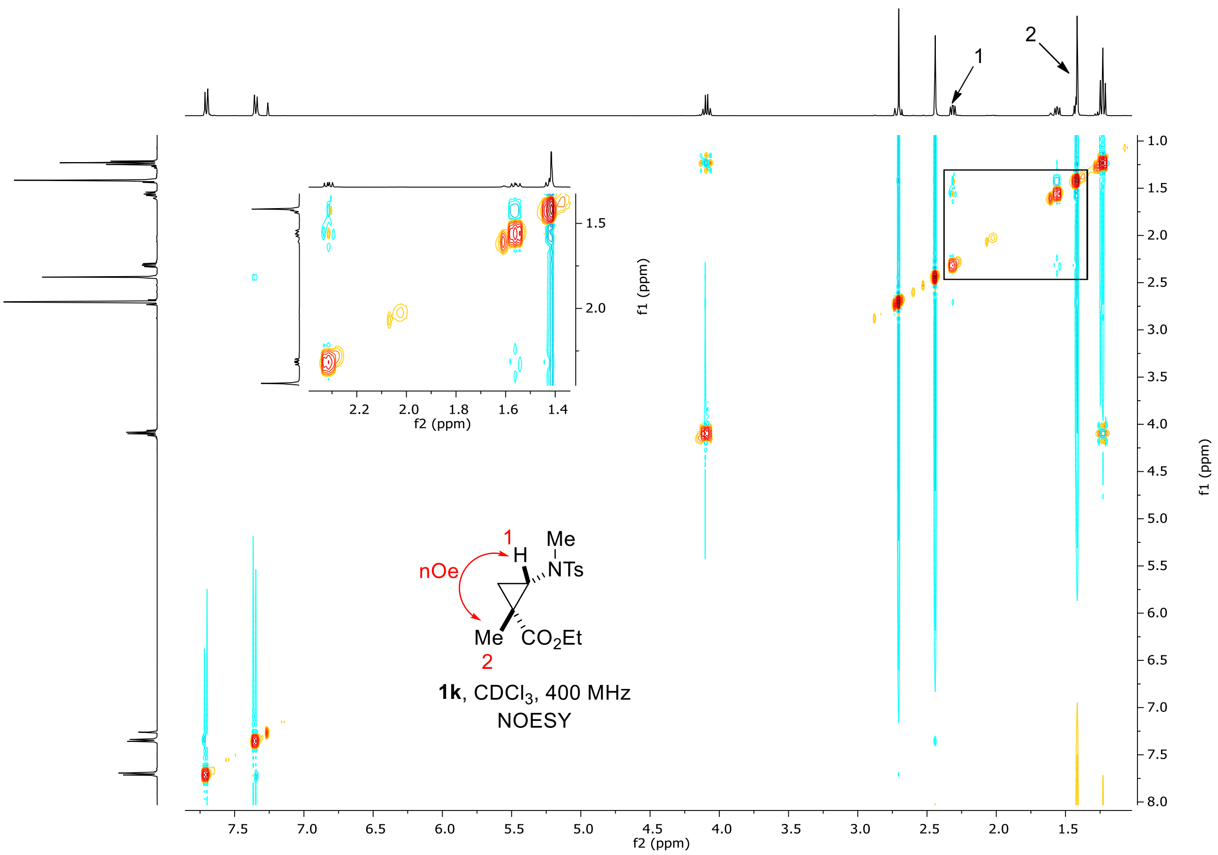

nOe
1
H
Me
NTs
Me
CO2Et
2
1k, CDCl3, 400 MHz
NOESY
f2 (ppm)
f1 (ppm)

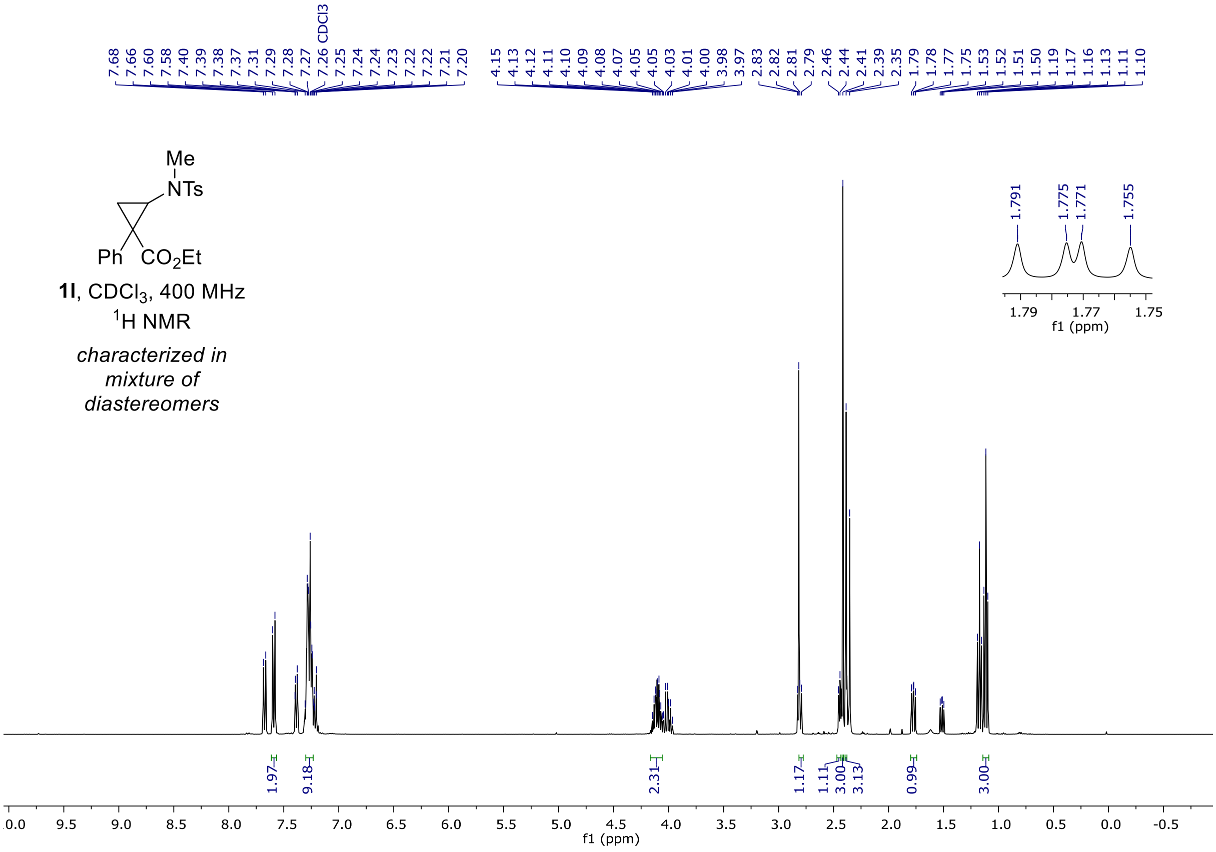
7.68 7.66 7.60 7.58 7.40 7.39 7.38 7.37 7.31 7.29 7.28 7.27 7.26 CDCl3 7.25 7.24 7.24 7.23 7.22 7.22 7.21 7.20 4.15 4.13 4.12 4.11 4.10 4.09 4.08 4.07 4.05 4.05 4.03 4.01 4.00 3.98 3.97 2.83 2.82 2.81 2.79 2.46 2.44 2.41 2.39 2.35 1.79 1.78 1.77 1.75 1.53 1.52 1.51 1.50 1.19 1.17 1.16 1.13 1.11 1.10
Me
NTs
Ph
CO2Et
1l, CDCl3, 400 MHz
1H NMR
characterized in mixture of diastereomers
1.791 1.775 1.771 1.755
f1 (ppm)
1.97 9.18 2.31 1.17 1.11 3.00 3.13 0.99 3.00
f1 (ppm)

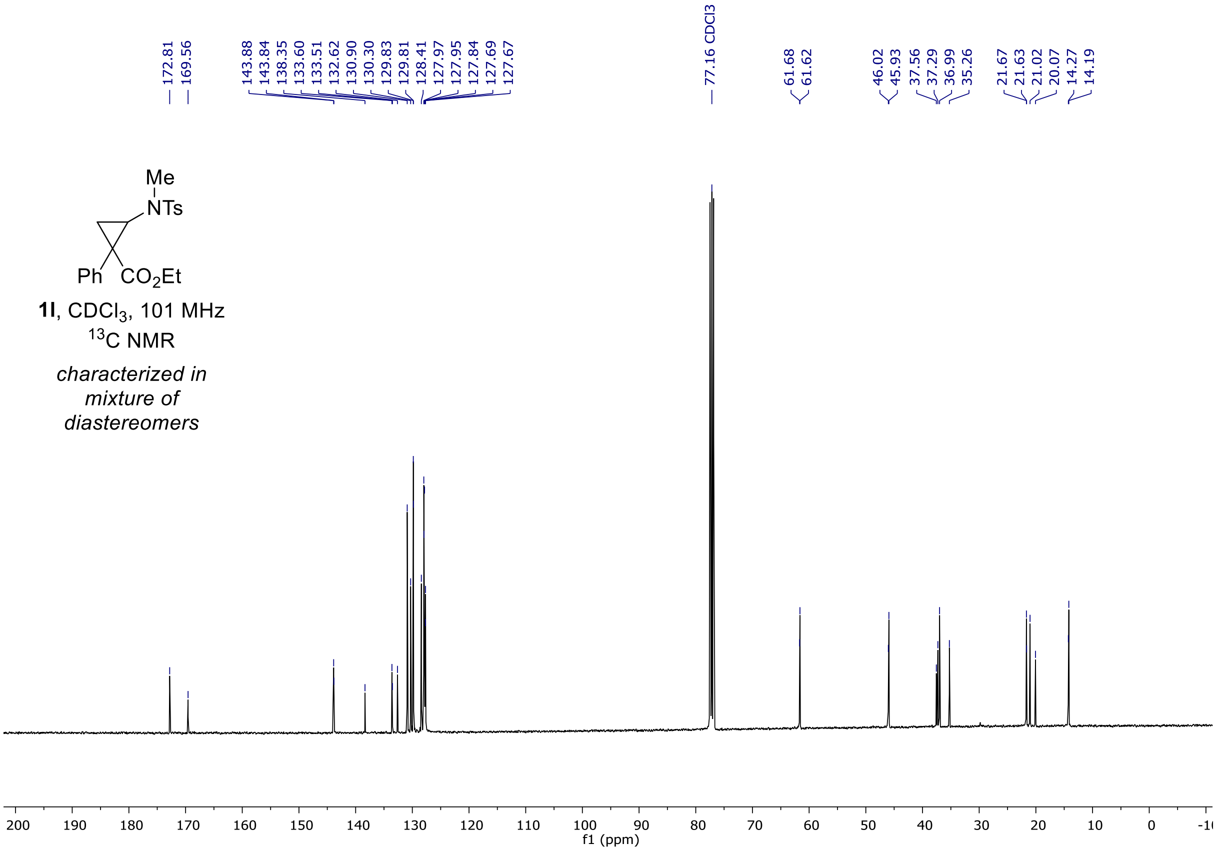
172.81 169.56 143.88 143.84 138.35 133.60 133.51 132.62 130.90 130.30 129.83 129.81 128.41 127.97 127.95 127.84 127.69 127.67 77.16 CDCl3 61.68 61.62 46.02 45.93 37.56 37.29 36.99 35.26 21.67 21.63 21.02 20.07 14.27 14.19
Me
NTs
Ph
CO2Et
1l, CDCl3, 101 MHz
13C NMR
characterized in mixture of diastereomers
f1 (ppm)

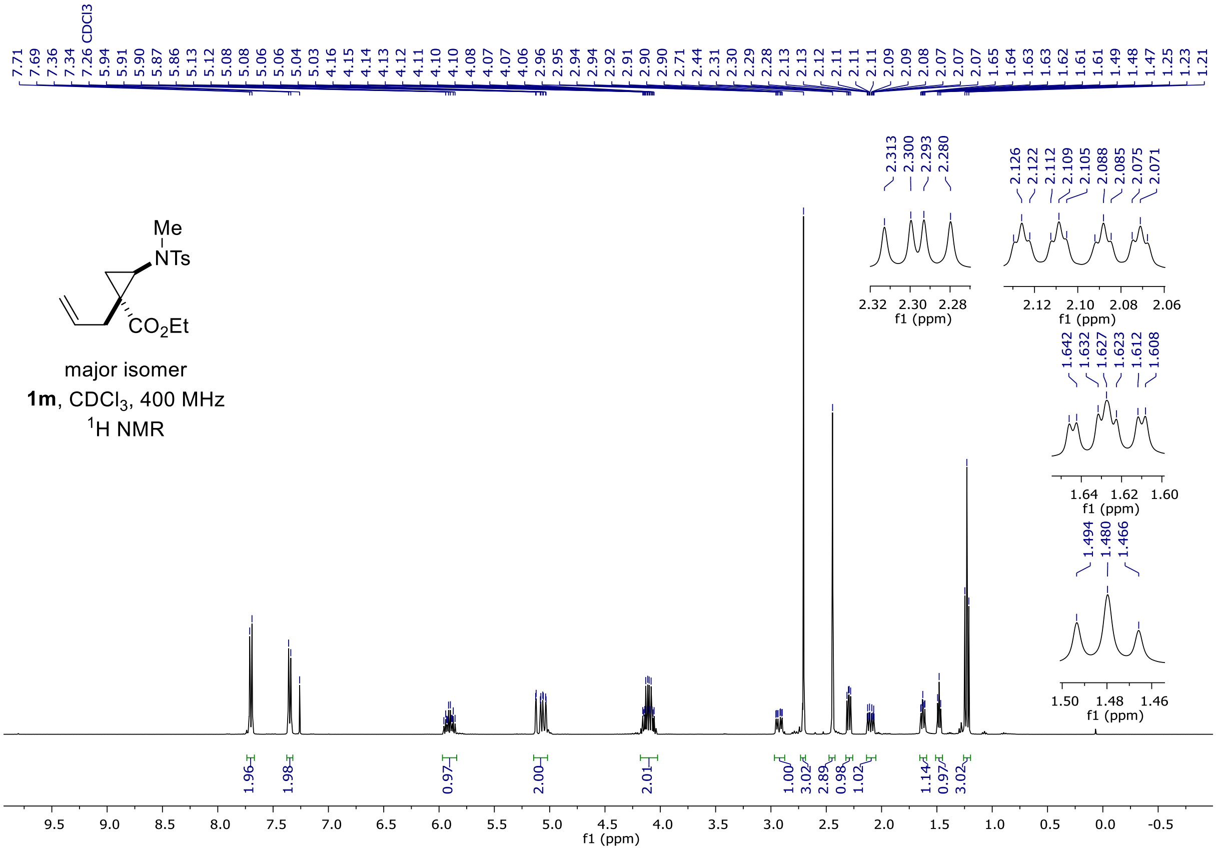
Me
NTs
CO2Et
major isomer
1m, CDCl3, 400 MHz
1H NMR
f1 (ppm)

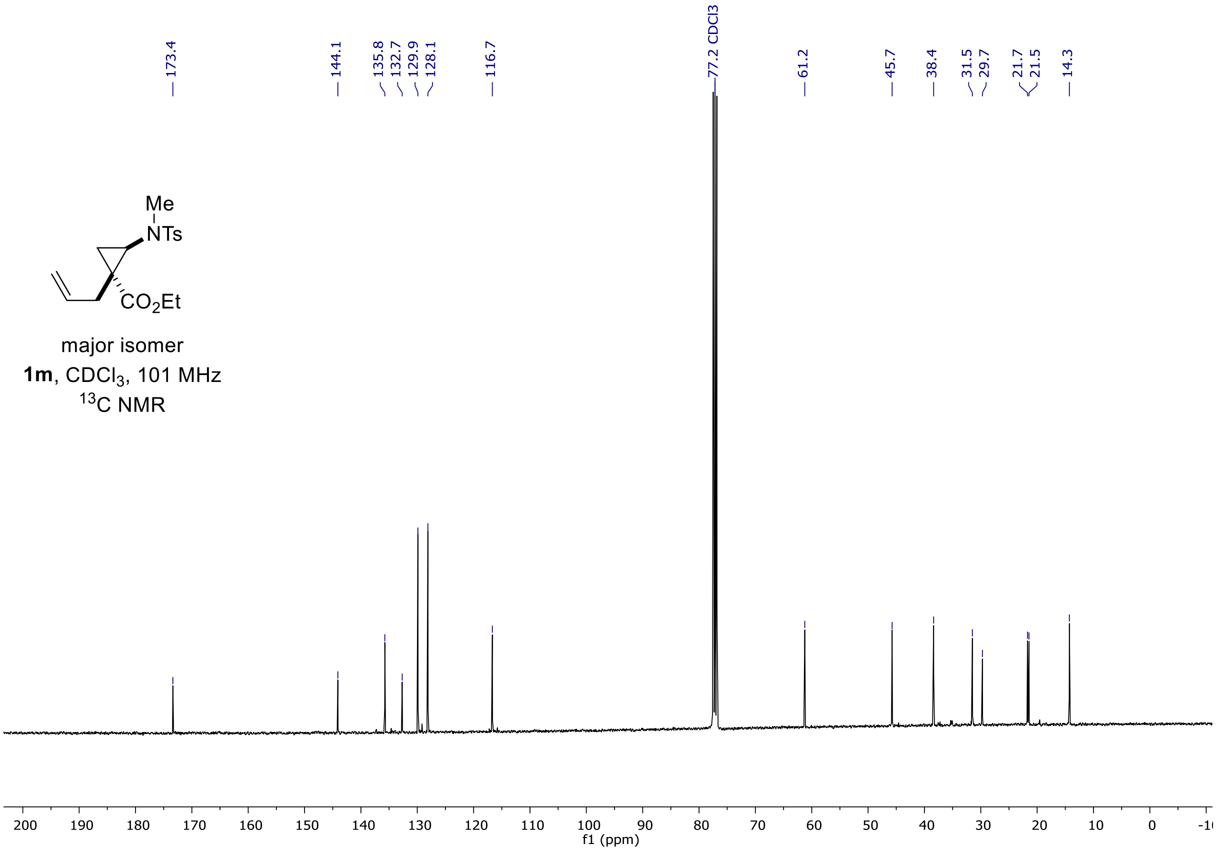
Me
NTs
CO2Et
major isomer
1m, CDCl3, 101 MHz
13C NMR
f1 (ppm)

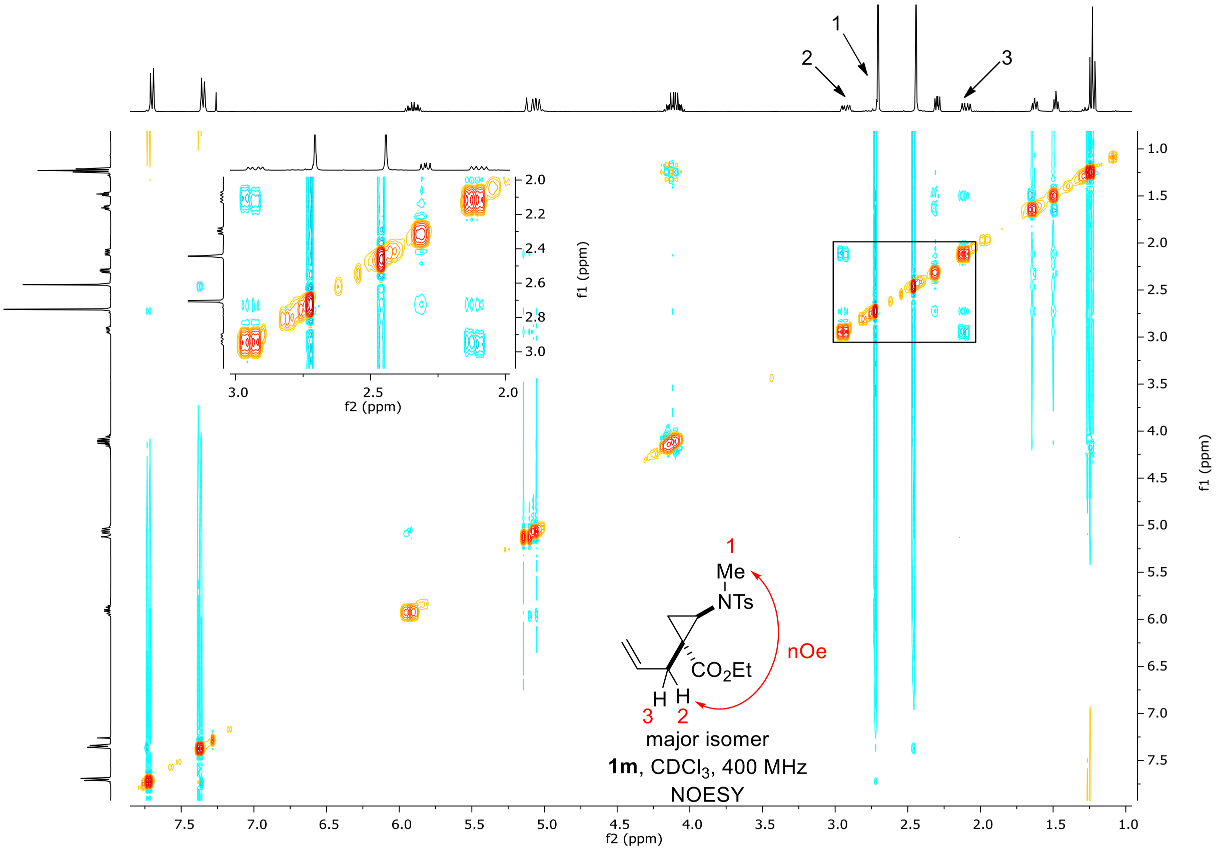

1
2
3
Me
NTs
CO2Et
nOe
H H
3 2
major isomer
1m, CDCl3, 400 MHz
NOESY
f2 (ppm)
f1 (ppm)

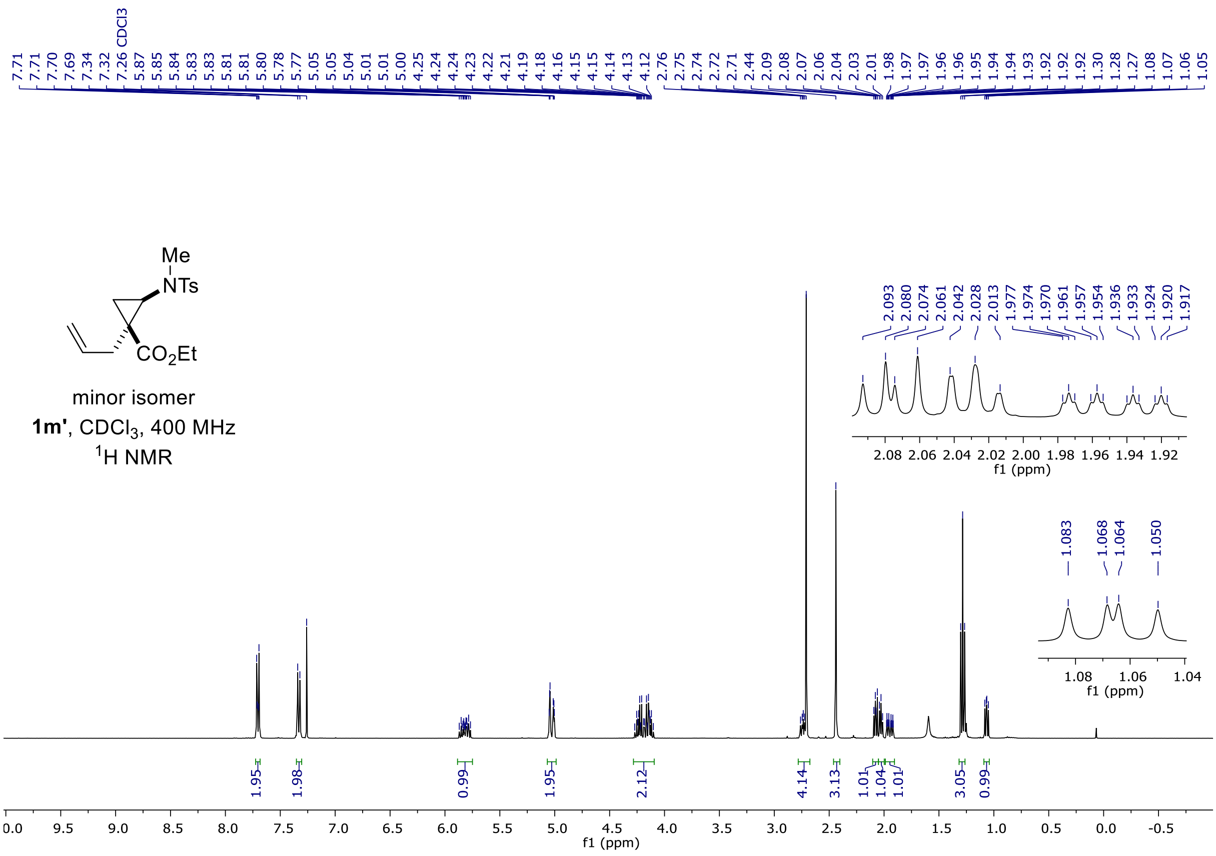

Me
NTs
CO2Et
minor isomer
1m', CDCl3, 400 MHz
1H NMR
f1 (ppm)

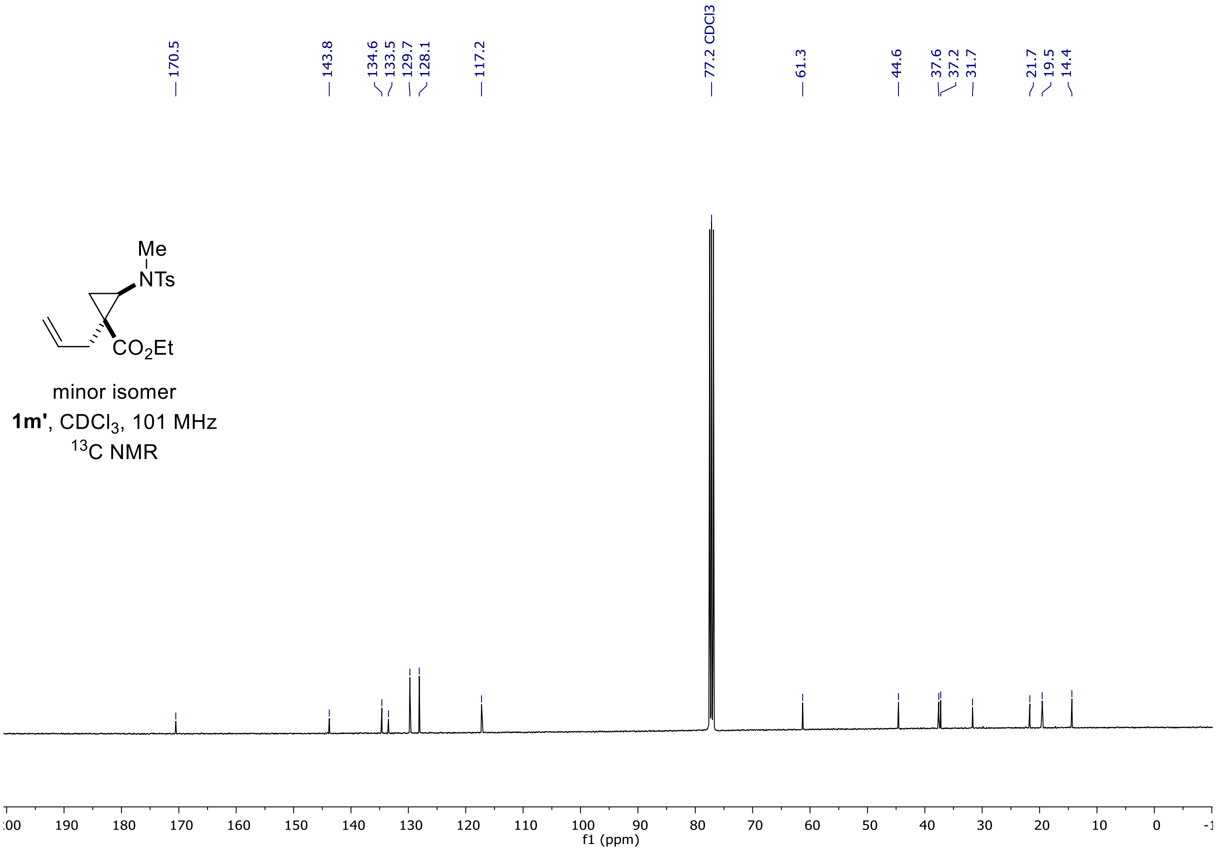

Me
NTs
CO2Et
minor isomer
1m', CDCl3, 101 MHz
13C NMR
f1 (ppm)

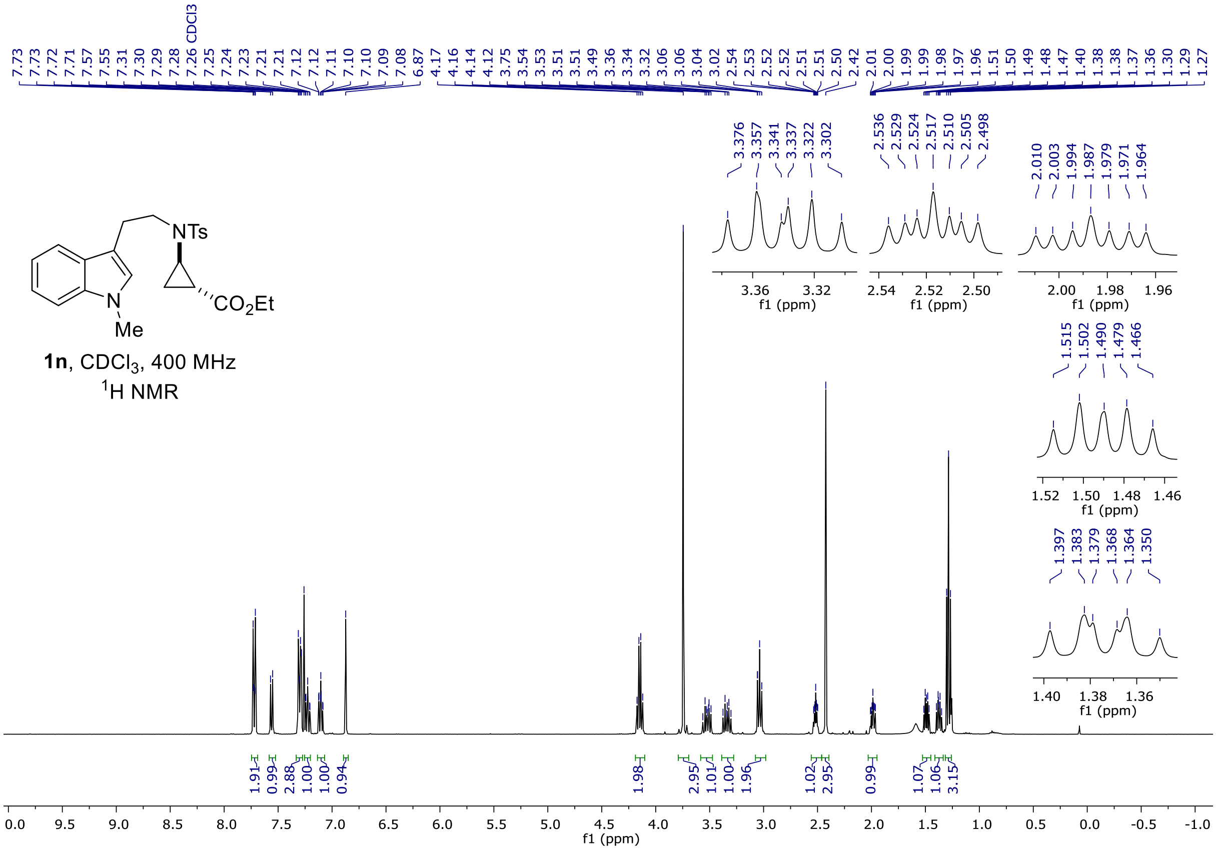

1n, $CDCl_3$, 400 MHz

^1H NMR

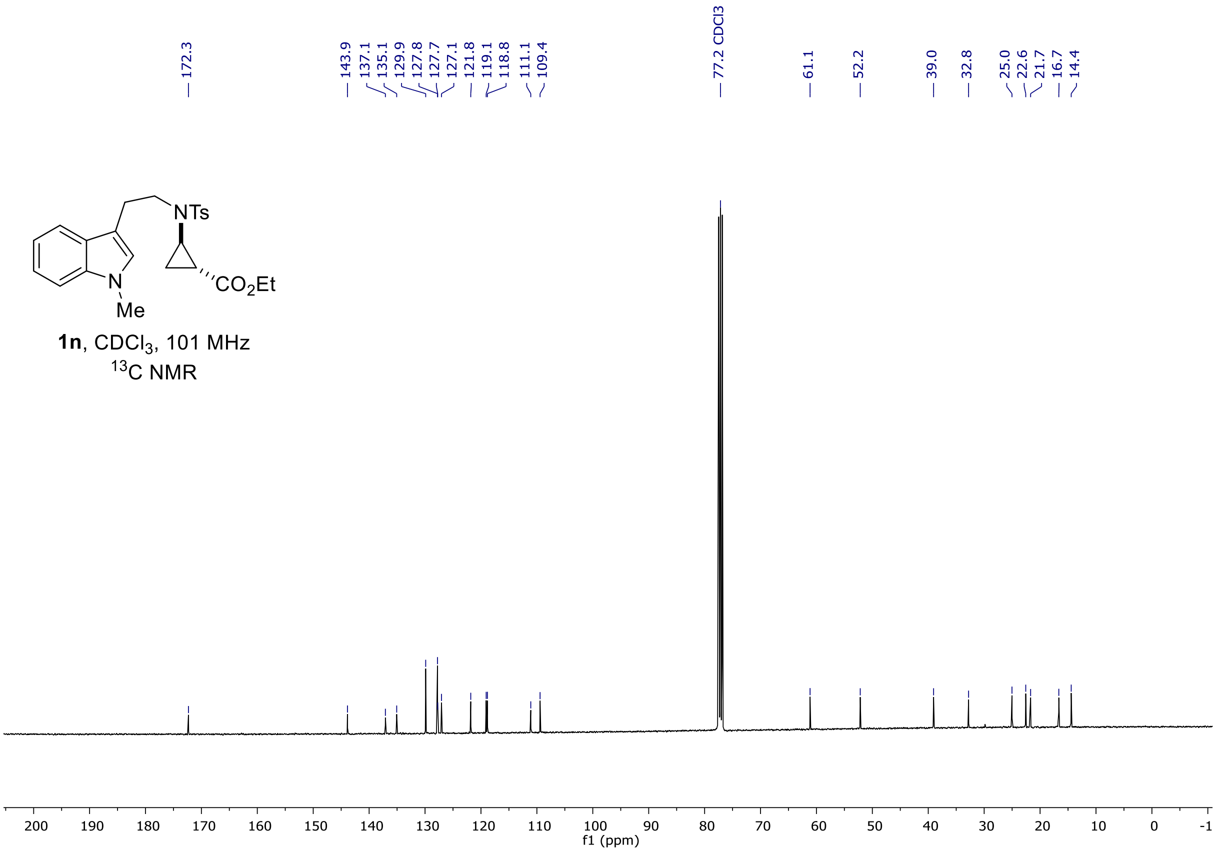

1n, $CDCl_3$, 101 MHz

^{13}C NMR

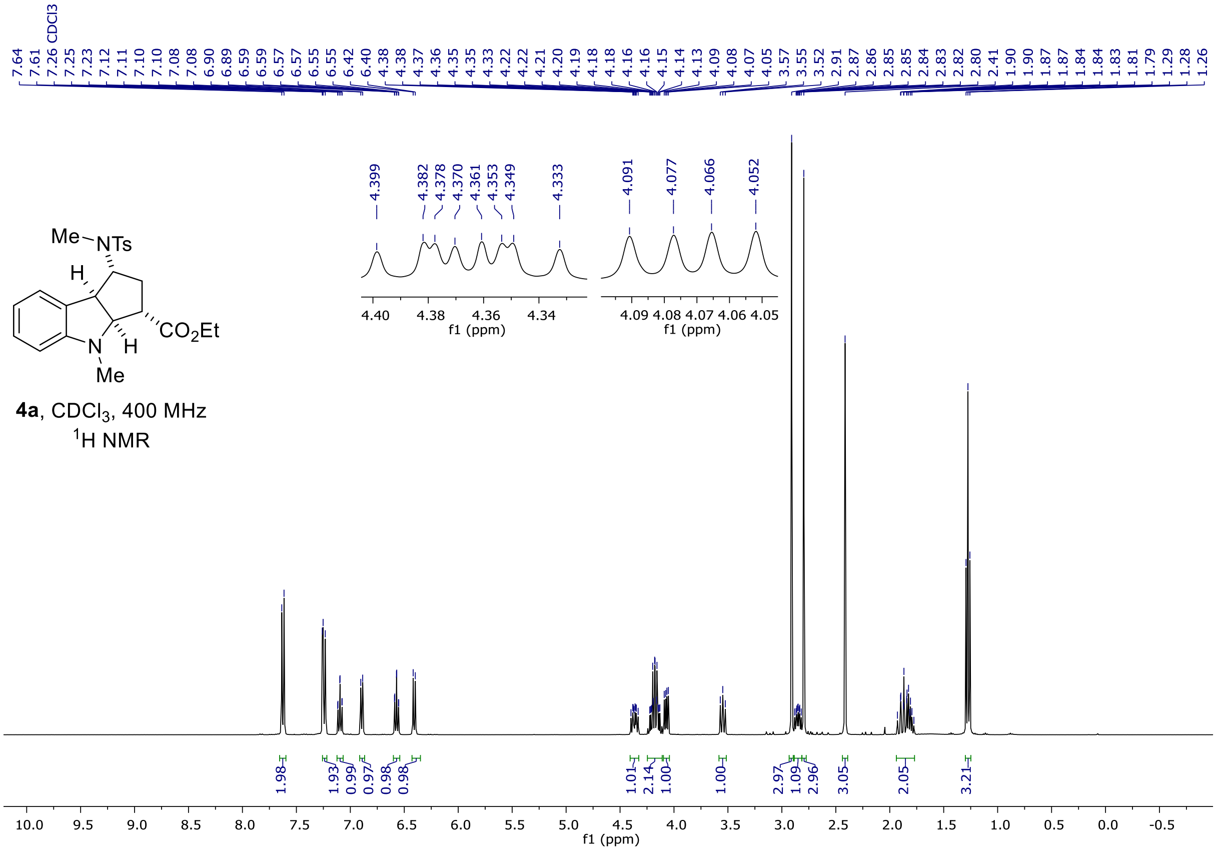

4a, $CDCl_3$, 400 MHz
^1H NMR

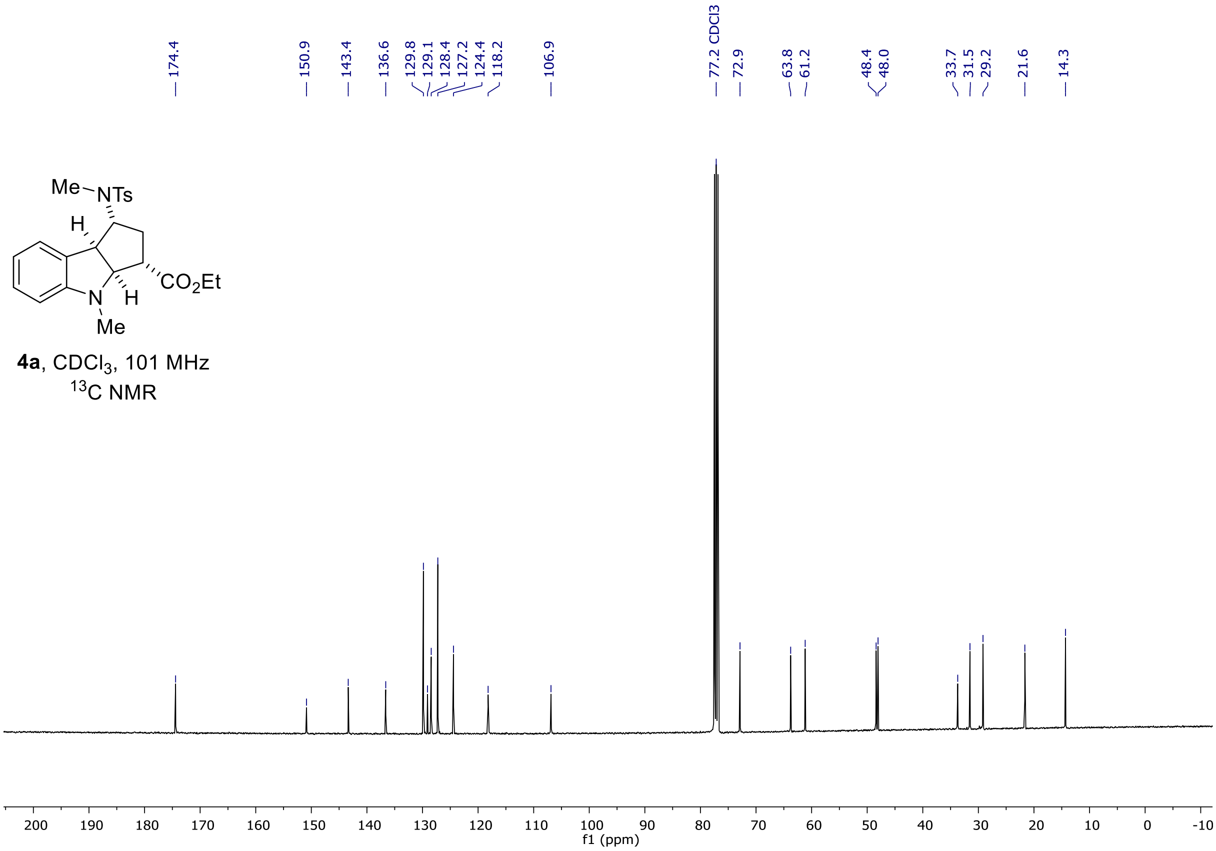

4a, $CDCl_3$, 101 MHz
^{13}C NMR

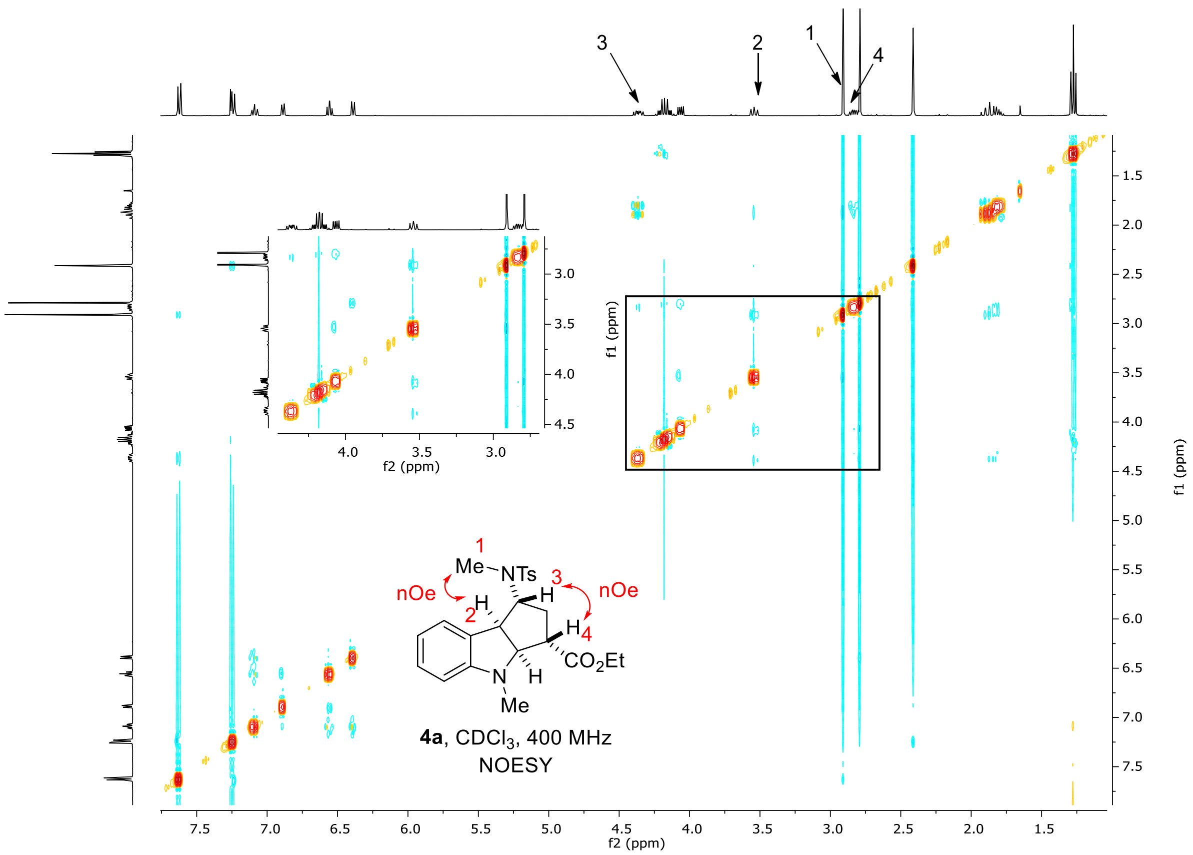

3
2
1
4
Me
NTs
H
H
nOe
nOe
CO2Et
N
H
Me
4a, CDCl3, 400 MHz
NOESY
f2 (ppm)
f1 (ppm)

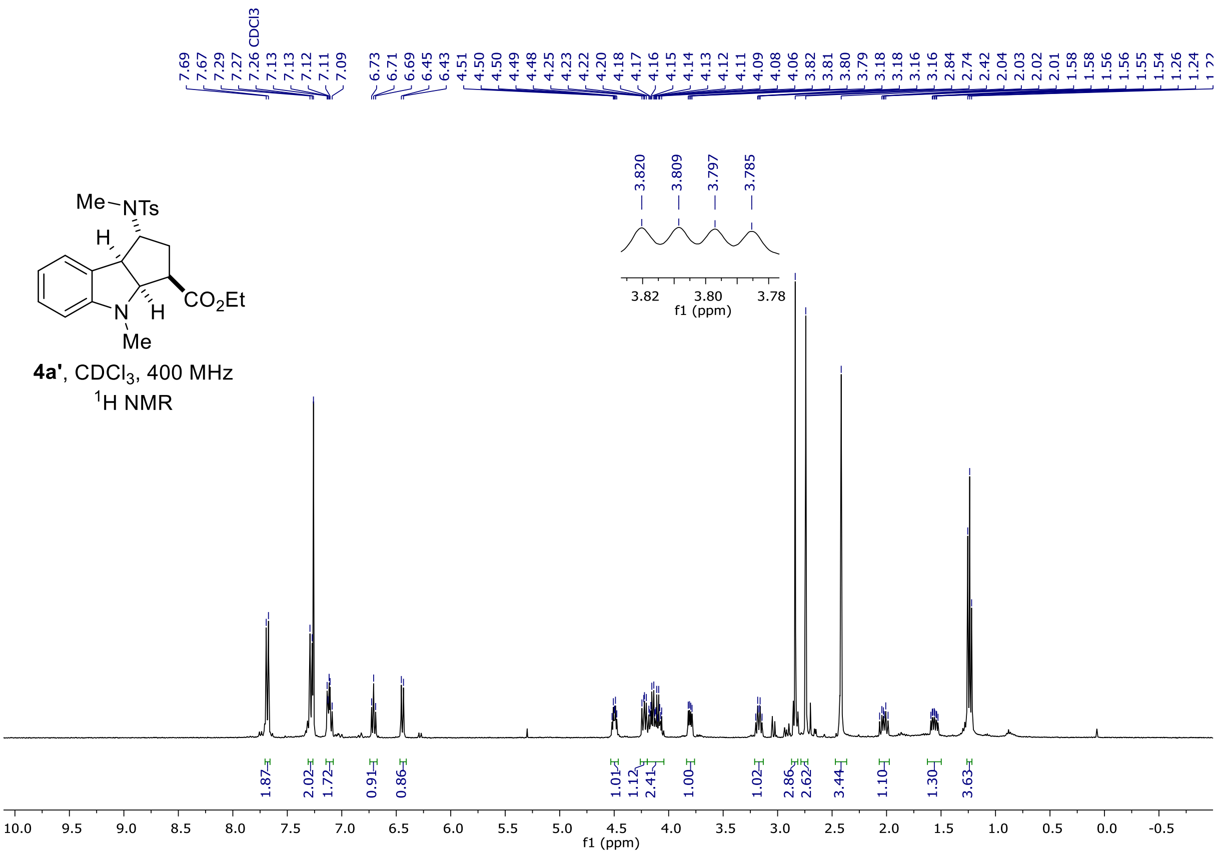

4a', $CDCl_3$, 400 MHz

^1H NMR

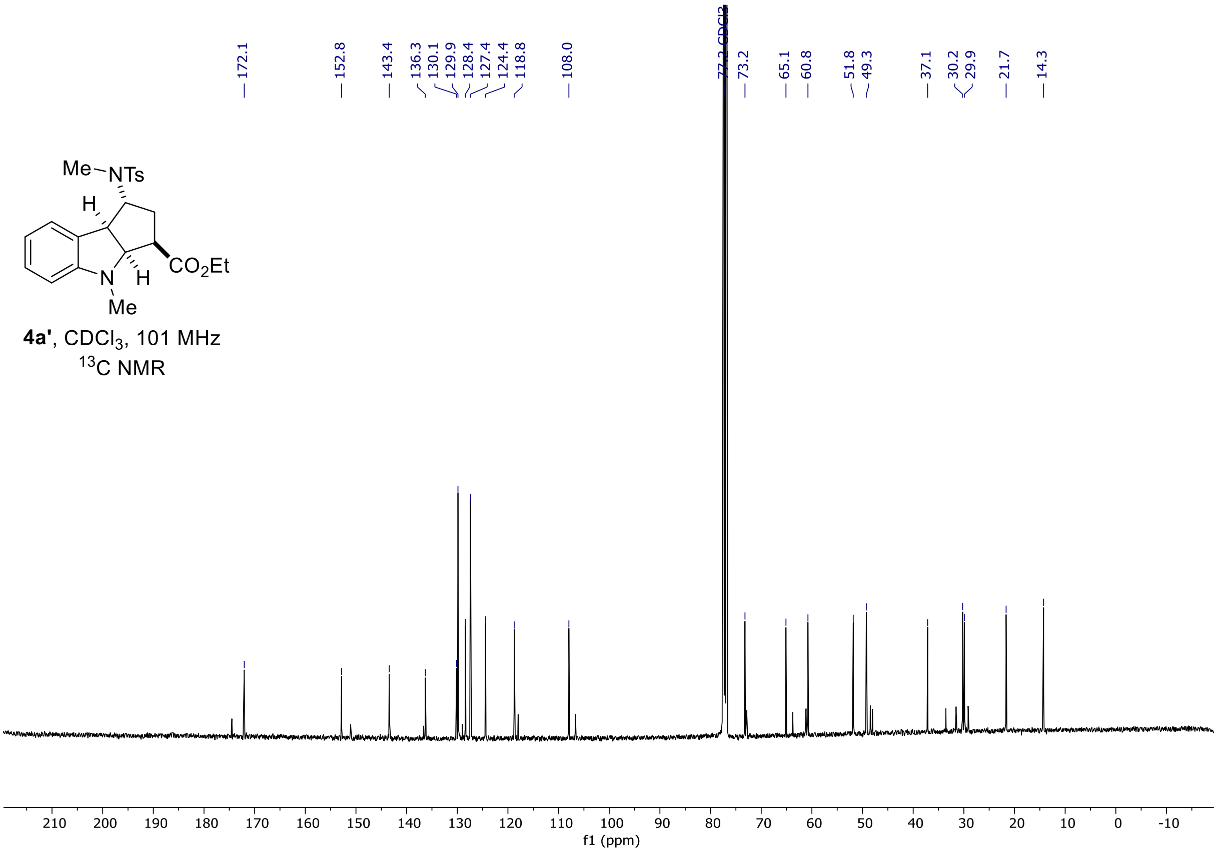

4a', $CDCl_3$, 101 MHz

^{13}C NMR

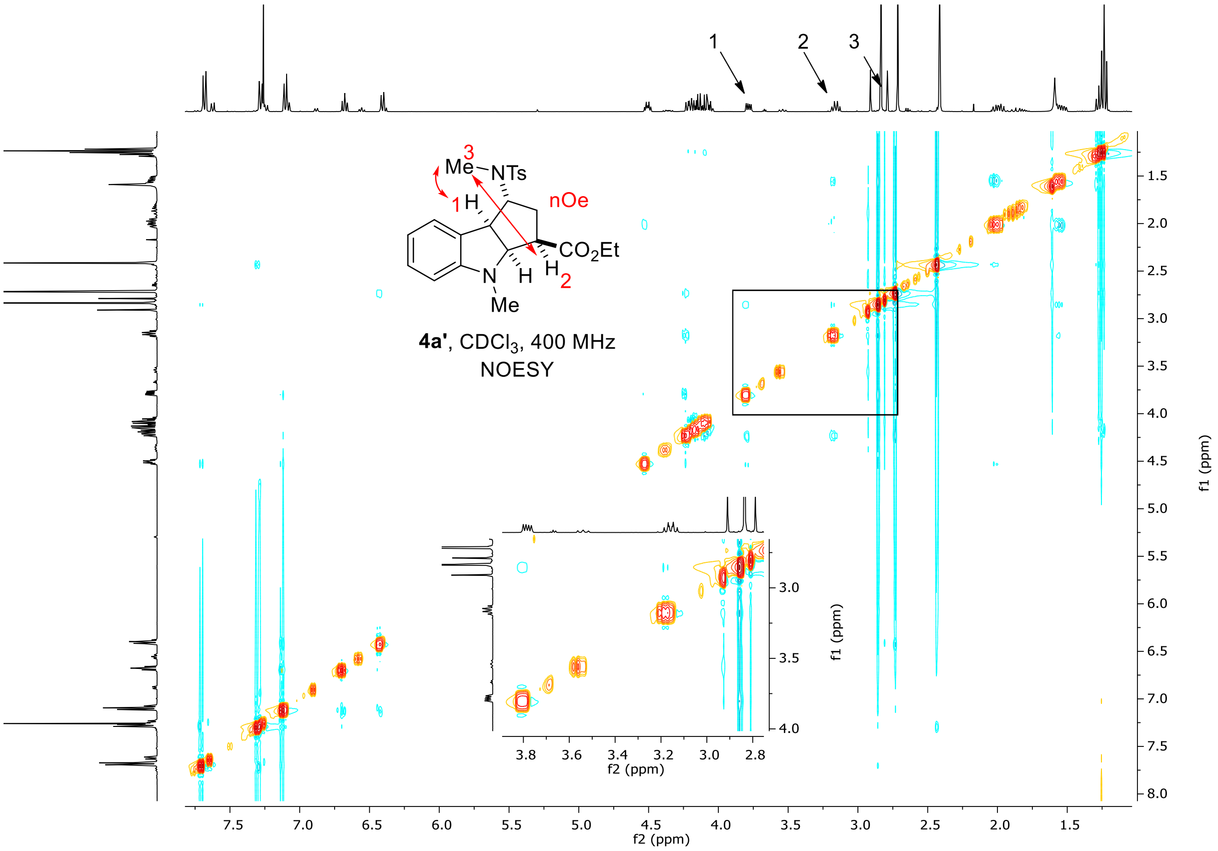

1
2
3
Me
NTs
H
nOe
CO2Et
H
N
H
Me
4a', CDCl3, 400 MHz
NOESY
f1 (ppm)
f2 (ppm)

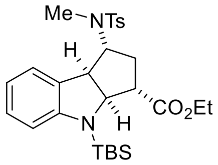

4b, $CDCl_3$, 400 MHz
^1H NMR

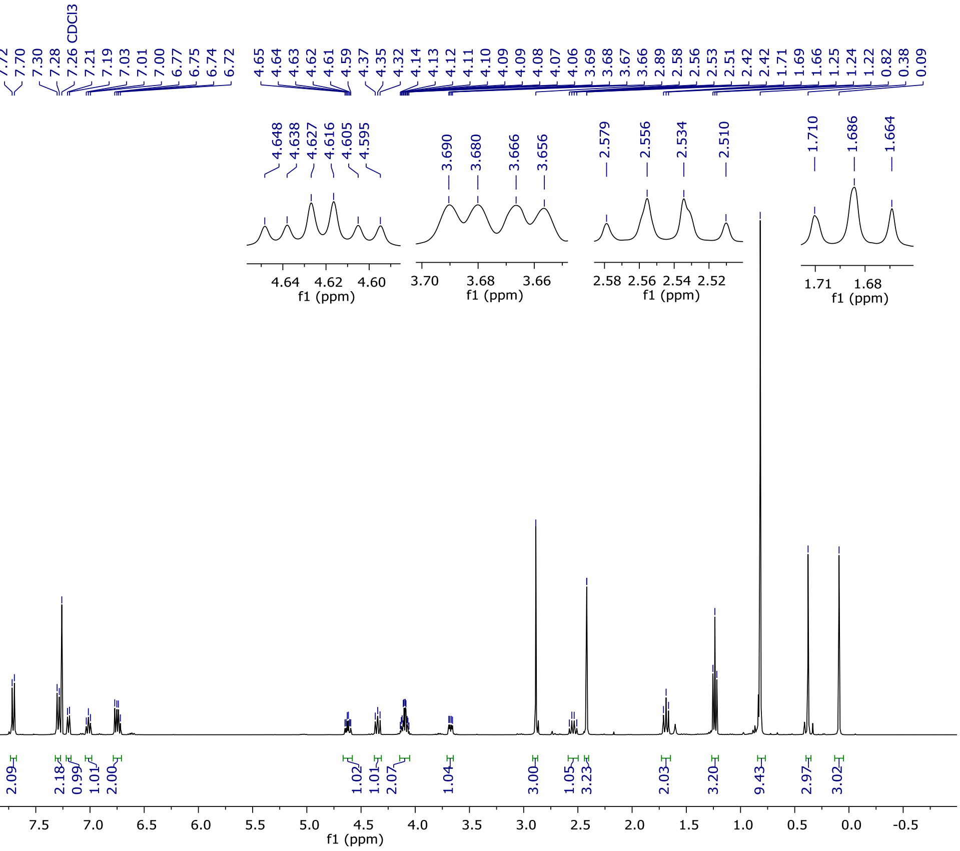

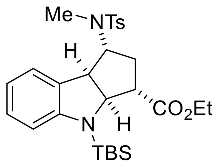

4b, $CDCl_3$, 101 MHz
^{13}C NMR

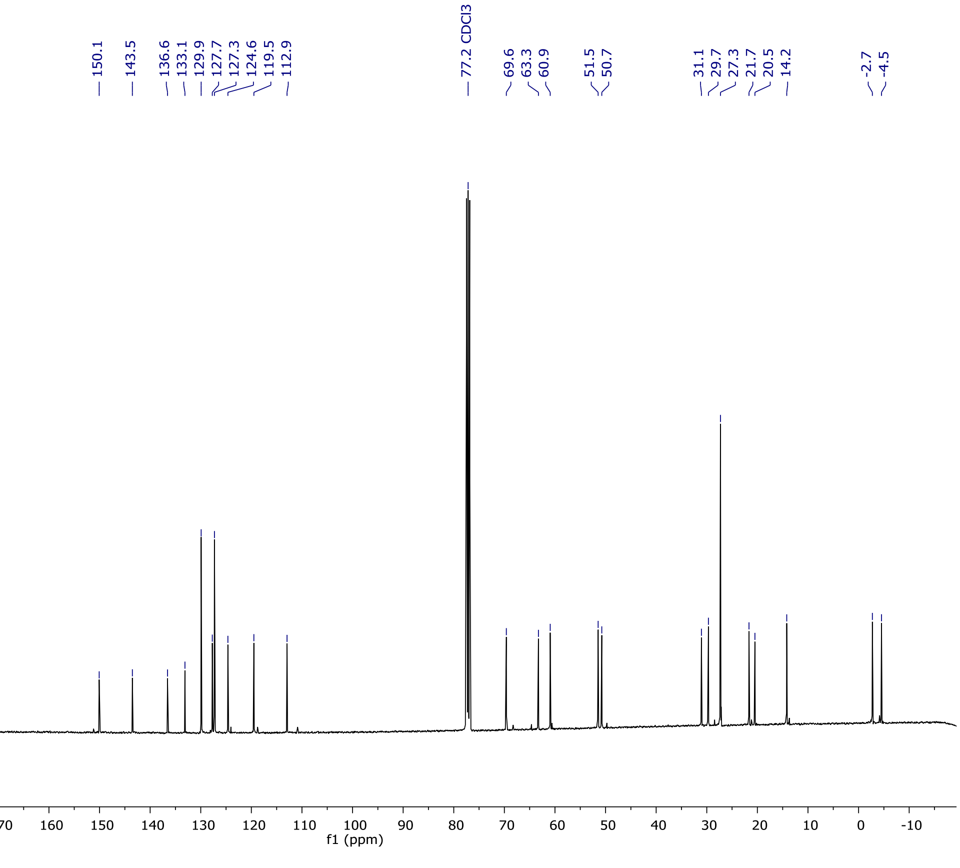

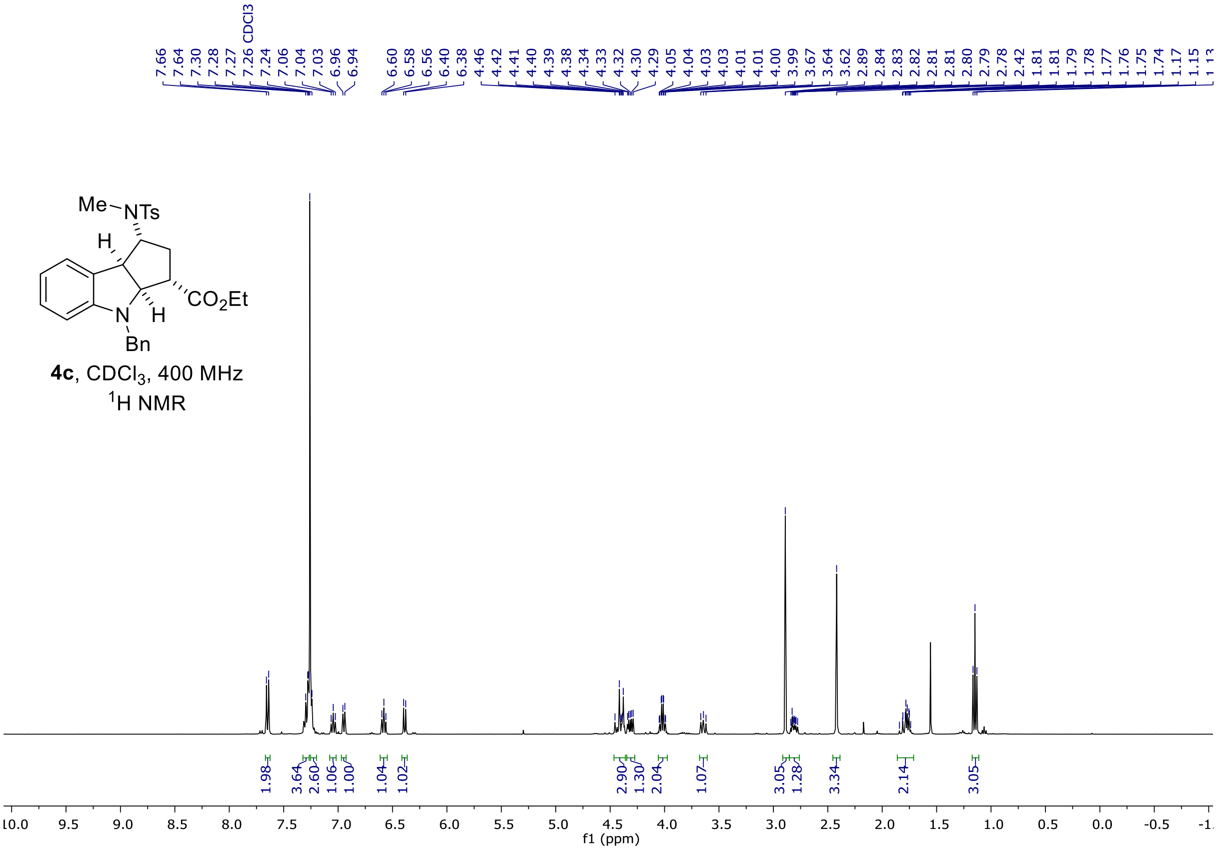
7.66 7.64 7.30 7.28 7.27 7.26 CDCl3 7.24 7.06 7.04 7.03 6.96 6.94 6.60 6.58 6.56 6.40 6.38 4.46 4.42 4.41 4.40 4.39 4.38 4.34 4.33 4.32 4.30 4.29 4.05 4.04 4.03 4.03 4.01 4.00 3.99 3.67 3.64 3.62 2.89 2.84 2.83 2.82 2.81 2.81 2.80 2.79 2.78 2.42 1.81 1.81 1.79 1.78 1.77 1.76 1.75 1.74 1.17 1.15
Me
NTs
H
CO2Et
N
H
Bn
4c, CDCl3, 400 MHz
1H NMR
1.98 3.64 2.60 1.06 1.00 1.04 1.02 2.90 1.30 2.04 1.07 3.05 1.28 3.34 2.14 3.05
f1 (ppm)

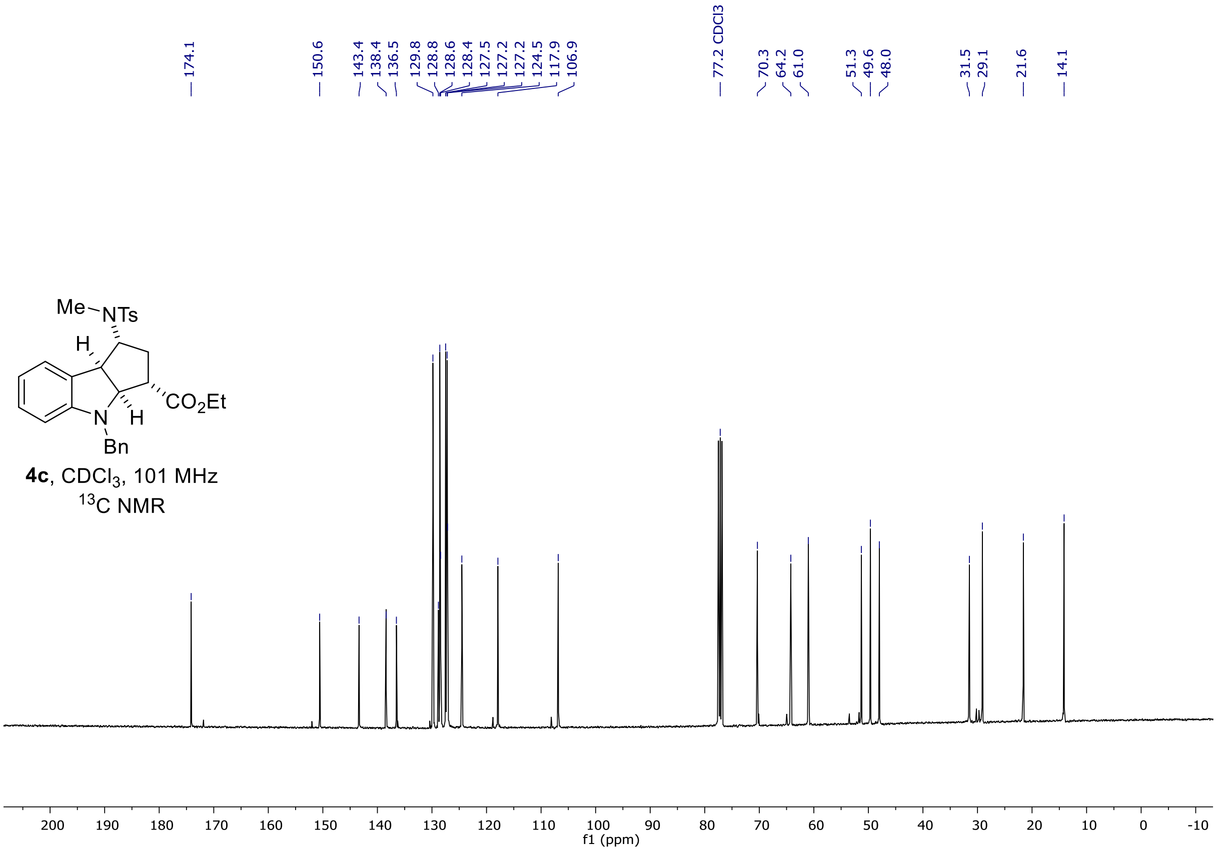
174.1 150.6 143.4 138.4 136.5 129.8 128.8 128.6 128.4 127.5 127.2 127.2 124.5 117.9 106.9 77.2 CDCl3 70.3 64.2 61.0 51.3 49.6 48.0 31.5 29.1 21.6 14.1
Me
NTs
H
CO2Et
N
H
Bn
4c, CDCl3, 101 MHz
13C NMR
f1 (ppm)

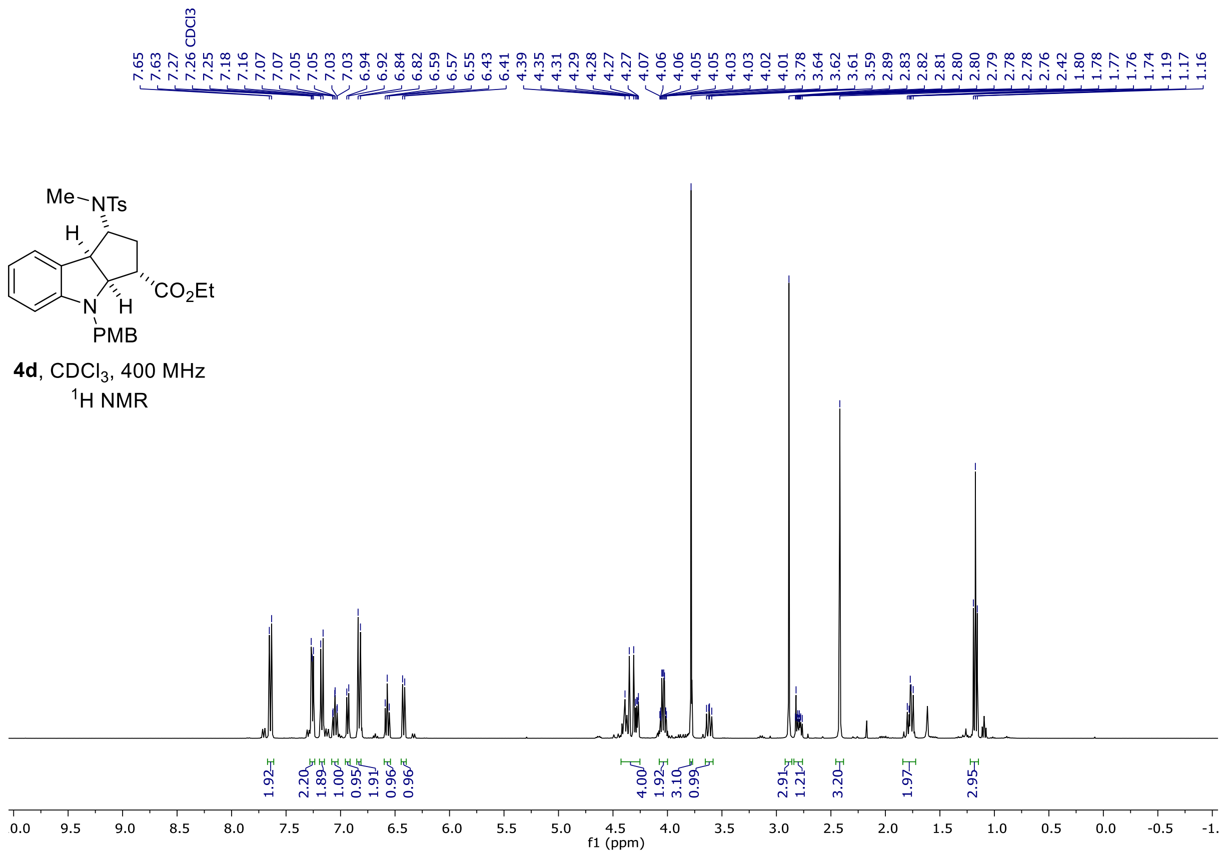

7.65 7.63 7.27 7.26 CDCl3 7.25 7.18 7.16 7.07 7.07 7.05 7.05 7.03 7.03 6.94 6.92 6.84 6.82 6.59 6.57 6.55 6.43 6.41 4.39 4.35 4.31 4.29 4.28 4.27 4.27 4.07 4.06 4.06 4.05 4.05 4.03 4.03 4.02 4.01 3.78 3.64 3.62 3.61 3.59 2.89 2.83 2.82 2.81 2.80 2.80 2.79 2.78 2.78 2.76 2.42 1.80 1.78 1.77 1.76 1.74 1.19 1.17 1.16
Me
NTs
H
CO2Et
N
H
PMB
4d, CDCl3, 400 MHz
1H NMR
1.92 2.20 1.89 1.00 0.95 1.91 0.96 0.96 4.00 1.92 3.10 0.99 2.91 1.21 3.20 1.97 2.95
f1 (ppm)

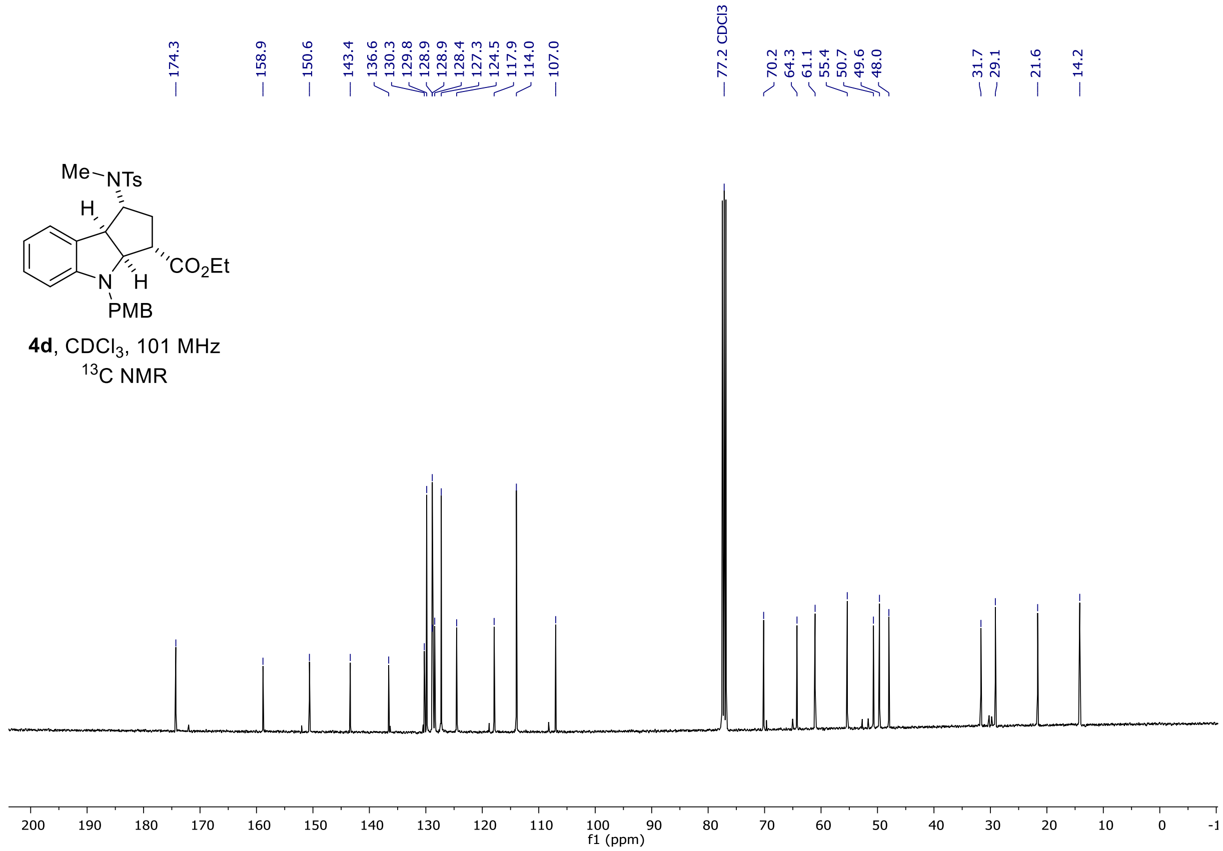

174.3 158.9 150.6 143.4 136.6 130.3 129.8 128.9 128.9 128.4 127.3 124.5 117.9 114.0 107.0 77.2 CDCl3 70.2 64.3 61.1 55.4 50.7 49.6 48.0 31.7 29.1 21.6 14.2
Me
NTs
H
CO2Et
N
H
PMB
4d, CDCl3, 101 MHz
13C NMR
f1 (ppm)

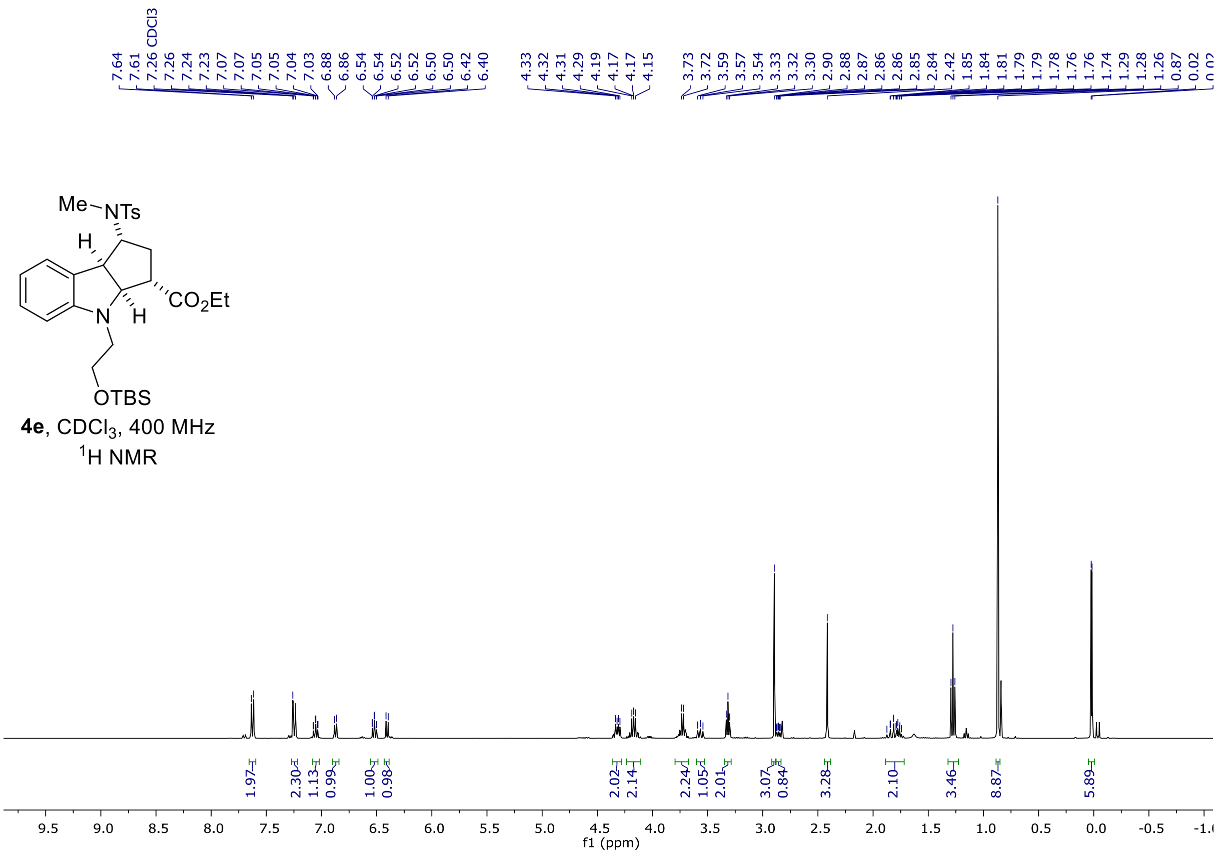
7.64 7.61 7.26 CDCl3 7.26 7.24 7.23 7.07 7.07 7.05 7.05 7.04 7.03 6.88 6.86 6.54 6.54 6.52 6.52 6.50 6.50 6.42 6.40
4.33 4.32 4.31 4.29 4.19 4.17 4.17 4.15
3.73 3.72 3.59 3.57 3.54 3.33 3.32 3.30 2.90 2.88 2.87 2.86 2.86 2.85 2.84 2.42 1.85 1.84 1.81 1.79 1.78 1.76 1.74 1.29 1.28 1.26 0.87 0.02
Me
NTs
H
CO2Et
N
H
OTBS
4e, CDCl3, 400 MHz
1H NMR
1.97 2.30 1.13 0.99 1.00 0.98 2.02 2.14 2.24 1.05 2.01 3.07 0.84 3.28 2.10 3.46 8.87 5.89
9.5 9.0 8.5 8.0 7.5 7.0 6.5 6.0 5.5 5.0 4.5 4.0 3.5 3.0 2.5 2.0 1.5 1.0 0.5 0.0 -0.5
f1 (ppm)

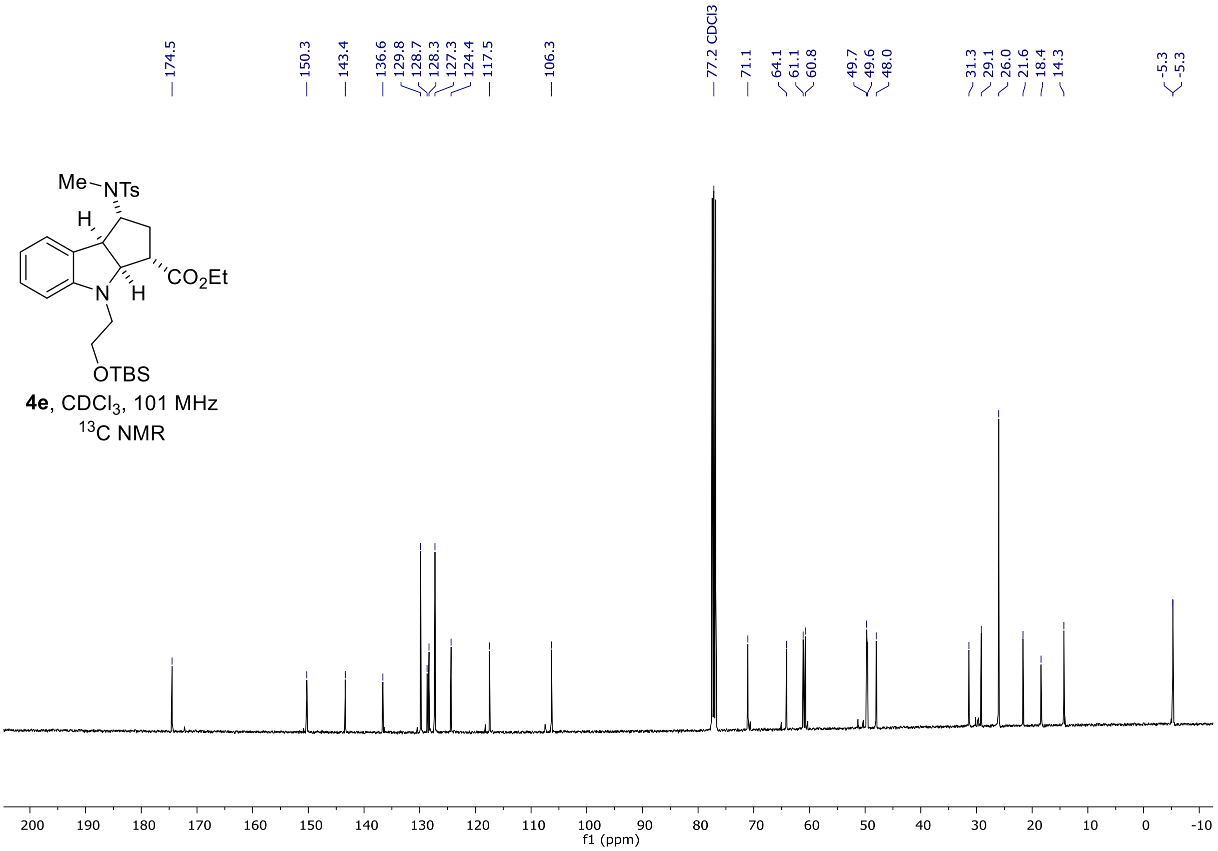
174.5 150.3 143.4 136.6 129.8 128.7 128.3 127.3 124.4 117.5 106.3
77.2 CDCl3 71.1 64.1 61.1 60.8 49.7 49.6 48.0 31.3 29.1 26.0 21.6 18.4 14.3 -5.3 -5.3
Me
NTs
H
CO2Et
N
H
OTBS
4e, CDCl3, 101 MHz
13C NMR
200 190 180 170 160 150 140 130 120 110 100 90 80 70 60 50 40 30 20 10 0 -10
f1 (ppm)

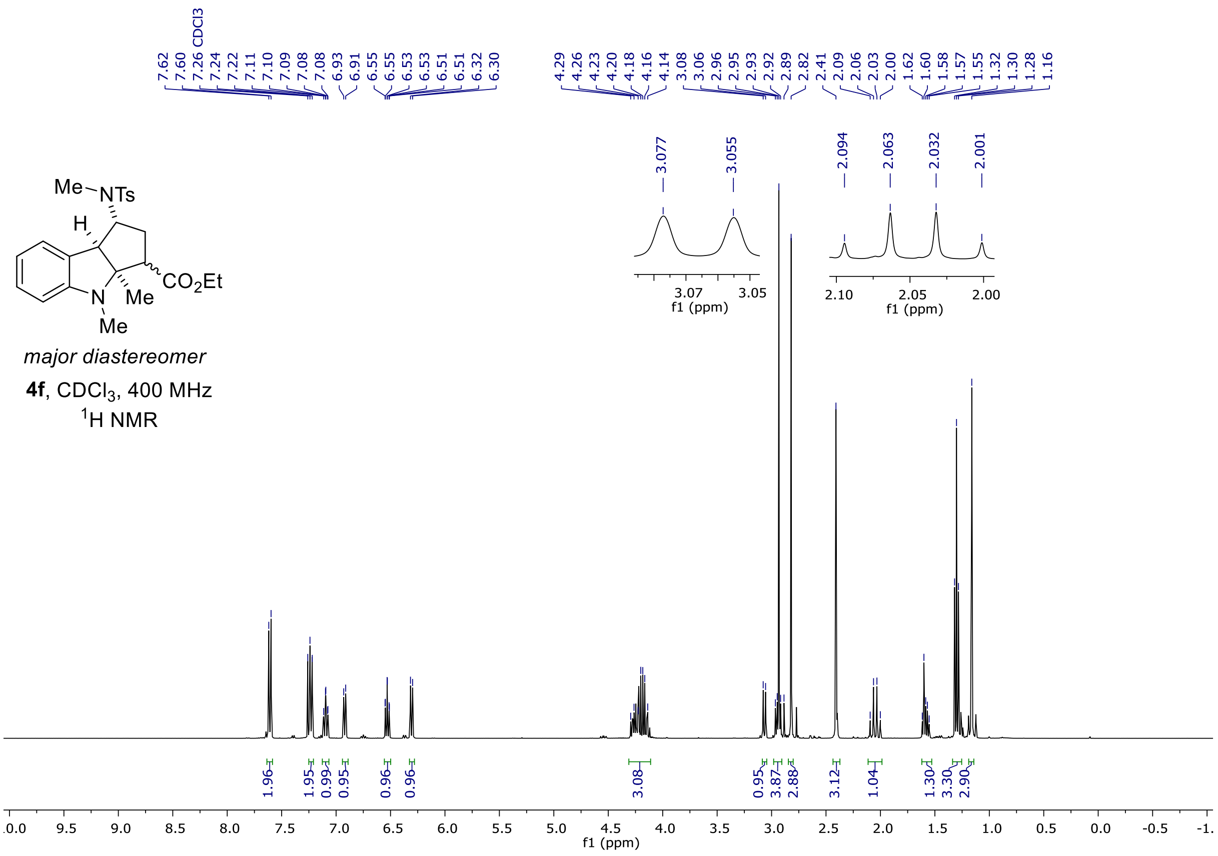

7.62
7.60
7.26 CDCl3
7.24
7.22
7.11
7.10
7.09
7.08
7.08
6.93
6.91
6.55
6.55
6.53
6.53
6.51
6.51
6.32
6.30
4.29
4.26
4.23
4.20
4.18
4.16
4.14
3.08
3.06
2.96
2.95
2.93
2.92
2.89
2.82
2.41
2.09
2.06
2.03
2.00
1.62
1.60
1.58
1.57
1.55
1.32
1.30
1.28
1.16
3.077
3.055
2.094
2.063
2.032
2.001
f1 (ppm)
Me
NTs
H
CO2Et
N
Me
Me
major diastereomer
4f, CDCl3, 400 MHz
1H NMR
1.96
1.95
0.99
0.95
0.96
0.96
3.08
0.95
3.87
2.88
3.12
1.04
1.30
3.30
2.90
f1 (ppm)

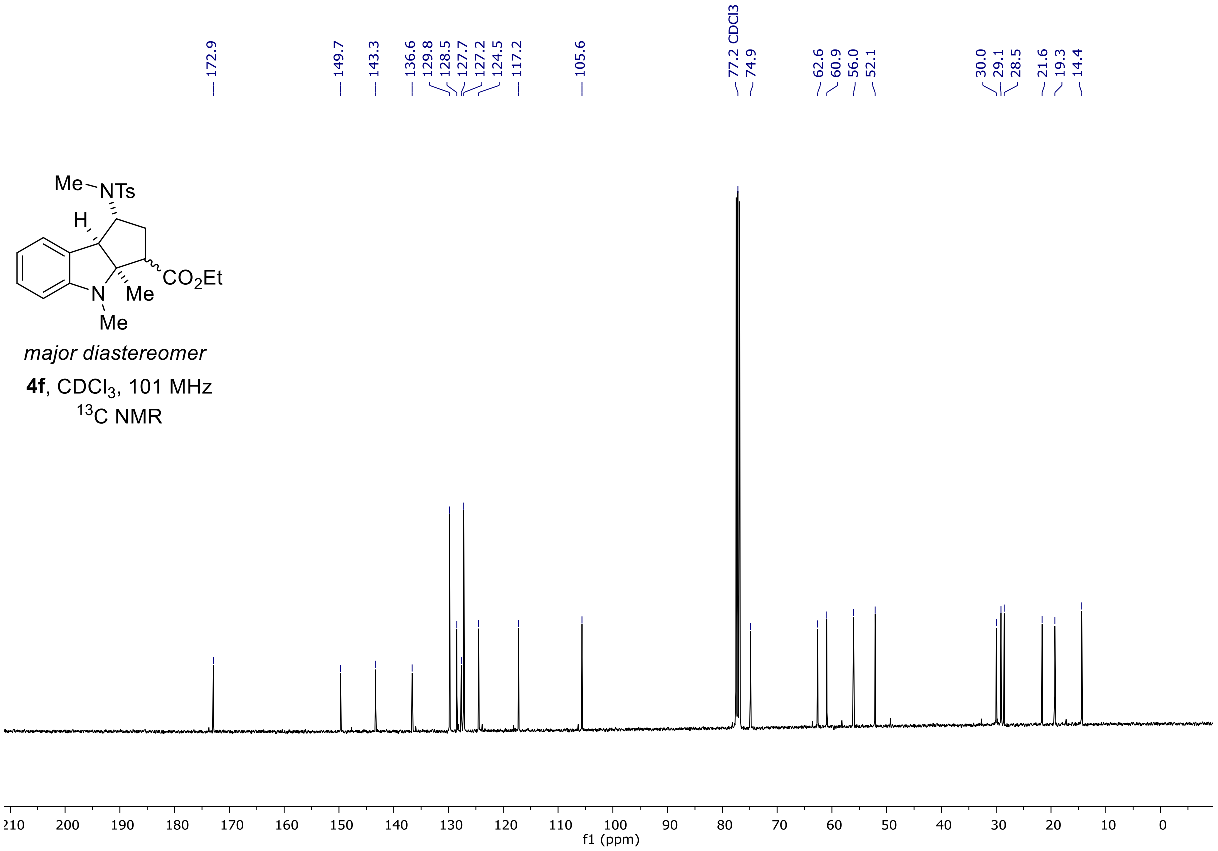

172.9
149.7
143.3
136.6
129.8
128.5
127.7
127.2
124.5
117.2
105.6
77.2 CDCl3
74.9
62.6
60.9
56.0
52.1
30.0
29.1
28.5
21.6
19.3
14.4
Me
NTs
H
CO2Et
N
Me
Me
major diastereomer
4f, CDCl3, 101 MHz
13C NMR
f1 (ppm)

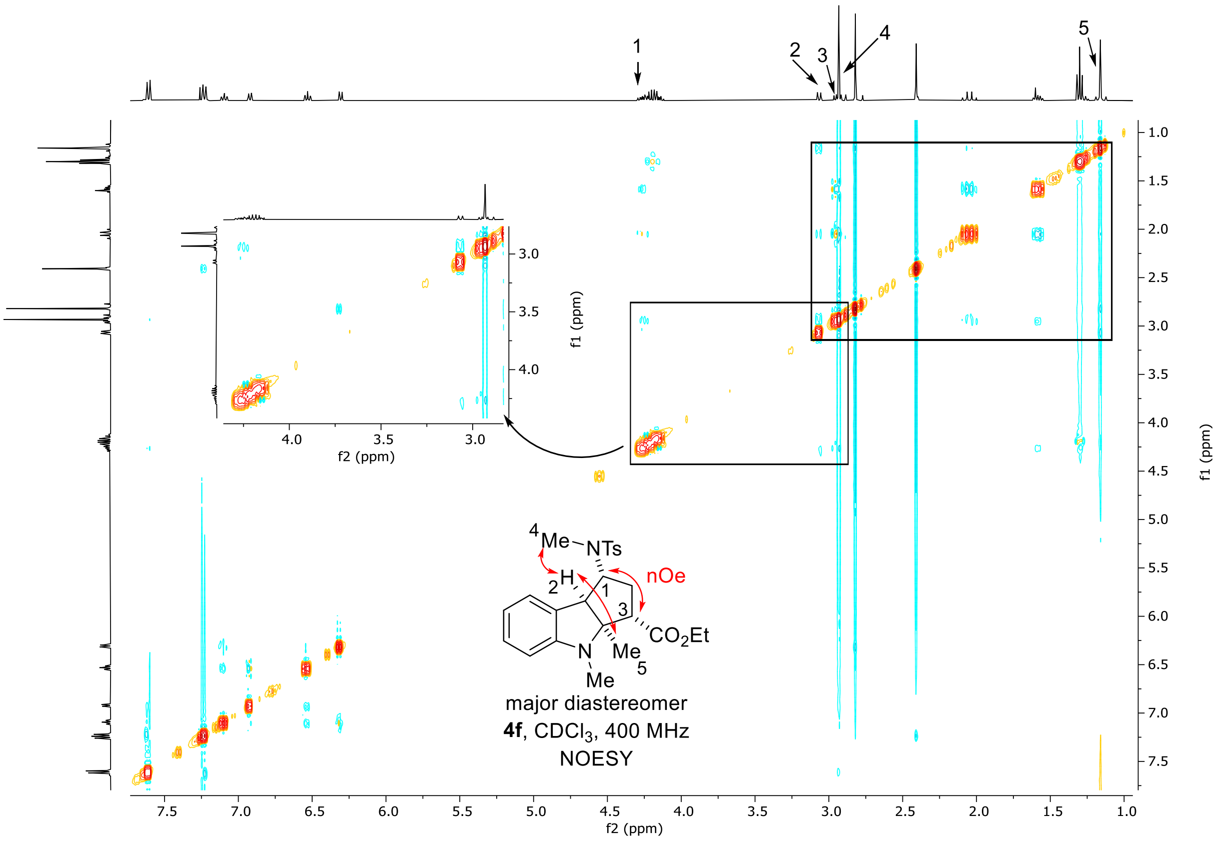
1
2
3
4
5
f1 (ppm)
f2 (ppm)
4.0
3.5
3.0
4Me
NTs
2H
1
3
nOe
CO2Et
N
Me
5
Me
major diastereomer
4f, CDCl3, 400 MHz
NOESY

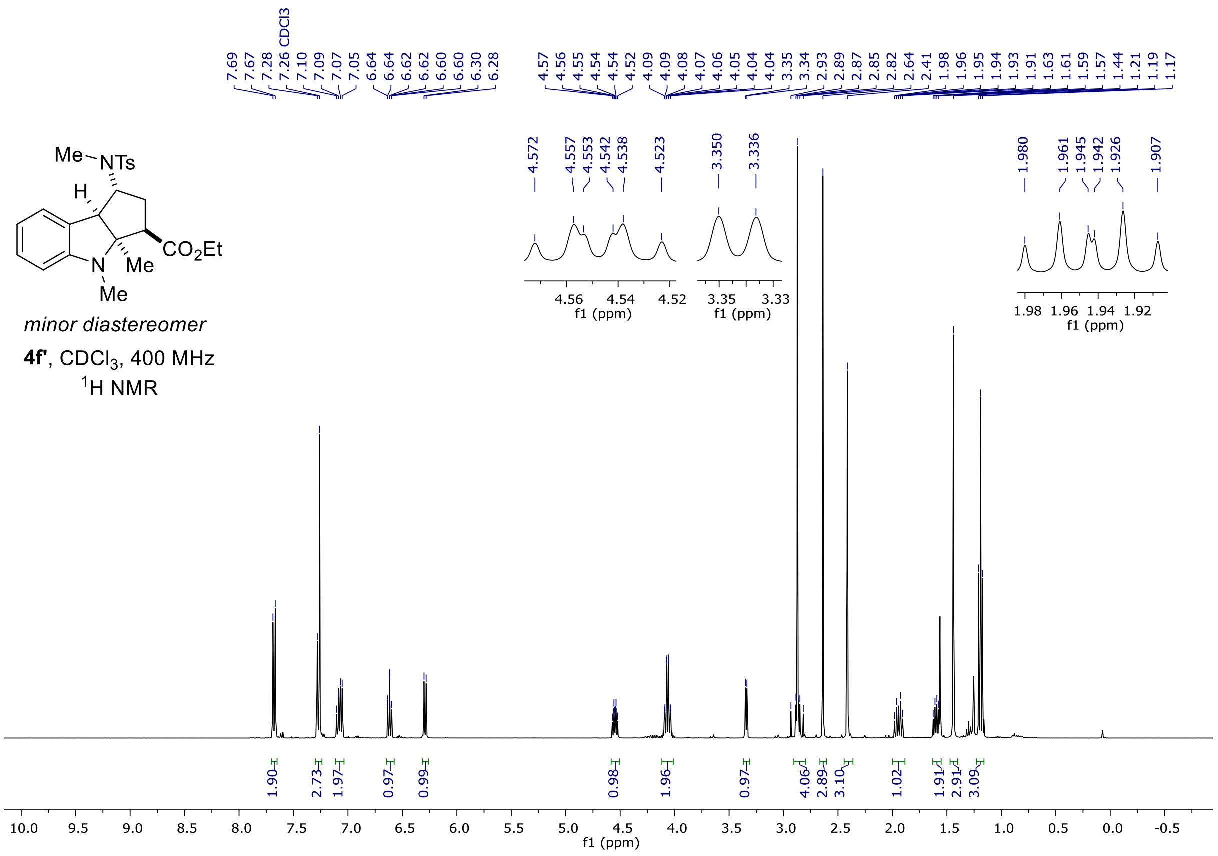
Me–NTs
H
CO2Et
N Me
Me
minor diastereomer
4f', CDCl3, 400 MHz
1H NMR
7.69 7.67 7.28 7.26 CDCl3 7.10 7.09 7.07 7.05 6.64 6.64 6.62 6.62 6.60 6.60 6.30 6.28
4.57 4.56 4.55 4.54 4.54 4.52 4.09 4.09 4.08 4.07 4.06 4.05 4.04 4.04 3.35 3.34 2.93 2.89 2.87 2.85 2.82 2.64 2.41 1.98 1.96 1.95 1.94 1.93 1.91 1.63 1.61 1.59 1.57 1.44 1.21 1.19 1.17
4.572 4.557 4.553 4.542 4.538 4.523
3.350 3.336
1.980 1.961 1.945 1.942 1.926 1.907
1.90 2.73 1.97 0.97 0.99 0.98 1.96 0.97 4.06 2.89 3.10 1.02 1.91 2.91 3.09
f1 (ppm)

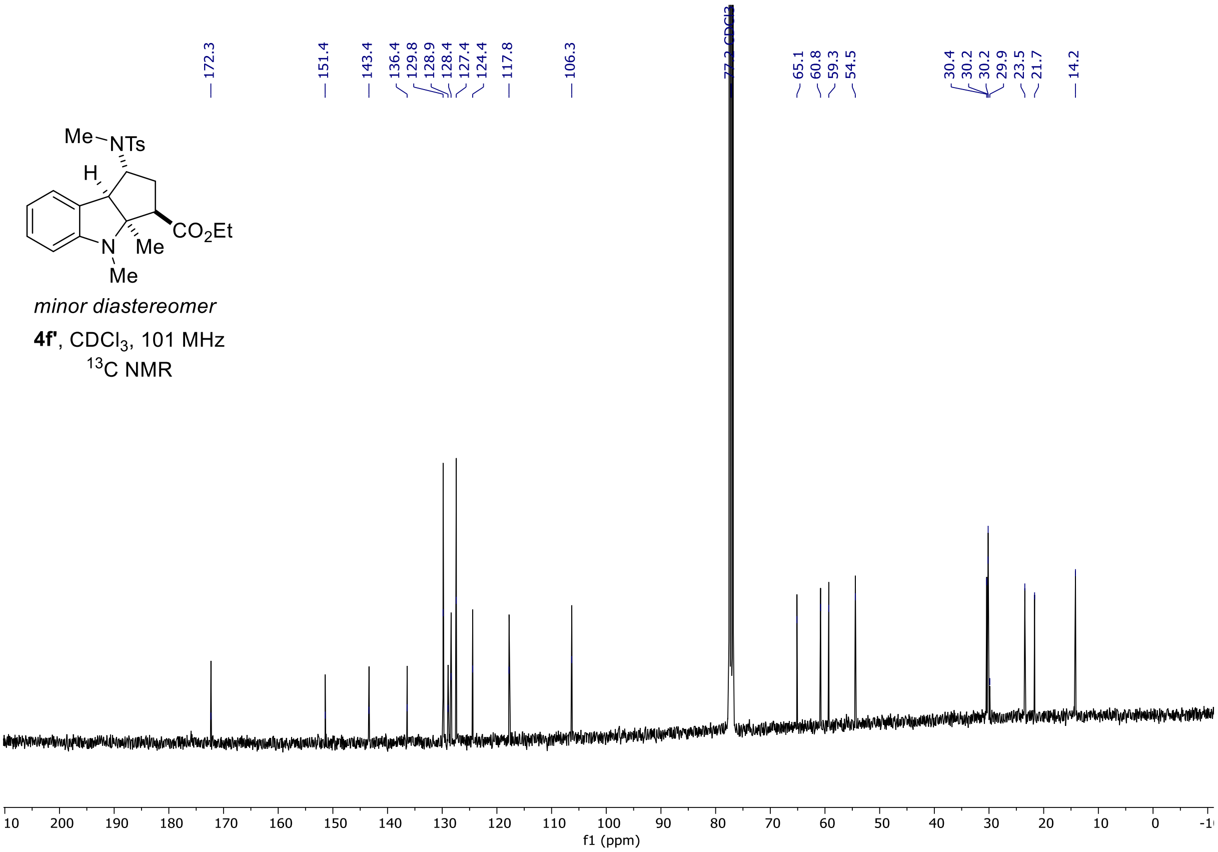
Me–NTs
H
CO2Et
N Me
Me
minor diastereomer
4f', CDCl3, 101 MHz
13C NMR
172.3 151.4 143.4 136.4 129.8 128.9 128.4 127.4 124.4 117.8 106.3 77.2 CDCl3 65.1 60.8 59.3 54.5 30.4 30.2 30.2 29.9 23.5 21.7 14.2
f1 (ppm)

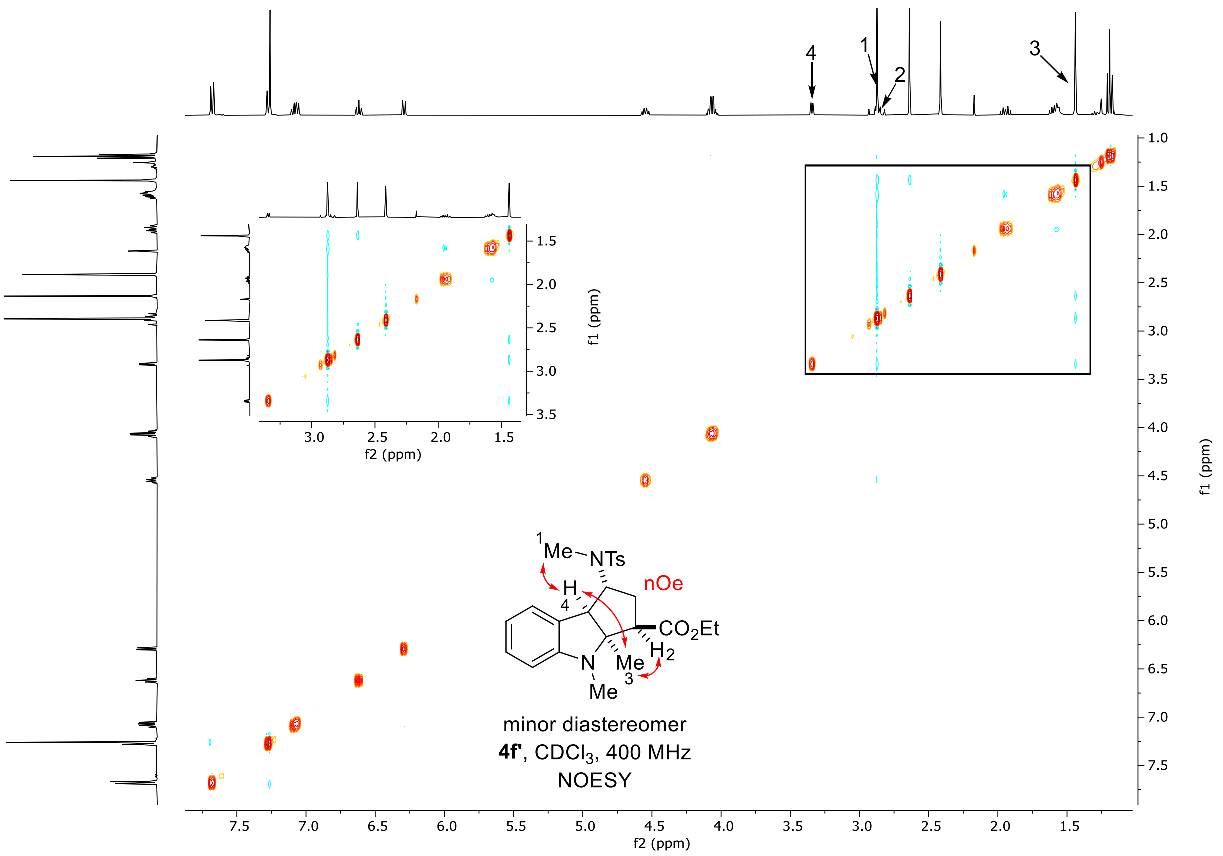
4
1
2
3
f1 (ppm)
f2 (ppm)
1Me
NTs
nOe
H
4
CO2Et
N
Me
3
H2
Me
minor diastereomer
4f', CDCl3, 400 MHz
NOESY

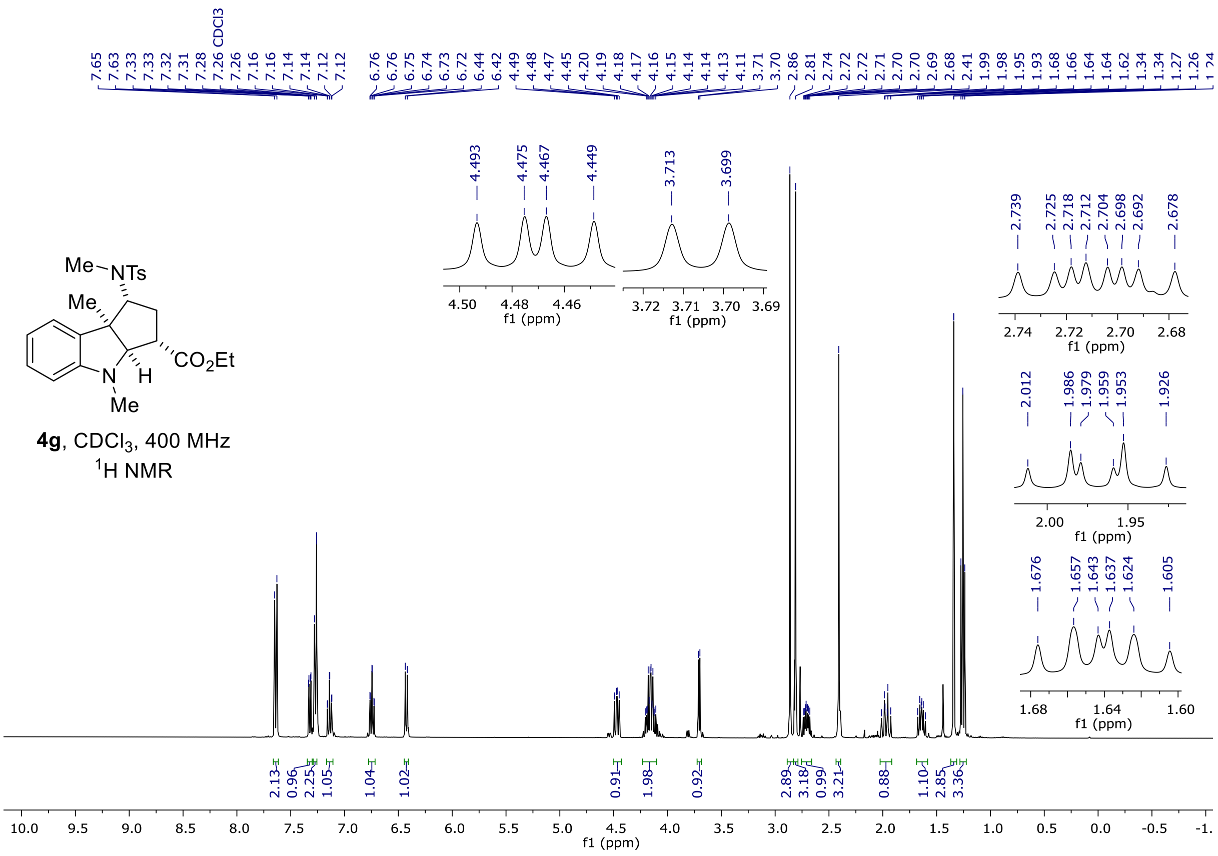
Me–NTs
Me
CO2Et
N H
Me
4g, CDCl3, 400 MHz
1H NMR
f1 (ppm)

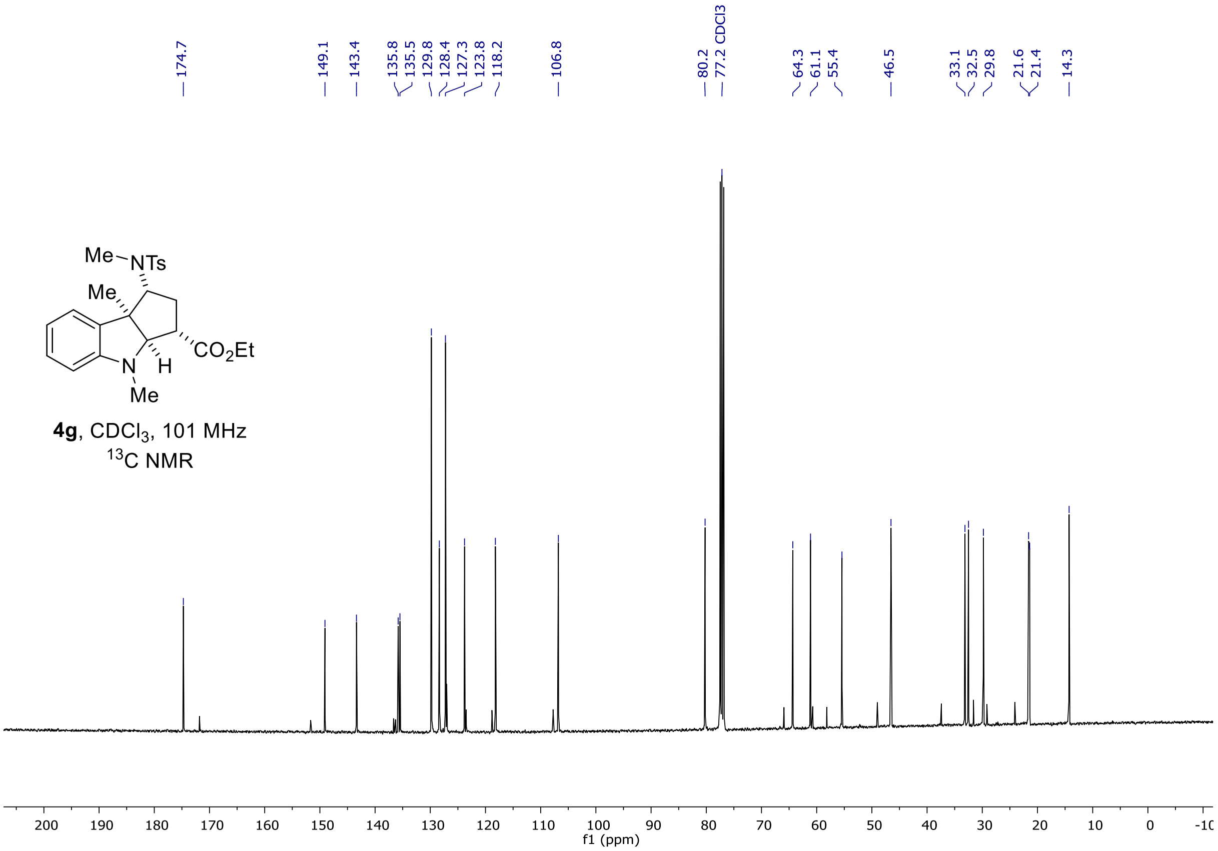
Me–NTs
Me
CO2Et
N H
Me
4g, CDCl3, 101 MHz
13C NMR
174.7
149.1
143.4
135.8
135.5
129.8
128.4
127.3
123.8
118.2
106.8
80.2
77.2 CDCl3
64.3
61.1
55.4
46.5
33.1
32.5
29.8
21.6
21.4
14.3
f1 (ppm)

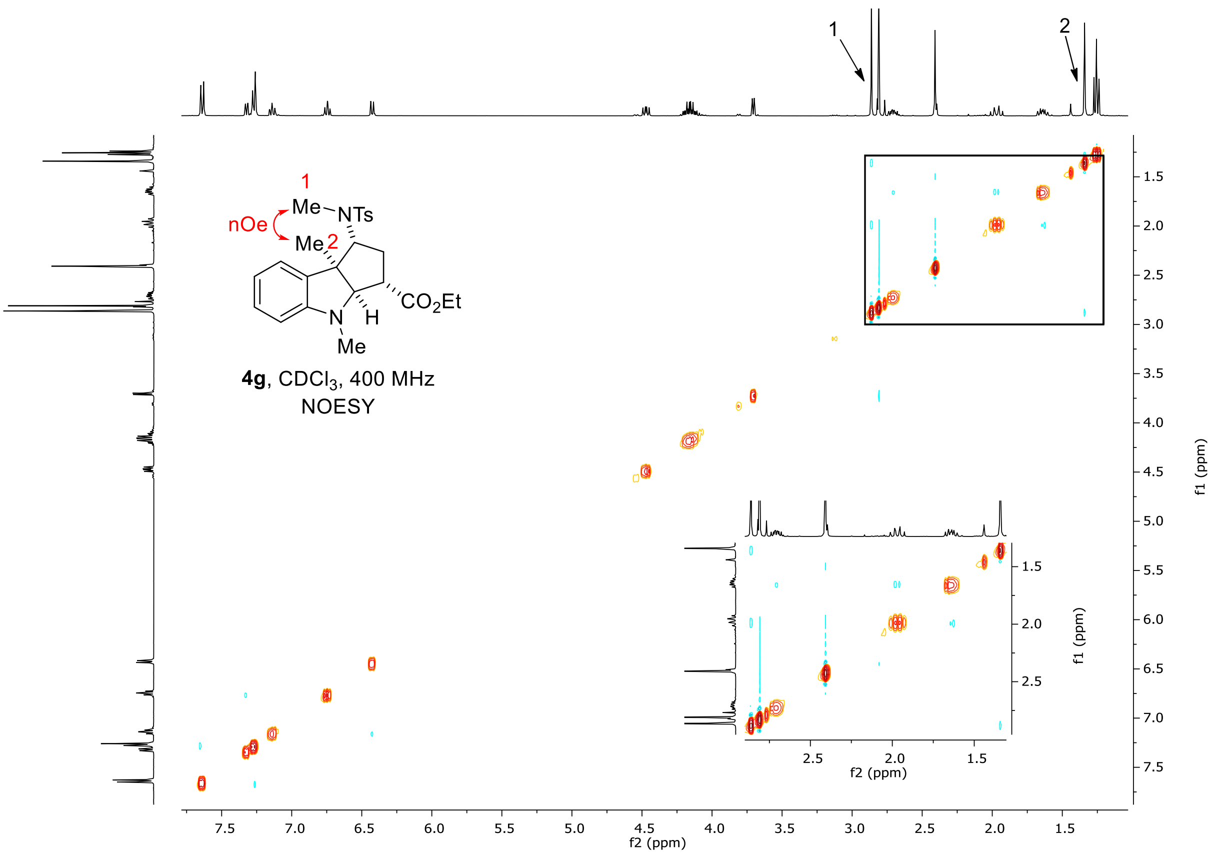

1
2
nOe
Me
NTs
Me
CO2Et
N
H
Me
4g, CDCl3, 400 MHz
NOESY
f1 (ppm)
f2 (ppm)

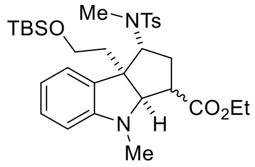

in mixture of diast.
(dr 71:29)

4h, $CDCl_3$, 400 MHz
^{1}H NMR

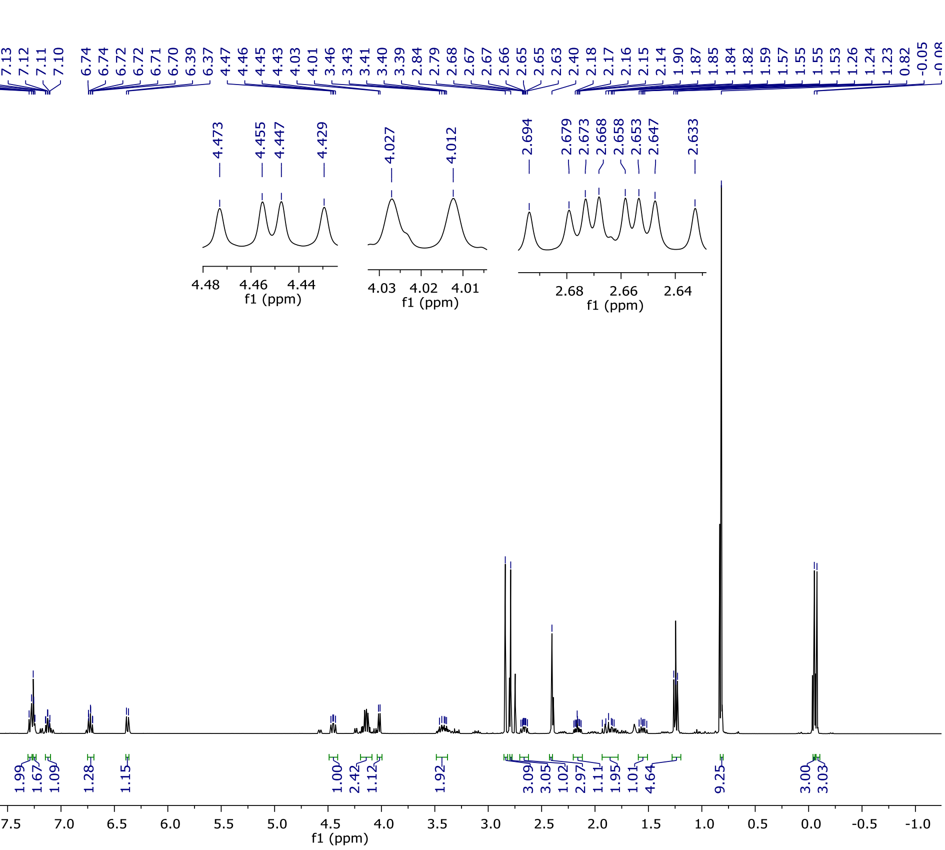

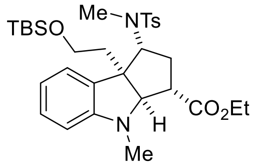

in mixture of diast.
(dr 71:29)

4h, $CDCl_3$, 101 MHz
^{13}C NMR

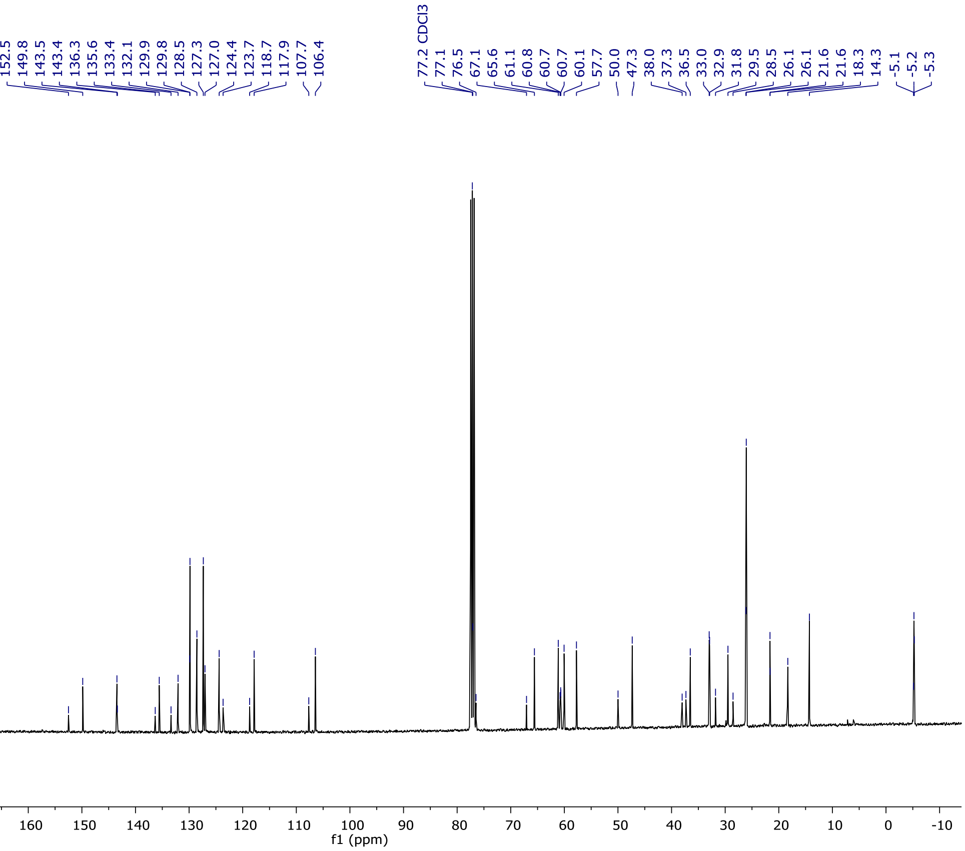

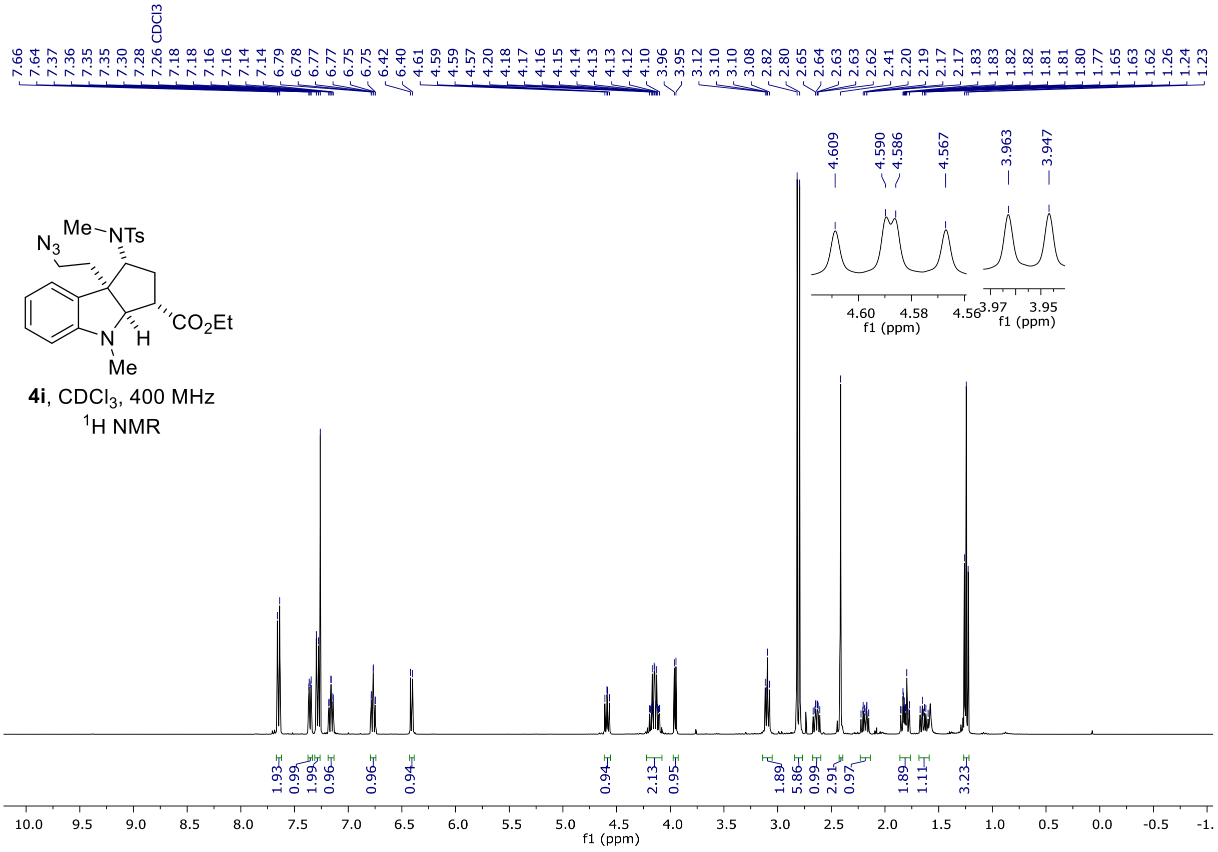
7.66 7.64 7.37 7.36 7.35 7.35 7.30 7.28 7.26 CDCl3 7.18 7.18 7.16 7.16 7.14 7.14 6.79 6.78 6.77 6.77 6.75 6.75 6.42 6.40 4.61 4.59 4.59 4.57 4.20 4.18 4.17 4.16 4.15 4.14 4.13 4.13 4.12 4.10 3.96 3.95 3.12 3.10 3.10 3.08 2.82 2.80 2.65 2.64 2.63 2.63 2.62 2.41 2.20 2.19 2.17 2.17 1.83 1.83 1.82 1.82 1.81 1.81 1.80 1.77 1.65 1.63 1.62 1.26 1.24 1.23
4.609 4.590 4.586 4.567 3.963 3.947
4.60 4.58 4.56 f1 (ppm)
3.97 3.95 f1 (ppm)
Me NTs
N3
CO2Et
N H
Me
4i, CDCl3, 400 MHz
1H NMR
1.93 0.99 1.99 0.96 0.96 0.94 0.94 2.13 0.95 1.89 5.86 0.99 2.91 0.97 1.89 1.11 3.23
10.0 9.5 9.0 8.5 8.0 7.5 7.0 6.5 6.0 5.5 5.0 4.5 4.0 3.5 3.0 2.5 2.0 1.5 1.0 0.5 0.0 -0.5 -1.
f1 (ppm)

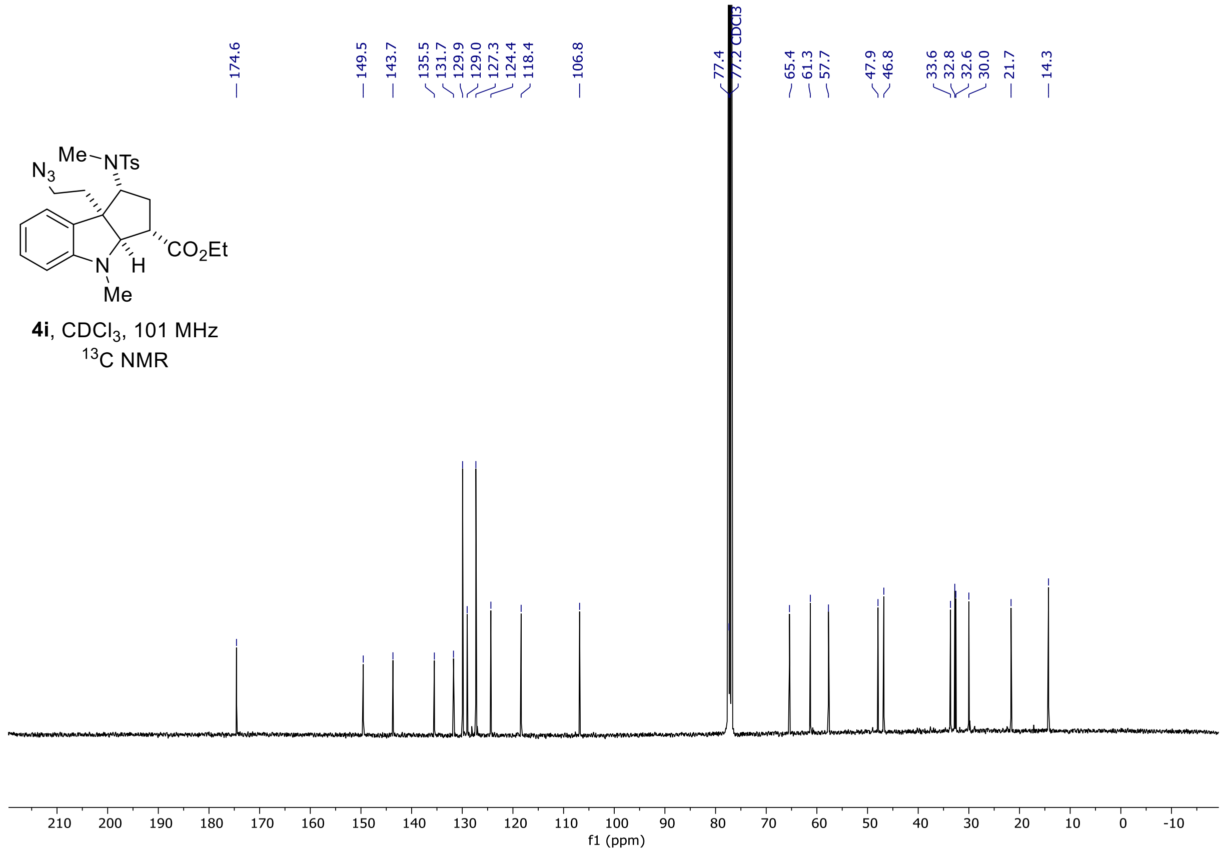
174.6 149.5 143.7 135.5 131.7 129.9 129.0 127.3 124.4 118.4 106.8 77.4 77.2 CDCl3 65.4 61.3 57.7 47.9 46.8 33.6 32.8 32.6 30.0 21.7 14.3
Me NTs
N3
CO2Et
N H
Me
4i, CDCl3, 101 MHz
13C NMR
210 200 190 180 170 160 150 140 130 120 110 100 90 80 70 60 50 40 30 20 10 0 -10
f1 (ppm)

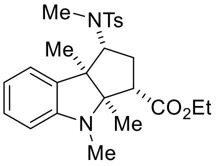

major diastereomer

4j, $CDCl_3$, 400 MHz

^{1}H NMR

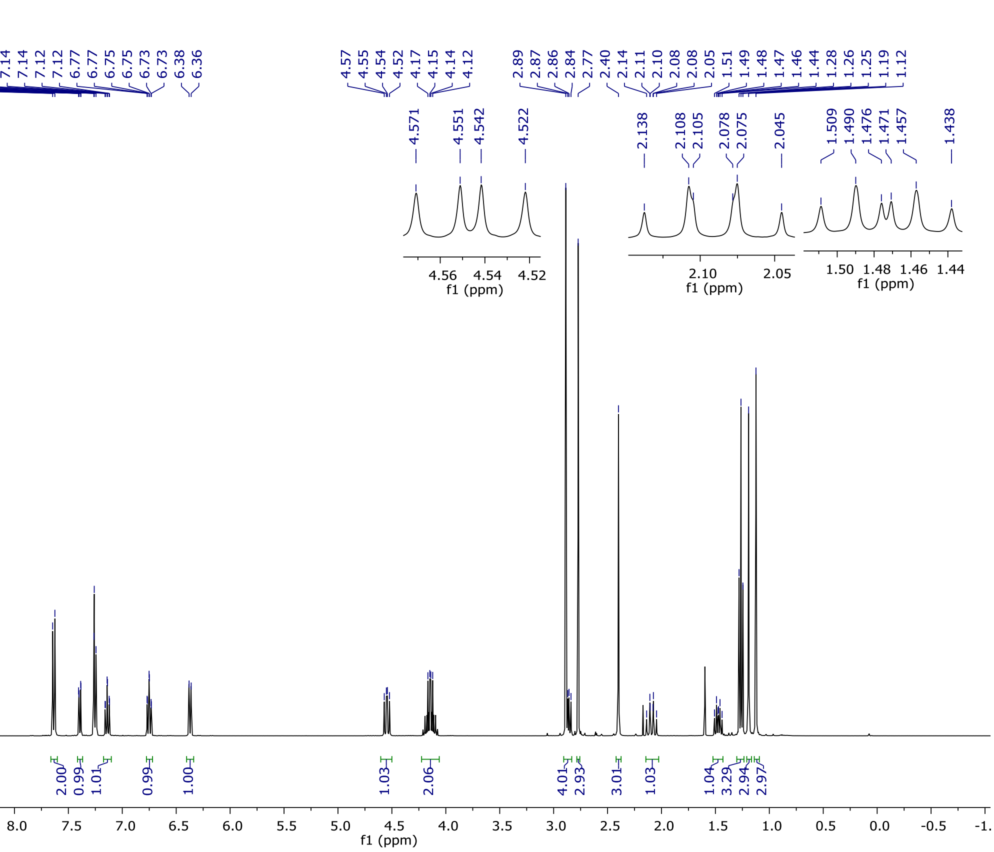

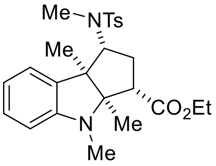

major diastereomer

4j, $CDCl_3$, 101 MHz

^{13}C NMR

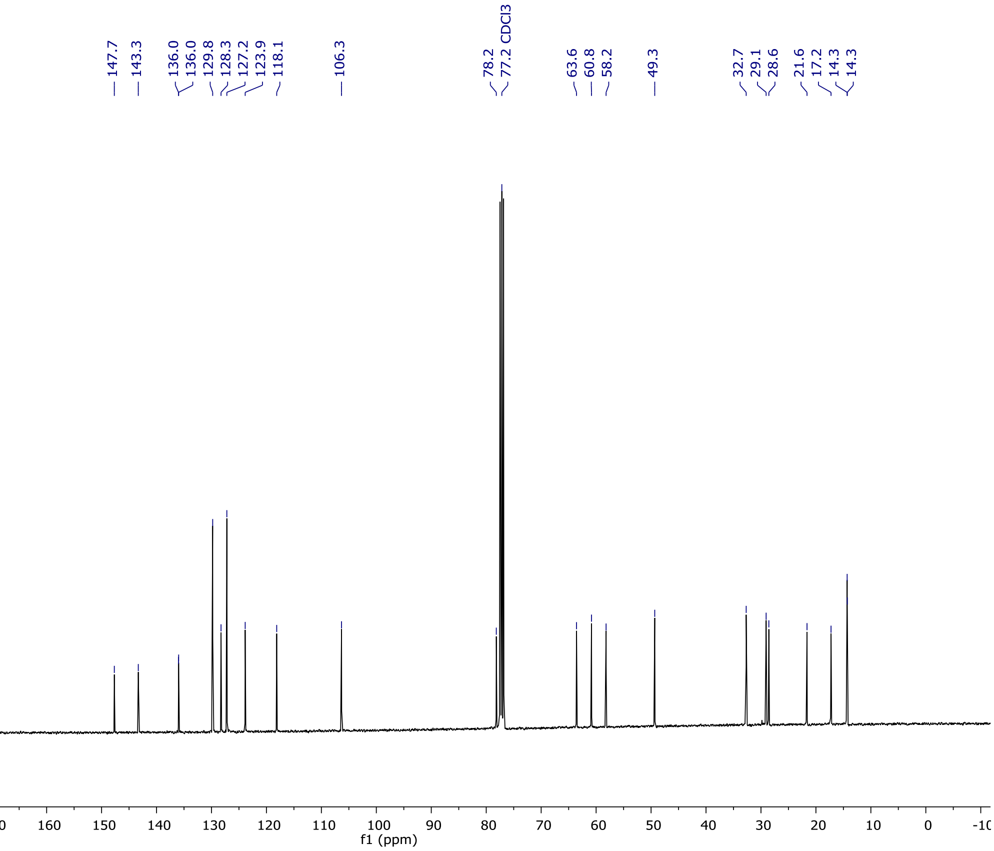

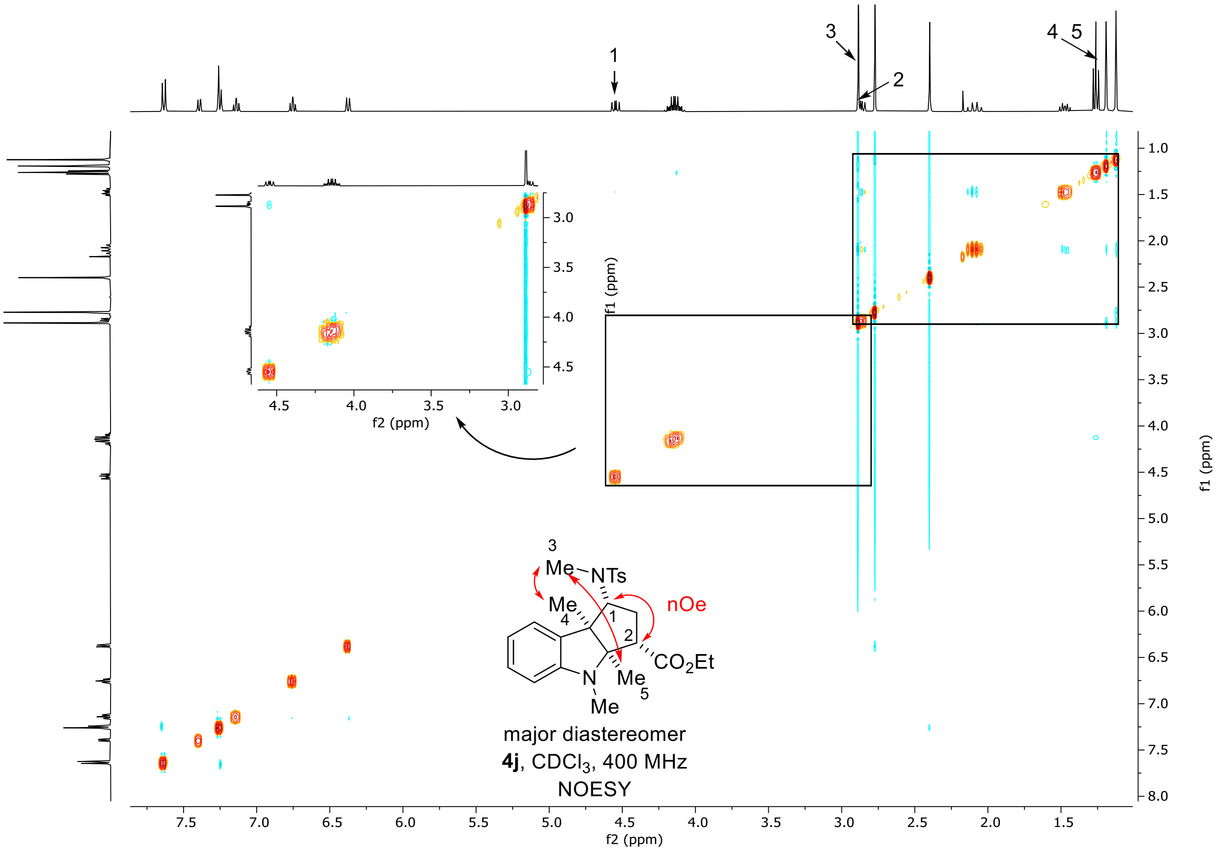

1
2
3
4 5
nOe
major diastereomer
4j, CDCl3, 400 MHz
NOESY
f2 (ppm)
f1 (ppm)

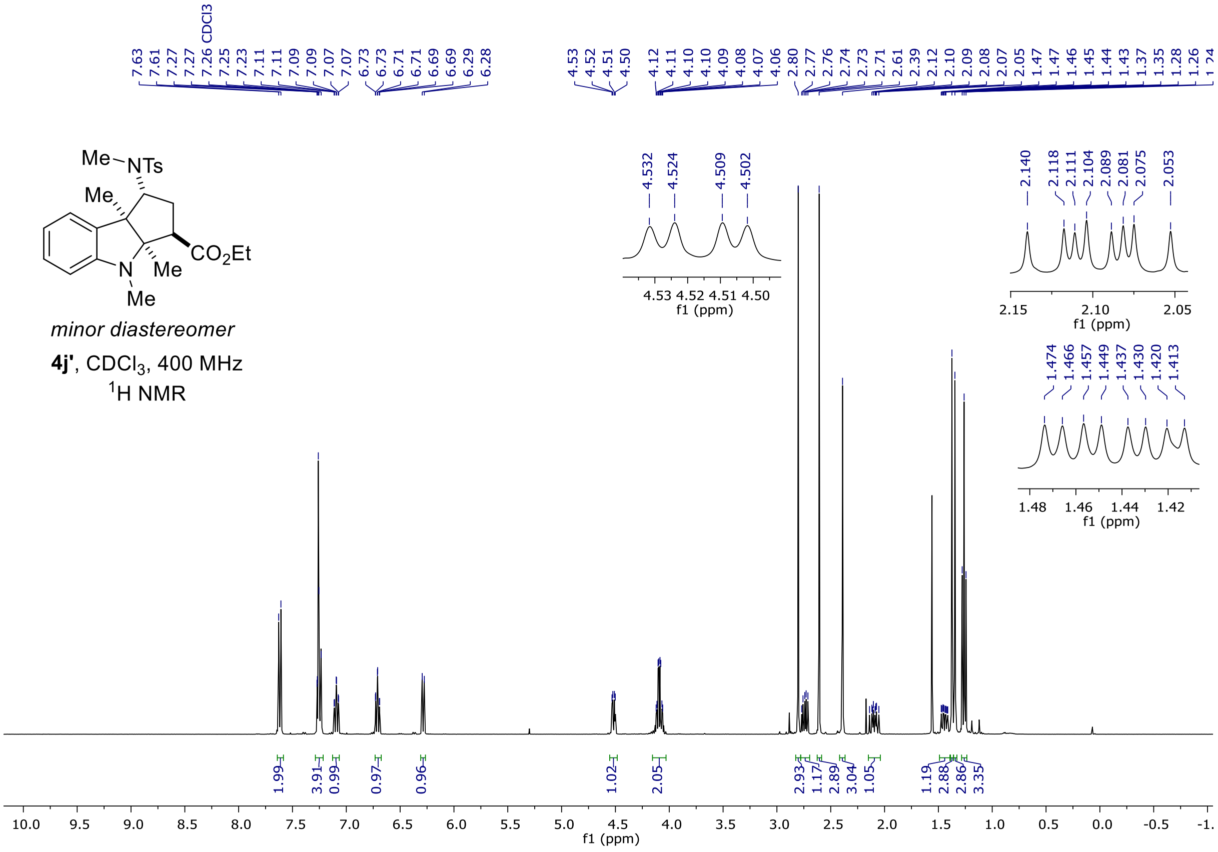
Me–NTs
Me
CO2Et
N
Me
Me
minor diastereomer
4j', CDCl3, 400 MHz
1H NMR
f1 (ppm)

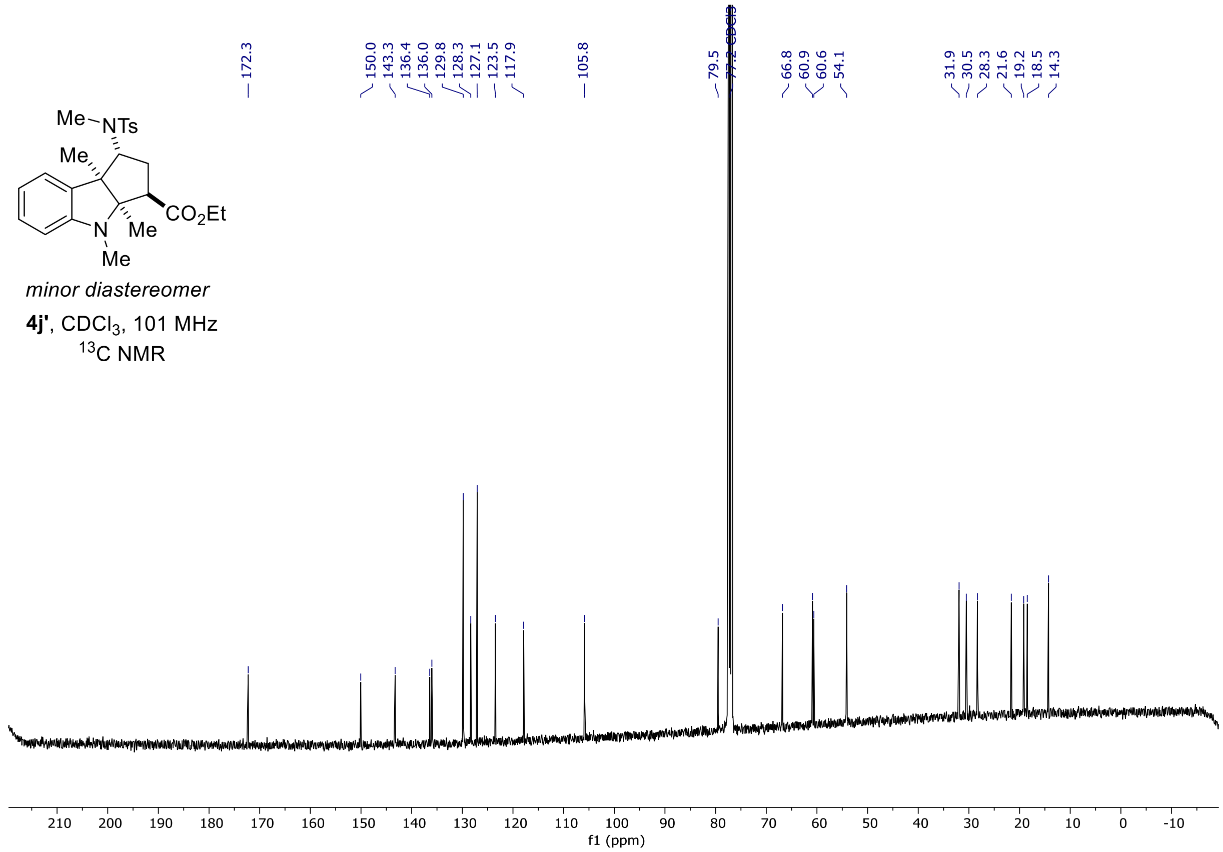
Me–NTs
Me
CO2Et
N
Me
Me
minor diastereomer
4j', CDCl3, 101 MHz
13C NMR
f1 (ppm)

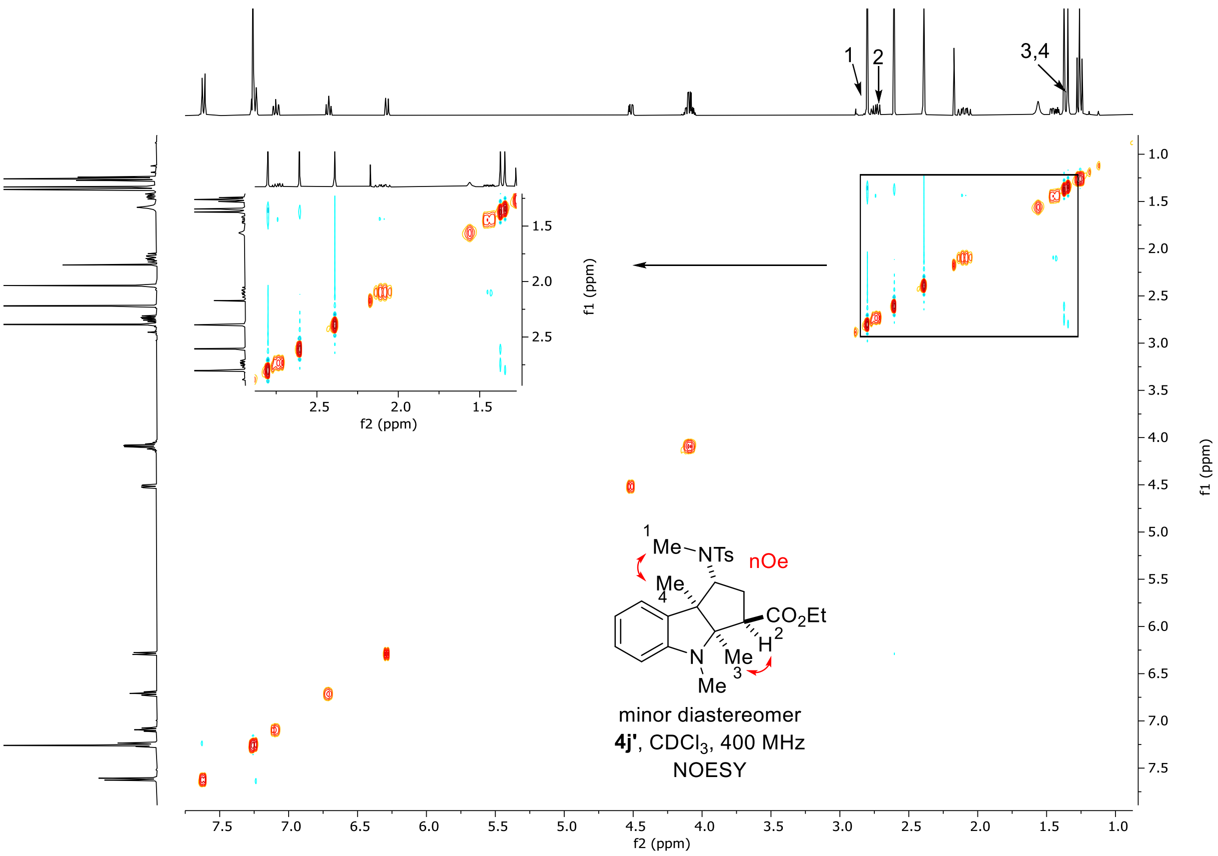

1
2
3,4
f1 (ppm)
f2 (ppm)
Me
NTs
nOe
CO2Et
H
N
Me
minor diastereomer
4j', CDCl3, 400 MHz
NOESY

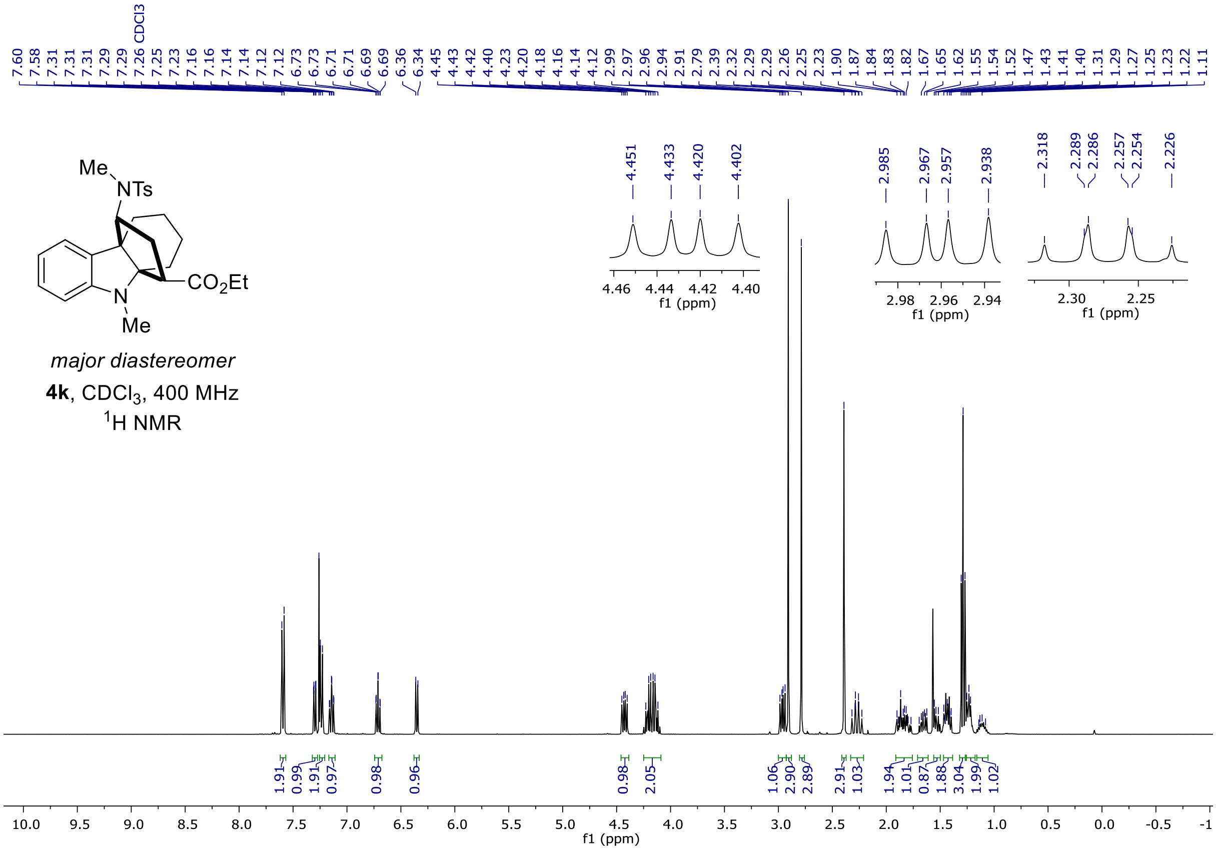

Me
NTs
CO_2Et
N
Me
major diastereomer
4k, $CDCl_3$, 400 MHz
^1H NMR
7.60 7.58 7.31 7.31 7.29 7.29 7.26 CDCl3 7.25 7.23 7.16 7.16 7.14 7.14 7.12 7.12 6.73 6.73 6.71 6.71 6.69 6.69 6.36 6.34 4.45 4.43 4.42 4.40 4.23 4.20 4.18 4.16 4.14 4.12 2.99 2.97 2.96 2.94 2.91 2.79 2.39 2.32 2.29 2.29 2.26 2.25 2.23 1.90 1.87 1.84 1.83 1.82 1.67 1.65 1.62 1.55 1.54 1.52 1.47 1.43 1.41 1.40 1.31 1.29 1.27 1.25 1.23 1.22 1.11
4.451 4.433 4.420 4.402
4.46 4.44 4.42 4.40
f1 (ppm)
2.985 2.967 2.957 2.938
2.98 2.96 2.94
f1 (ppm)
2.318 2.289 2.286 2.257 2.254 2.226
2.30 2.25
f1 (ppm)
1.91 0.99 1.91 0.97 0.98 0.96 0.98 2.05 1.06 2.90 2.89 2.91 1.03 1.94 1.01 0.87 1.88 3.04 1.99 1.02
10.0 9.5 9.0 8.5 8.0 7.5 7.0 6.5 6.0 5.5 5.0 4.5 4.0 3.5 3.0 2.5 2.0 1.5 1.0 0.5 0.0 -0.5 -1
f1 (ppm)

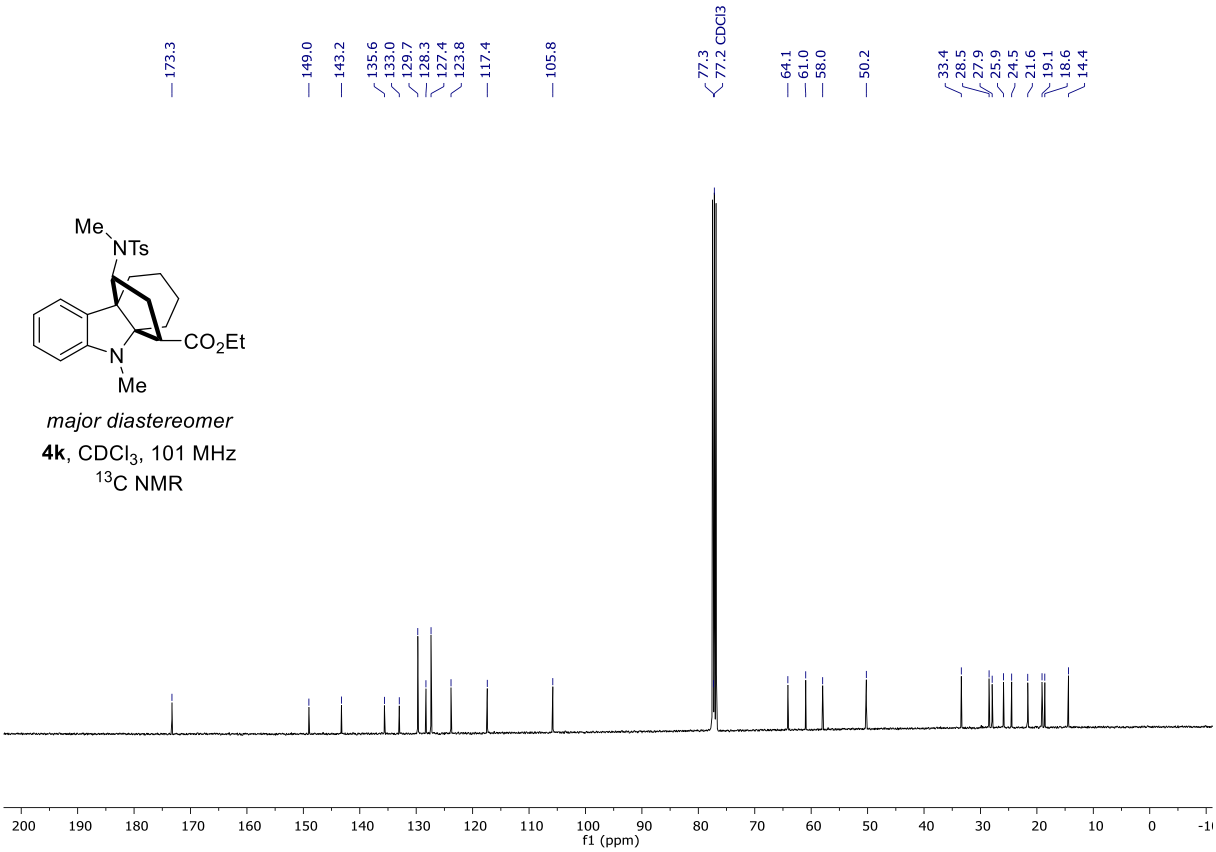

173.3 149.0 143.2 135.6 133.0 129.7 128.3 127.4 123.8 117.4 105.8 77.3 77.2 CDCl3 64.1 61.0 58.0 50.2 33.4 28.5 27.9 25.9 24.5 21.6 19.1 18.6 14.4
Me
NTs
CO_2Et
N
Me
major diastereomer
4k, $CDCl_3$, 101 MHz
^{13}C NMR
200 190 180 170 160 150 140 130 120 110 100 90 80 70 60 50 40 30 20 10 0 -10
f1 (ppm)

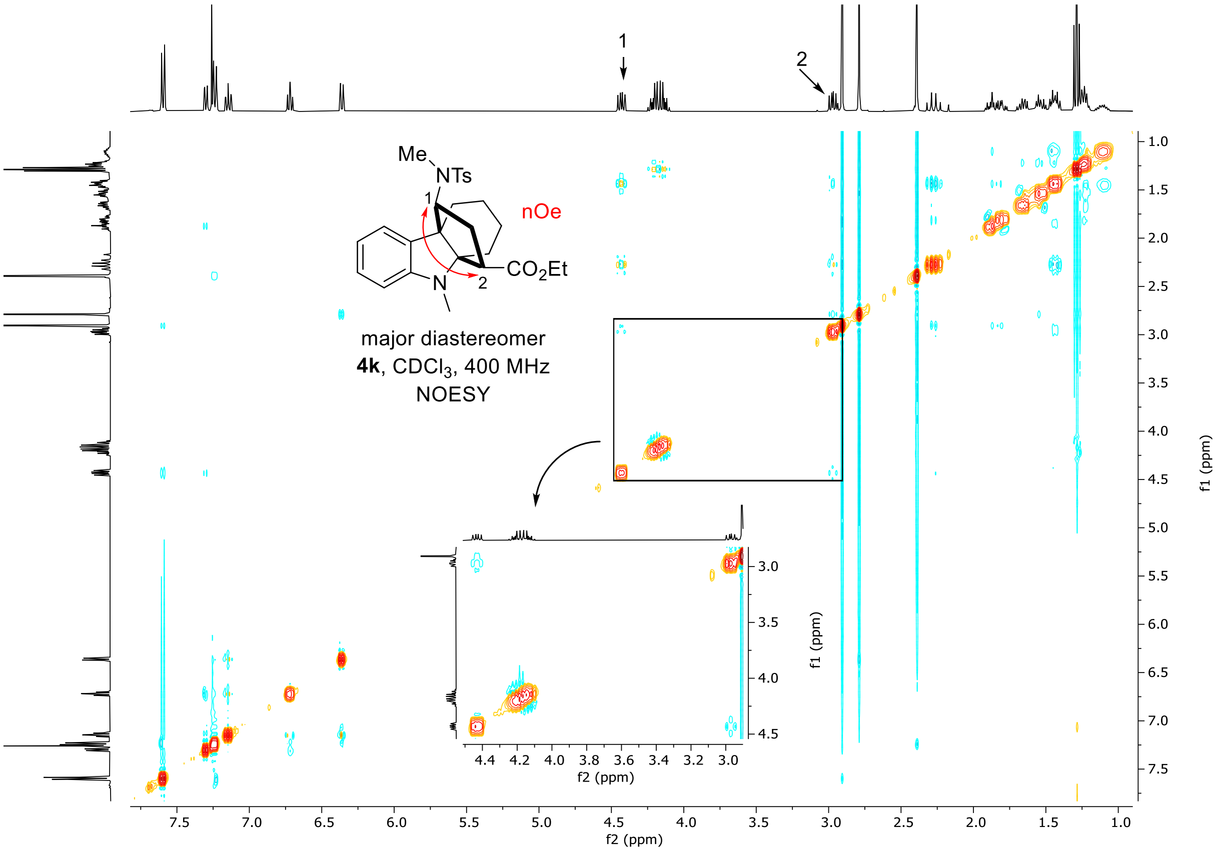
1
2
Me
NTs
1
nOe
CO2Et
2
N
major diastereomer
4k, CDCl3, 400 MHz
NOESY
f1 (ppm)
f2 (ppm)
f1 (ppm)
f2 (ppm)

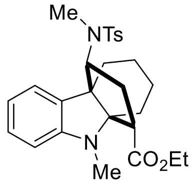

minor diastereomer

4k', $CDCl_3$, 400 MHz

^1H NMR

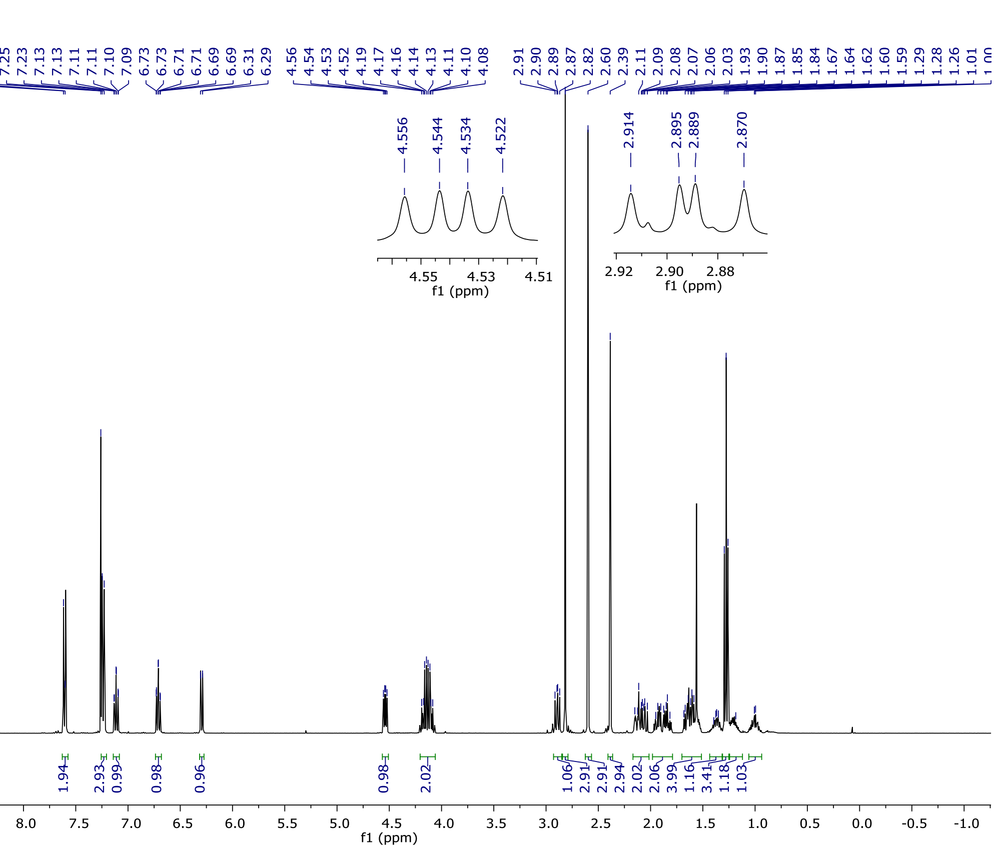

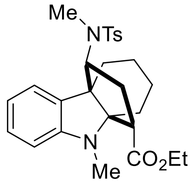

minor diastereomer

4k', $CDCl_3$, 101 MHz

^{13}C NMR

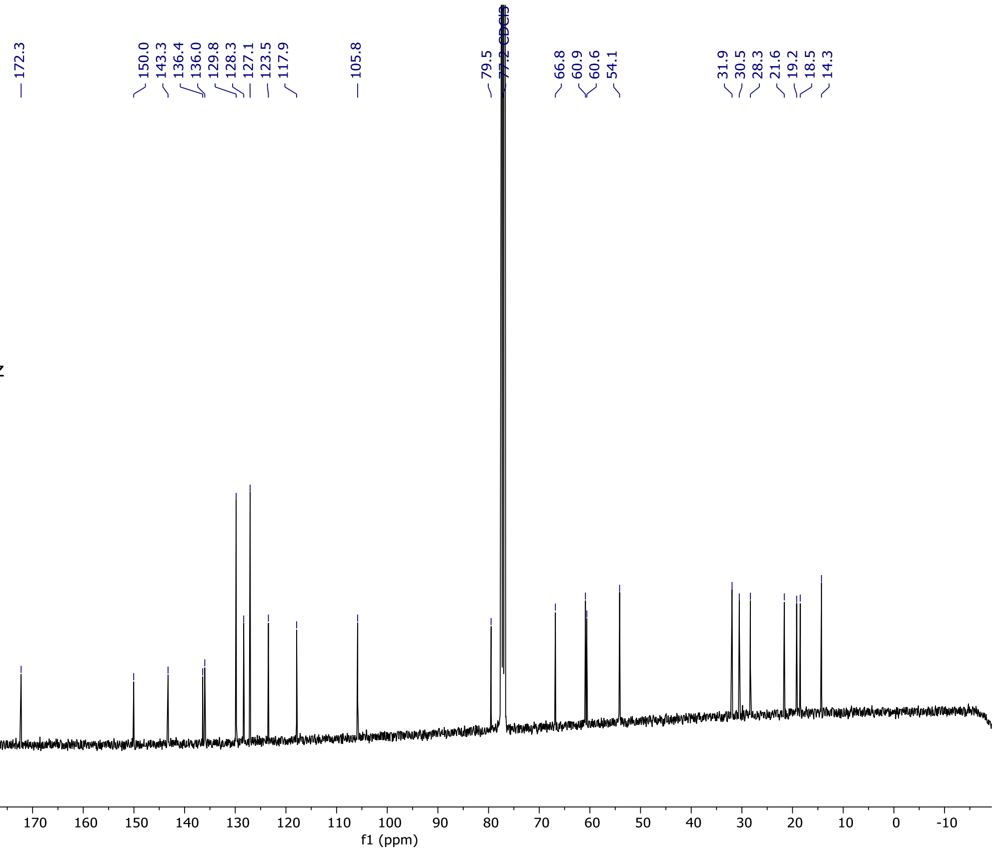

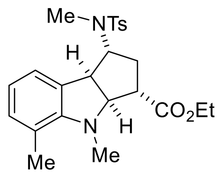

4l, $CDCl_3$, 400 MHz
^1H NMR

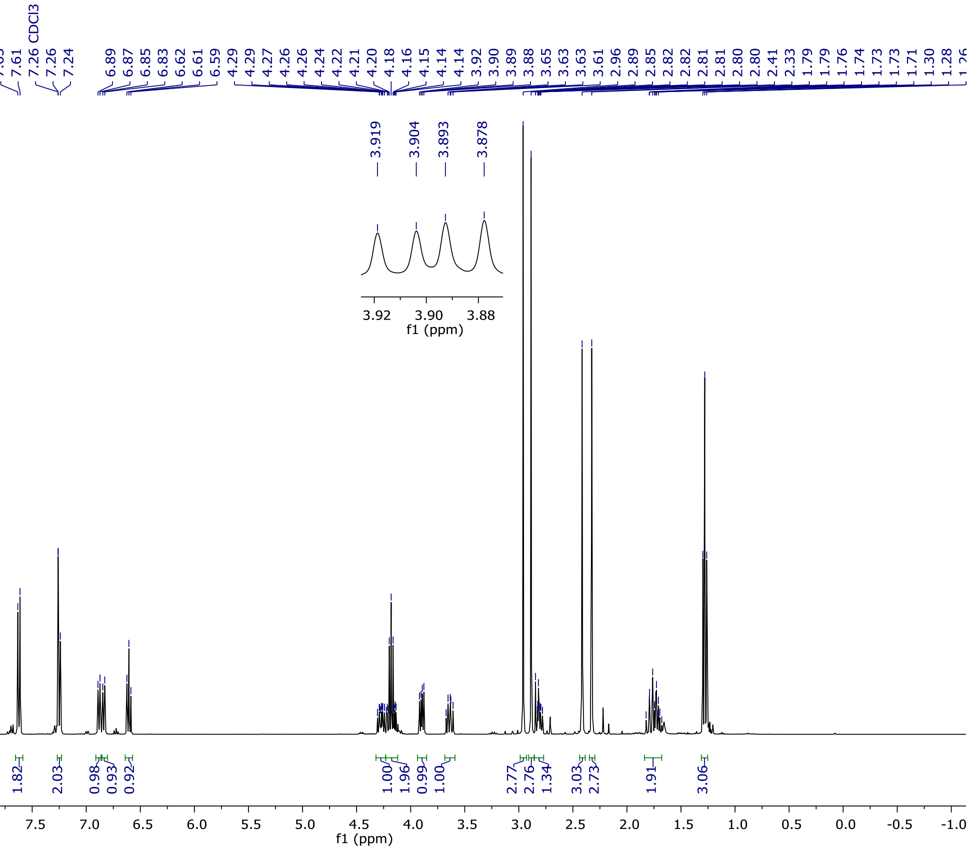

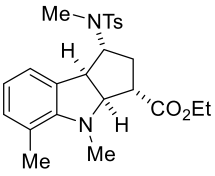

4l, $CDCl_3$, 101 MHz
^{13}C NMR

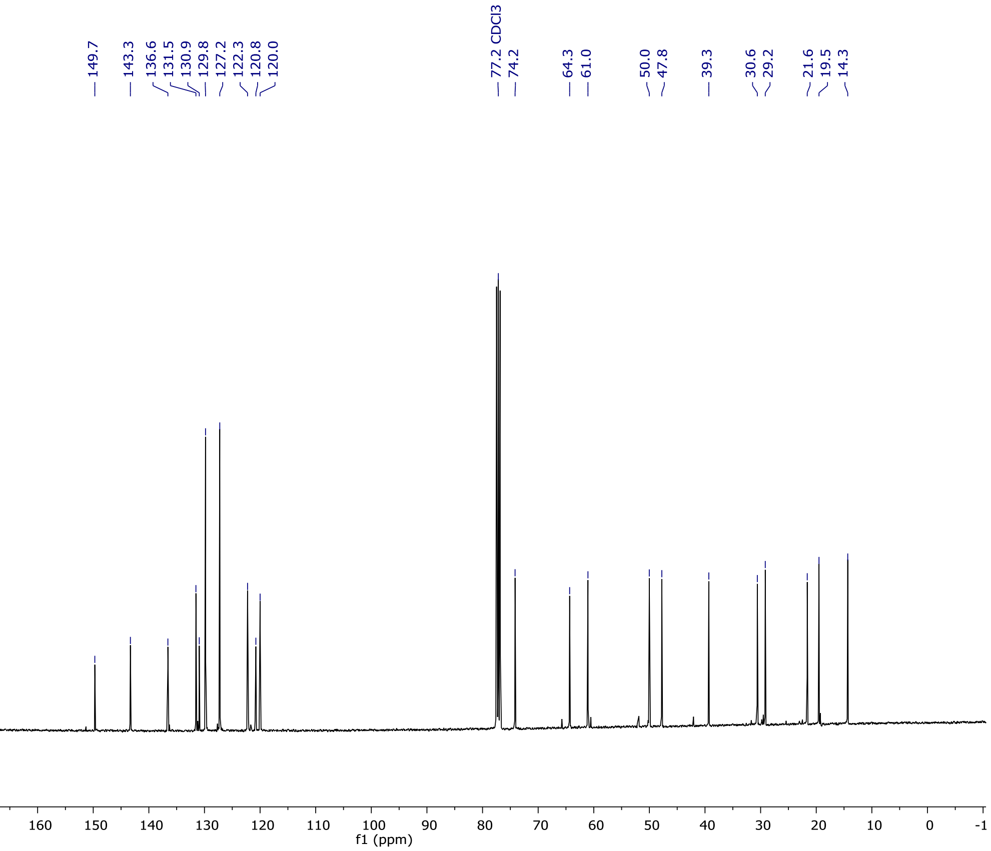

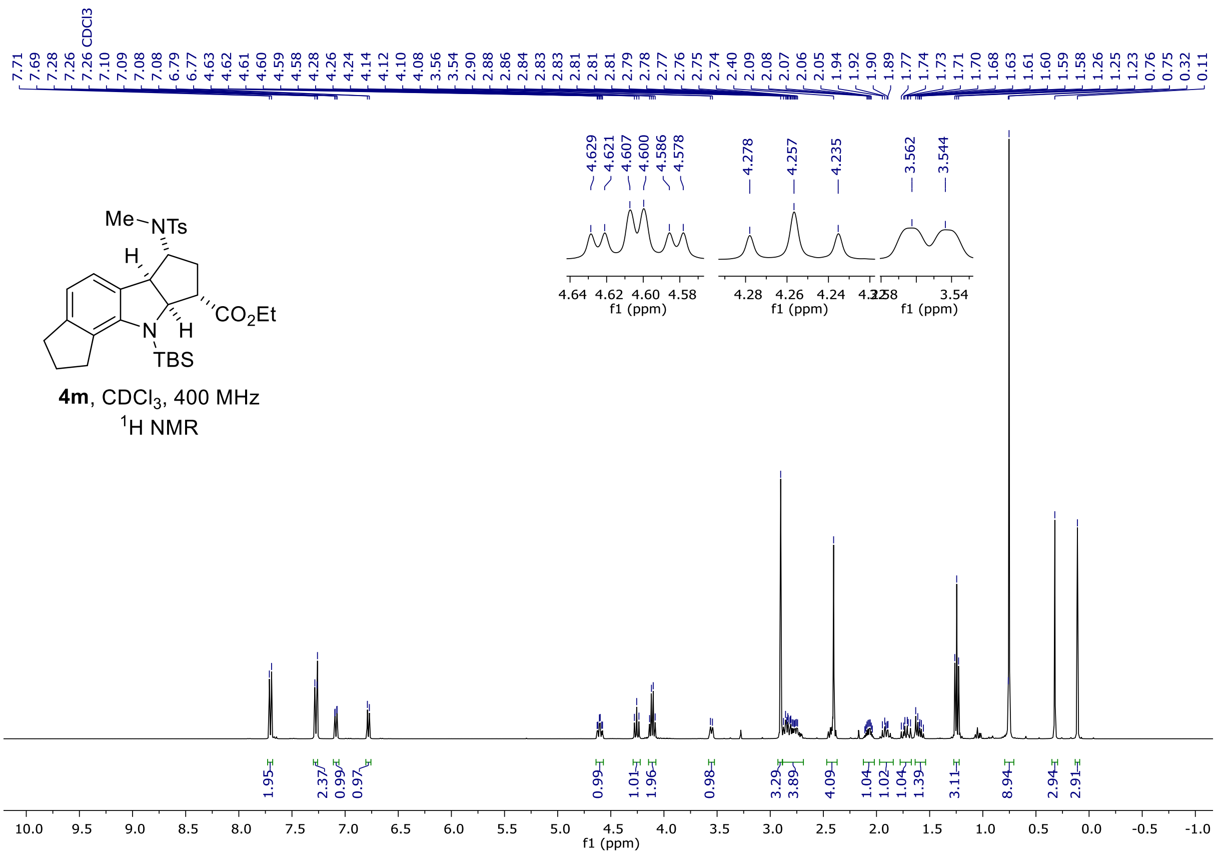

4m, $CDCl_3$, 400 MHz
^1H NMR

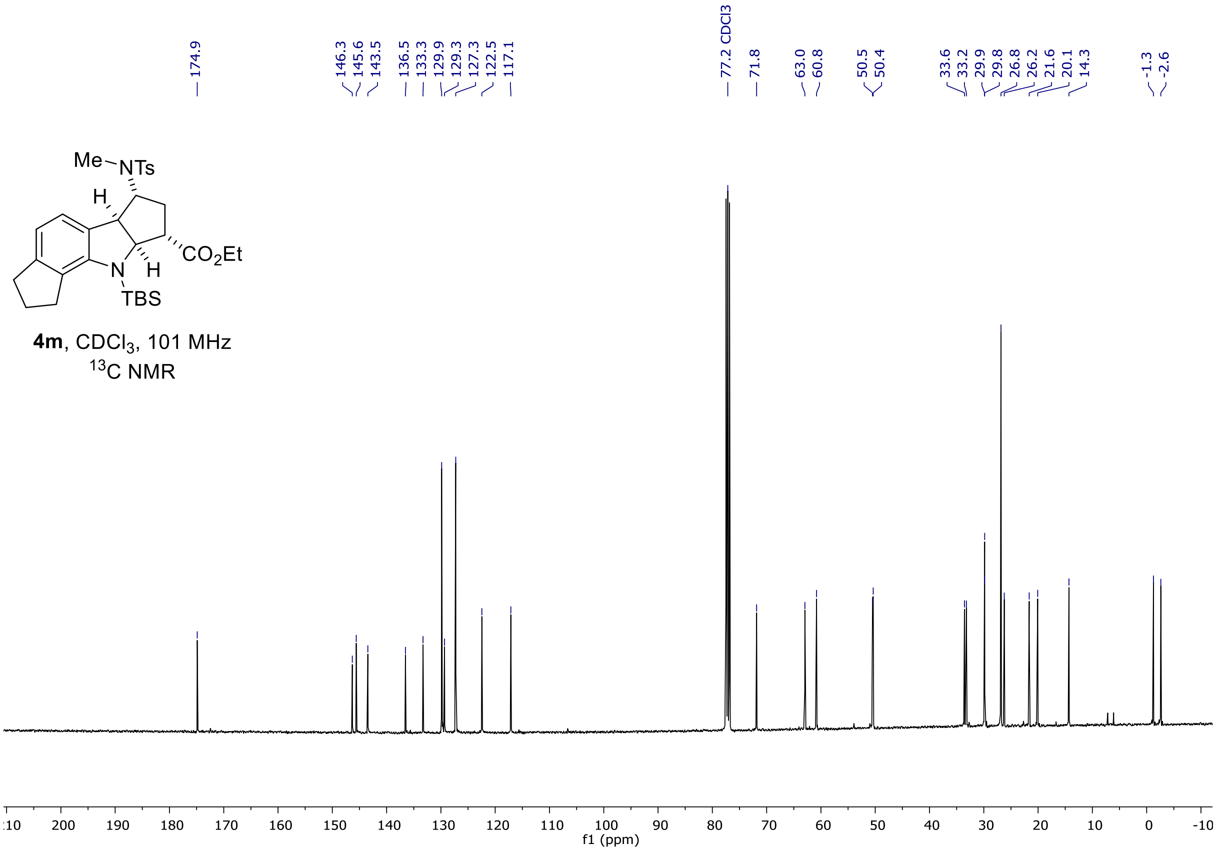

4m, $CDCl_3$, 101 MHz
^{13}C NMR

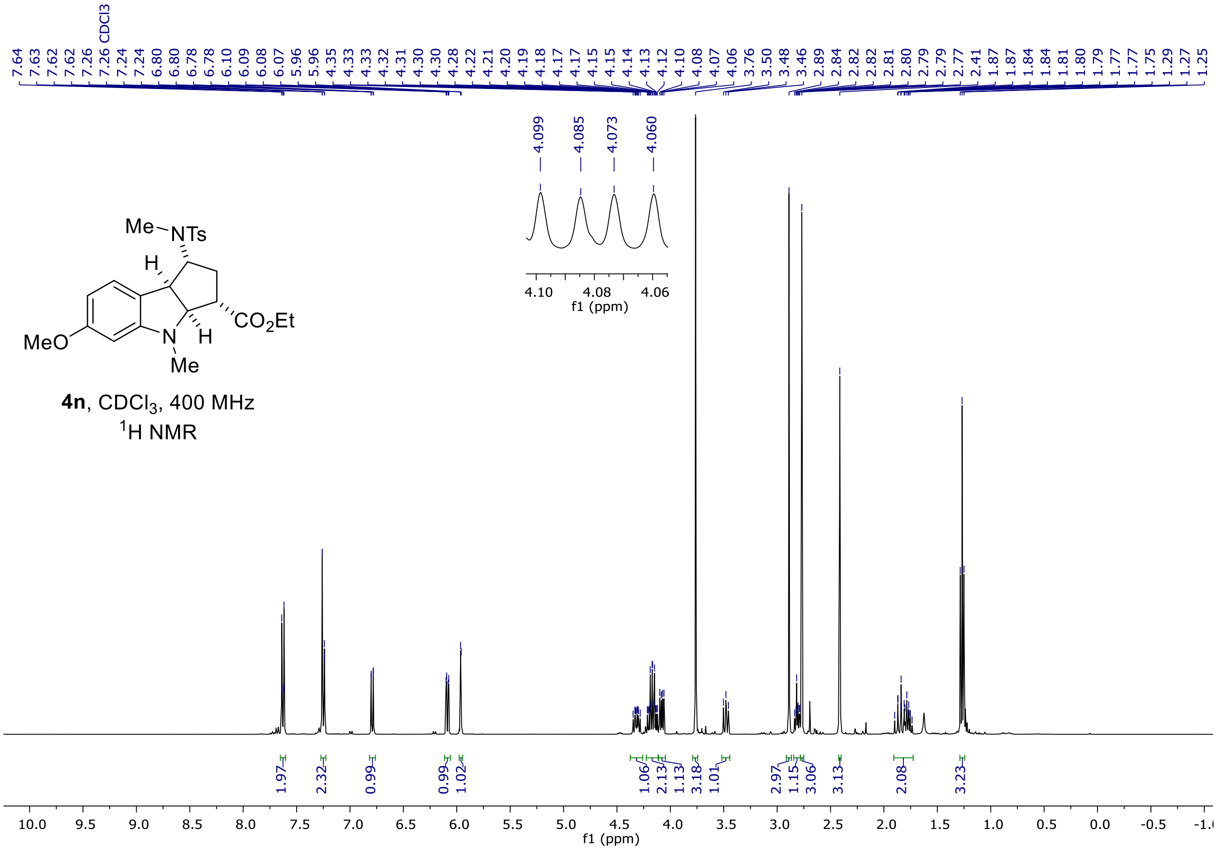

4n, $CDCl_3$, 400 MHz

^1H NMR

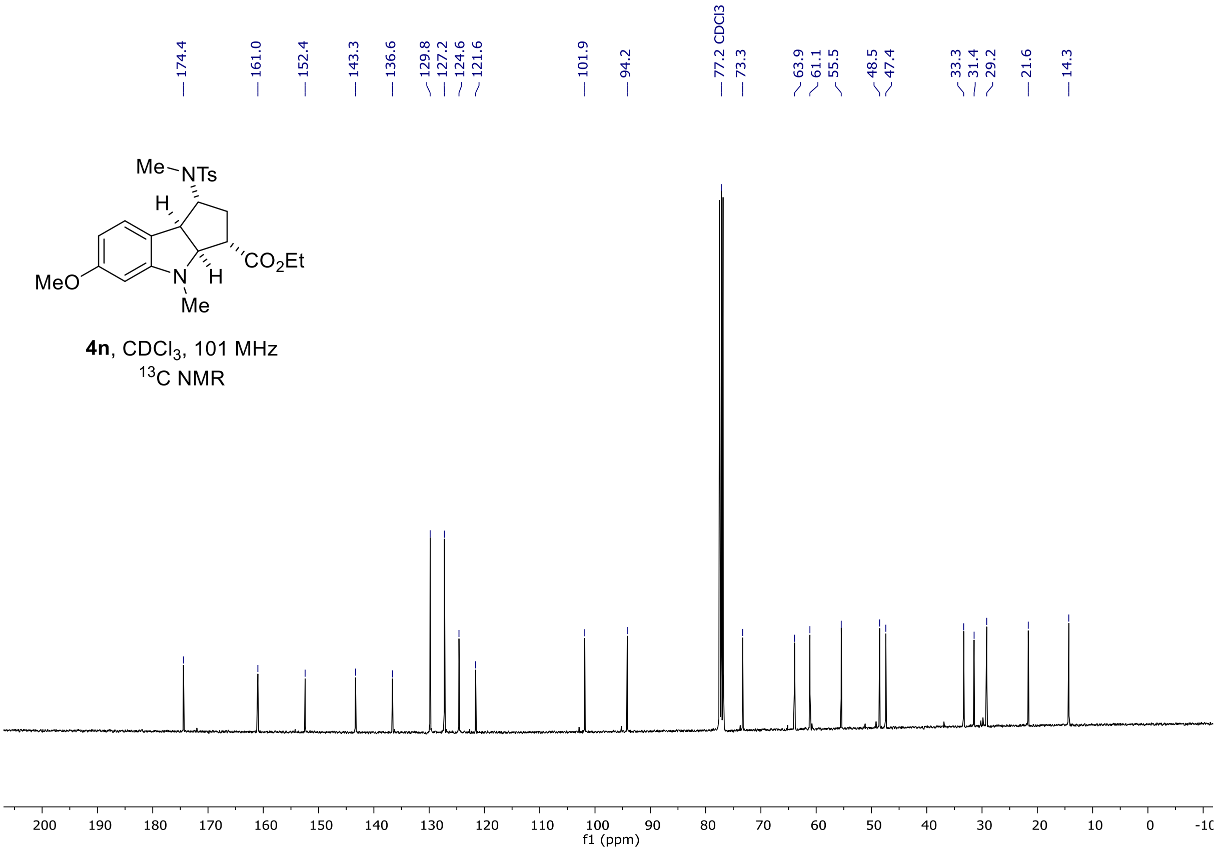

4n, $CDCl_3$, 101 MHz

^{13}C NMR

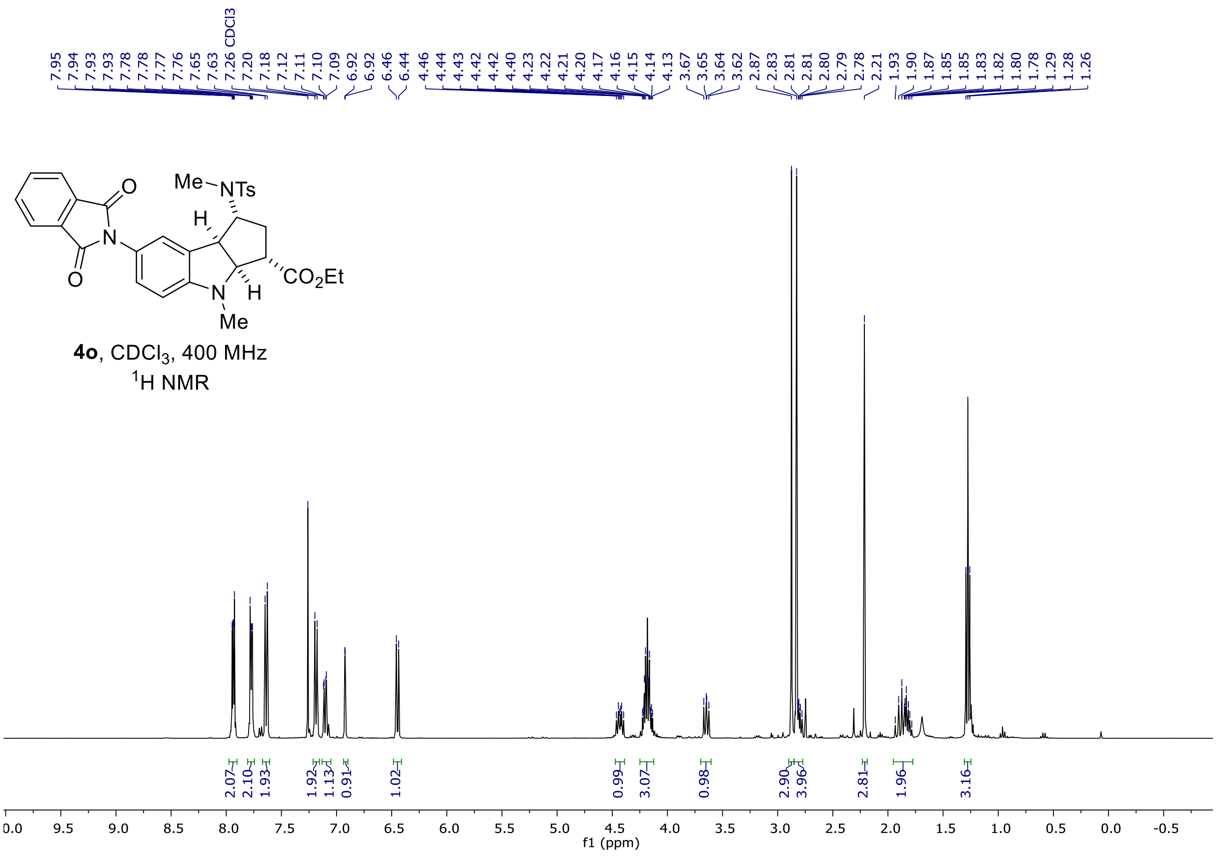

4o, $CDCl_3$, 400 MHz
^{1}H NMR
f1 (ppm)

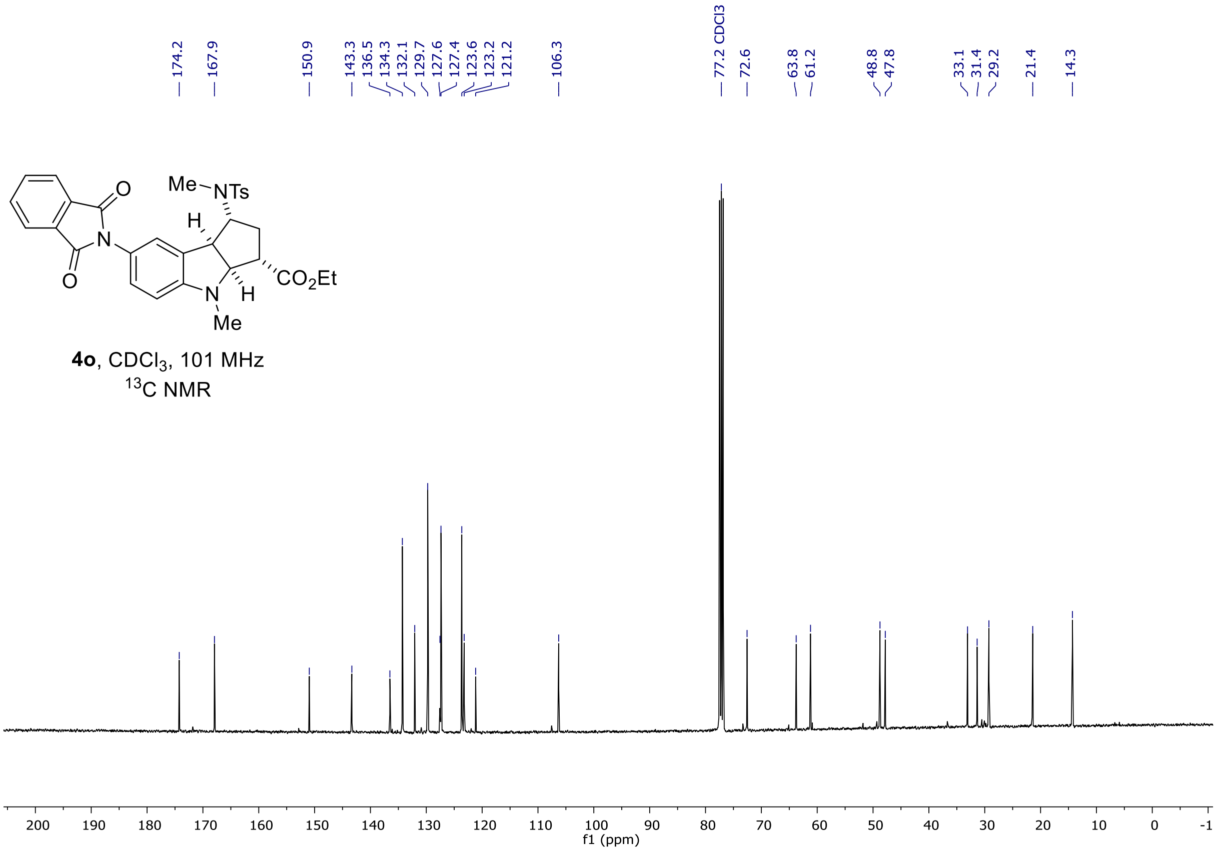

4o, $CDCl_3$, 101 MHz
^{13}C NMR
f1 (ppm)

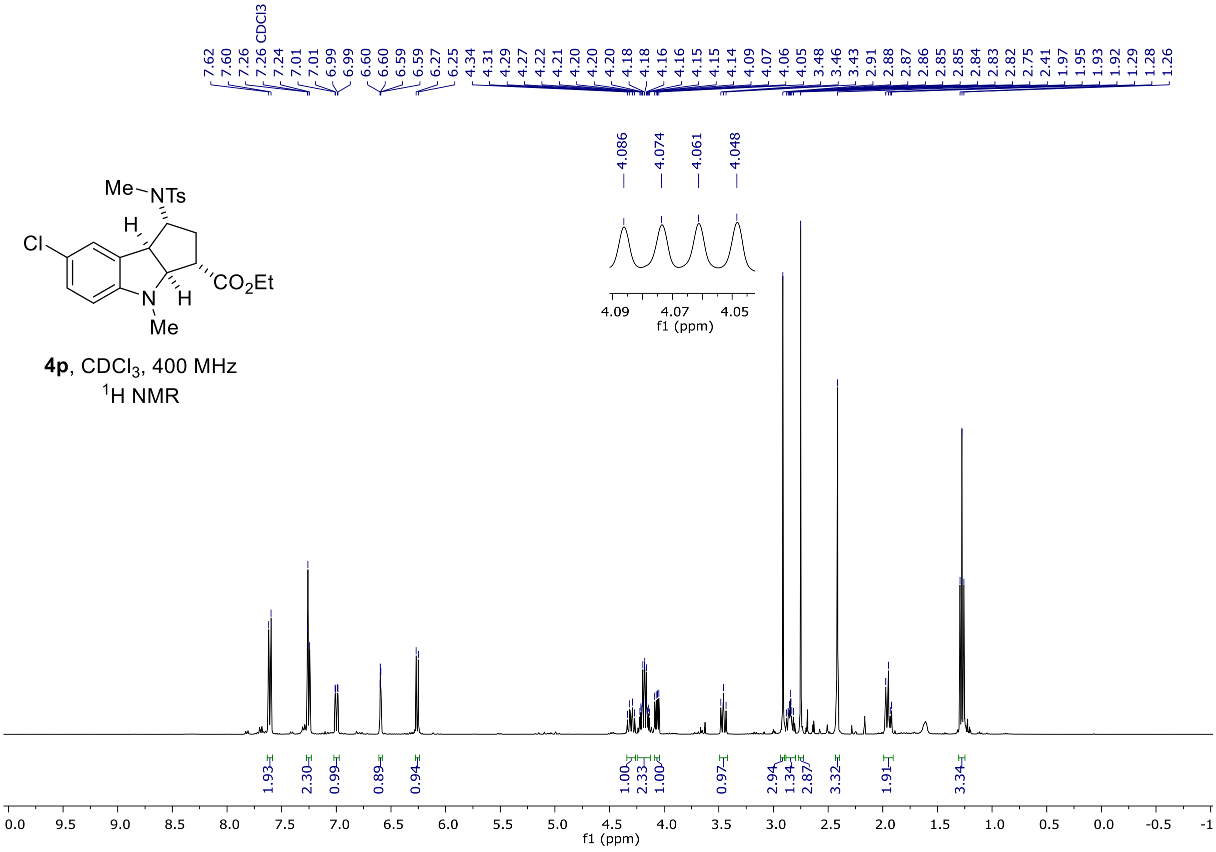

4p, $CDCl_3$, 400 MHz
^1H NMR

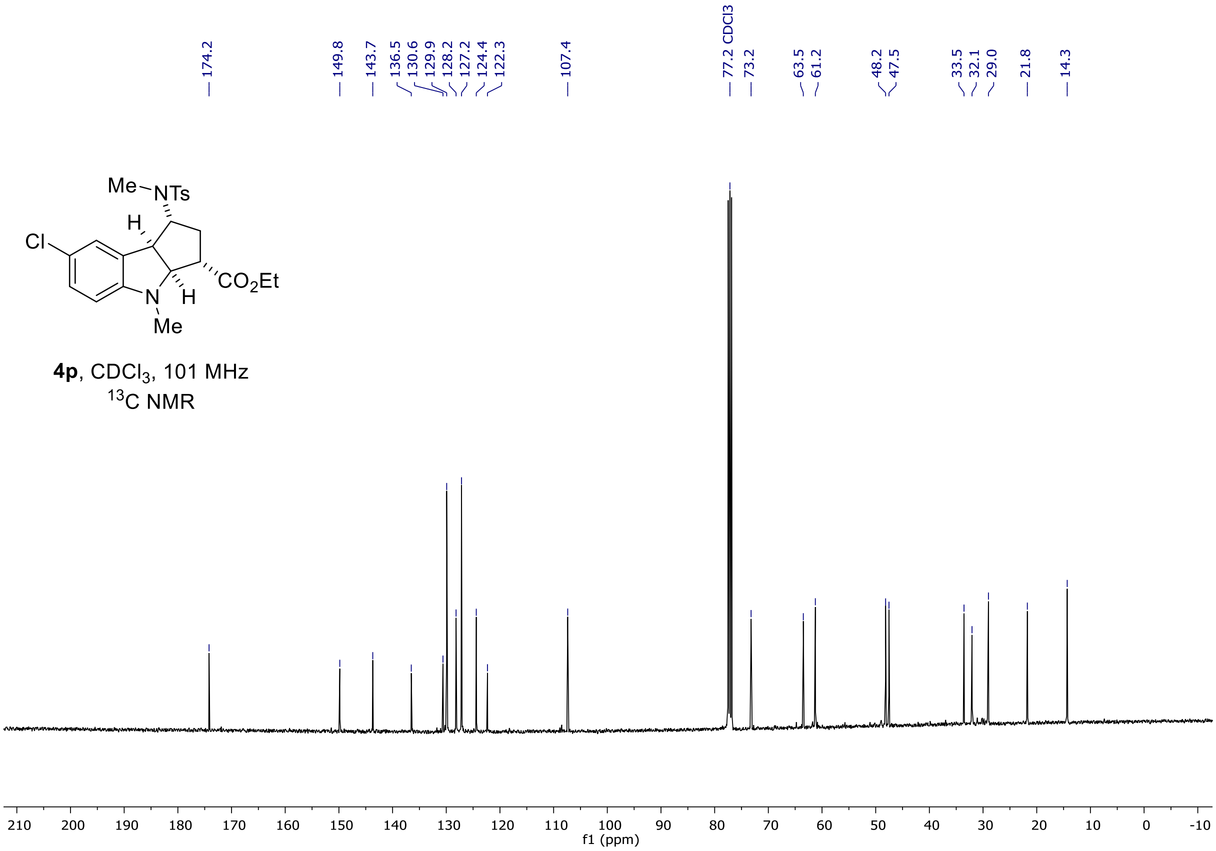

4p, $CDCl_3$, 101 MHz
^{13}C NMR

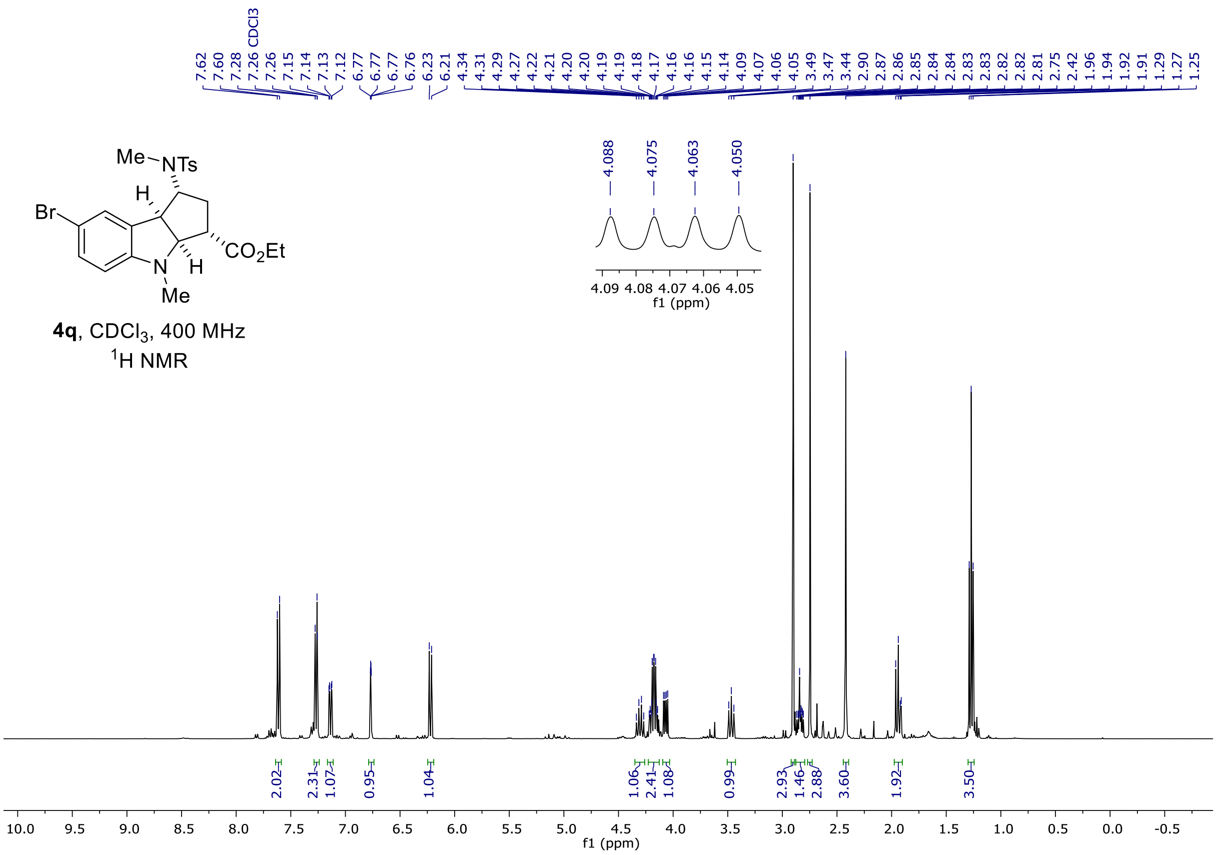

4q, $CDCl_3$, 400 MHz

^1H NMR

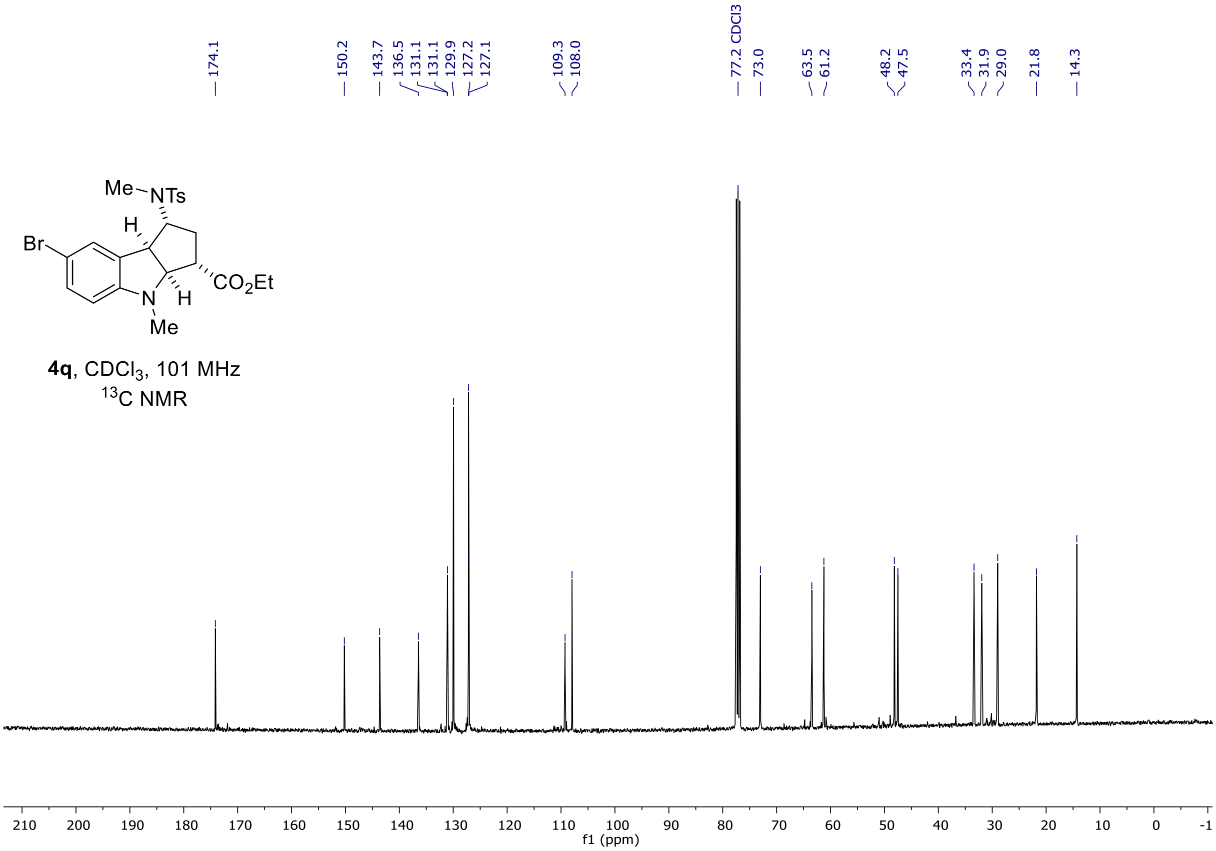

4q, $CDCl_3$, 101 MHz

^{13}C NMR

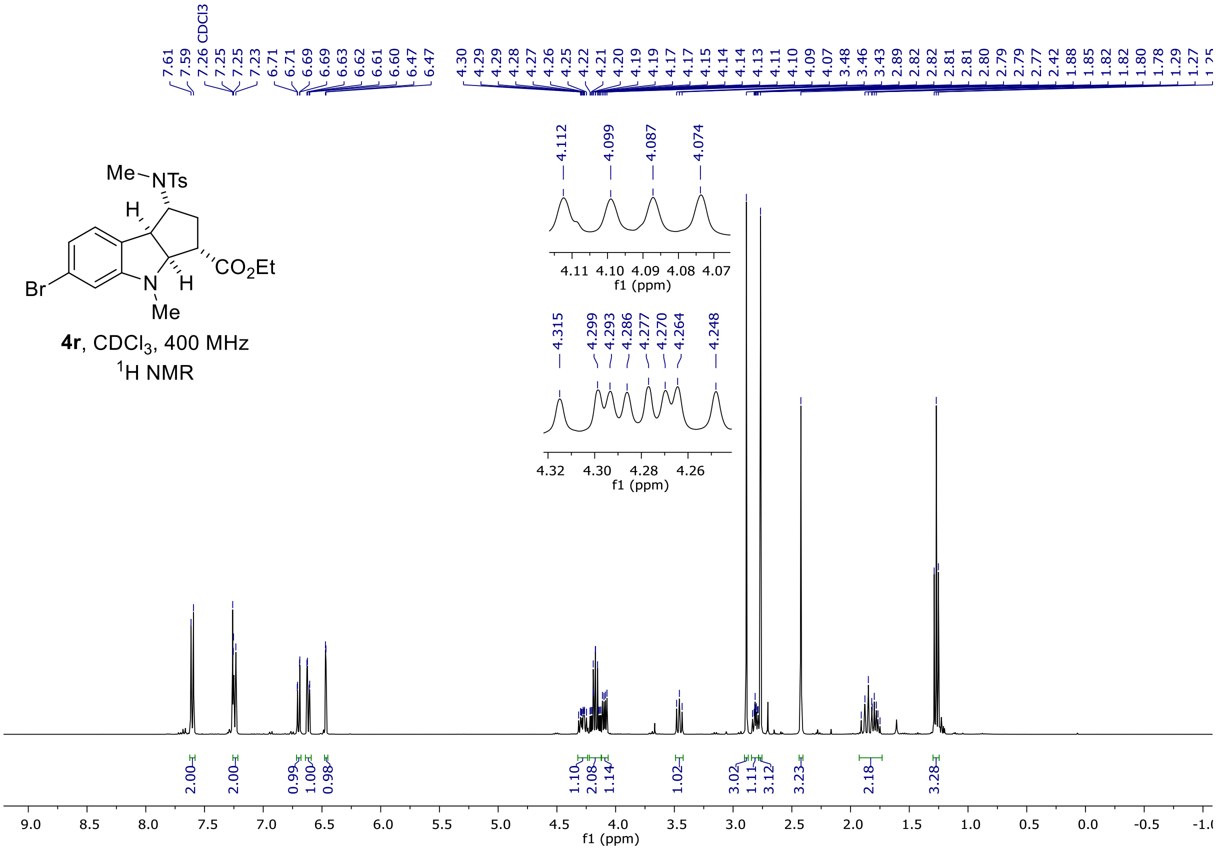

4r, $CDCl_3$, 400 MHz

^1H NMR

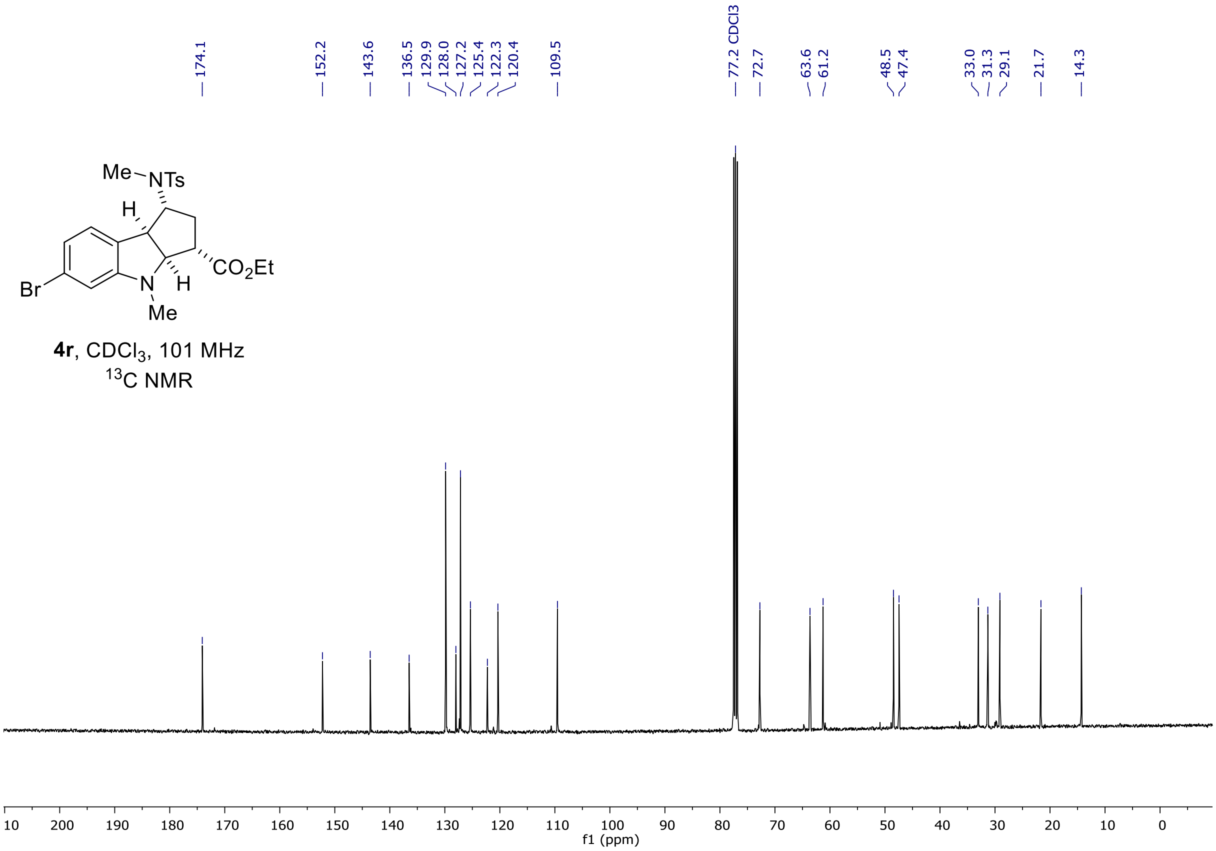

4r, $CDCl_3$, 101 MHz

^{13}C NMR

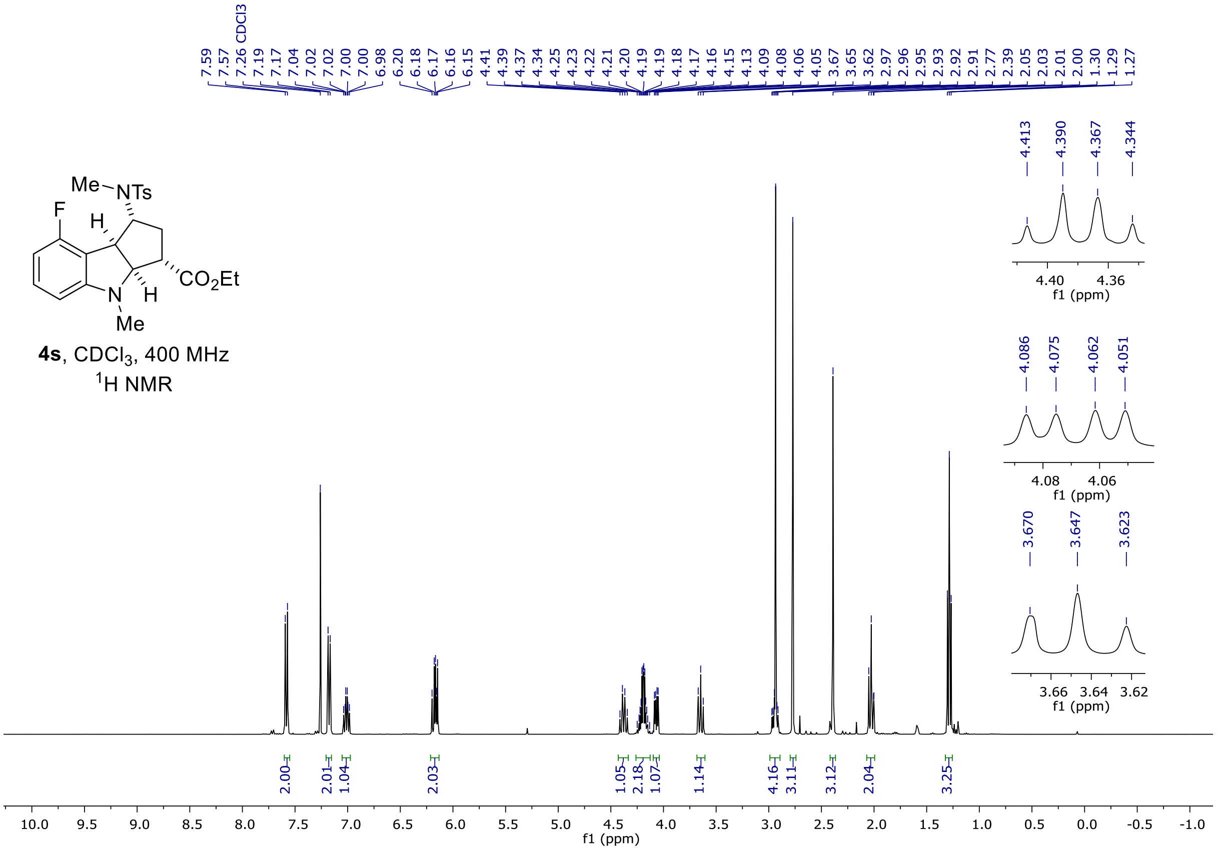
7.59 7.57 7.26 CDCl3 7.19 7.17 7.04 7.02 7.02 7.00 7.00 6.98 6.20 6.18 6.17 6.16 6.15 4.41 4.39 4.37 4.34 4.25 4.23 4.22 4.21 4.20 4.19 4.19 4.18 4.17 4.16 4.15 4.13 4.09 4.08 4.06 4.05 3.67 3.65 3.62 2.97 2.96 2.95 2.93 2.92 2.91 2.77 2.39 2.05 2.03 2.01 2.00 1.30 1.29 1.27
Me
NTs
F
H
CO2Et
N
H
Me
4s, CDCl3, 400 MHz
1H NMR
4.413 4.390 4.367 4.344
4.40 4.36
f1 (ppm)
4.086 4.075 4.062 4.051
4.08 4.06
f1 (ppm)
3.670 3.647 3.623
3.66 3.64 3.62
f1 (ppm)
2.00 2.01 1.04 2.03 1.05 2.18 1.07 1.14 4.16 3.11 3.12 2.04 3.25
10.0 9.5 9.0 8.5 8.0 7.5 7.0 6.5 6.0 5.5 5.0 4.5 4.0 3.5 3.0 2.5 2.0 1.5 1.0 0.5 0.0 -0.5 -1.0
f1 (ppm)

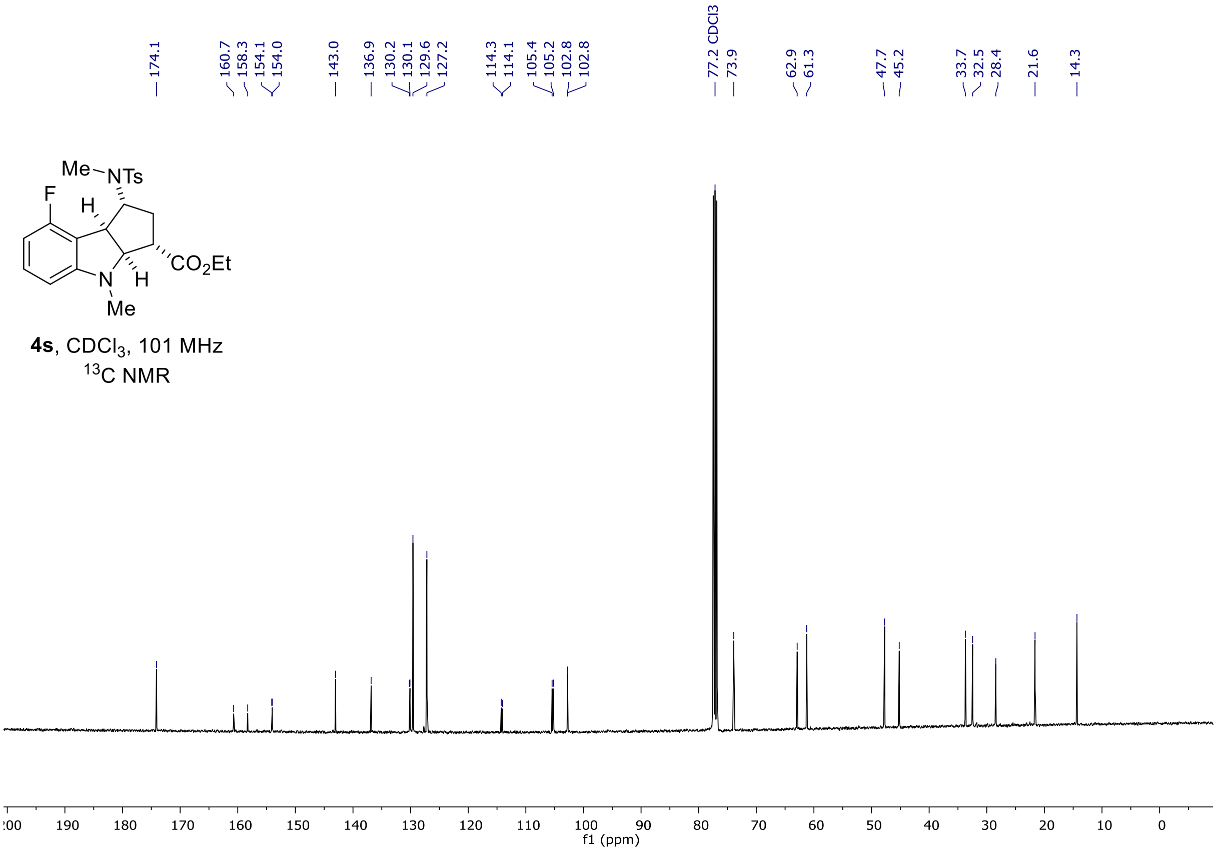
174.1 160.7 158.3 154.1 154.0 143.0 136.9 130.2 130.1 129.6 127.2 114.3 114.1 105.4 105.2 102.8 102.8 77.2 CDCl3 73.9 62.9 61.3 47.7 45.2 33.7 32.5 28.4 21.6 14.3
Me
NTs
F
H
CO2Et
N
H
Me
4s, CDCl3, 101 MHz
13C NMR
200 190 180 170 160 150 140 130 120 110 100 90 80 70 60 50 40 30 20 10 0
f1 (ppm)

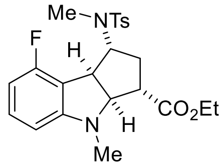

4s, $CDCl_3$, 376 MHz
^{19}F NMR

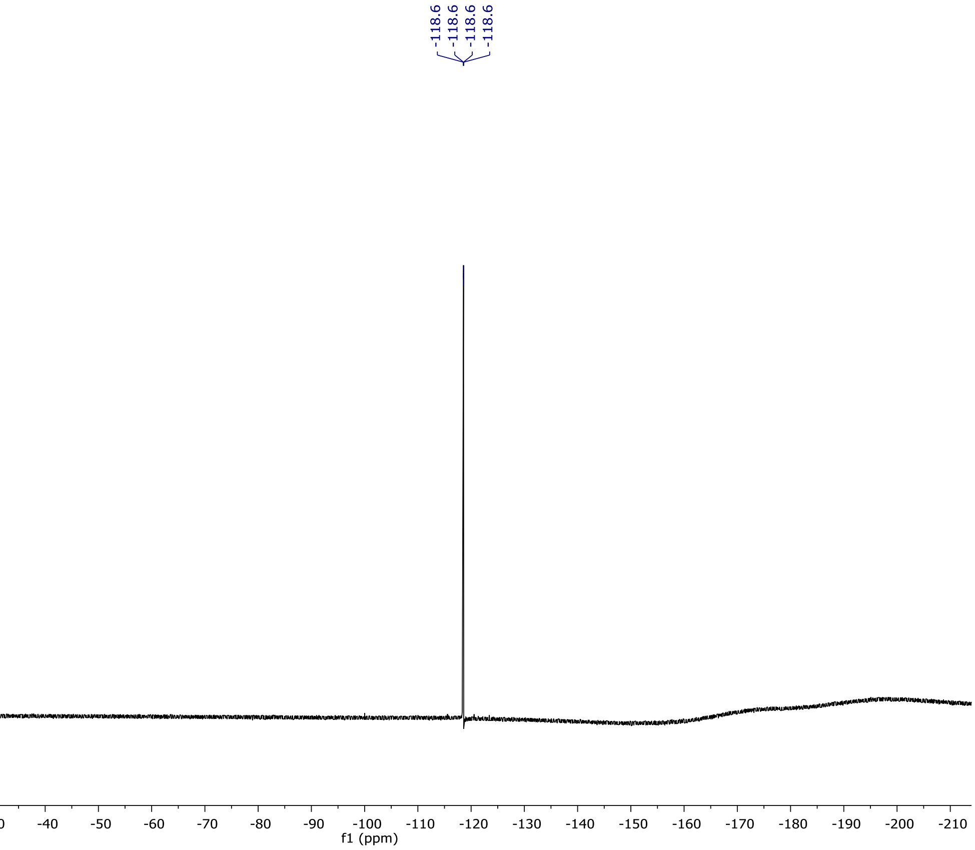

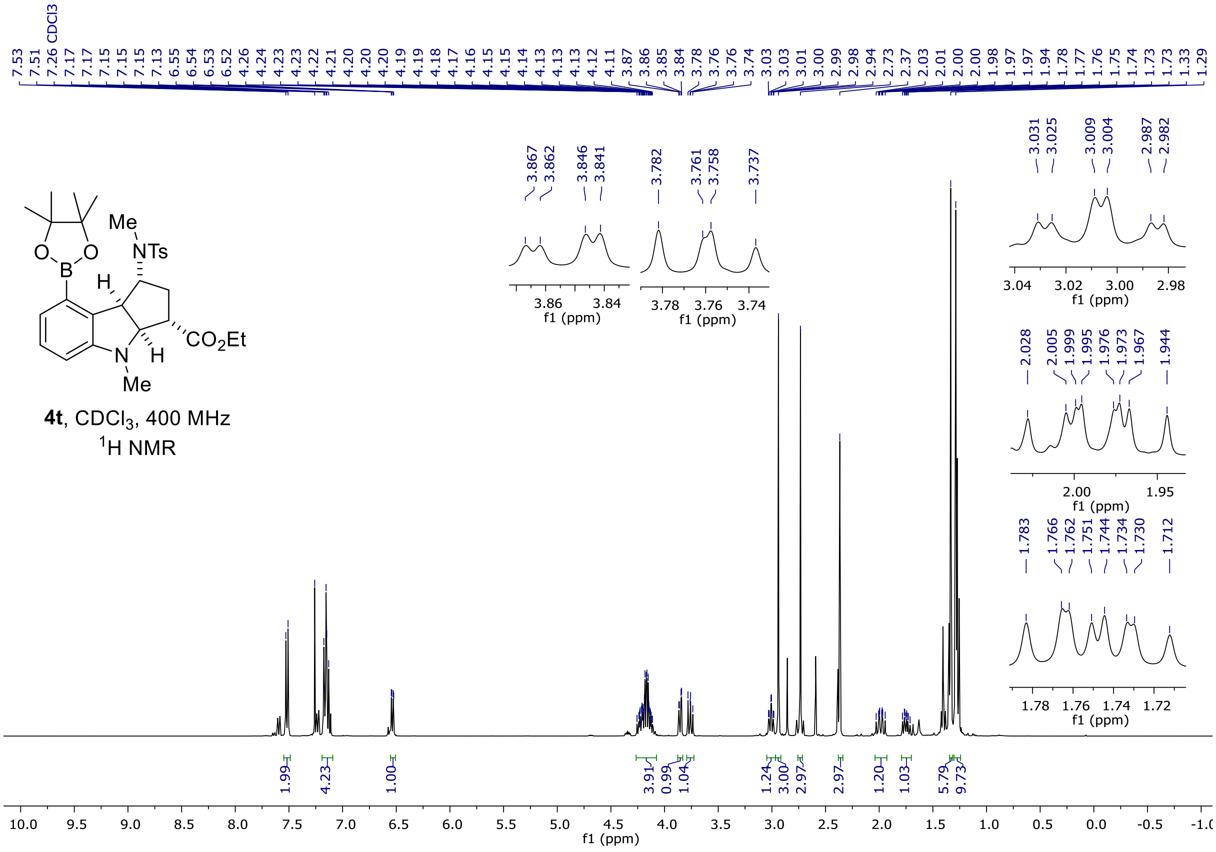

4t, $CDCl_3$, 400 MHz
^1H NMR

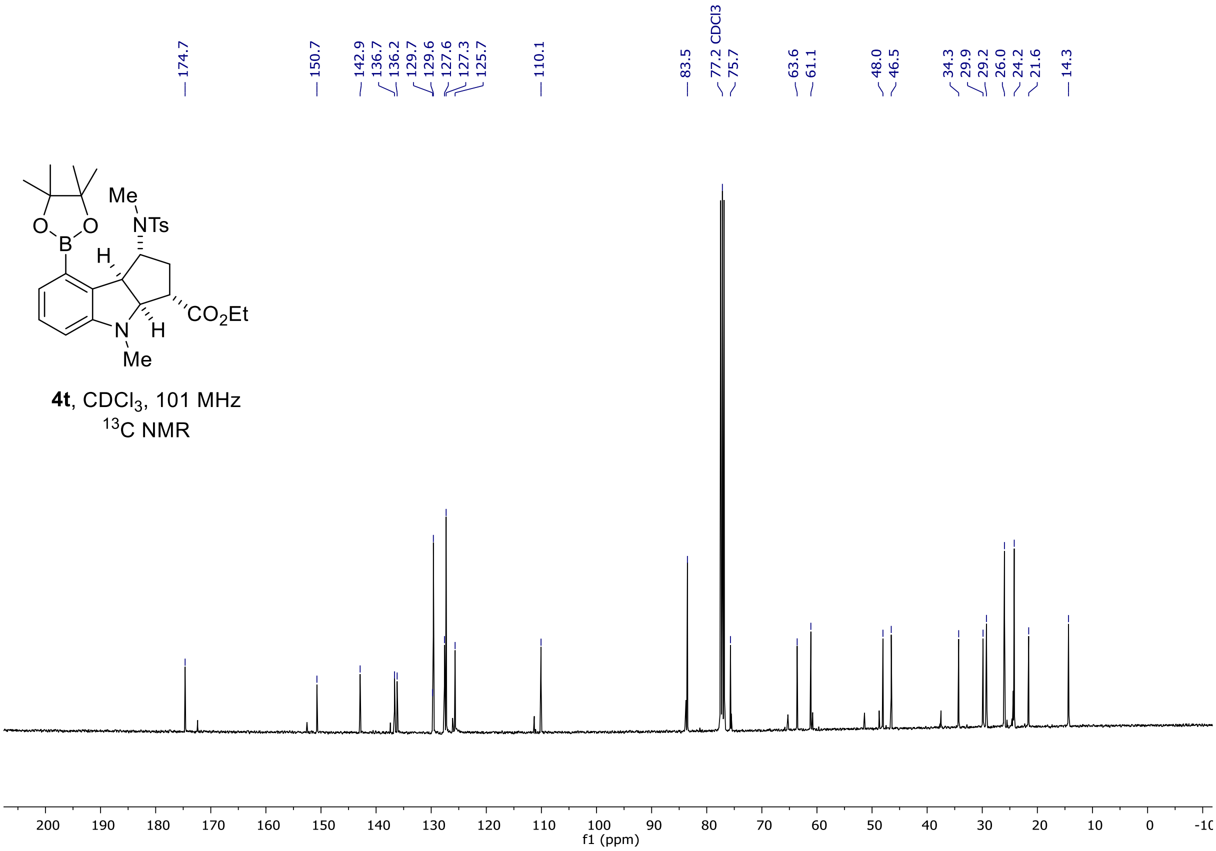

4t, $CDCl_3$, 101 MHz
^{13}C NMR

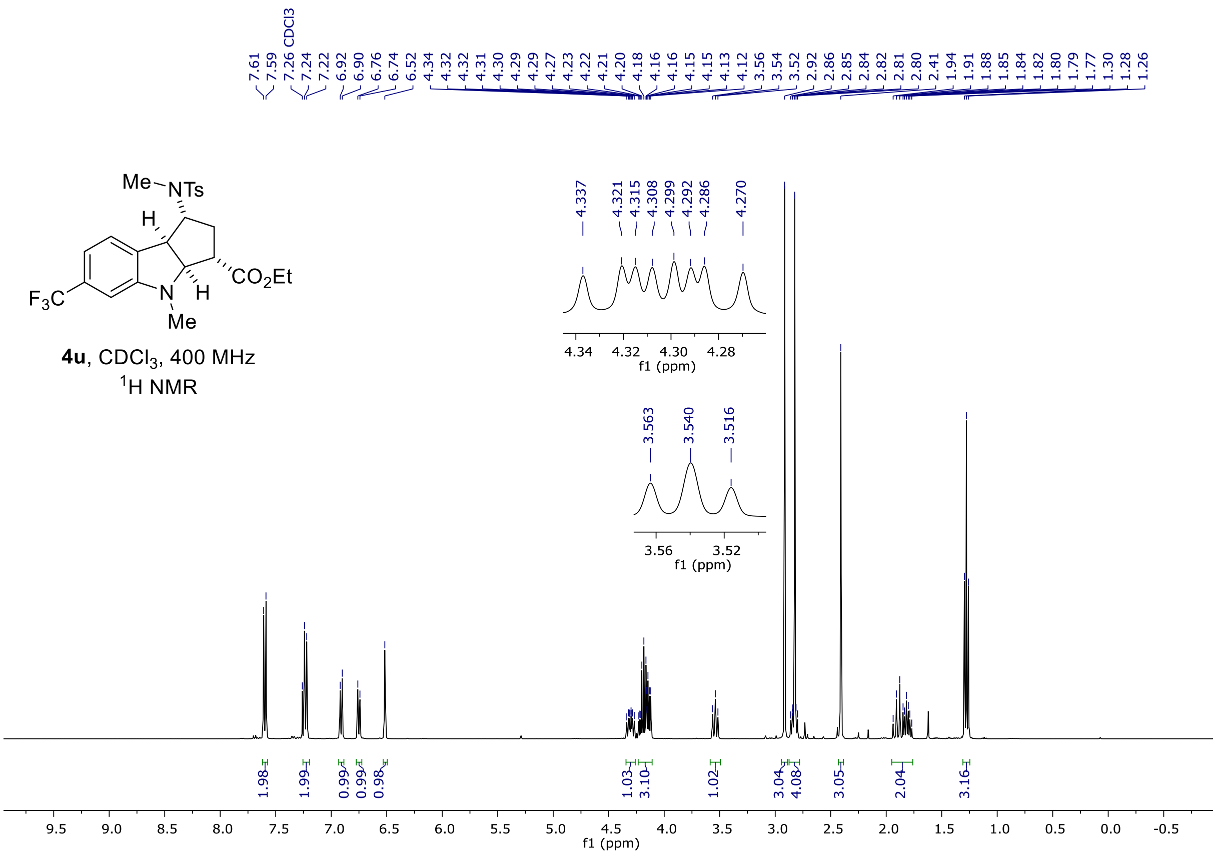
7.61
7.59
7.26 CDCl3
7.24
7.22
6.92
6.90
6.76
6.74
6.52
4.34
4.32
4.31
4.30
4.29
4.29
4.27
4.23
4.22
4.21
4.20
4.18
4.16
4.16
4.15
4.15
4.13
4.12
3.56
3.54
3.52
2.92
2.86
2.85
2.84
2.82
2.81
2.80
2.41
1.94
1.91
1.88
1.85
1.84
1.82
1.80
1.79
1.77
1.30
1.28
1.26
Me
NTs
H
CO2Et
F3C
N
H
Me
4u, CDCl3, 400 MHz
1H NMR
4.337
4.321
4.315
4.308
4.299
4.292
4.286
4.270
4.34
4.32
4.30
4.28
f1 (ppm)
3.563
3.540
3.516
3.56
3.52
f1 (ppm)
1.98
1.99
0.99
0.99
0.98
1.03
3.10
1.02
3.04
4.08
3.05
2.04
3.16
9.5
9.0
8.5
8.0
7.5
7.0
6.5
6.0
5.5
5.0
4.5
4.0
3.5
3.0
2.5
2.0
1.5
1.0
0.5
0.0
-0.5
f1 (ppm)

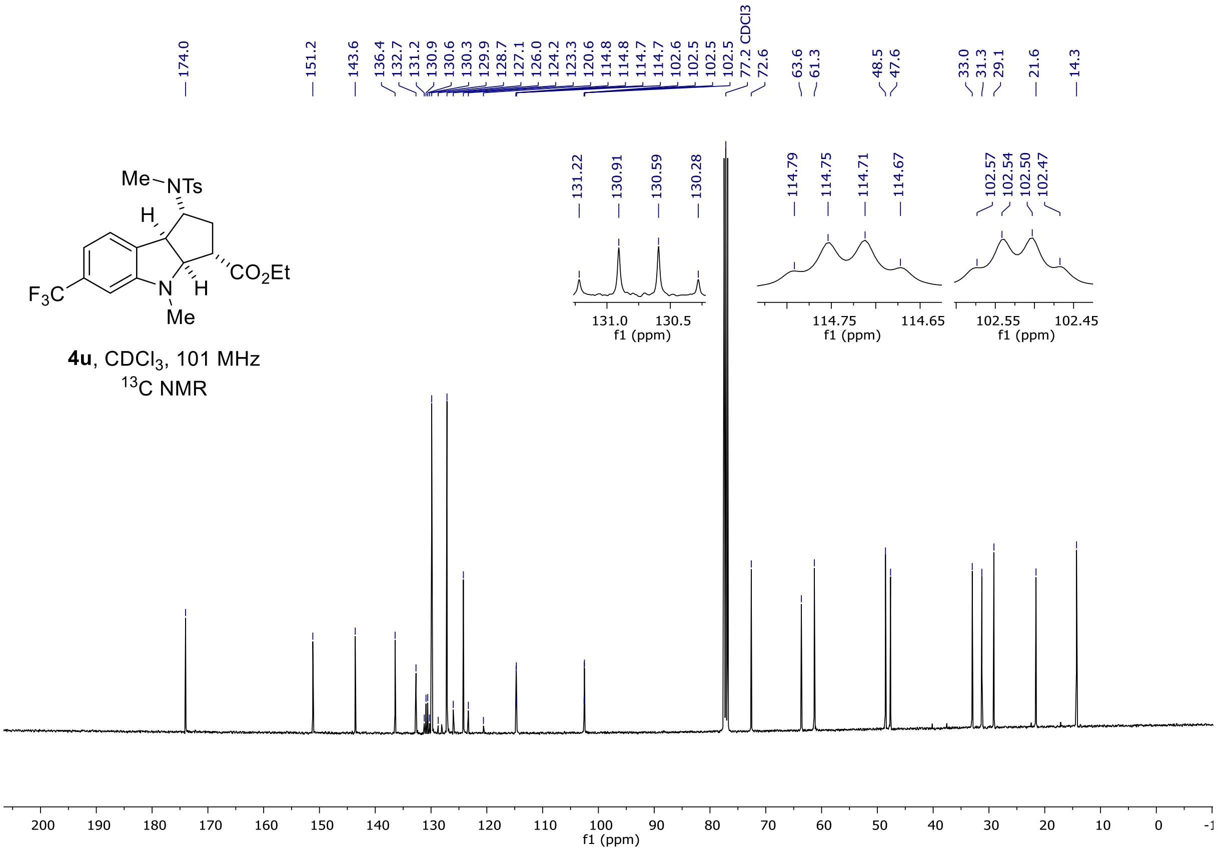
174.0
151.2
143.6
136.4
132.7
131.2
130.9
130.6
130.3
129.9
128.7
127.1
126.0
124.2
123.3
120.6
114.8
114.8
114.7
114.7
102.6
102.5
102.5
102.5
77.2 CDCl3
72.6
63.6
61.3
48.5
47.6
33.0
31.3
29.1
21.6
14.3
Me
NTs
H
CO2Et
F3C
N
H
Me
4u, CDCl3, 101 MHz
13C NMR
131.22
130.91
130.59
130.28
131.0
130.5
f1 (ppm)
114.79
114.75
114.71
114.67
114.75
114.65
f1 (ppm)
102.57
102.54
102.50
102.47
102.55
102.45
f1 (ppm)
200
190
180
170
160
150
140
130
120
110
100
90
80
70
60
50
40
30
20
10
0
f1 (ppm)

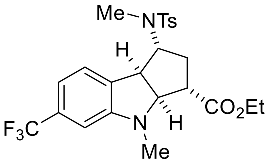

4u, $CDCl_3$, 376 MHz
^{19}F NMR

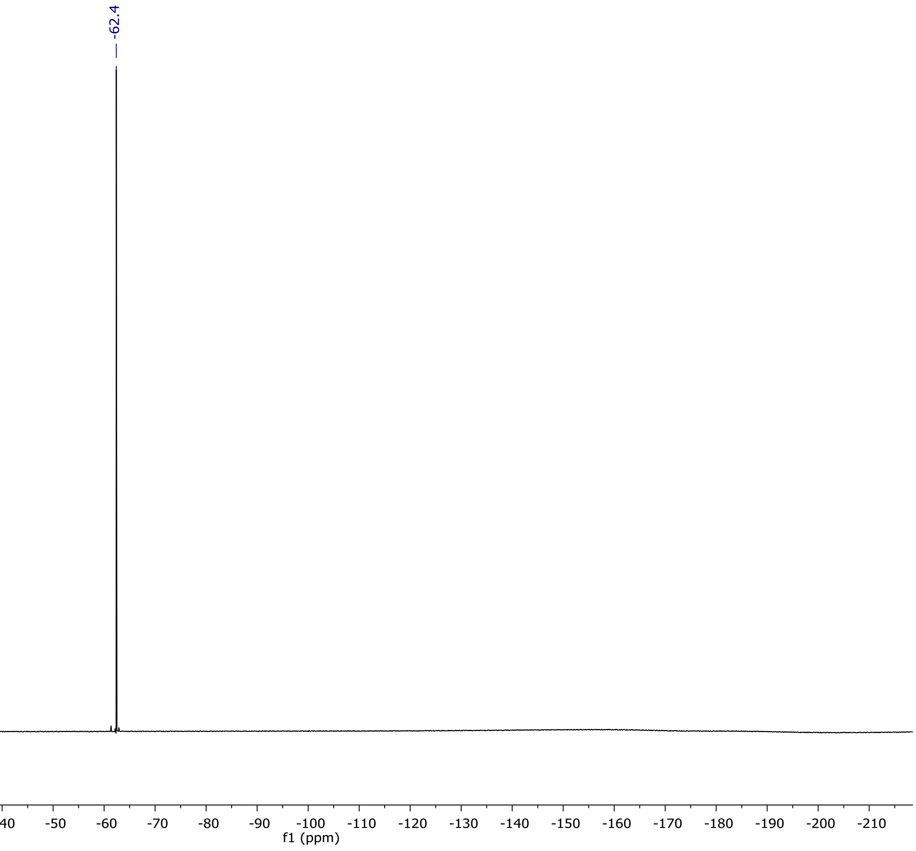

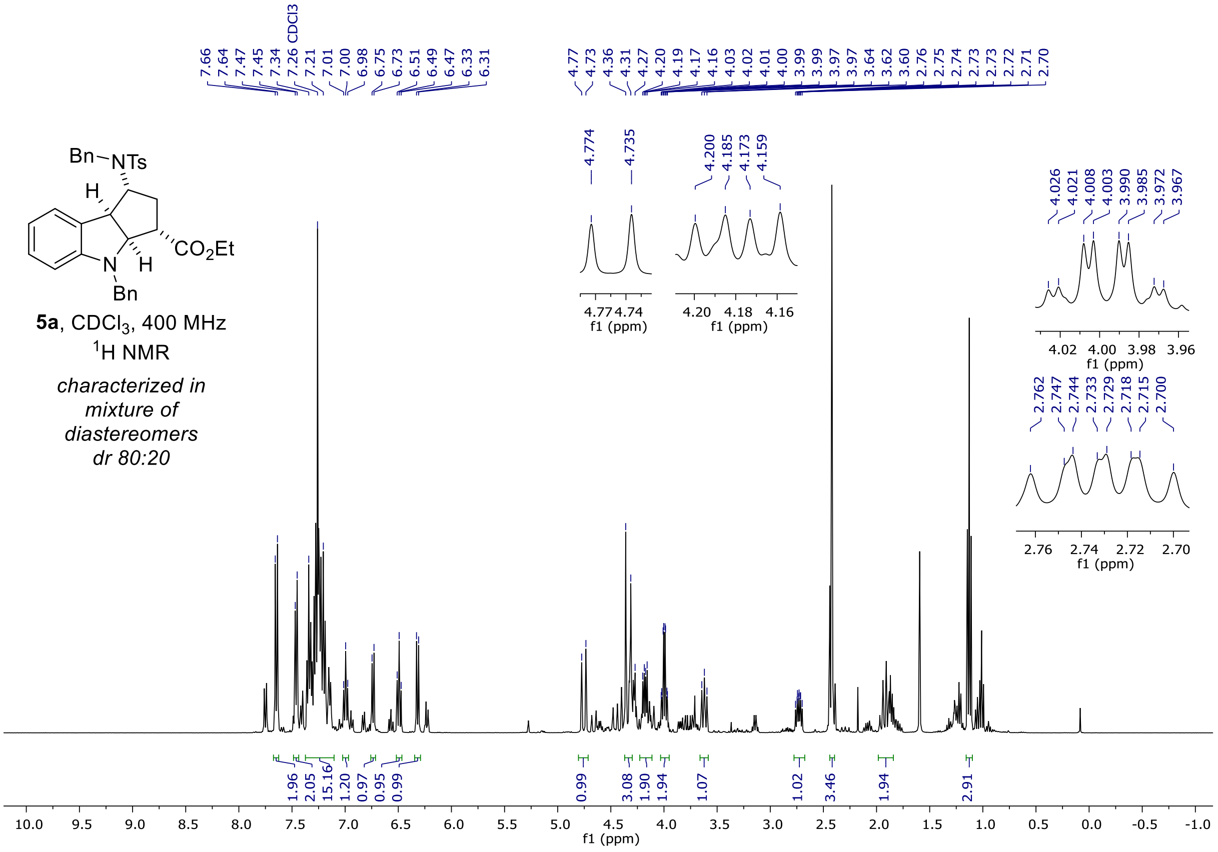

5a, $CDCl_3$, 400 MHz
^{1}H NMR
characterized in mixture of diastereomers dr 80:20

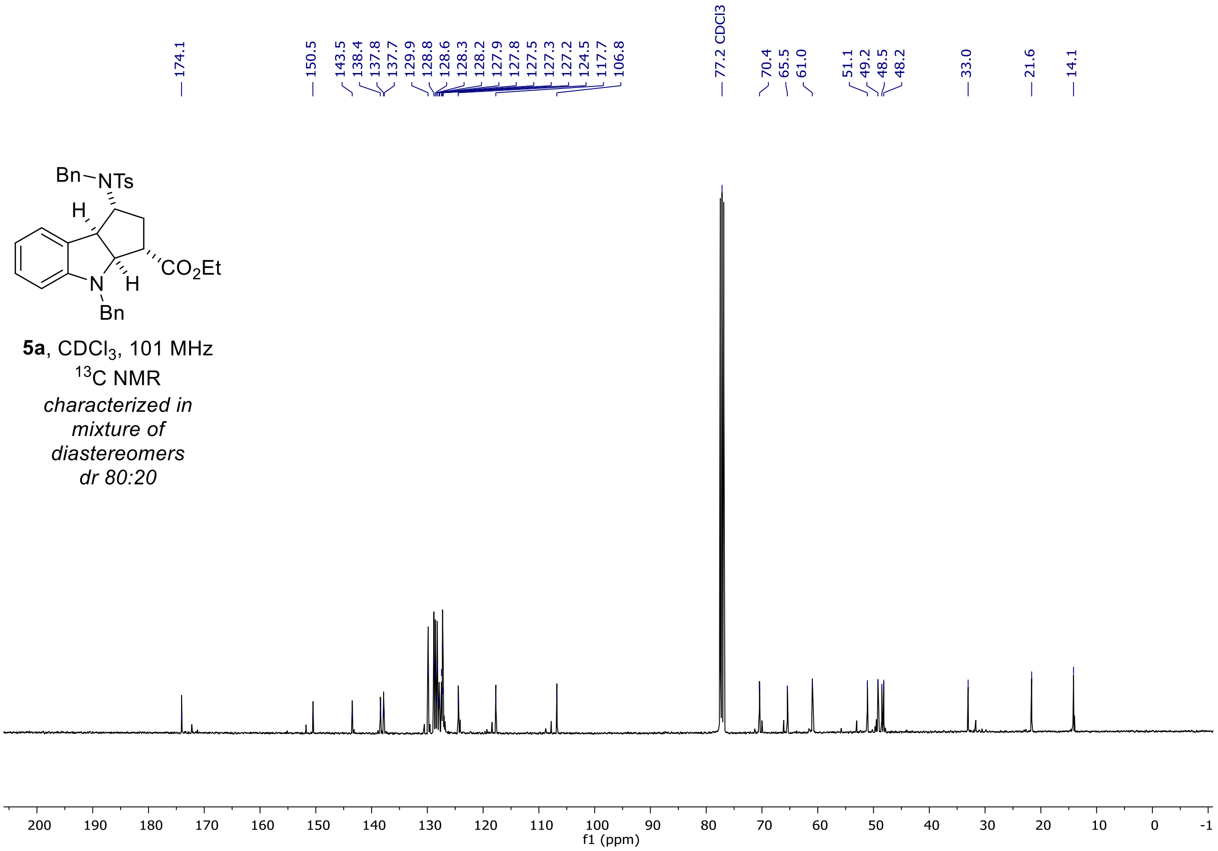

5a, $CDCl_3$, 101 MHz
^{13}C NMR
characterized in mixture of diastereomers dr 80:20

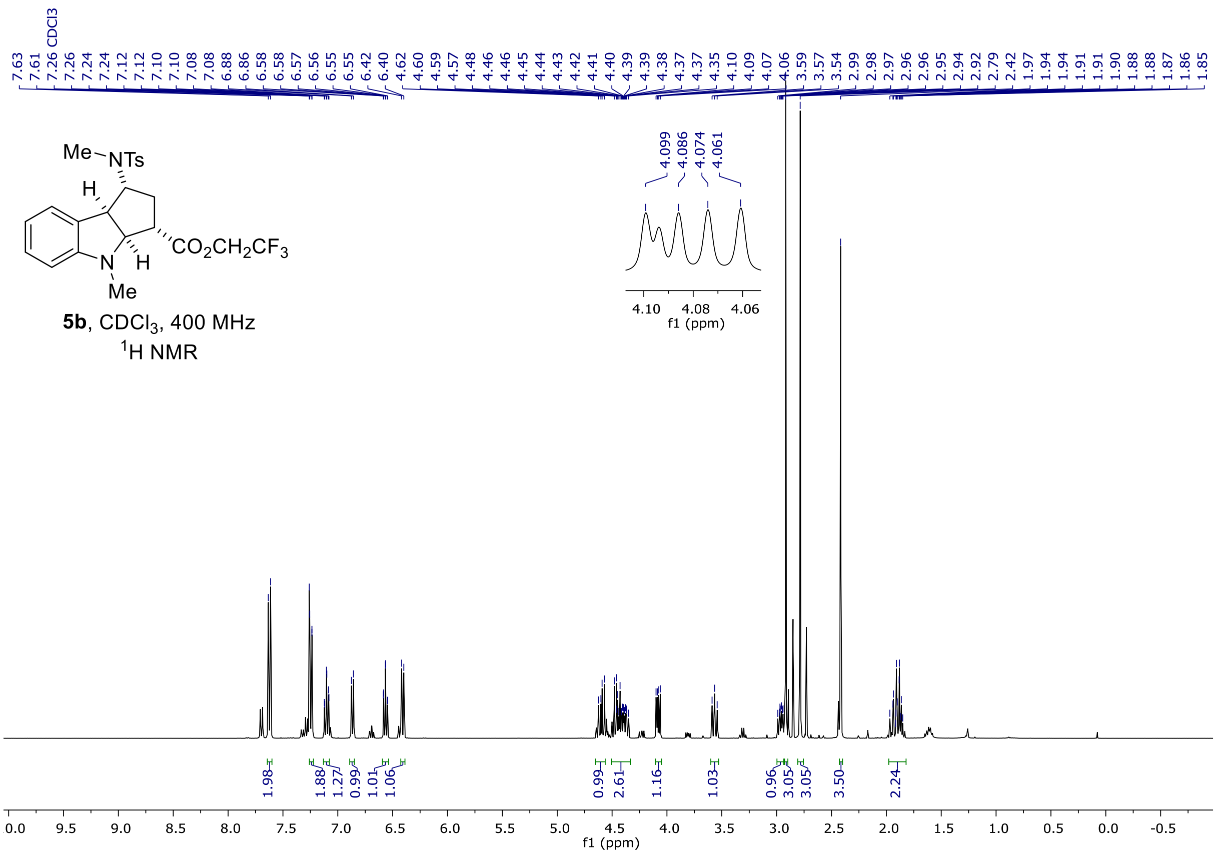

Me
NTs
H
CO2CH2CF3
N
H
Me
5b, CDCl3, 400 MHz
1H NMR
f1 (ppm)

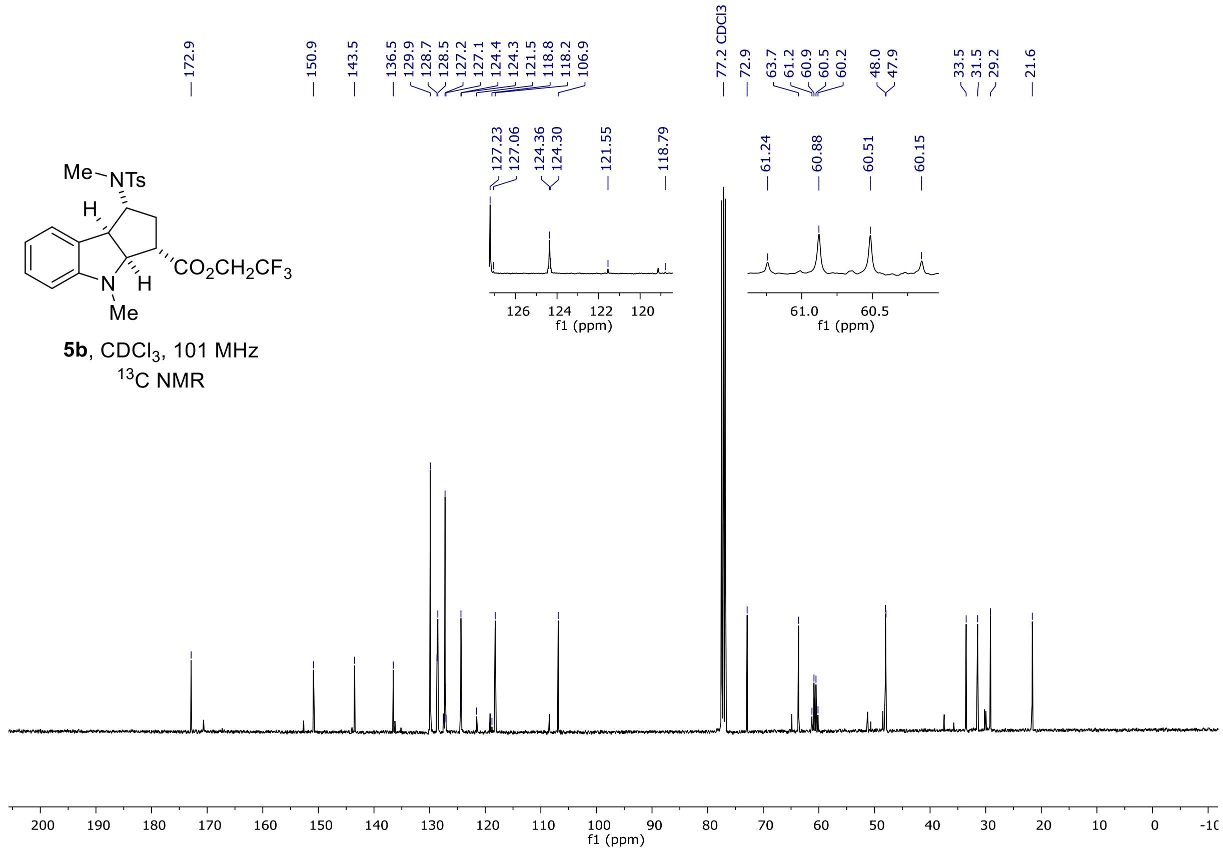

Me
NTs
H
CO2CH2CF3
N
H
Me
5b, CDCl3, 101 MHz
13C NMR
f1 (ppm)

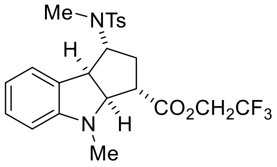

5b, $CDCl_3$, 376 MHz
^{19}F NMR

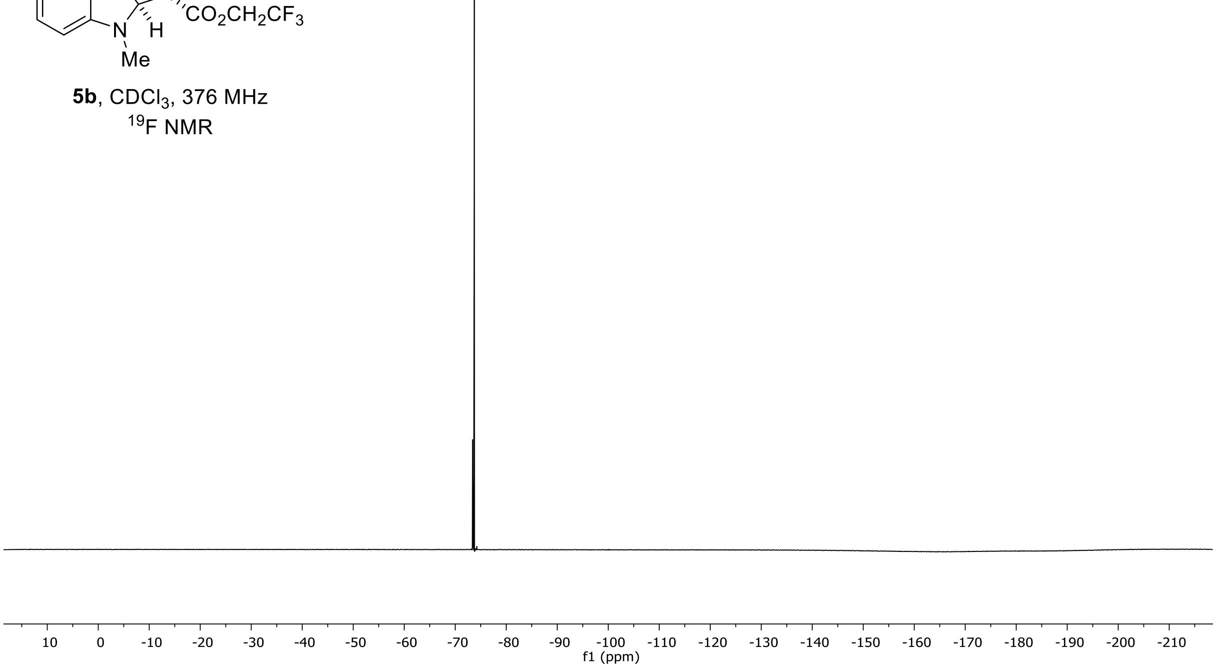

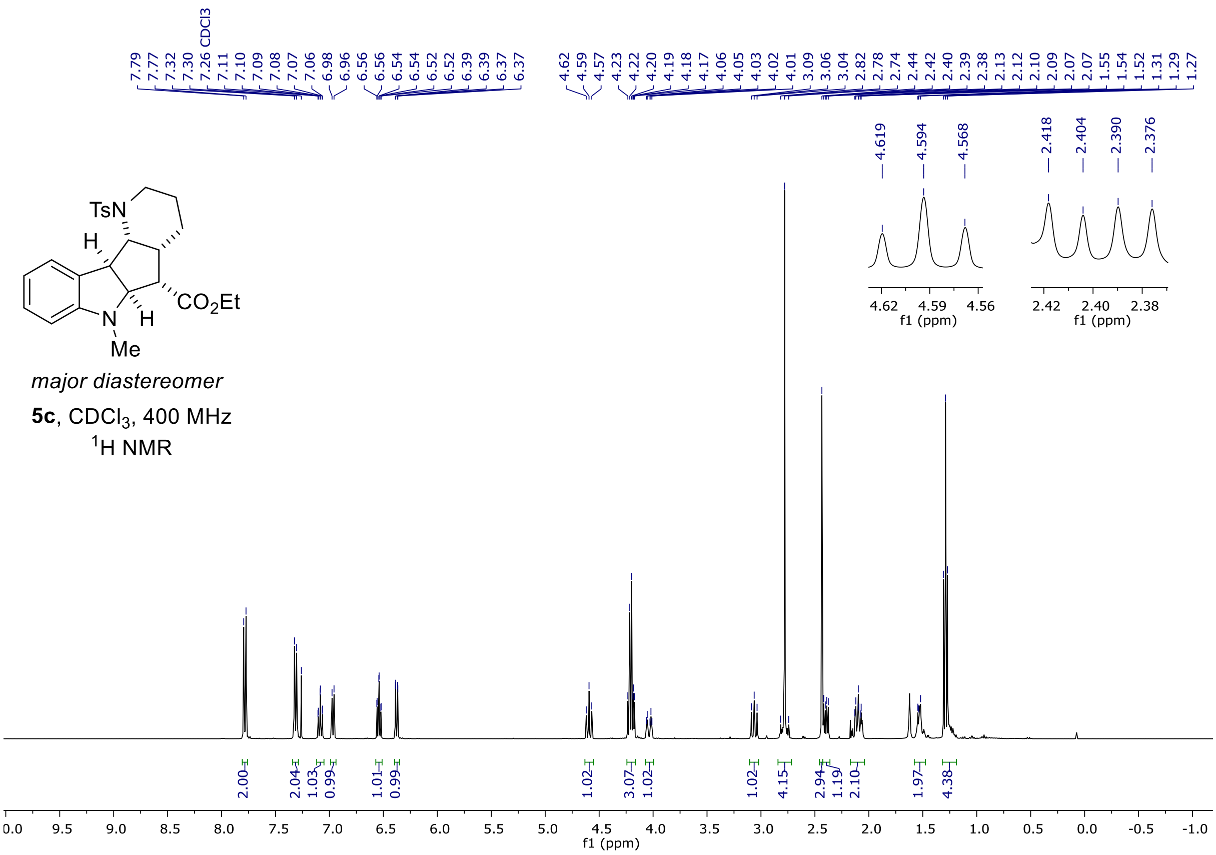
TsN
H
CO2Et
N
H
Me
major diastereomer
5c, CDCl3, 400 MHz
1H NMR
7.79 7.77 7.32 7.30 7.26 CDCl3 7.11 7.10 7.09 7.08 7.07 7.06 6.98 6.96 6.56 6.56 6.54 6.54 6.52 6.52 6.39 6.39 6.37 6.37
4.62 4.59 4.57 4.23 4.22 4.20 4.19 4.18 4.17 4.06 4.05 4.03 4.02 4.01 3.09 3.06 3.04 2.82 2.78 2.74 2.44 2.42 2.40 2.39 2.38 2.13 2.12 2.10 2.09 2.07 1.55 1.54 1.52 1.31 1.29 1.27
4.619 4.594 4.568
2.418 2.404 2.390 2.376
4.62 4.59 4.56
f1 (ppm)
2.42 2.40 2.38
f1 (ppm)
2.00 2.04 1.03 0.99 1.01 0.99 1.02 3.07 1.02 1.02 4.15 2.94 1.19 2.10 1.97 4.38
f1 (ppm)

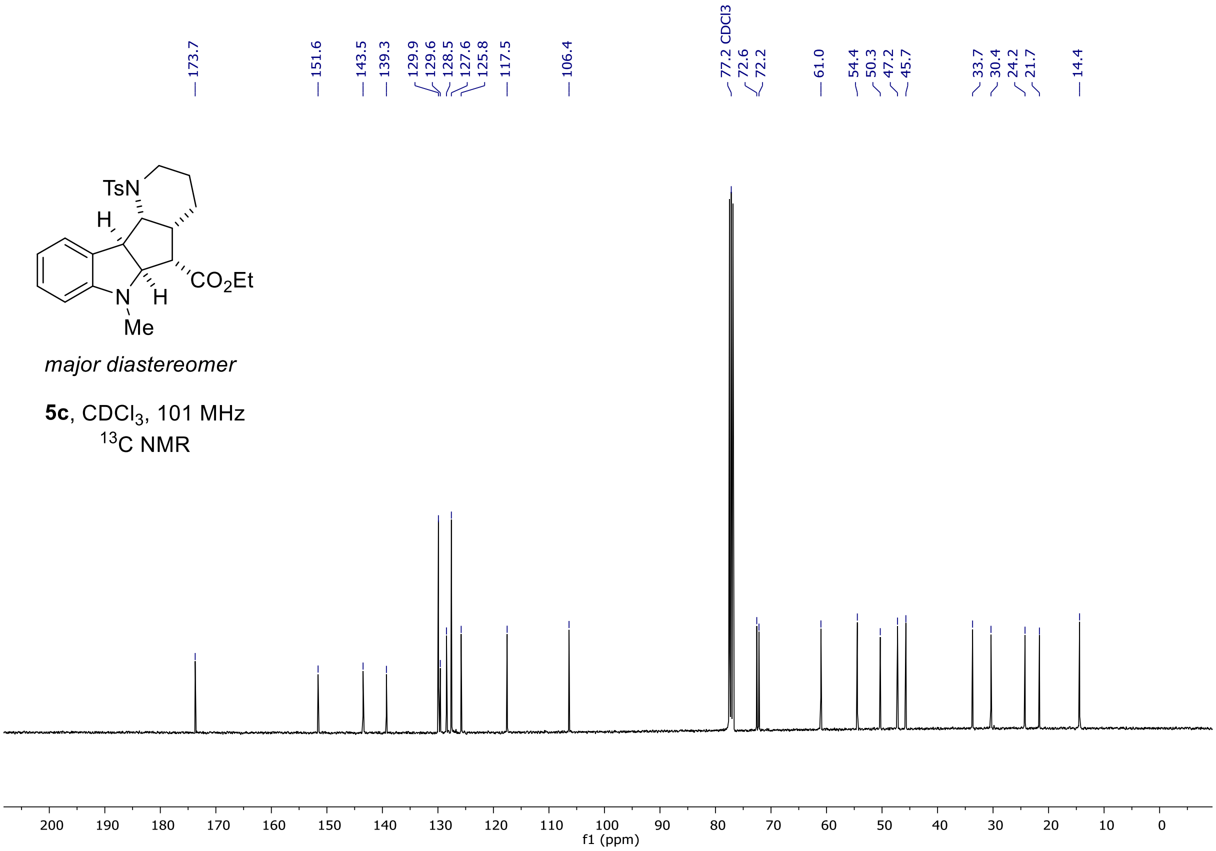
173.7 151.6 143.5 139.3 129.9 129.6 128.5 127.6 125.8 117.5 106.4
77.2 CDCl3 72.6 72.2 61.0 54.4 50.3 47.2 45.7 33.7 30.4 24.2 21.7 14.4
TsN
H
CO2Et
N
H
Me
major diastereomer
5c, CDCl3, 101 MHz
13C NMR
f1 (ppm)

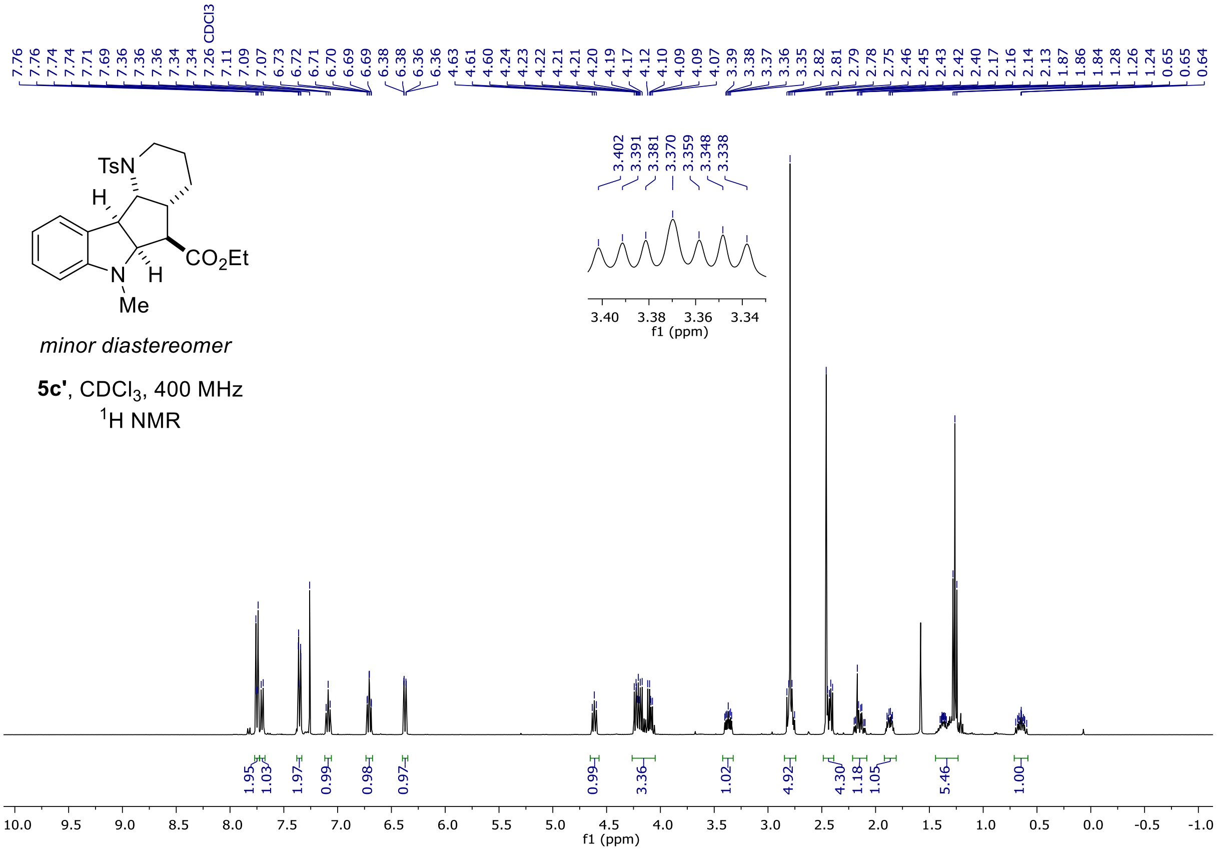

minor diastereomer

5c', $CDCl_3$, 400 MHz

^1H NMR

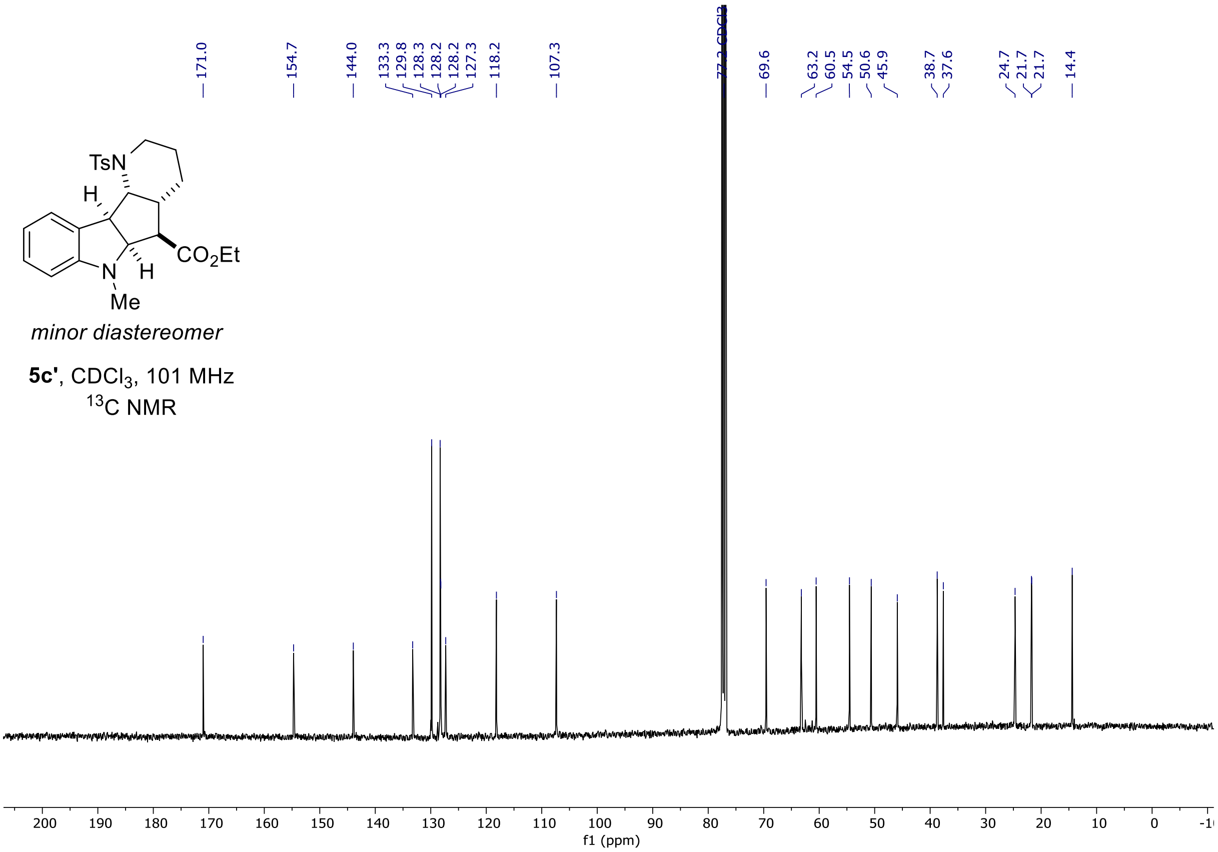

minor diastereomer

5c', $CDCl_3$, 101 MHz

^{13}C NMR

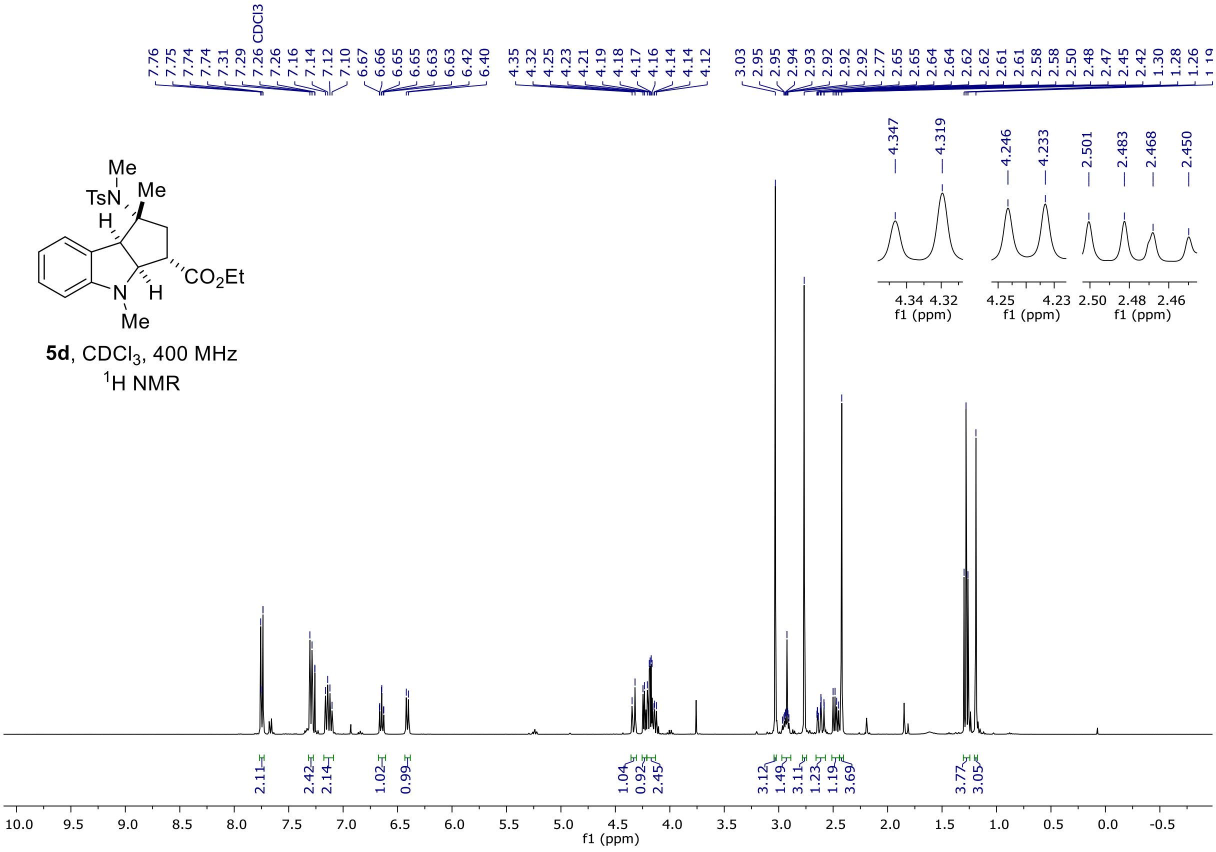
7.76 7.75 7.74 7.74 7.31 7.29 7.26 CDCl3 7.26 7.16 7.14 7.12 7.10 6.67 6.66 6.65 6.63 6.63 6.42 6.40 4.35 4.32 4.25 4.23 4.21 4.19 4.18 4.17 4.16 4.14 4.14 4.12 3.03 2.95 2.95 2.94 2.93 2.92 2.92 2.92 2.77 2.65 2.65 2.64 2.64 2.62 2.62 2.61 2.61 2.58 2.58 2.50 2.48 2.47 2.45 2.42 1.30 1.28 1.26
4.347 4.319 4.246 4.233 2.501 2.483 2.468 2.450
4.34 4.32 f1 (ppm)
4.25 4.23 f1 (ppm)
2.50 2.48 2.46 f1 (ppm)
Me
TsN
Me
H
CO2Et
N
H
Me
5d, CDCl3, 400 MHz
1H NMR
2.11 2.42 2.14 1.02 0.99 1.04 0.92 2.45 3.12 1.49 3.11 1.23 1.19 3.69 3.77 3.05
10.0 9.5 9.0 8.5 8.0 7.5 7.0 6.5 6.0 5.5 5.0 4.5 4.0 3.5 3.0 2.5 2.0 1.5 1.0 0.5 0.0 -0.5
f1 (ppm)

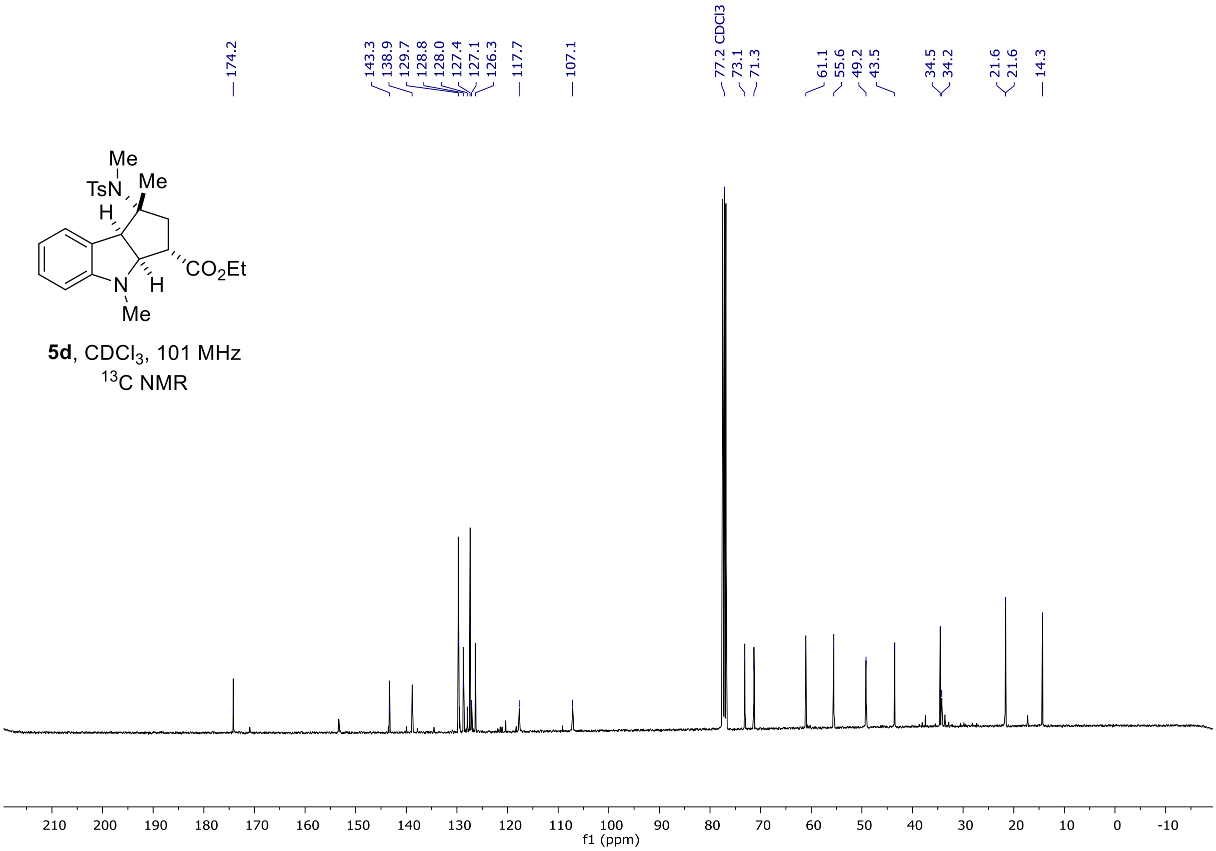
174.2 143.3 138.9 129.7 128.8 128.0 127.4 127.1 126.3 117.7 107.1 77.2 CDCl3 73.1 71.3 61.1 55.6 49.2 43.5 34.5 34.2 21.6 21.6 14.3
Me
TsN
Me
H
CO2Et
N
H
Me
5d, CDCl3, 101 MHz
13C NMR
210 200 190 180 170 160 150 140 130 120 110 100 90 80 70 60 50 40 30 20 10 0 -10
f1 (ppm)

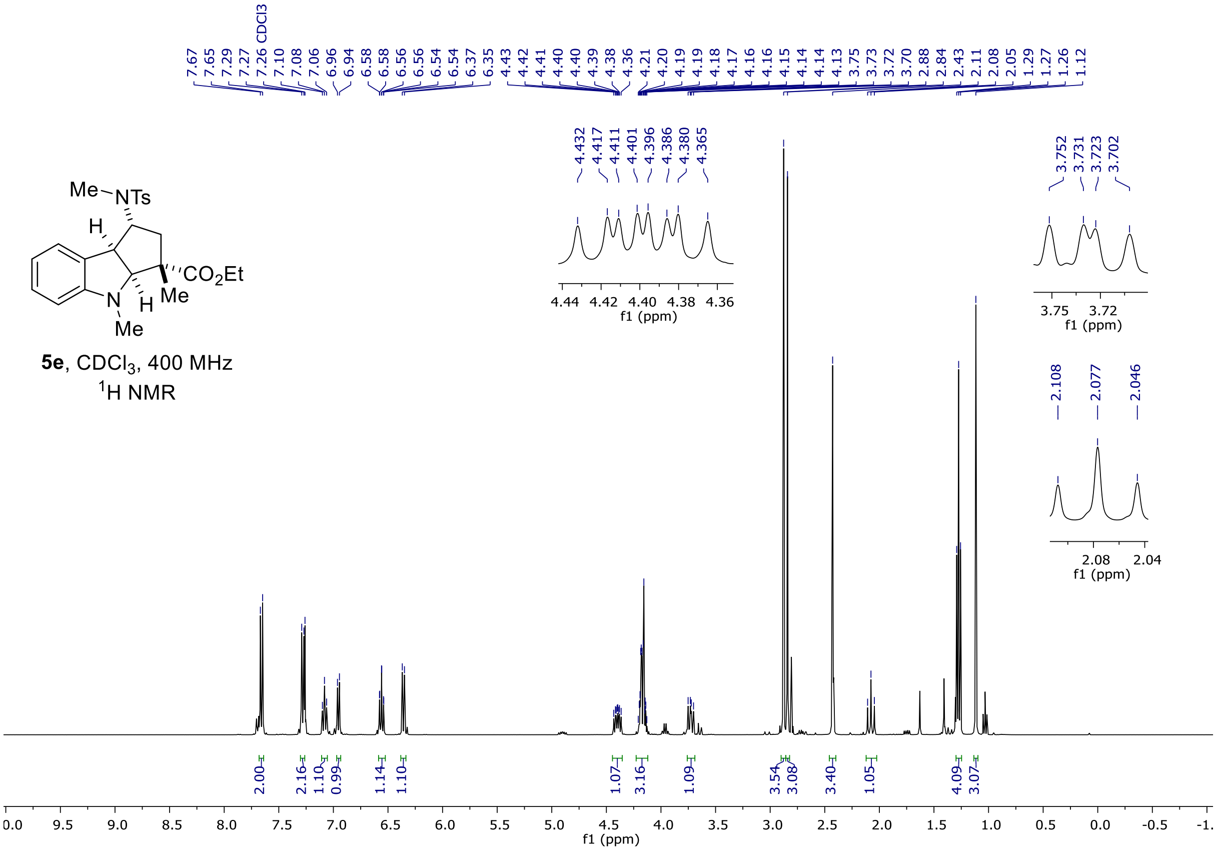
Me
NTs
H
CO2Et
N
H
Me
Me
5e, CDCl3, 400 MHz
1H NMR
7.67 7.65 7.29 7.27 7.26 CDCl3 7.10 7.08 7.06 6.96 6.94 6.58 6.58 6.56 6.56 6.54 6.54 6.37 6.35 4.43 4.41 4.40 4.39 4.38 4.36 4.21 4.20 4.19 4.19 4.18 4.17 4.16 4.15 4.14 4.14 4.13 3.75 3.73 3.72 3.70 2.88 2.84 2.43 2.11 2.08 2.05 1.29 1.27 1.26 1.12
4.432 4.417 4.411 4.401 4.396 4.386 4.380 4.365
3.752 3.731 3.723 3.702
2.108 2.077 2.046
2.00 2.16 1.10 0.99 1.14 1.10 1.07 3.16 1.09 3.54 3.08 3.40 1.05 4.09 3.07
f1 (ppm)

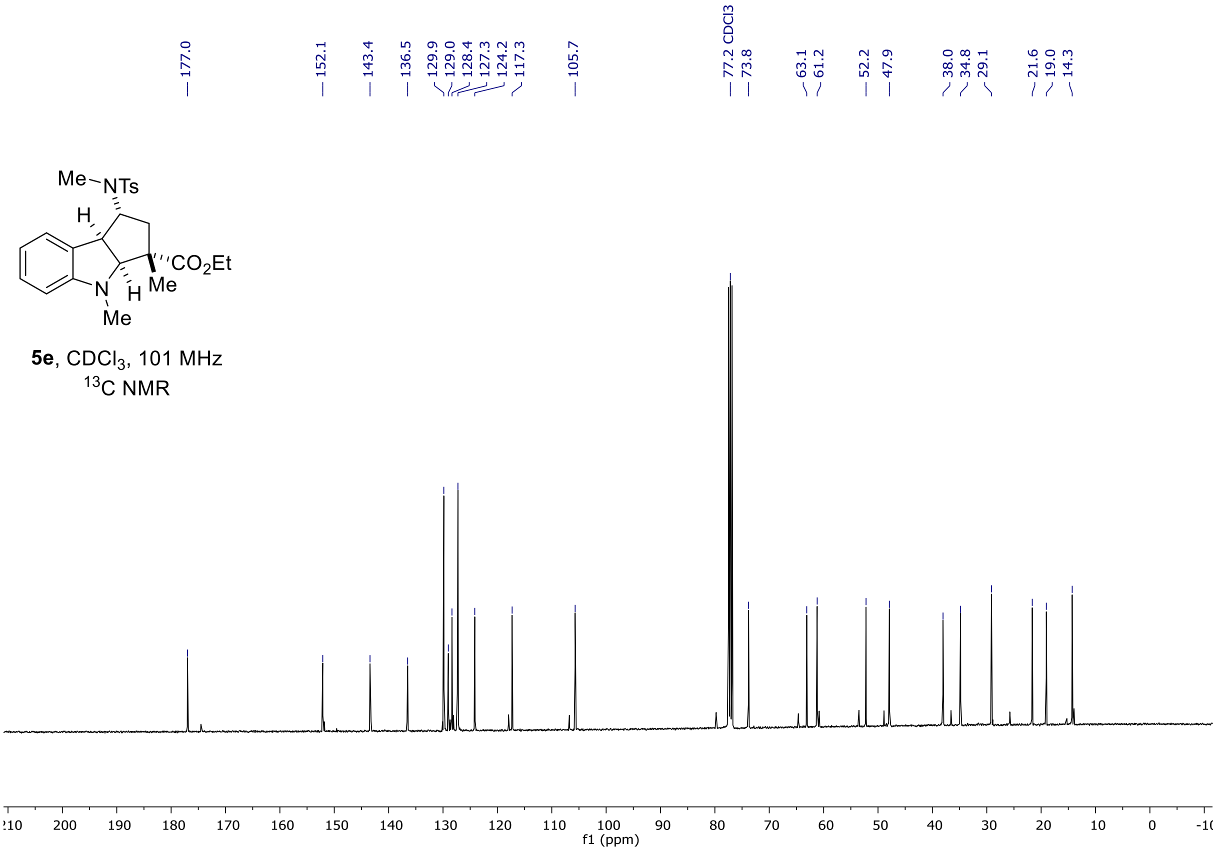
177.0 152.1 143.4 136.5 129.9 129.0 128.4 127.3 124.2 117.3 105.7 77.2 CDCl3 73.8 63.1 61.2 52.2 47.9 38.0 34.8 29.1 21.6 19.0 14.3
Me
NTs
H
CO2Et
N
H
Me
Me
5e, CDCl3, 101 MHz
13C NMR
f1 (ppm)

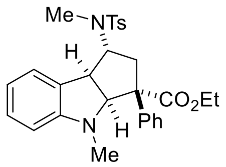

major diastereomer

5f, $CDCl_3$, 400 MHz

^{1}H NMR

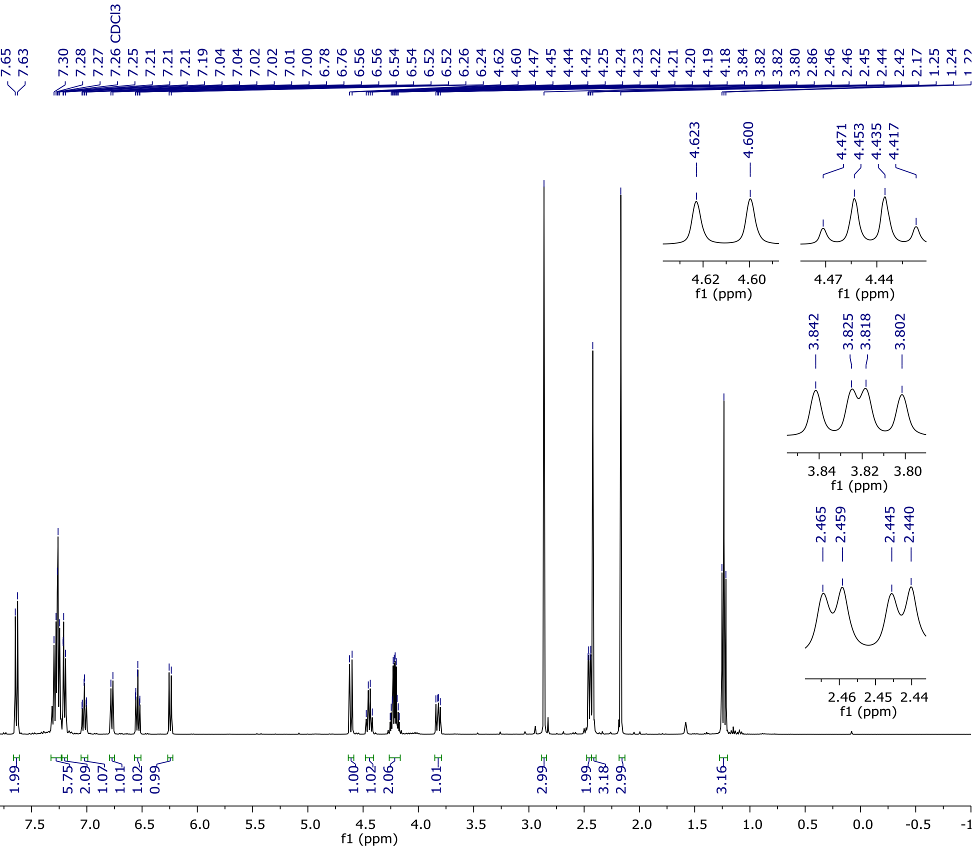

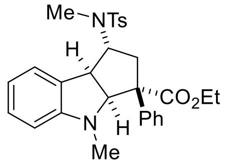

major diastereomer

5f, $CDCl_3$, 101 MHz

^{13}C NMR

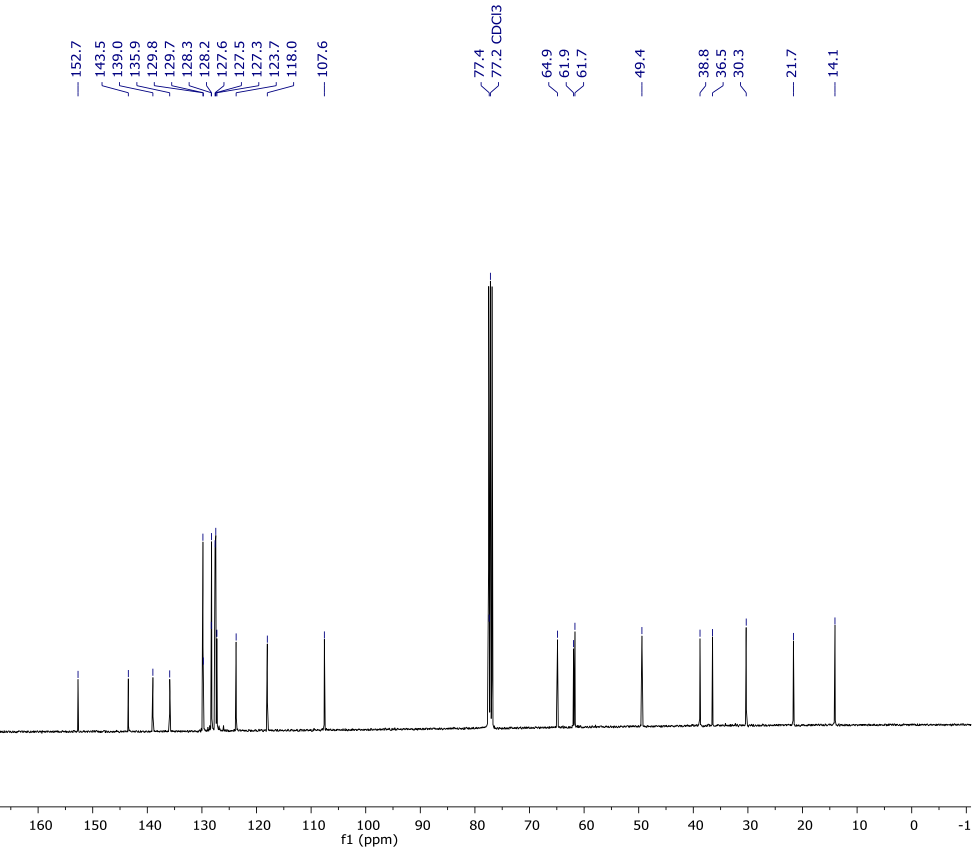

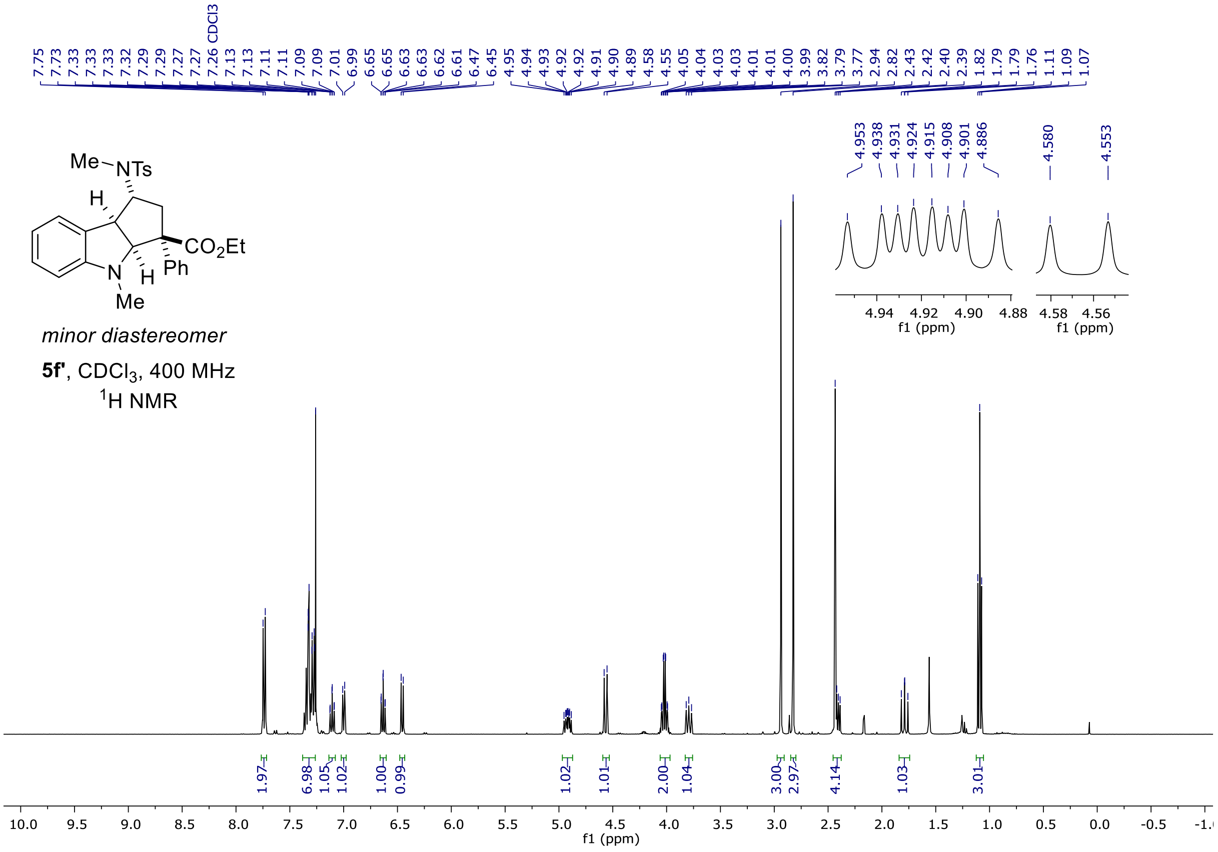
Me–NTs
H
CO2Et
N H Ph
Me
minor diastereomer
5f', CDCl3, 400 MHz
1H NMR
CDCl3
f1 (ppm)

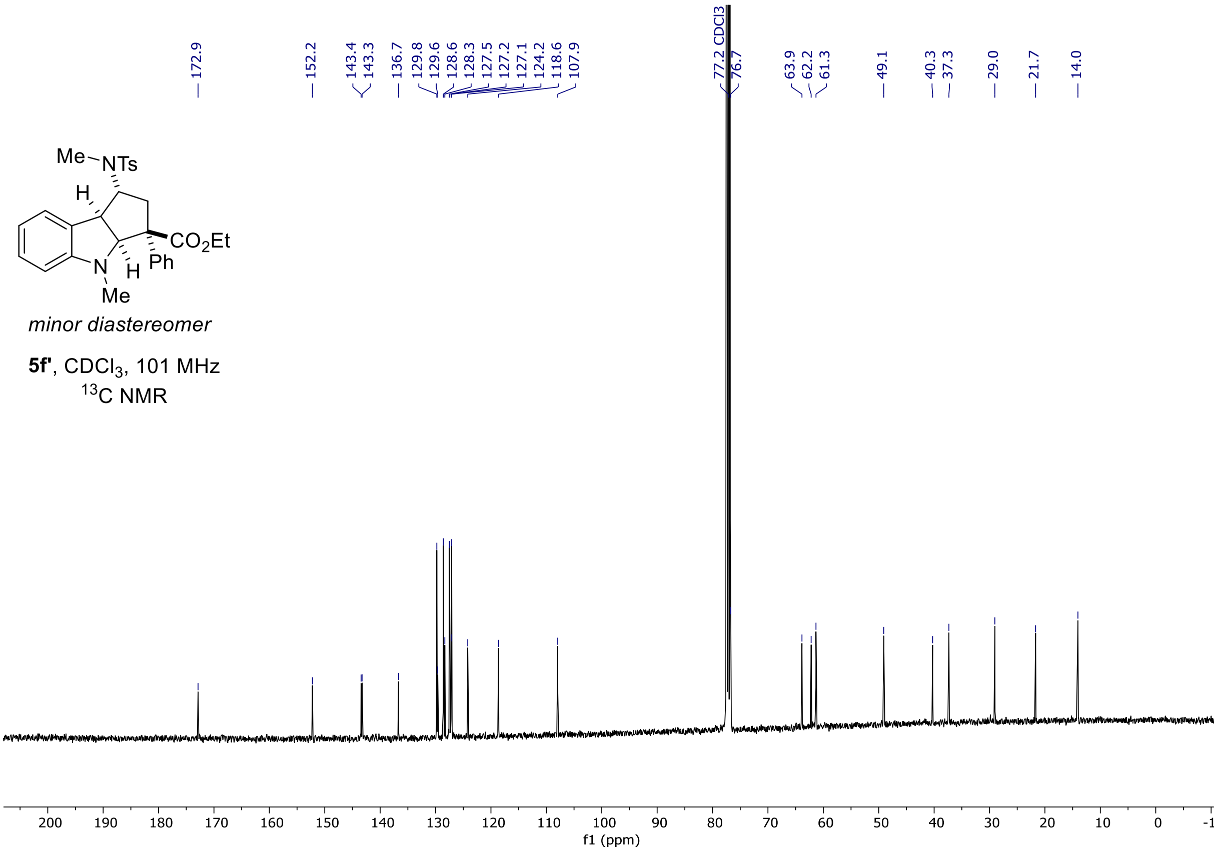
Me–NTs
H
CO2Et
N H Ph
Me
minor diastereomer
5f', CDCl3, 101 MHz
13C NMR
172.9
152.2
143.4
143.3
136.7
129.8
129.6
128.6
128.3
127.5
127.2
127.1
124.2
118.6
107.9
77.2 CDCl3
76.7
63.9
62.2
61.3
49.1
40.3
37.3
29.0
21.7
14.0
f1 (ppm)

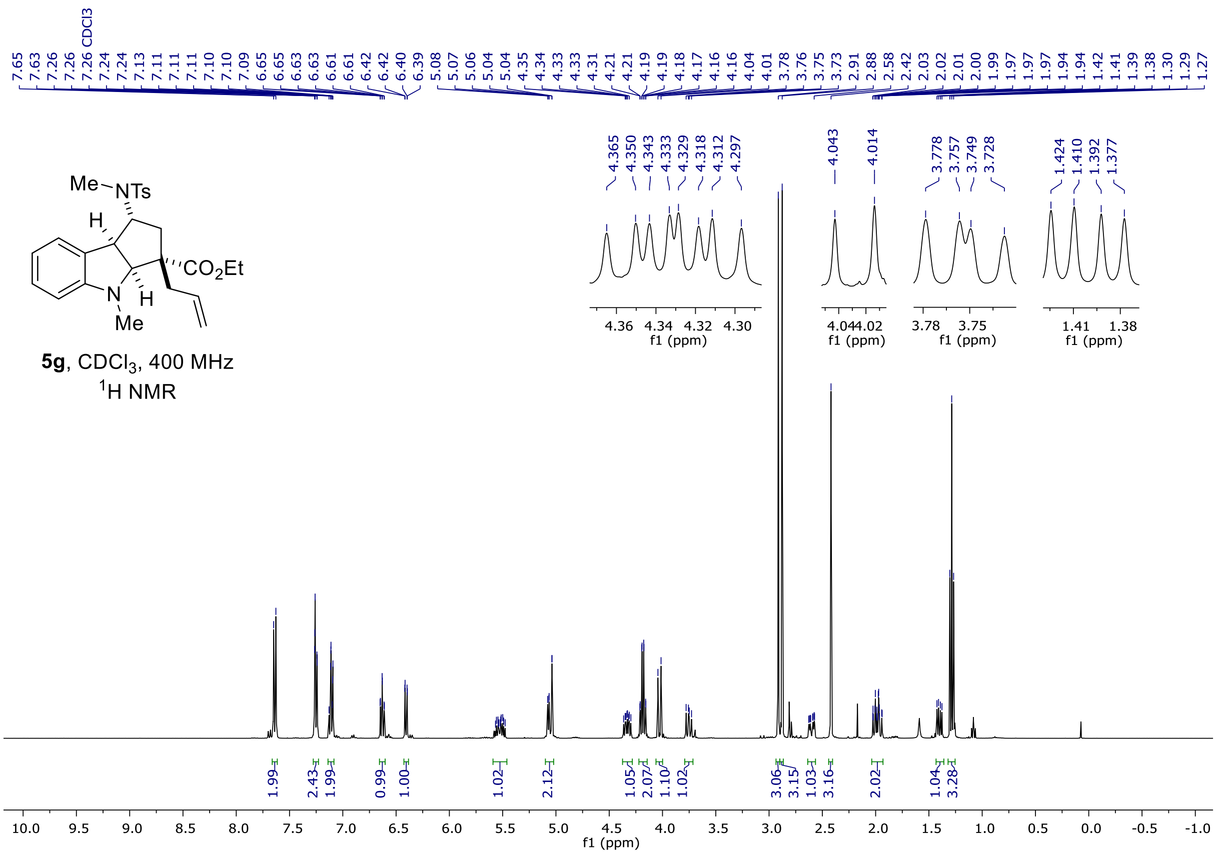

Me–NTs
H
CO2Et
N
H
Me
5g, CDCl3, 400 MHz
1H NMR
f1 (ppm)

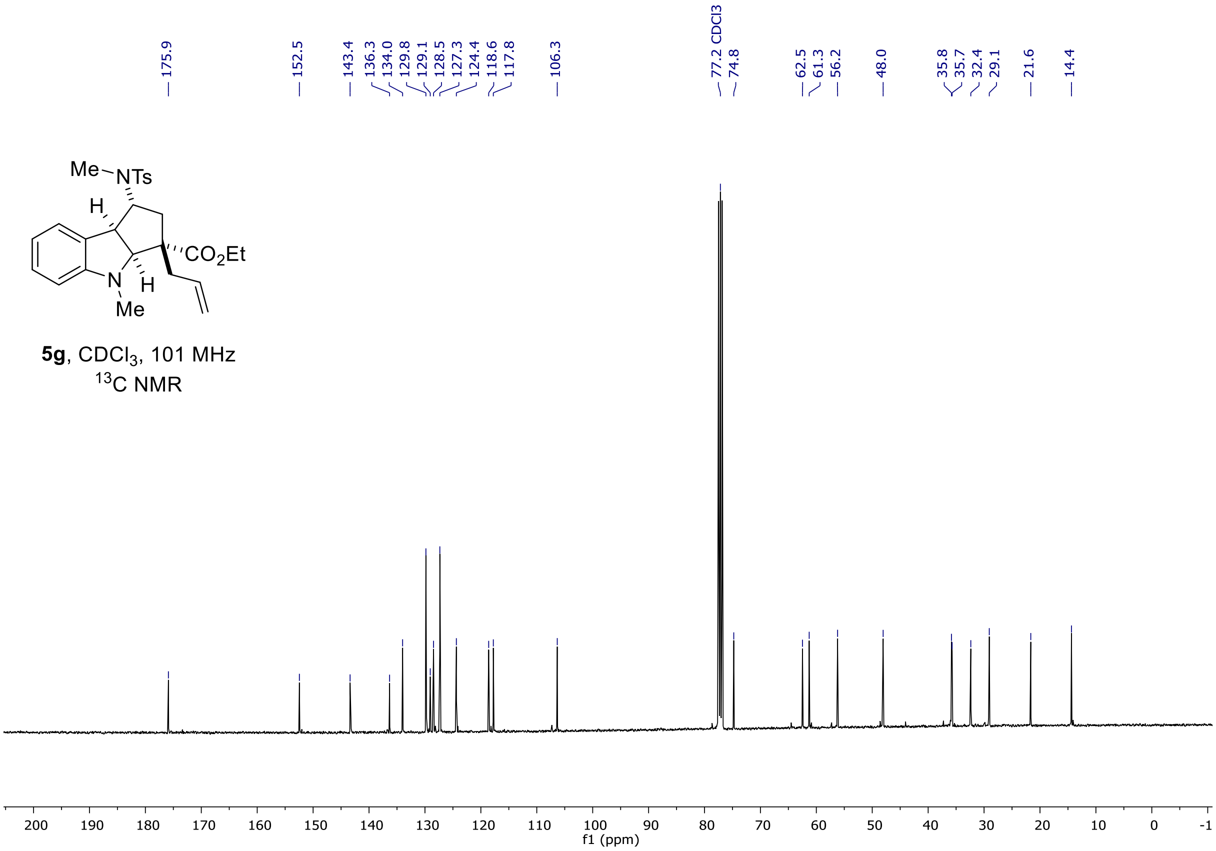

Me–NTs
H
CO2Et
N
H
Me
5g, CDCl3, 101 MHz
13C NMR
175.9
152.5
143.4
136.3
134.0
129.8
129.1
128.5
127.3
124.4
118.6
117.8
106.3
77.2 CDCl3
74.8
62.5
61.3
56.2
48.0
35.8
35.7
32.4
29.1
21.6
14.4
f1 (ppm)

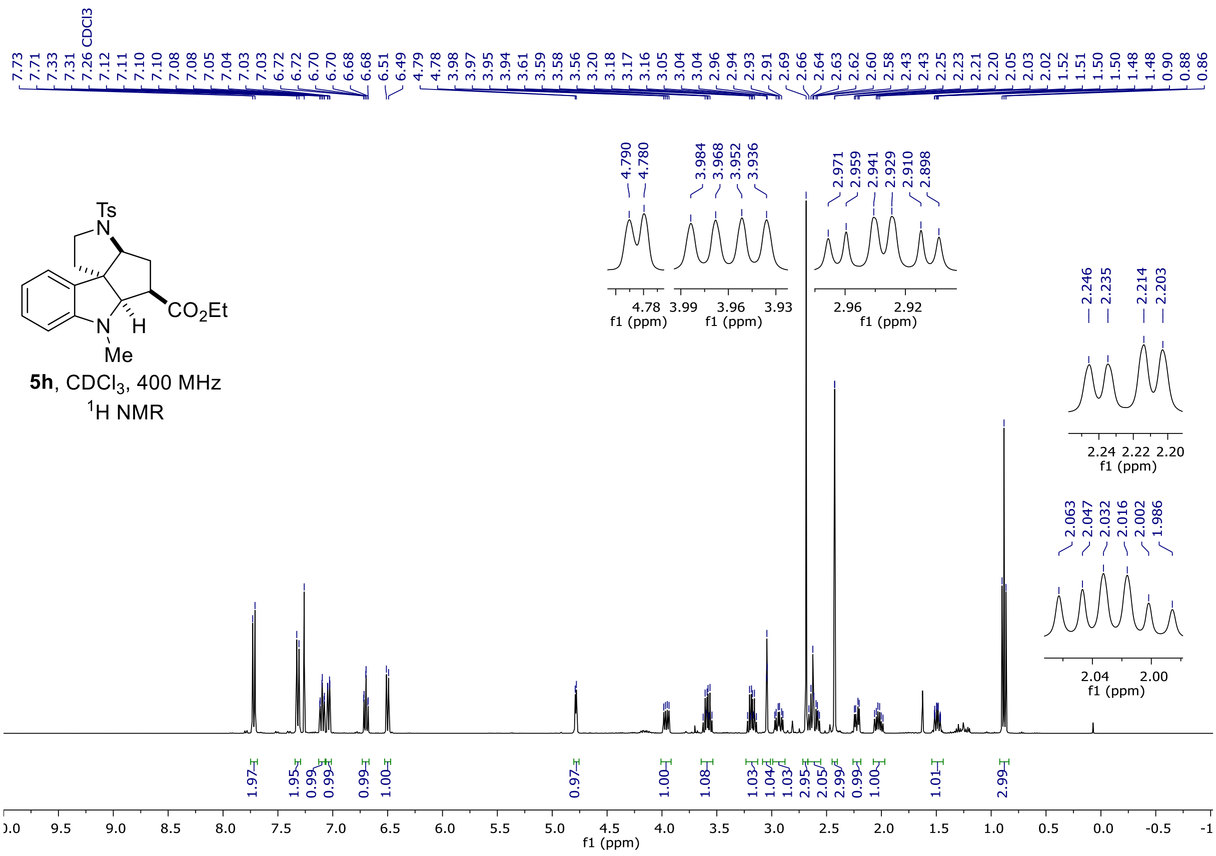

5h, $CDCl_3$, 400 MHz
^1H NMR

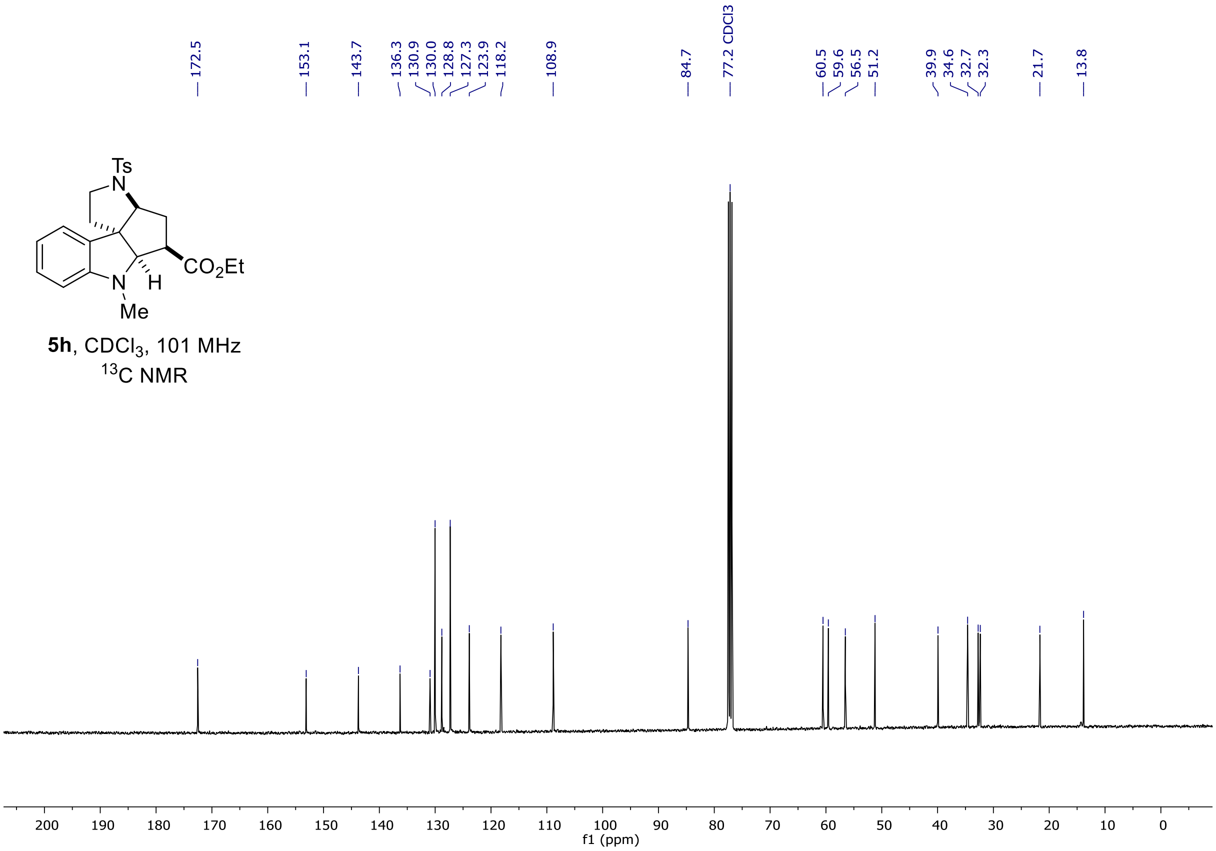

5h, $CDCl_3$, 101 MHz
^{13}C NMR

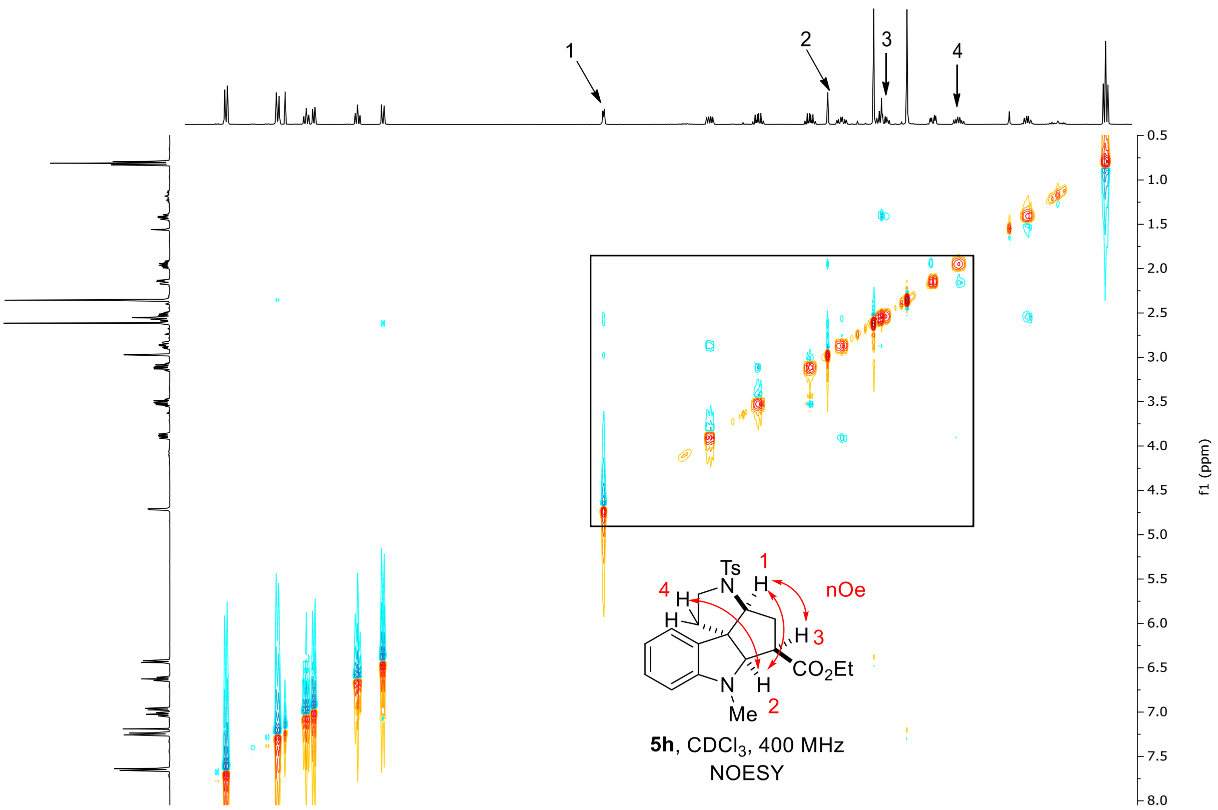

1
2
3
4
nOe
Ts
N
H
Me
CO2Et
5h, CDCl3, 400 MHz
NOESY
f2 (ppm)
f1 (ppm)

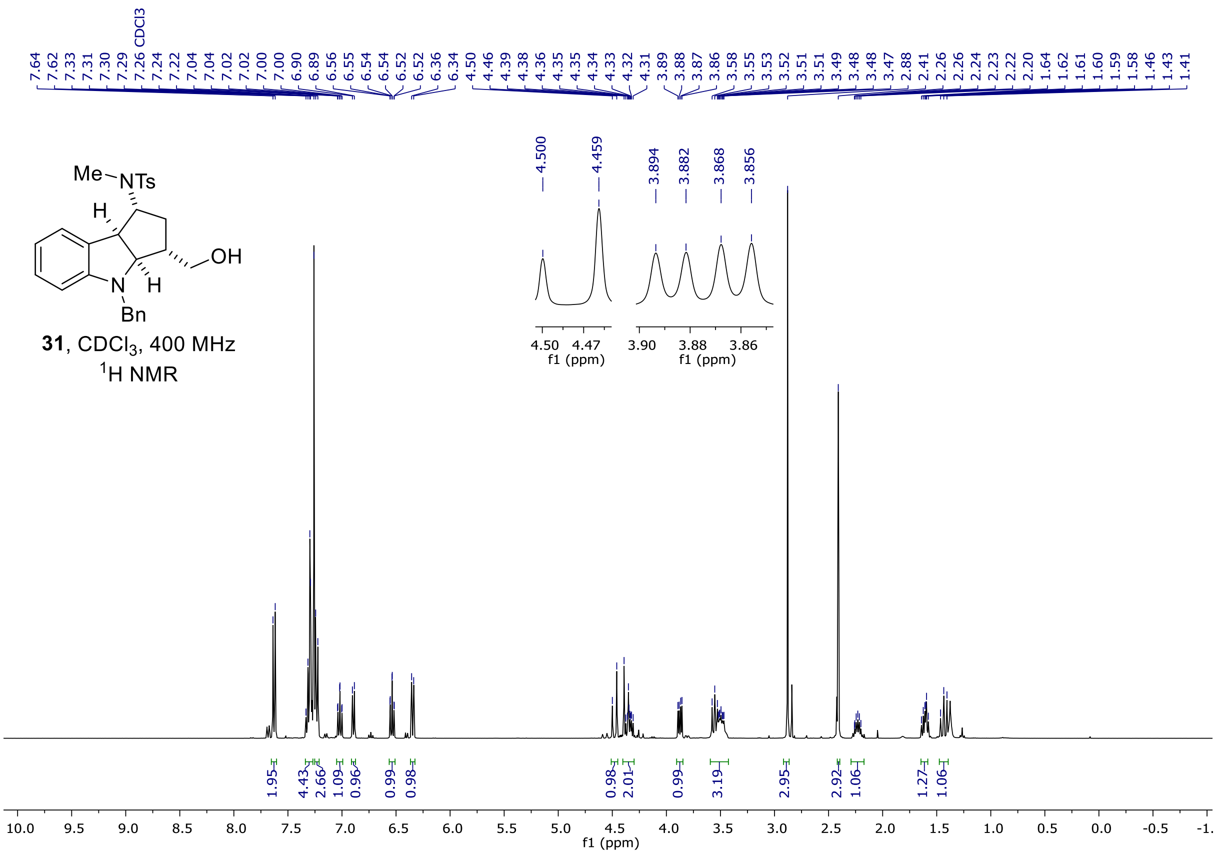

31, $CDCl_3$, 400 MHz

^1H NMR

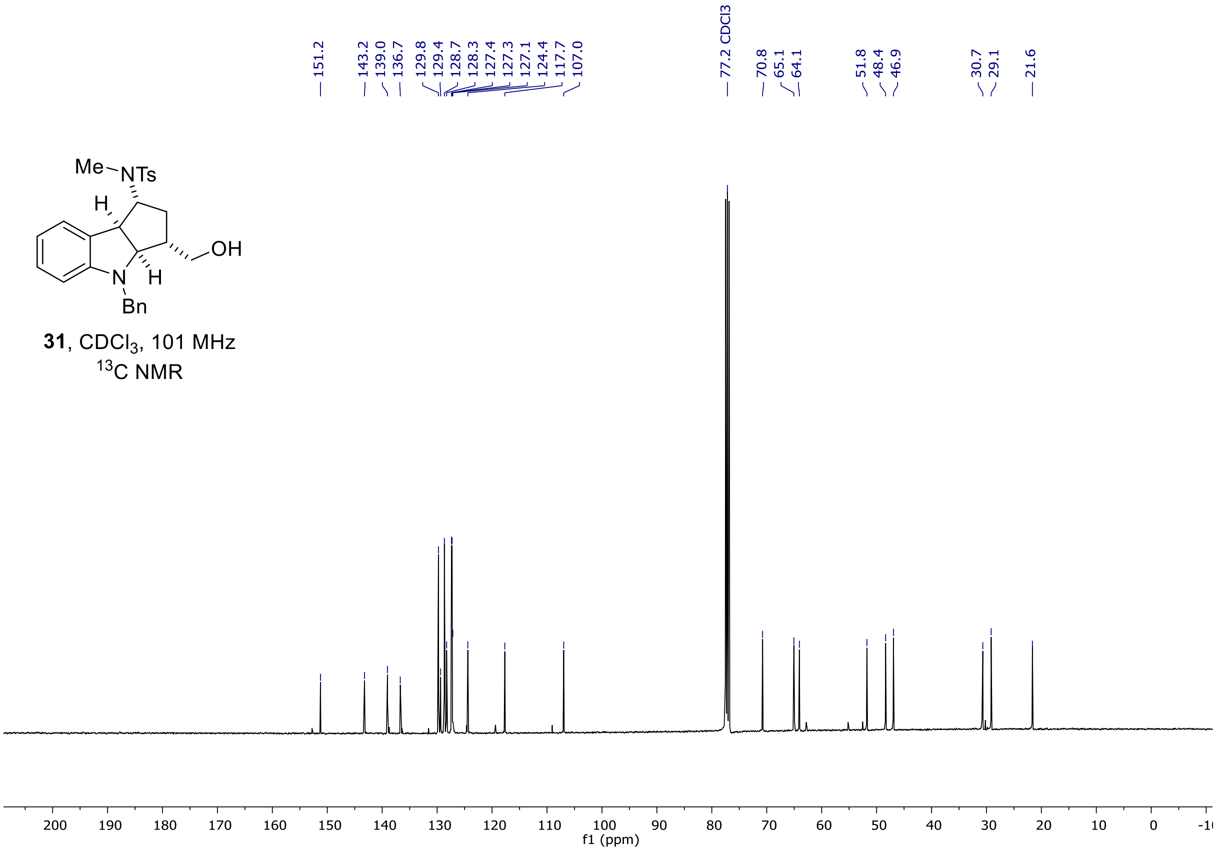

31, $CDCl_3$, 101 MHz

^{13}C NMR

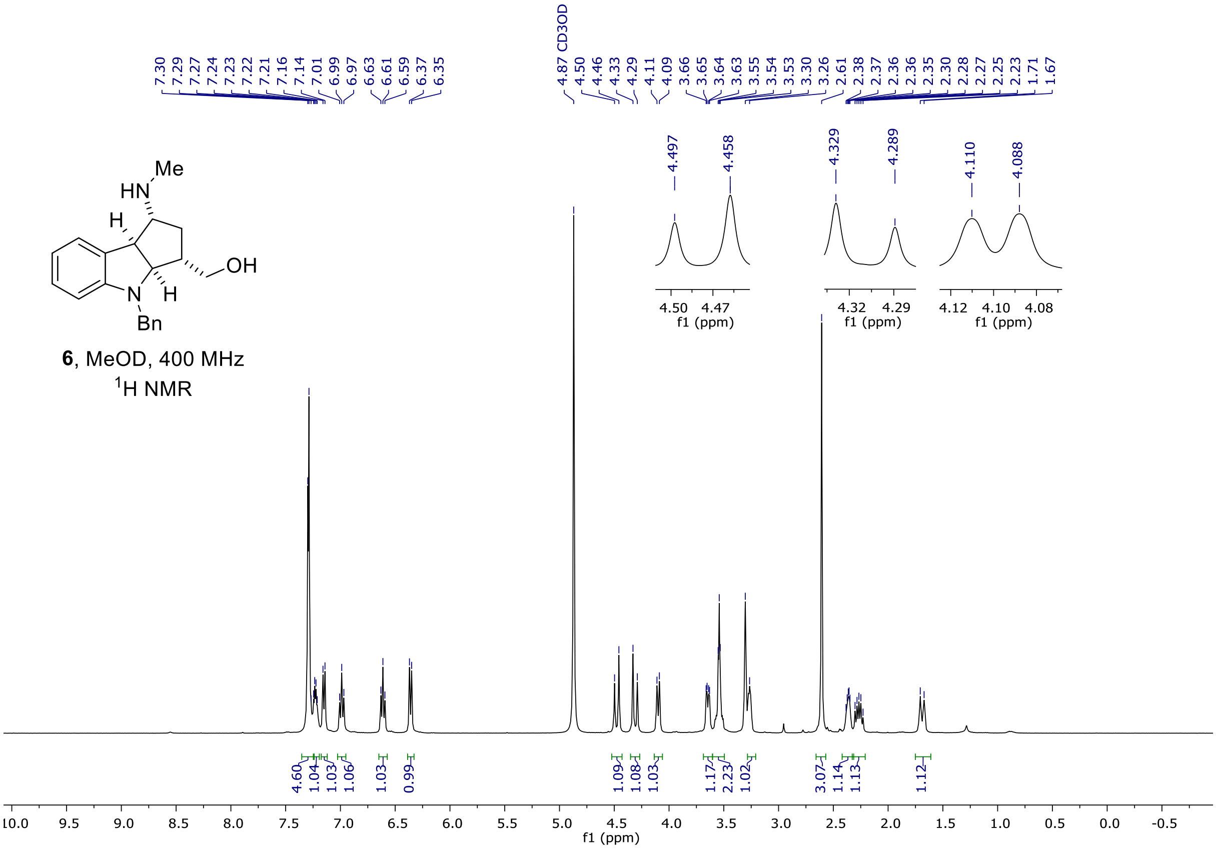
HN
Me
H
OH
N
H
Bn
6, MeOD, 400 MHz
^{1}H NMR
4.87 CD3OD
4.497
4.458
4.329
4.289
4.110
4.088
f1 (ppm)

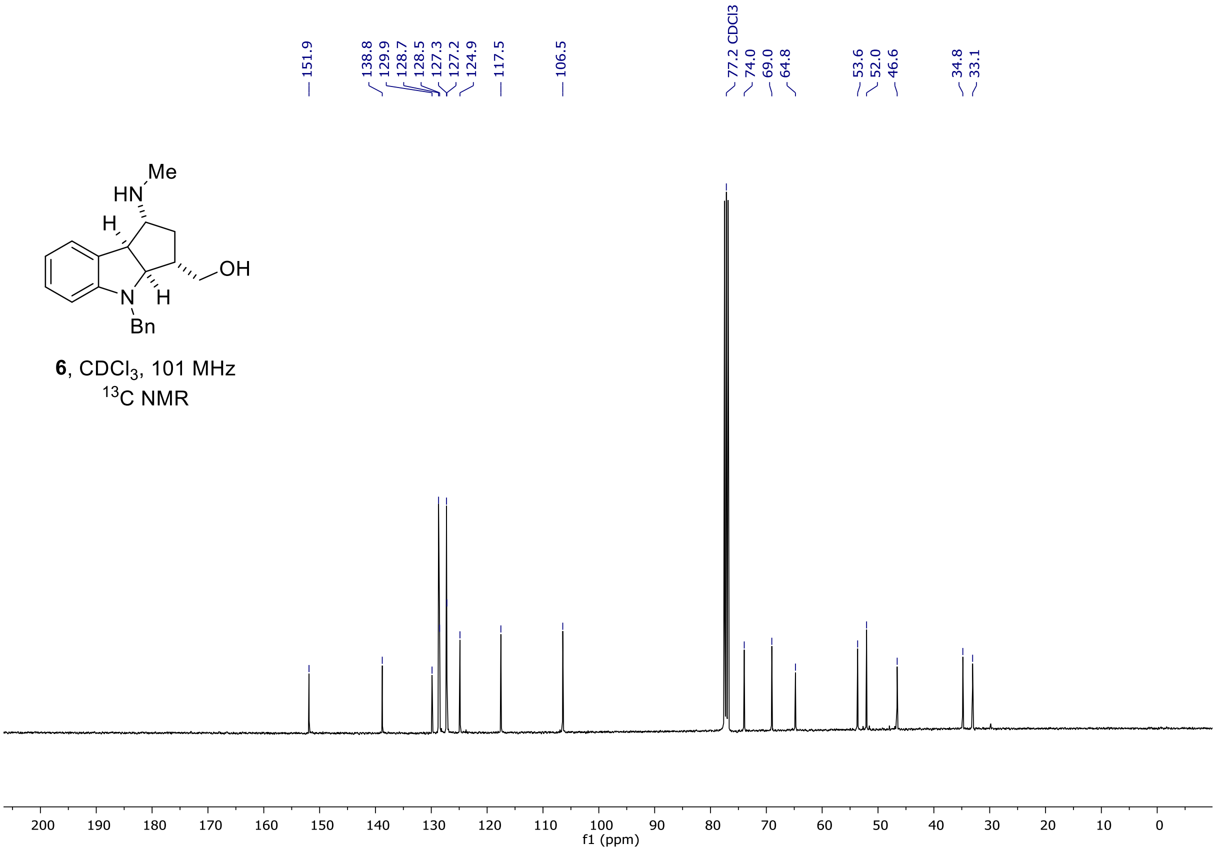
HN
Me
H
OH
N
H
Bn
6, CDCl$_3$, 101 MHz
^{13}C NMR
151.9
138.8
129.9
128.7
128.5
127.3
127.2
124.9
117.5
106.5
77.2 CDCl3
74.0
69.0
64.8
53.6
52.0
46.6
34.8
33.1
f1 (ppm)

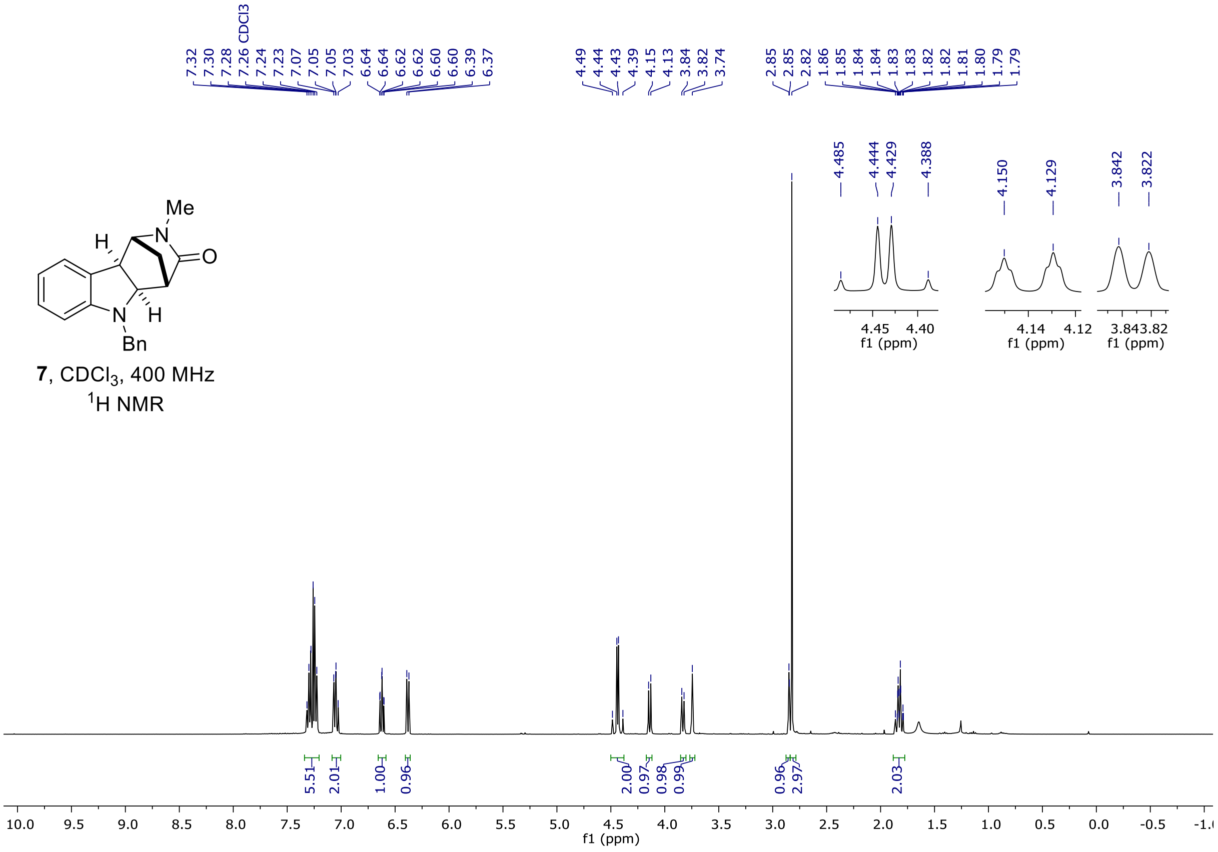

7, $CDCl_3$, 400 MHz
^1H NMR

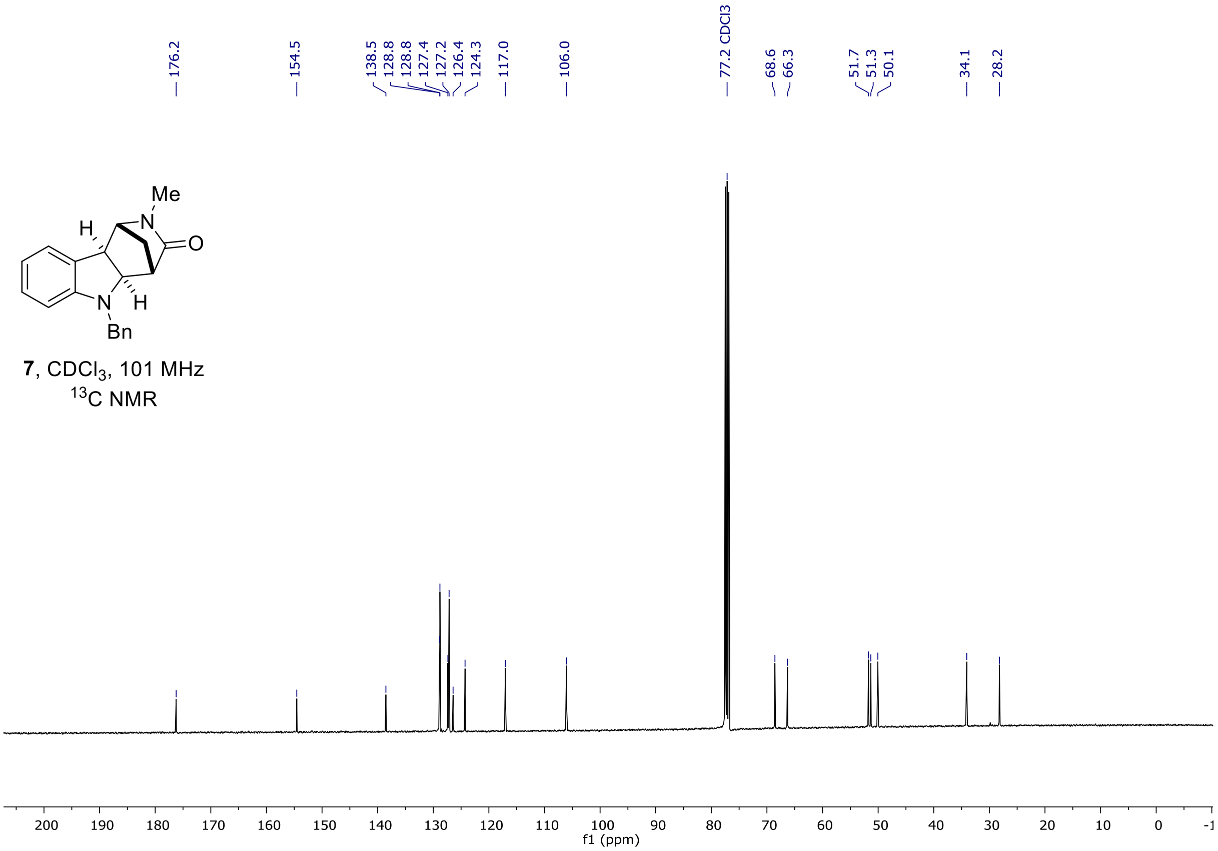

7, $CDCl_3$, 101 MHz
^{13}C NMR

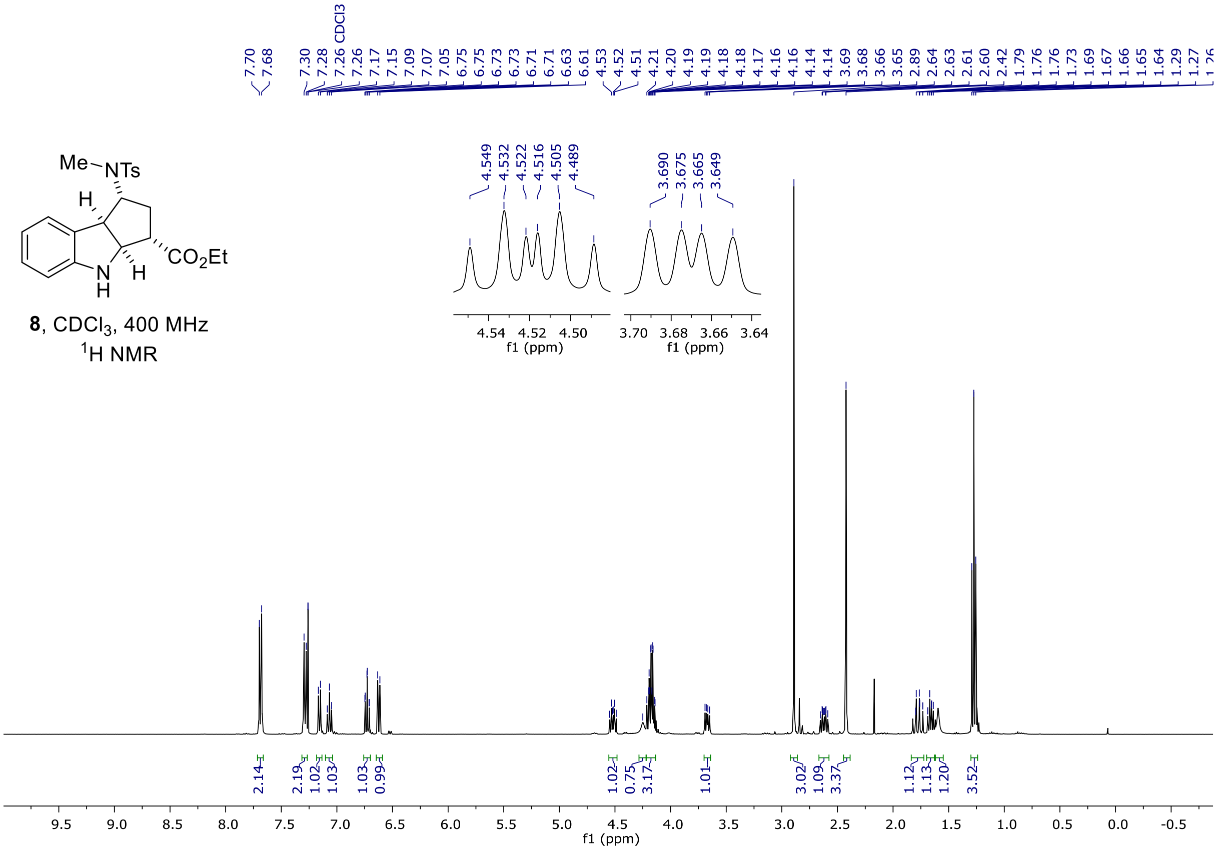

7.70
7.68
7.30
7.28
7.26 CDCl3
7.26
7.17
7.15
7.09
7.07
7.05
6.75
6.75
6.73
6.73
6.71
6.71
6.63
6.61
4.53
4.52
4.51
4.21
4.20
4.19
4.19
4.18
4.17
4.16
4.16
4.14
4.14
3.69
3.68
3.66
3.65
2.89
2.64
2.63
2.61
2.60
2.42
1.79
1.76
1.76
1.73
1.69
1.67
1.66
1.65
1.64
1.29
1.27
Me
NTs
H
N
H
H
CO2Et
8, CDCl3, 400 MHz
1H NMR
4.549
4.532
4.522
4.516
4.505
4.489
4.54 4.52 4.50
f1 (ppm)
3.690
3.675
3.665
3.649
3.70 3.68 3.66 3.64
f1 (ppm)
2.14
2.19
1.02
1.03
1.03
0.99
1.02
0.75
3.17
1.01
3.02
1.09
3.37
1.12
1.13
1.20
3.52
9.5 9.0 8.5 8.0 7.5 7.0 6.5 6.0 5.5 5.0 4.5 4.0 3.5 3.0 2.5 2.0 1.5 1.0 0.5 0.0 -0.5
f1 (ppm)

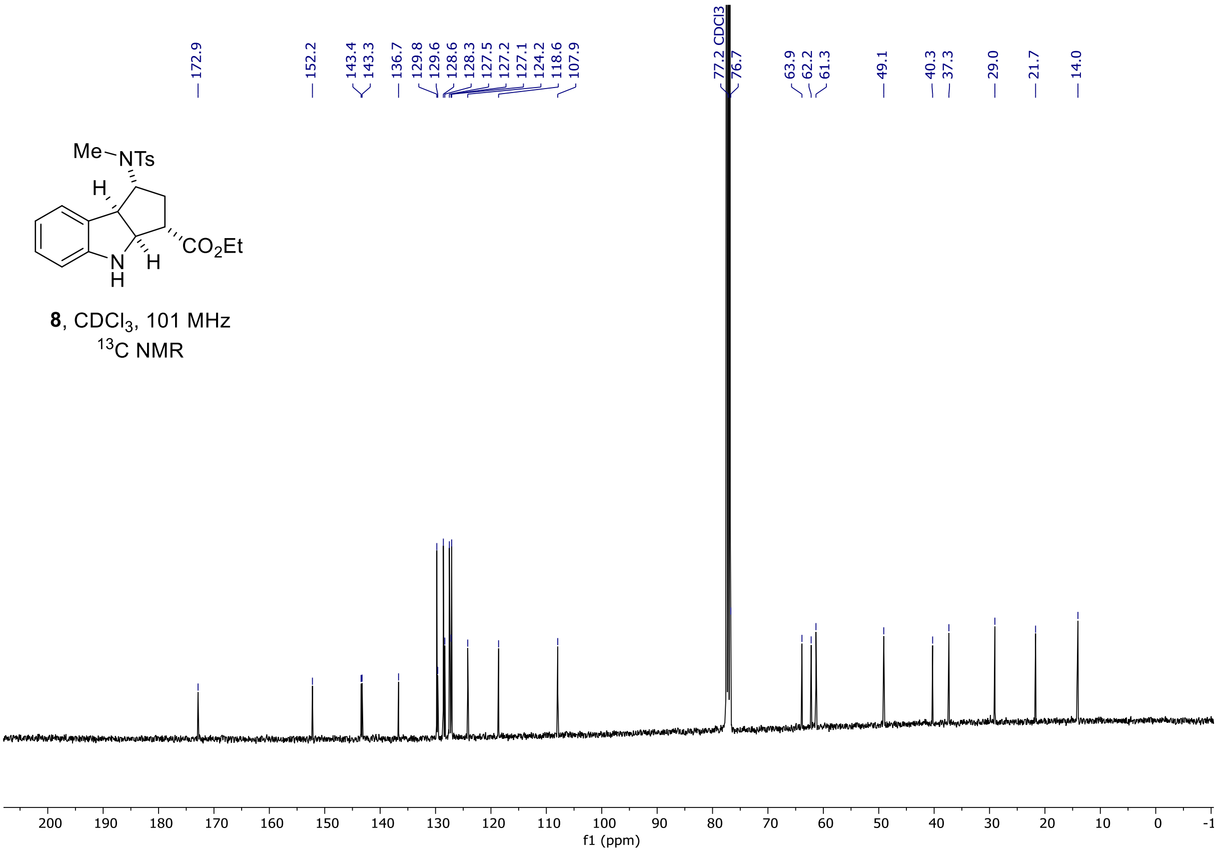

172.9
152.2
143.4
143.3
136.7
129.8
129.6
128.6
128.3
127.5
127.2
127.1
124.2
118.6
107.9
77.2 CDCl3
76.7
63.9
62.2
61.3
49.1
40.3
37.3
29.0
21.7
14.0
Me
NTs
H
N
H
H
CO2Et
8, CDCl3, 101 MHz
13C NMR
200 190 180 170 160 150 140 130 120 110 100 90 80 70 60 50 40 30 20 10 0 -1
f1 (ppm)
